# Efficient mucin *O-*glycan degradation by specific mucin degrading intestinal bacteria: towards understanding enzyme-glycan interactions

**DOI:** 10.1093/glycob/cwag004

**Published:** 2026-01-12

**Authors:** Carol de Ram, Maryse D Berkhout, Marta Kozioł, Laura Blasco Matias, Cynthia Klostermann, Carolina O Pandeirada, Sjef Boeren, Athanasia Ioannou, Jean-Paul Vincken, Clara Belzer, Henk Schols

**Affiliations:** Laboratory of Food Chemistry, Wageningen University & Research, Bornse Weilanden 9, 6708 WG Wageningen, the Netherlands; Laboratory of Microbiology, Wageningen University & Research, Stippeneng 4, 6708 WE Wageningen, the Netherlands; Laboratory of Food Chemistry, Wageningen University & Research, Bornse Weilanden 9, 6708 WG Wageningen, the Netherlands; Laboratory of Food Chemistry, Wageningen University & Research, Bornse Weilanden 9, 6708 WG Wageningen, the Netherlands; Laboratory of Food Chemistry, Wageningen University & Research, Bornse Weilanden 9, 6708 WG Wageningen, the Netherlands; Laboratory of Food Chemistry, Wageningen University & Research, Bornse Weilanden 9, 6708 WG Wageningen, the Netherlands; Laboratory of Biochemistry, Wageningen University & Research, Stippeneng 4, 6708 WE Wageningen, the Netherlands; Laboratory of Microbiology, Wageningen University & Research, Stippeneng 4, 6708 WE Wageningen, the Netherlands; Laboratory of Food Chemistry, Wageningen University & Research, Bornse Weilanden 9, 6708 WG Wageningen, the Netherlands; Laboratory of Microbiology, Wageningen University & Research, Stippeneng 4, 6708 WE Wageningen, the Netherlands; Laboratory of Food Chemistry, Wageningen University & Research, Bornse Weilanden 9, 6708 WG Wageningen, the Netherlands

**Keywords:** *A. muciniphila*, *B. thetaiotaomicron*, enzymatic cleavage, glycan degradation, *R. torques*

## Abstract

Intestinal mucin glycan-degrading bacteria are important for mucus turnover, stimulating mucus production, and producing beneficial metabolites. The mucin-degrading bacteria require various enzymes to break down mucin *O*-glycans. In this study, mucin glycan-degrading bacteria *Akkermansia muciniphila*, *Ruminococcus torques*, and *Bacteroides thetaiotaomicron*, were grown on porcine gastric mucin in monocultures, co-cultures, and a synthetic bacterial community. Enzyme extracts from these cultures were incubated with a selection of glycans, varying in sugar and linkage composition, to investigate enzyme specificities. Proteomics identified β-galactosidases, α-*N*-acetylgalactosaminidases, β-*N*-acetylglucosaminidases, α-fucosidases, α-sialidases, sulphatases, carbohydrate esterases, and polysaccharide lyases involved in *O*-glycan degradation. Enzymes produced by *A. muciniphila* and *R. torques* efficiently cleaved β-linked galactose and *N*-acetylgalactosamine. *B. thetaiotaomicron* enzymes minimally cleaved mucin glycans although multiple β-galactosidases and β-*N*-acetylglucosaminidases were produced. *A. muciniphila* favoured removal of fucose linked to non-terminal sugars whereas *R. torques* and *B. thetaiotaomicron* favoured removal of fucose linked to terminal sugars. *A. muciniphila* enzymes favoured cleavage of fucose α1–2 linked over α1–3 linked and cleavage of *N*-acetylglucosamine β1–3 linked over β1–4 linked. Both *A. muciniphila* and *B. thetaiotaomicron* favoured cleavage of galactose β1–4 linked over β1–3 linked and sialic acid α2–3 linked over α2–6 linked. Removal of sulphate from mucin structures was only observed by *B. thetaiotaomicron*. Bacterial co-cultures and the synthetic community produced all enzymes identified in the monocultures resulting in efficient mucin *O*-glycan degradation. Combining proteomics and glycan linkage cleavage by bacterial enzymes, showed differences in glycan degradation by the bacteria. This highlighted the importance of intestinal bacterial composition in mucin glycan degradation.

## Introduction

The intestinal epithelial surface is covered by a mucus layer that functions as a physical barrier protecting the intestinal epithelium (Berkhout et al. 2022; Song et al. 2023). The colonic mucus layer is organised as a firmly attached impenetrable inner layer, held in place by goblet cells, and a loosely attached thicker outer layer (Johansson et al. 2011; Song et al. 2023). The constantly renewed inner layer is converted by endogenous proteases into the outer layer, which is an important niche for intestinal bacteria (Johansson et al. 2008; Paone and Cani 2020). Among the intestinal bacterial community, specific mucin glycan-degrading bacteria can remove and utilise mucin glycans (McGuckin et al. 2011; Cornick et al. 2015). The mucin glycan monomers are utilised by the bacteria as a carbon source for growth and for the production of metabolites, including short-chain fatty acids (SCFAs), which have a beneficial impact on the host (Morrison and Preston 2016; Wardman et al. 2022).

To utilise the highly complex and diverse mucin *O*-glycan structures, mucin glycan-degrading bacteria encode an extensive repertoire of carbohydrate-active enzymes (CAZymes), including glycoside hydrolyses (GHs) and sulphatases, to cleave off glycosyl units (Tailford et al. 2015; Belzer 2022). To remove terminal substituents, specific GH families and sulphatases are employed by the bacteria (Raba and Luis 2023). For example, removal of fucose (Fuc) substituents requires production of fucosidases belonging to GH family 29 (GH29) and/or GH95 (Glover et al. 2022; Wu et al. 2023). Removal of end-capping sialic acid (Sia) can be performed with hydrolytic sialidases and trans-sialidases, mostly found in the GH33 family (Juge et al. 2016; Raba and Luis 2023). Sulphate groups are removed from mucin glycans by sulphatases directly or via enzymes that can remove glycosyl residues with the sulphate group still attached (Katoh et al. 2017; Raba and Luis 2023). After removal of terminal substituents, additional GHs are required to further break down the glycan chains. These GHs include β-*N-*acetylglucosaminidases (GH20, GH84, GH85, and GH89), β-galactosidases (GH2, GH35, GH42, and GH98), α-*N-*acetylgalactosaminidases (GH101 and GH129), and endo-acting *O-*glycanases (GH16) (Tailford et al. 2015; Crouch et al. 2020; Glover et al. 2022; Takada et al. 2023; Bakshani et al. 2025). Complete glycan degradation and utilisation is a cooperative action between mucin glycan-degrading bacteria and cross-feeders (D’Souza et al. 2018; Berkhout et al. 2022; Raba and Luis 2023).

Mucin glycan-degrading bacteria commonly present in the human gastrointestinal tract (GIT) include *Akkermansia muciniphila*, *Ruminococcus torques*, and *Bacteroides thetaiotaomicron*. *A. muciniphila* and *R. torques* are mucin glycan-degrading specialists that thrive in the mucosal environment. *B. thetaiotaomicron*, on the other hand, is a glycan-degrading generalist. It is able to utilise mucin glycans but its preference is for dietary glycans (Berkhout et al. 2024). Supporting information (SI) Table S1 shows an overview of recognised GH families present in *A. muciniphila*, *R. torques*, and *B. thetaiotaomicron*. A co-culture of *A. muciniphila* and *B. thetaiotaomicron* was reported to exhibit higher growth rates and metabolic production, and increased expression of CAZymes compared to monoculture *A. muciniphila* or *B. thetaiotaomicron* (Kostopoulos et al. 2021). Increased growth of *B. thetaiotaomicron* was also reported when *R. torques* and *B. thetaiotaomicron* were grown in co-culture as *B. thetaiotaomicron* utilises *O-*glycan degradation products from *R. torques* (Schaus et al. 2024). *A. muciniphila*, on the other hand, grew poorly on *O-*glycan degradation products from *R. torques,* indicating that *A. muciniphila* has a preference for mucin *O-*glycans still attached to the protein backbone (Schaus et al. 2024). These studies stress the importance of understanding the interactions between bacteria in monocultures and co-cultures and their impact on mucin glycan degradation via enzyme-glycan interactions.

This research aims to study the activity of enzymes produced by intestinal mucin glycan-degrading bacteria on degradation of mucin *O*-glycans. Therefore, *A. muciniphila*, *R. torques*, and *B. thetaiotaomicron*, in monocultures, co-cultures, and as part of a mucin-degrading synthetic community (MDSC), were grown on porcine gastric mucin (PGM) and the resulting samples were sonicated. Proteomics was performed on the bacterial lysates to identify the enzymes produced. The bacterial lysates were also incubated with a selection of neutral core human milk oligosaccharides (HMOs), fucosylated (neutral) HMOs, and sialylated (acidic) HMOs as well as sulphated glycans. These glycans were selected as reference components for their structural similarity to mucin *O-*glycans and their known linkage types. This assisted in understanding glycosidic linkage specificities of the enzymes and glycan-bacterial interactions. Moreover, the bacterial lysates containing enzymes produced, were incubated with *O-*glycans chemically released from PGM and bovine submaxillary glands mucin (BSM) to study the specificity of the bacterial enzymes towards mucin *O-*glycan linkages. This study takes a step forward in understanding enzyme specificity related to mucin glycan linkage cleavage by mucin glycan-degrading bacteria.

## Results

Bacterial lysates of *A. muciniphila, R. torques,* and *B. thetaiotaomicron* in monocultures, co-cultures, and the mucin-degrading synthetic community (MDSC) after incubation with porcine gastric mucin (PGM) were studied regarding their enzyme production and the glycan linkage specificities of the enzymes produced. All cultures showed growth on PGM (Figs. S1 and S2) and compositional analysis of bacterial co-cultures showed that after 12 h incubation on PGM each co-culture was a mixture of the species present at 0 h (Fig. S3). The relative abundance of *A. muciniphila* was more than 40% after 12 and 24 h incubation on PGM in each of the co-cultures containing *A. muciniphila*.

### Analysis of mucin glycan degrading enzymes expressed by monocultures, co-cultures, and the synthetic community

**Figure 1:**
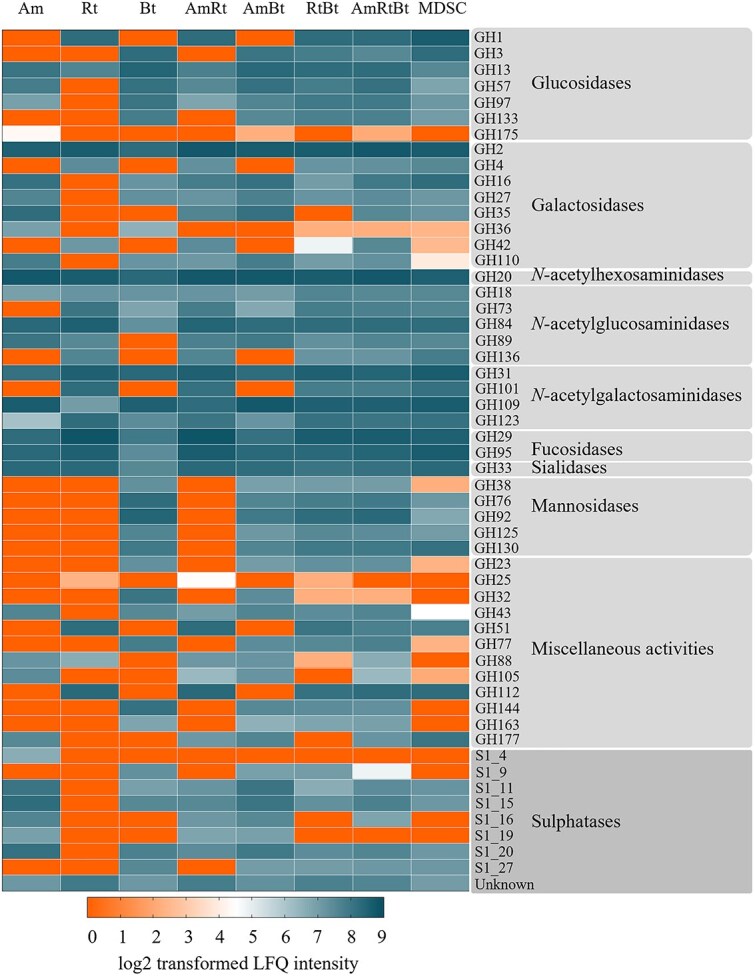
Heatmap representing glycoside hydrolyse (GH) and sulphatase as identified by proteomics produced by monocultures *A. muciniphila* (Am), *R. torques* (Rt), *B. thetaiotaomicron* (Bt), co-cultures thereof (AmRt, AmBt, RtBt, and AmRtBt), and the MDSC. Results are based on proteomic analysis (triplicate) of bacterial lysates resulting from cultures grown on PGM for 24 h. the values in the heatmap are the mean of log2 transformed label free quantification (LFQ) intensities of the sum of enzymes that belong to a certain enzyme family. GHs are grouped by associated enzyme activity based on literature (Montanier et al. 2009; Ficko-Blean et al. 2012; Etzold et al. 2014; Nakamura et al. 2017; Drula et al. 2022; Wardman et al. 2022; Labourel et al. 2023). The scale ranges from 0–9 indicating enzyme expression. 0 (orange) indicates enzyme not expressed and 9 (dark blue) indicates enzyme expressed.

The bacterial lysates, containing the produced enzymes, after 24 h growth on PGM were subjected to proteomics to identify CAZymes produced by the bacteria and involved in mucin glycan degradation. An overview of the GH and sulphatase enzyme families identified in this study is shown in Fig. 1. The identified genes and activities for each GH and sulphatase family are summarised in Table 1. Identified carboxyl esterases (CEs), polysaccharides lyases (PLs), glycosyltransferases (GTs), auxiliary activity enzymes (AAs), and carbohydrate-binding modules (CBMs) are summarised in Table S2. Each of the monocultures produced enzymes from GH families involved in mucin glycan degradation, however, differences in abundance and GH types were observed among the species. The co-cultures produced all CAZymes as produced by the monocultures. Co-culture *A. muciniphila*/*R. torques*/*B. thetaiotaomicron* produced the broadest range of GHs and sulphatases (Fig. 1). The MDSC produced a broad range of enzymes as well. The enzymes produced by the MDSC were produced mostly by *A. muciniphila*, *Bacteroides fragilis*, *B. thetaiotaomicron*, *R. torques*, and *Phocaeicola vulgatus* (Table S3). For example, GH16 and GH35 enzymes were produced by *A. muciniphila* and *B. fragilis*, GH42 enzymes by *R. torques*, GH18 enzymes by *A. muciniphila* and *B. thetaiotaomicron*, GH84 and GH89 enzymes by *A. muciniphila* and *R. torques*, GH29 enzymes by *B. thetaiotaomicron*, GH95 enzymes by *A. muciniphila*, *R. torques*, and *B. thetaiotaomicron*, and GH33 enzymes by *A. muciniphila*, *R. torques*, and *P. vulgatus*. Multiple sulphatases (acting on 3S, 4S, and 6S) were produced by the MDSC by *A. muciniphila*, *B. fragilis*, and *B. thetaiotaomicron* (Table S3). Proteomics elucidated enzyme families produced by *A. muciniphila*, *R. torques*, and *B. thetaiotaomicron* and highlighted the differences in enzyme production (Fig. 1 and Table 1).

### Degradation of neutral core HMOs LNH and LNnT by enzymes produced by *A. muciniphila*, *R. torques*, *B. thetaiotaomicron*, and the MDSC

The bacterial lysates of the monocultures and the MDSC after 12 h growth on PGM were incubated for 24 h with reference glycans containing mucin relevant glycan substituents and linkages. The reference glycans included neutral core HMOs lacto-*N-*hexaose (LNH; Galβ1–4GlcNAcβ1–6(Galβ1–3GlcNAcβ1–3)Galβ1–4Glc) and lacto-*N-*neotetraose (LNnT; Galβ1–4GlcNAcβ1–3Galβ1–4Glc), fucosylated (neutral) HMOs difucosyllactose (DF-L; Fucα1–2Galβ1–4(Fucα1–3)Glc) and lacto-*N-*fucopentaose I (LNFPI; Fucα1–2Galβ1–3GlcNAcβ1–3Galβ1–4Glc), and sialylated (acidic) HMOs LS-tetrasaccharide a (LSTa; Siaα2–3Galβ1–3GlcNAcβ1–3Galβ1–4Glc) and LS-tetrasaccharide c (LSTc; Siaα2–6Galβ1–4GlcNAcβ1–3Galβ1–4Glc). Furthermore, sulphated glycans, namely 6S-GlcNAc, 3S6S-GlcNAc, and 6′-*O*-sulphated Lewis a, were included.

**Table 1:** Overview of enzyme classes glycoside hydrolyses (GHs) and sulphatases identified using proteomics of bacterial lysates of *A. muciniphila* (Am), *R. torques* (Rt), and *B. thetaiotaomicron* (Bt) grown on porcine gastric mucin (PGM) for 24 h. per CAZyme family the identified genes of which proteomics identified enzymes for the different bacteria are indicated and their associated activity based on literature (Luis et al. 2021; Drula et al. 2022; Stam et al. 2023; Schaus et al. 2024; Bakshani et al. 2025; Bateman et al. 2025; Ndeh et al. 2025).

Enzyme class and function	Family number	Am		Rt		Bt	
GH α- and β-glucosidases	GH1			RUMTOR_00102	Glcβ1–4		
				BglH	Glcβ1–4		
	GH3					BT_3314	Glcβ1-X
						BT_3567	Glcβ1-X
	GH13	Amuc_1637	Unknown	RUMTOR_00751	Unknown	BT_4689	Glcα1–6
		Amuc_1812	Glcα1–4	RUMTOR_02566	Unknown	BT_1663	Glcα1–6
		Amuc_1751	Glcα1–4	RUMTOR_00566	Glcα1–6	BT_0773	Unknown
				RUMTOR_01473	Unknown	BT_4690	Glcα1–4
						BT_0771	Glcα1–4
	GH57	Amuc_1868	Glcα1–4			BT_4305	Glcα1–4
	GH97	Amuc_1420	Unknown			BT_1871	Glcα1-X
						susB	Glcα1-X
						BT_3294	Glcα1-X
						BT_2620	Glcα1-X
	GH133					BT_4303	Glcα1–6
	GH175	Amuc_1260	Unknown				
GH α- and β-galactosidases	GH2	Amuc_0539	Galβ1–4Gal/GlcNAc	RUMTOR_02191	Galβ1–3/4Gal	BT_0461	Galβ1-X
		Amuc_0824	Galβ1–3X	RUMTOR_02465	Galβ1–3/4Gal	BT_3340	Galβ1-X
		Amuc_1686	Galβ1–3X Galβ1–6Gal/GalNAc			BT_4241	Galβ1-X
		Amuc_0290	Galβ1-X			BT_0458	Manβ1-X
		Amuc_0539	Galβ1–4Gal/GalNAc				
		Amuc_1667	Galβ1–3GalNAc Galβ1–4Gal/Glc				
	GH4			RUMTOR_02460	Galα1-X		
	GH16	Amuc_2108	Endo-*O*-glycanase - Poly LacNac/Galβ1–4GlcNAc			BT_2824	*O*-glycan endo Galβ1–4X
		Amuc_0724	Endo-*O*-glycanase - Poly LacNac/Galβ1–4GlcNAc				
		Amuc_0875	Endo-*O*-glycanase - Poly LacNac/Galβ1–4GlcNAc				
	GH27	Amuc_1187	Galα1-X			BT_2662	Galα1-X
	GH35	Amuc_1686	Galβ1–3X Galβ1–6Gal/GalNAc				
		Amuc_0771	Galβ1–3X Galβ1–4Glc/GlcNAc				
	GH36	Amuc_0855	Galα1-X			BT_2851	Galα1-X
	GH42			RUMTOR_02570	Galβ1–3/4Gal		
	GH110	glaA	Galα1–3X			glaB	Galα1–3X
		glaB	Galα1–3X				
GH *N*-acetylhexos-aminidases	GH20	Amuc_1815	GalNAcβ1–3Gal	RUMTOR_01268	HexNAcβ1-X	BT_3868	GlcNAcβ1-X
		Amuc_0868	GlcNAcβ1-X/ HexNAcβ1-X	RUMTOR_01685	HexNAcβ1-X	BT_0459	GlcNAcβ1-X
		Amuc_2019	GlcNAcβ1-X/ HexNAcβ1-X	RUMTOR_02581	HexNAcβ1-X	BT_0456	HexNAcβ1-X
		Amuc_2136	GlcNAcβ1-X/ HexNAcβ1-X	RUMTOR_02806	HexNAcβ1-X	BT_0506	HexNAcβ1-X
		Amuc_0369	GlcNAcβ1-X/ HexNAcβ1-X			BT_1051	HexNAcβ1-X
		Amuc_1669	GlcNAcβ1–3Gal			BT_4394	HexNAcβ1-X
		Amuc_1924	GalNAcβ1-X/ HexNAcβ1-X			BT_4337	HexNAcβ1-X
		Amuc_1032	HexNAcβ1-X				
GH β-GlcNAc	GH18	Amuc_2164	GlcNAcβ1-X	RUMTOR_02665	Unknown	BT_1285	GlcNAcβ1-X
	GH73			RUMTOR_00705	GlcNAcβ1–4	BT_1538	GlcNAcβ1–4
	GH84	Amuc_0052	GlcNAcβX	nagH	Unknown	BT_4395	Endo GlcNAcβ1–4
	GH89	Amuc_1220	GlcNAcβ1-X	RUMTOR_00787	GlcNAcβ1-X		
		Amuc_0060	GlcNAcβ1-X				
	GH136			RUMTOR_00181	Unknown		
GH α- and β-GalNAc	GH31	Amuc_1008	Exo-α-GalNAc			BT_3169	Exo-α-GalNAc
	GH101			RUMTOR_01594	Endo-α-GalNAc		
	GH109	Amuc_0017	GalNAcα1–3Gal	RUMTOR_00774	Oxidoreductase	BT_4243	Endo-α-GalNAc
		Amuc_0920	GalNAcα1–3Gal			BT_4252	Oxidoreductase, NAD-bin
						BT_2158	Dehydrogenase
						BT_3470	Dehydrogenase
	GH123	Amuc_0803	GalNAcβ1-X	RUMTOR_01211	Unknown	BT_2751	Neuraminidase like protein
				RUMTOR_01966	Unknown		
GH α-fucosidases	GH29	Amuc_0010	Fucα1–2Lac/LacNAc	RUMTOR_02528	Fucα1–3/6X	BT_4713	Fucα1-X
		Amuc_0392	Fucα1-X			BT_3665	Fucα1-X
						BT_1842	Fucα1-X
						BT_1625	Fucα1-X
	GH95	Amuc_0186	Fucα1–2	RUMTOR_01294	Fucα1-X	BT_4682	Fucα1-X
		Amuc_1120	Fucα1–2	RUMTOR_02288	Fucα1-X		
GH α-sialidases	GH33	Amuc_1835	Siaα2-X	RUMTOR_00151	Unknown	BT_0455	Siaα2-X
		Amuc_0625	Siaα2-X	RUMTOR_01193	Unknown		
				RUMTOR_01211	Unknown		
GH α- and β-mannosidases	GH38					BT_3774	Manα1-X
	GH76					BT_3792	Manα1–6
						BT_3782	Unknown
						BT_3521	Manα1–6
						BT_2623	Manα1–6
						BT_1883	Manα1–6
	GH92					BT_3994	Manα1–2
						BT_3991	Manα1–2
						BT_3990	Manα1–2
						BT_3965	Manα1–2
						BT_3962	Unknown
						BT_3963	Unknown
						BT_3784	Manα1–2
						BT_3773	Manα1–2
						BT_3527	Manα1–2
						BT_2629	Manα1–2
						BT_1878	Manα1–2
						BT_1769	Manα1–2
						BT_1032	Manα1–2
	GH125					BT_3781	Unknown
	GH130					BT_1033	Manβ1–4
						BT_3780	Manβ1–2
GH Miscellaneous activities	GH23					BT_4487	Peptidoglycan lytic transglycosylase
						BT_3999	Transglycosidase
	GH25			RUMTOR_01337	Unknown		
				RUMTOR_01567	Unknown		
		GH32				BT_3082	Sucrose β2–1-fructosidase
						BT_1760	
						BT_1795	Sucrose β2–1-fructosidase
		GH43	Amuc_0698	Important for growth on mucin		BT_3516	Arabinan endo-α-1, 5-arabinofurano-sidase
			Amuc_0697	Important for growth on mucin		BT_3515	Putative glycosylhydrolyse
						BT_3467	Putative glycosylhydrolyse
		GH51		RUMTOR_01174	Unknown		
		GH77				BT_3348	Unsaturated glucuronyl hydrolase
						BT_4658	Glucuronyl hydrolase
		GH88	Amuc_1621	4-α-glucano-transferase	malQ	4-α-glucano-transferase	
		GH105	Amuc_0863	Unsaturated rhamnogalacturonyl hydrolase			
		GH112		gnpA	β1–3-galactosyl-*N*-HexNAc-phosphorylase		
		GH144				BT_3566	Glycoamylase-like
		GH163				BT_1035	Coagulation factor 5/8 type
	GH177	Amuc_2166	Unknown				
		Amuc_1216	Unknown				
Sulphatases	S1_4	Amuc_0565	Unknown				
	S1_9				BT_1596	2-*O*-S-Hex	
	S1_11	Amuc_1033	6-*O*-S-GlcNAc		BT_1628	6-*O*-S-GlcNAc	
		Amuc_1074	6-*O*-S-GlcNAc		BT_4656	6-*O*-S-Gal/GalNAc	
	S1_15	Amuc_0121	6-*O*-S-GlcNAc		BT_1624	6-*O*-S-Gal/GalNAc	
					BT_3333	Exo 6-*O*-S-GalNAc	
	S1_16	Amuc_1655	4-*O*-S-Gal/GalNAc				
		Amuc_1755	4-*O*-S-Gal/GalNAc				
	S1_19	Amuc_1182	Unknown				
	S1_20	Amuc_0451	6-*O*-S-GlcNAc		BT_1636	3-*O*-S-Gal	
		Amuc_0491	3-*O*-S-Gal				
		Amuc_0953	3-*O*-S-Gal				
	S1_27				BT_3349	4-*O*-S-GalNAc	
	Unknown	Amuc_1118	Unknown	RUMTOR_01190	Arylsulphatase	BT_0412	Na+/sulphate symporter
		Amuc_2022	Unknown			BT_1853	Unknown
						BT_3177	6-*O*-S-GlcNAc

**Figure 2:**
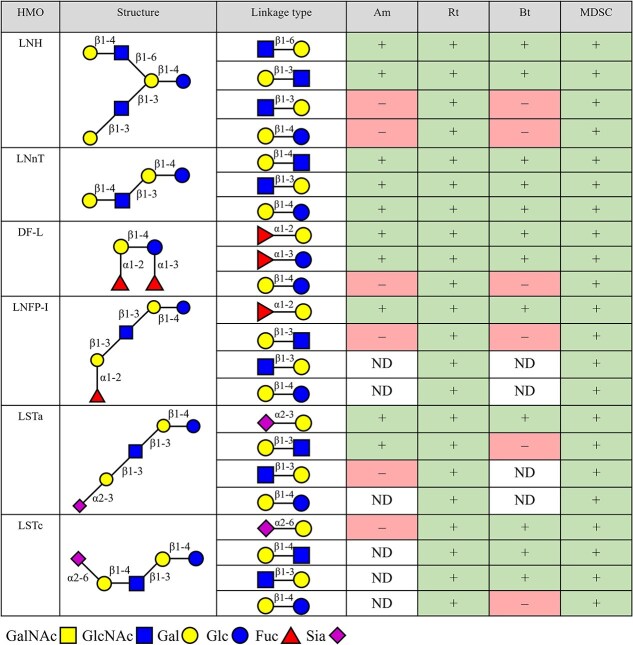
Overview of the degradation of tested neutral core HMOs, fucosylated (neutral) HMOs, and sialylated (acidic) HMOs by enzymes of bacterial lysates from *A. muciniphila* (Am), *R. torques* (rR), *B. thetaiotaomicron* (Bt), and the mucin-degrading synthetic community (MDSC) after growth on porcine gastric mucin (PGM) for 12 h. degradation is expressed by cleavage of linkages present in the HMOs measured by formation and degradation of intermediate products. Linkage cleavage within 24 h is indicated with “+,” linkage not cleaved within 24 h is indicated with “–,” and no formation of the intermediate is indicated with “not detected (ND)”. This overview is based on results obtained from HPAEC analysis, the chromatograms of which are shown in Fig. S5.

An overview of the linkages cleaved during the various incubations is shown in Fig. 2 and HPAEC chromatograms illustrating HMO breakdown are shown in Fig. S5A – B. The monocultures and the MDSC degraded both LNH and LNnT (Fig. 2). The structures of HMOs and formed intermediates are shown in Fig. S4A, B, and  E. Degradation of LNH mainly resulted in the formation of LNT (Galβ1–3GlcNAcβ1–3Galβ1–4Glc), LNT2 (GlcNAcβ1–3Galβ1–4Glc), and lactose (Galβ1–4Glc), whereas degradation of LNnT mainly resulted in LNT2 and lactose. Enzymes from *A. muciniphila* lysate further degraded the formed LNT and LNT2 but lactose was not degraded within 24 h. Enzymes from *R. torques* lysate showed little to no intermediary LNT or LNT2 formation following LNH or LNnT degradation, suggesting a rapid break down of these intermediate products. Lactose formed was also rapidly degraded. Enzymes from *B. thetaiotaomicron* lysate further degraded the formed LNT2 but LNT and lactose were not degraded within 24 h. Enzymes from the MDSC lysate degraded all formed intermediate products.

### Degradation of fucosylated (neutral) HMOs DF-L and LNFP-I by enzymes produced by *A. muciniphila*, *R. torques*, *B. thetaiotaomicron*, and the MDSC

The monocultures and the MDSC degraded both DF-L and LNFP-I (Fig. 2, Fig. S4C and E, and Fig. S5C–D). Degradation of DF-L mainly resulted in the formation of 2’FL (Fucα1–2Galβ1–4Glc) and 3FL (Galβ1–4(Fucα1–3)Glc), whereas degradation of LNFP-I mainly resulted in the formation of LNT, LNT2, and lactose (Fig. S4A). Enzymes from *A. muciniphila* lysate degraded the formed 2’FL, but formed 3FL was not degraded within 24 h. Lactose formation was observed resulting from 2’FL degradation. LNT formed from degradation of LNFP-I was not further degraded by *A. muciniphila*. Enzymes from *B. thetaiotaomicron* lysate degraded both 2’FL and 3FL, resulting in formation of lactose, which was not degraded. Degradation of LNFP-I only resulted in formation of LNT, which was not broken down and accumulated. Enzymes from *R. torques* and the MDSC lysate degraded all intermediate products formed.

### Degradation of sialylated (acidic) HMOs LSTa and LSTc by enzymes produced by *A. Muciniphila*, *R. Torques*, *B. Thetaiotaomicron*, and the MDSC

The monocultures and the MDSC degraded LSTa, while LSTc was degraded by all except *A. muciniphila* (Fig. 2, Fig. S4D and E, and Fig. S5E–F). Degradation of LSTa mainly resulted in the formation of LNT, Sia, LNT2, and lactose, whereas degradation of LSTc mainly resulted in the formation of LNnT, Sia, and lactose. Enzymes from *A. muciniphila* lysate did not degrade LNT formed from LSTa, and LSTc was not degraded at all. Degradation by enzymes from *R. torques* lysate showed little to no intermediatory LNT or LNnT during degradation of LSTa or LSTc, suggesting a rapid break down of these intermediate products to lactose and monosaccharides. Enzymes from *B. thetaiotaomicron* lysate did not degrade LNT from LSTa further but LNnT formed from LSTc was degraded. Lactose was formed from LSTc degradation but not degraded. Enzymes from the MDSC lysate degraded all formed intermediate products.

### Degradation of sulphated glycans 6S-GlcNAc, 3S6S-GlcNAc, and 6′-O-sulphated Lewis a by produced enzymes from *A. Muciniphila*, *R. Torques*, *B. Thetaiotaomicron*, and the MDSC

**Figure 3:**
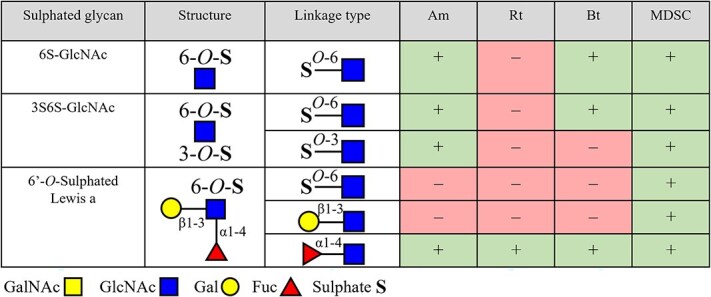
Overview of the degradation of tested sulphated glycans by enzymes of bacterial lysates from *A. muciniphila* (Am), *R. torques* (Rt), *B. thetaiotaomicron* (Bt), and the mucin-degrading synthetic community (MDSC) after growth on porcine gastric mucin (PGM) for 12 h. degradation is expressed by cleavage of linkages present in the sulphated glycans as measured by degradation of the sulphated glycans and formation of intermediate products. Linkage cleavage within 24 h is indicated with “+” and linkage not cleaved within 24 h is indicated with “–.” this overview is based on the obtained results from PGC-LC-MS/MS analysis of which the chromatograms are shown in Figs. S6–S21.

The degradation of sulphated glycans was followed by analysis with PGC-LC–MS/MS. Enzymes from *A. muciniphila* lysate cleaved 3- and 6-linked sulphate from 6S-GlcNAc and 3S6S-GlcNAc as indicated in Fig. 3. Structures and degradation products are illustrated in Fig. S4F–I. Cleavage of 6-linked sulphate from 6′-*O*-sulphated Lewis a by enzymes from *A. muciniphila* lysate was not observed, as shown in Fig. 3, although the sulphate group did not hinder cleavage of α1–4 linked Fuc from 6′-*O*-sulphated Lewis a (Fig. S6–S9). Enzymes from *R. torques* lysate did not cleave any of the sulphate linkages tested while again α1–4 linked Fuc could be cleaved off from 6′-*O*-sulphated Lewis a as shown in Fig. 3 and Fig. S10–S13. Enzymes from *B. thetaiotaomicron* lysate cleaved 6-linked sulphate from 6S-GlcNAc but cleavage of 3-linked sulphate (3S6S-GlcNAc) and 6-linked sulphate from 6′-*O*-sulphated Lewis a was not observed as indicated in Fig. 3 and Figs. S14–S17. The α1–4 linked Fuc was cleaved from 6′-*O*-dulphated Lewis a. Enzymes from the MDSC lysate cleaved 3- and 6-linked sulphate from 6S-GlcNAc, 3S6S-GlcNAc, and 6′-*O*-sulphated Lewis a as shown in Fig. 3 and Figs. S18–S21. Enzymes from the MDSC lysate degraded 6′-*O*-sulphated Lewis a by sequential cleavage of first α1–4 linked Fuc, followed by 6-linked sulphate, and then β1–3 linked Gal.

### Degradation of *O*-glycans released from PGM by enzymes present in bacterial lysates from *A. muciniphila*, *R. torques*, *B. thetaiotaomicron*, co-cultures, and the MDSC

**Figure 4:**
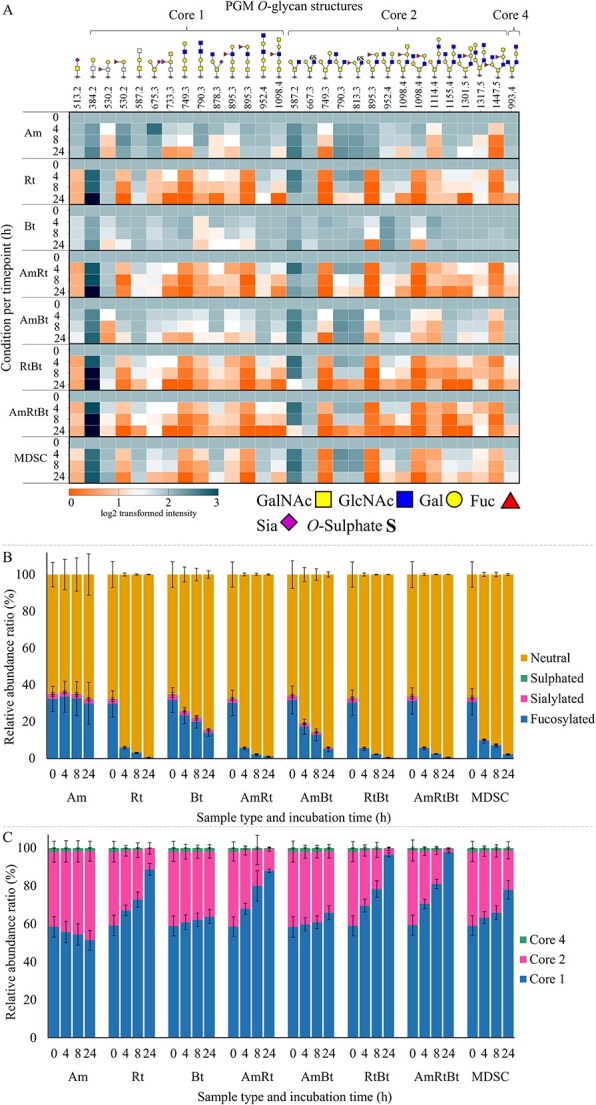
(A) Heatmap showing the degradation of *O-*glycans released from porcine gastric mucin (PGM) after 0, 4, 8, and 24 h incubation with bacterial lysates of *A. muciniphila* (Am), *R. torques* (Rt), *B. thetaiotaomicron* (Bt), co-cultures thereof (AmRt, AmBt, RtBt, and AmRtBt), and the MDSC after growth on PGM for 12 h (average of three replicates). The value at 0 h is set to 100% (log2 scale value 2) and the values at the following timepoints are shown relative to this value (log2 scale; 0 (orange) degraded and 2 or higher (blue) not degraded). (B) Bar graphs showing the relative amount of neutral, sulphated, sialylated, and fucosylated *O-*glycans released from PGM after 0, 4, 8, and 24 h incubation with bacterial lysates of Am, rt, Bt, AmRt, AmBt, RtBt, AmRtBt, and the MDSC after growth on PGM for 12 h. (C) Bar graphs showing the relative amount of core 1, core 2, core 3, and core 4 *O-*glycans released from PGM after 0, 4, 8, and 24 h incubation with bacterial lysates of Am, rt, Bt, AmRt, AmBt, RtBt, AmRtBt, and the MDSC after growth on PGM for 12 h.

To assess the degradation of mucin *O*-glycans by enzymes from *A. muciniphila, R. torques, B. thetaiotaomicron*, co-cultures thereof, and the MDSC, bacterial lysates of the cultures after 12 h incubation on PGM were incubated during 24 h with *O*-glycans released from PGM. The structures of the different *O*-glycans and the resulting PGM-derived *O*-glycan profiles after enzymatic digestion are shown in Fig. 4 and the related chromatograms and fragmentation data are shown in Figs. S22–S30. *O*-glycans with Galβ1–4GlcNAc, GlcNAcβ1–3Gal, and GalNAcα1–3Gal linkages were degraded by monocultures *A. muciniphila* and *R. torques* and co-cultures containing these bacteria. Core 1 *O*-glycan *m/z* 790.3 was degraded by all bacterial lysates, except for monoculture *A. muciniphila*. Core 4 *O*-glycan *m/z* 993.4 was only degraded by monoculture *R. torques* and co-cultures containing *R. torques*. Accumulation over time of *O*-glycans with *m/z* 384.2 (core 1) and *m/z* 587.2 (core 2) in all tested bacterial lysates suggested that these structures originated from cleavage of longer *O*-glycans. *O*-glycans with Fucα1–2Gal (e.g. core 1 *O*-glycan *m/z* 733.3) and Fucα1–3GlcNAc (e.g. core 1 *O*-glycan *m/z* 1098.4) were degraded by all tested bacterial lysates. Enzymes produced by *A. muciniphila* preferentially degraded fucosylated *O*-glycans with Fuc attached to non-terminal Gal or GlcNAc, whereas enzymes from *R. torques* and *B. thetaiotaomicron* preferentially degraded fucosylated *O*-glycans with Fuc attached to terminal Gal. Siaα2–3Gal linkages were degraded by all tested bacterial conditions (e.g. core 1 *O*-glycan *m/z* 878.3) and, additionally, *O*-glycans with Siaα2–6Gal linkages (e.g. core 1 *O*-glycan *m/z* 675.3) were degraded by all bacterial lysates, except for monoculture *A. muciniphila*. Sulphated core 2 *O*-glycan *m/z* 667.3 (6-linked sulphate) was only degraded by monoculture *B. thetaiotaomicron* and co-cultures containing *B. thetaiotaomicron*. Sulphated core 2 *O*-glycan *m/z* 813.3 (6-linked sulphate) was only degraded by *R. torques* and co-cultures containing *R. torques*. However, since we demonstrated no sulphate removal activity for *R. torques* this is expected to be the result from removal of results leading to degradation of this structure. *R. torques* was shown to be able to remove fucose from sulphated structures.

Enzymes from *A. muciniphila* preferentially degraded *O*-glycan structures with terminal Gal, enzymes from *R. torques* preferentially degraded *O*-glycan structures with terminal Gal or structures with Fuc or Sia, and enzymes from *B. thetaiotaomicron* preferred *O*-glycans with Fuc, Sia, or sulphate groups.

Enzymes produced by the co-cultures degraded all *O-*glycans that were degraded by enzymes from the monoculture lysates, reflecting a combination of the degradation patterns as observed by the monocultures (Fig. 4). The *O*-glycan degradation patterns observed in co-cultures containing *R. torques* show mostly similar patterns to *R. torques* in monoculture. Enzymes from the co-culture *A. muciniphila*/*R. torques*/*B. thetaiotaomicron* and the MDSC degraded similar *O*-glycans suggesting that *A. muciniphila*, *R. torques*, and *B. thetaiotaomicron* are sufficient for the degradation of mucin *O*-glycans present and the main contributors in the MDSC regarding *O*-glycan degradation. Mucin glycan degraders *Bacteroides caccae*, *B. fragilis*, and *Ruminococcus gnavus*, present in the MDSC, are also involved in *O*-glycan degradation as proteomics identified GH families involved in mucin glycan degradation. The other bacteria present in the MDSC have an important role in utilisation of the released glycans and producing beneficial SCFAs such as butyrate (de Ram et al. 2025).

### Degradation of *O*-glycans released from BSM by enzymes present in bacterial lysates from *A. muciniphila*, *R. torques*, *B. thetaiotaomicron*, co-cultures, and a bacterial community

**Figure 5:**
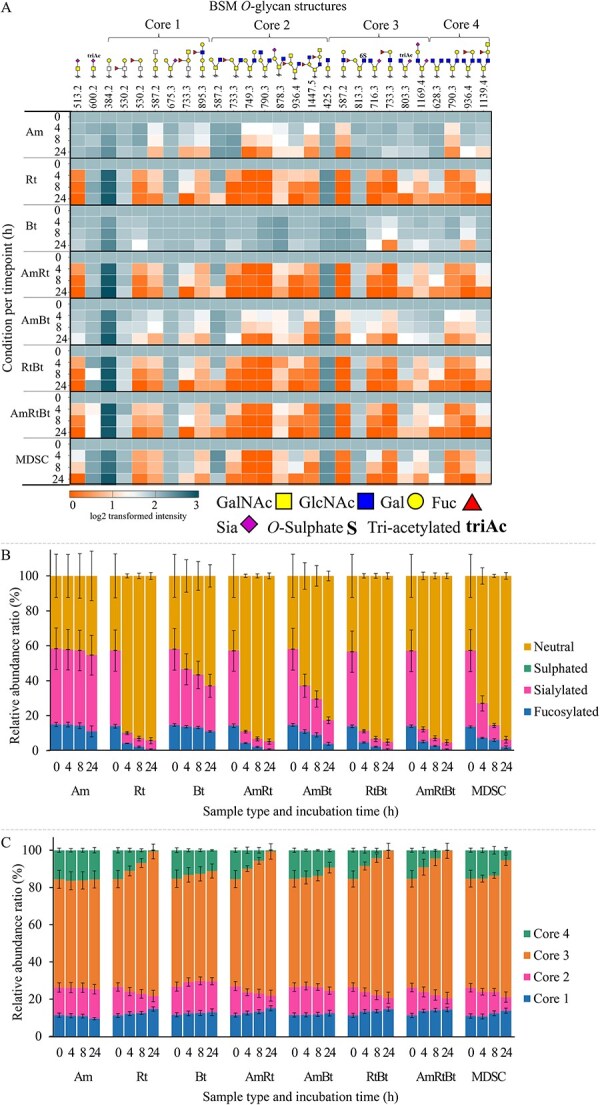
(A) Heatmap showing the degradation of *O-*glycans released from bovine submaxillary glands (BSM) after 0, 4, 8, and 24 h incubation with bacterial lysates of *A. muciniphila* (Am), *R. torques* (Rt), *B. thetaiotaomicron* (Bt), co-cultures thereof (AmRt, AmBt, RtBt, and AmRtBt), and the MDSC after growth on PGM for 12 h (average of three replicates). The value at 0 h is set to 100% (log2 scale value 2) and the values at the following timepoints are shown relative to this value (log2 scale; 0 (orange) degraded and 2 or higher (blue) not degraded). (B) Bar graph of the ratio between present neutral, sulphated, sialylated, and fucosylated *O-*glycans released from BSM after 0, 4, 8, and 24 h incubation with bacterial lysates of Am, rt, Bt, AmRt, AmBt, RtBt, AmRtBt, and the MDSC after growth on PGM for 12 h. (C) Bar graphs of the ratio between present core 1, core 2, core 3, and core 4 *O-*glycans released from BSM after 0, 4, 8, and 24 h incubation with bacterial lysates of Am, rt, Bt, AmRt, AmBt, RtBt, AmRtBt, and the MDSC after growth on PGM for 12 h.

Degradation of the *O*-glycans released from BSM was included as BSM contains a higher abundance of sialylated and sulphated *O*-glycans and includes different core structures including core 3 *O*-glycans compared to PGM. The structures of the different *O*-glycans and the resulting BSM-derived *O*-glycan profiles after enzymatic digestion are shown in Fig. 5 and the related chromatograms are shown in Figs. S31–S39. Similar patterns were observed compared to degradation of PGM *O*-glycans but the structural diversity of *O*-glycans present revealed additional insights. Briefly, *O*-glycans with Fucα1–2Gal and Fucα1–3GlcNAc linkages were degraded by all tested bacterial conditions. Preferential degradation of Fuc attached to non-terminal Gal or GlcNAc by enzymes of monoculture *A. muciniphila* was observed. Enzymes from monoculture *R. torques* and *B. thetaiotaomicron* demonstrated preferential degradation of Fuc attached to terminal Gal. *O*-glycans with Siaα2–3Gal linkages were degraded by all tested bacterial conditions and Siaα2–6Gal linkages were degraded by all conditions except for monoculture *A. muciniphila*. Enzymes from the co-culture lysates degraded all *O-*glycans that were also degraded by enzymes from the monocultural lysates and the degradation patterns reflected degradation by the monocultures as shown in Fig. 5. Specifically for BSM, core 4 *O*-glycan *m/z* 628.3, not present in PGM, was degraded by monoculture *R. torques* and *B. thetaiotaomicron* but not by *A. muciniphila*, and core 3 *O*-glycan *m/z* 425.2 accumulated overtime for all tested bacterial conditions, suggesting that this structure originated from the degradation of longer *O*-glycans (core 3 and core 4 structures).

## Discussion

**Figure 6:**
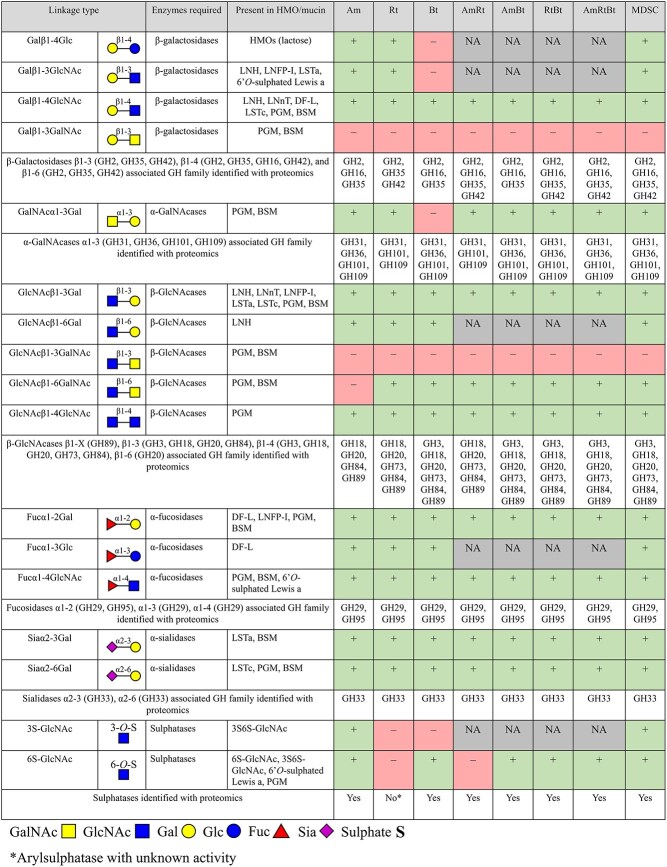
Overview of results obtained from proteomics and enzymatic experiments regarding the cleavage of glycan linkages present in HMOs, sulphated glycans, and released *O-*glycans from PGM and BSM by enzymes of bacterial lysates of *A. muciniphila*, *R. torques*, and *B. thetaiotaomicron*, in monocultures, co-cultures, and the MDSC. Linkage cleavage within 24 h is indicated with “+,” linkage not cleaved within 24 h is indicated with “–,” and not applicable/not tested is indicated with “NA.”

The activity and specificity of enzymes induced upon 12 h incubation of PGM in monocultures *A. muciniphila*, *R. torques*, and *B. thetaiotaomicron*, in co-cultures thereof, and the MDSC was investigated by their ability to cleave glycan linkages present in HMOs, sulphated glycans, and *O-*glycans released from PGM and BSM. Furthermore, proteomics was performed on the bacterial lysates, after 24 h growth on PGM, and this was combined with the information retrieved from enzyme databases (e.g. CAZymes and Uniprot) and literature to further study enzyme-glycan interactions. To summarise the information from proteomics and of the biochemical characterisation of the digests as presented in the results section an overview is shown in Fig. 6.

### Cleavage of glycan linkages by enzymes produced by monocultures *A. muciniphila*, *R. torques*, and *B. thetaiotaomicron*

Enzymes produced by *A. muciniphila* efficiently cleaved terminally linked Gal (Galβ1–4GlcNAc) and degraded poly-LacNAc chains (Galβ1–4GlcNAc and GlcNAcβ1–3Gal) present in PGM and BSM *O*-glycans. This corroborated with favoured cleavage of β1–4 linked Gal and β1–3 linked GlcNAc as evidenced by the HMO experiments and the characterisation by proteomics of β-galactosidases and β-GlcNAcases. Similar observations were discussed by Kostopoulos et al. (2020) during a HMO breakdown study with *A. muciniphila*. Cleavage of GlcNAcβ1–3GalNAc and GlcNAcβ1–6GalNAc was not observed in our study, even though *A. muciniphila* produced multiple GH20 enzymes with wide specificity towards cleaving GlcNAc β-linkages (Schaus et al. 2024; Bakshani et al. 2025) and *N*-GlcNAcases from GH18, GH84, and GH89. Enzymes from *A. muciniphila* favoured cleavage of α1–2 linked Fuc over α1–3 linked Fuc and faster degradation of *O*-glycan structures with Fuc linked to non-terminal Gal or GlcNAc compared to Fuc linked to terminal Gal was observed. Identified Amuc_0010^GH29^ acts specifically on Fuc α1–2 linked to lactose or LacNAc (Gal), identified Amuc_0186^GH95^ and Amuc_1120^GH95^ also act specifically on α1–2 linked Fuc, and only Amuc_0392^GH29^ acts on any Fuc linkage (Bakshani et al. 2025). This could explain the preference regarding α1–2 linked Fuc over α1–3 linked Fuc as more enzymes are produced effective on Fucα1–2X. The sugar to which the Fuc is linked also influences enzyme activity e.g. Amuc_0010^GH29^ acts specifically on Fuc linked to lactose and LacNAc. Enzymes produced by *A. muciniphila* showed more affinity towards cleavage of α2–3 linked Sia compared to α2–6 linked Sia. Using proteomics, sialidases able to cleave both linkages were identified and it has been shown that sialidases produced by *A. muciniphila* can remove both α2–3 and α2–6 linked Sia (Muñoz-Provencio and Yebra 2023; Bakshani et al. 2025). Therefore it is speculated that Siaα2–6 linkages are cleaved slower than Siaα2–3 linkages by *A. muciniphila* or that the specifically tested structures influence the enzyme activity. As only two sialylated glycan standards (LSTa and LSTc) were used in this study no definite conclusion can be drawn. Enzymes from *A. muciniphila* removed sulphate groups from sulphated glycans 3S-GlcNAc and 3S6S-GlcNAc, however, sulphate was not removed from 6’-*O*-sulphated Lewis a nor from sulphated PGM *O*-glycan 6S-GlcNAcβ1–6(Galβ1–3)GalNAc (*m/z* 667.3). Proteomics identified various sulphatases produced by *A. muciniphila* active on 6S-GlcNAc linkages (S1_11 and S1_20 shown in Table 1) which suggests that the specific configuration of the Lewis structure influenced the enzymatic activity. This corroborated with literature where break down of 6’-*O*-sulphated Lewis a by *A. muciniphila* was neither reported (Bakshani et al. 2025).

*R. torques* produced less different GH families compared to *A. muciniphila* and *B. thetaiotaomicron*, but *R. torques* still efficiently degraded the glycan linkages in the tested HMOs and mucin *O*-glycans. This shows that enzymes produced by *R. torques* are sufficient for complete degradation of glycan linkages and suggests that these enzymes have broader enzyme activity and specificity compared to *A. muciniphila* and *B. thetaiotaomicron*. Enzymes expressed by *R. torques* are predominantly secreted enzymes which affects the structural breakdown (Schaus et al. 2024). The efficient degradation of the glycan linkages in the tested structures is in line with described strong α-L-fucosidase, sialidase, and β1–4-galactosidase activity in *R. torques* strains (Schaus et al. 2024). Sulphated glycans were not degraded by *R. torques*, indicating that the identified arylsulphatase with unknown activity is not active on these sulphate linkages.

*B. thetaiotaomicron* can adapt to its environment depending on the available carbohydrates, but dietary glycans are its preferred substrate (Kostopoulos et al. 2021; Pruss et al. 2021; Jegatheesan et al. 2024). This could explain the slow growth and low cell count of *B. thetaiotaomicron* when grown on PGM (Fig. S1) and the limited degradation of *O-*glycans released from PGM and BSM compared to readily degraded HMOs and sulphate structures. A wide range and high abundance of enzymes from GH families involved in mucin glycan cleavage was identified with proteomics, indicating that necessary enzymes are produced by *B. thetaiotaomicron*. Galβ1–4Glc, present in lactose, was not cleaved by *B. thetaiotaomicron* which could indicate that *B. thetaiotaomicron* insufficiently expressed necessary enzymes when grown on PGM. Enzymes produced by *B. thetaiotaomicron* removed 6-linked sulphate from the sulphated glycans 6S-GlcNAc and 3S6S-GlcNAc but cleavage of 3-linked sulphate from 3S6S-GlcNAc and 6-linked sulphate from 6’-*O*-sulphated Lewis a was not observed. The neighbouring monomers could have influence on the enzyme activity. *B. thetaiotaomicron* cleaved 6-linked sulphate from sulphated PGM *O*-glycan 6S-GlcNAcβ1–6(Galβ1–3)GalNAc (*m/z* 667.3) which indicated a unique niche and function for *B. thetaiotaomicron* in co-culture. If no other mucin glycan degraders are present, it is vital for *B. thetaiotaomicron* to be able to remove terminal residues by itself.

Discrepancies between proteomics characterisation of GH enzymes and the specific preferences for cleavage of glycan linkages could be related to the amount of enzyme produced, the enzyme activity, or the enzyme specificity. The configurations of specific structures, *O*-glycans for example, could also hinder enzymatic activity. With proteomics, soluble and membrane enzymes are characterised while with the biochemical experiments the activity of mostly soluble enzymes will be observed. Furthermore, far from all genes are yet characterised and correctly annotated which highlights the added value of biochemical experiments (Drula et al. 2022; Rodríguez del Río et al. 2024).

### Cleavage of glycan linkages by enzymes produced by co-cultures of *A. muciniphila*, *R. torques*, and *B. thetaiotaomicron*, and the MDSC

Co-cultures of the three bacteria mostly demonstrated similar degradation patterns compared to the respective monocultures. This suggests that each bacterium participated in *O*-glycan degradation. Furthermore, this implies that the present monocultures each produce the necessary enzyme repertoire regardless of other bacteria present. This was confirmed by proteomics showing the presence of all enzymes from each monoculture in the corresponding co-cultures. The enzyme abundance in the co-cultures did occasionally differ from the related monocultures which suggested that the enzyme production was influenced by cooperation or competition between the bacteria. As a result of multiple bacteria present, a wider enzyme repertoire was identified in the co-cultures and MDSC compared to the monocultures. The co-cultures containing *R. torques* showed rapid growth on PGM and the enzymes produced efficiently degraded the majority of the *O-*glycans. Co-cultures containing *B. thetaiotaomicron*, showed degradation of sulphated *O-*glycans. *B. thetaiotaomicron* has been mentioned for its ability to release sulphate, which can be used as a substrate by sulphate-reducing bacteria, highlighting an important role for *B. thetaiotaomicron* (Ndeh et al. 2018; Davies et al. 2024). Furthermore, this could make the remaining glycan chains easier accessible by enzymes from *B. thetaiotaomicron* and other bacteria. Enzymes produced by co-culture *A. muciniphila*/*R. torques*/*B. thetaiotaomicron* degraded all present *O-*glycans except Galβ1–3GalNAc (*m/z* 384.2), showing that the three bacteria can produce almost all enzymes necessary for breakdown of glycan linkages present in PGM and BSM *O-*glycans. Enzymes produced by the MDSC demonstrated similar degradation capabilities of *O-*glycans released from PGM and BSM compared to co-culture *A. muciniphila*/*R. torques*/*B. thetaiotaomicron*. This suggests that *A. muciniphila*, *R. torques*, and *B. thetaiotaomicron* are capable of primary mucin glycan degradation emphasising the effectivity of especially mucin glycan-degrading specialists *A. muciniphila* and *R. torques*.

### The relevance of *A. muciniphila*, *R. torques*, and *B. thetaiotaomicron* in the human gut

The results from this study show that *A. muciniphila*, *R. torques*, and *B. thetaiotaomicron* each produce their unique GH enzyme repertoire in monocultures, co-cultures, and in a synthetic community. Furthermore, the unique degradation and utilisation of mucin glycans by each bacterium is highlighted indicating different roles and interactions in the human gut. *A. muciniphila* and *R. torques* produce GHs involved in mucin degradation corresponding to efficient removal of glycans from mucin structures whereas *B. thetaiotaomicron* produces a broader repertoire of GHs resulting in less mucin glycan degradation but more tools for degrading a broader range of dietary fibre glycans. Mucin degradation and utilisation in the human gut is a coordinated effort between different bacterial species, fulfilling all necessary functions (Joja et al. 2025). *A. muciniphila* and *R. torques* can both remove glycans from mucins, but different preferences and specificities were observed in this study. Furthermore, the degradation patterns of the tested HMOs might indicate that *A. muciniphila* is more impacted by structural configuration compared to *R. torques*. This could support the efficient degradation of mucin glycans by *R. torques*. *B. thetaiotaomicron* was able to remove sulphate from the mucin structures and thereby performs an important role in mucin glycan utilisation by making the remaining glycan chain accessible for other species and providing released sulphate as a resource for other bacteria. It should be taken into account that the mucin structures in humans are different from the tested structures in this study. For example, human colon mucins contain a higher level of sialylation and sulphation (Luis and Hansson 2023; Berkhout et al. 2024). This study indicates that the relative abundance of each bacteria is also of importance and that the threshold for beneficial activity per bacteria is different. A low relative abundance of *R. torques* in the co-cultures would be sufficient for effective mucin *O*-glycan degradation. Furthermore, co-cultures as well as the MDSC illustrate that bacteria interact with each other and influence the relative abundance of other bacteria as well as their capabilities in glycan degradation and utilisation. This suggests a complex interplay between the different bacteria and degradation and utilisation of available glycan sources in a complex ecosystem.

### Studying the interaction between mucin glycans and gut microbiota requires multiple techniques

During this study, it was illustrated that *A. muciniphila*, *R. torques*, and *B. thetaiotaomicron* in monocultures, co-cultures, and a synthetic bacterial community produce a different enzyme repertoire associated with specific mucin glycan degradation patterns and preferences. This highlights the strength of using multiple techniques and experiments to explore the presence of bacterial enzymes and their degradation capacities regarding mucin glycan degradation. Specific preferences of linkage cleavage by enzymes produced by bacteria or the effect of substrate configuration on enzyme activity cannot be concluded from proteomics alone. The actual specificity of bacterial enzymes can only be discovered using biochemical experimentation, monitoring the fate of individual glycans present in mucin. This type of information is essential to understand the mechanisms used by bacteria to degrade mucin glycans in all its complexity and diversity. Care should be taken regarding the influence of the chosen bacteria regarding their glycan degradation functions and the included substrates as these can influence the enzyme production. In addition, the use of cell lysates as a source of carbohydrate active enzymes as done in this research, informs us of the mucin-degrading potential of these enzymes but it does not give information on the localisation nor their function in a broader cellular context.

This research has taken a step forward to understanding enzyme-glycan interactions. It was shown that each bacterium produces a unique repertoire of enzymes and that bacteria in co-culture still efficiently produce the necessary enzymes for mucin glycan degradation. By identification of enzymes produced and combining this with enzyme specificity during biochemical experiments we can understand the function and role of bacteria in the gut regarding glycan degradation. This can help to understand the effect of bacterial abundance in the human GIT.

## Materials and methods

### Chemicals

Water (H_2_O), acetonitrile (ACN), methanol (MeOH), acetic acid, and trifluoroacetic acid (TFA) ULC/MS-CC/SFC grade ≥ 99% were all obtained from Biosolve (Dieuze, France). Lacto-*N-*hexaose (LNH), lacto-*N-*neotetraose (LNnT), difucosyllactose (DF-L), lacto-*N-*fucopentaose I (LNFPI), LS-Tetrasaccharide a (LSTa), LS-Tetrasaccharide c (LSTc), *N-*acetyl-d-glucosamine-6-*O-*sulphate sodium salt (6SGlcNAc), and *N-*acetyl-d-glucosamine-3-6-di-*O-*sulphate sodium salt (3S6SGlcNAc) all >95% purity were obtained from Dextra Laboratories Ltd. (Reading, Berkshire, UK). 6'-*O-*Sulphated Lewis a > 95% purity was obtained from Biosynth Ltd. (Compton, Berkshire, UK). 1,4-α-D-maltopentaose 99% (MP5), acetone ≥99%, partially purified powder of mucin type III from porcine stomach, mucin from bovine submaxillary glands type I-S, ammonium bicarbonate (NH_4_HCO_3_) BioUltra ≥98%, sodium borohydride (NaBH_4_) caplets 98%, sodium hydroxide (NaOH) ≥98% pellets, sodium phosphate monobasic monohydrate ≥99%, tris(hydroxymethyl)aminomethane ≥98%, SpeedBeads magnetic carboxylate modified particles 0.70–1.10 μm and 1 μm average size 5% solids in suspension, Roche cOmplete protease inhibitor cocktail, DL-dithiothreitol (DTT) ≥99%, urea ≥99%, and Supelco Supelclean Envi-Carb solid phase extraction (SPE) tubes (250 mg, 3 mL) were all obtained from Sigma Aldrich (Darmstadt, Germany). Sep-Pak Vac 6 cc 500 mg 6 mL C18 SPE cartridges were obtained from Waters (Eschborn, Germany). Sodium sulphate (Na_2_SO_4_) ≥99%, acrylamide ≥98%, BGB 0.2 mL PP short thread vials 32 × 11.6 mm and BGB ND9 short thread screw caps with slitted septa silicone/PTFE were obtained from Thermo Scientific (San Jose, CA, USA). 1.5 mL and 2.0 mL Eppendorf Safe-Lock microcentrifuge tubes were obtained from VWR (Boxmeer, the Netherlands).

### Growth of monocultures, co-cultures, and the mucin-degrading synthetic community and preparation of bacterial lysates to be used in glycan degradation experiments

The mucin-degrading synthetic community (MDSC) consisted of a variety of intestinal bacteria known as mucin glycan degraders (specialists and generalists) and cross-feeders as described previously (Berkhout et al. 2024). The MDSC contained *Akkermansia muciniphila* DSM 22959, *Ruminococcus gnavus* ATCC 29149, *Ruminococcus torques* ATCC 27756, *Bacteroides fragilis* DSM 2151, *Bacteroides thetaiotaomicron* DSM 2079, *Phocaeicola vulgatus* ATCC 8482, *Bacteroides caccae* DSM 19024, *Anaerostipes caccae* DSM 14662, *Agathobacter rectalis* ATCC 33656, *Faecalibacterium duncaniae* DSM 17677, *Roseburia intestinalis* DSM 14610, *Desulfovibrio piger* DSM 749, and *Blautia hydrogenotrophica* DSM 10507. Pre-culturing of monocultures *A. muciniphila*, *R. torques*, and *B. thetaiotaomicron*, co-cultures *A. muciniphila*/*R. torques*, *A. muciniphila*/*B. thetaiotaomicron*, *R. torques*/*B. thetaiotaomicron*, and *A. muciniphila*/*R. torques*/*B. thetaiotaomicron*, and the MDSC on PGM was performed as described previously (Berkhout et al. 2024). Incubation of *A. muciniphila* on GlcNAc and *R. torques* and *B. thetaiotaomicron* on Glc, and preparation of crude PGM (0.5% w/v) was also performed as described previously (Berkhout et al. 2024). Sampling of the incubation mixture was performed after 0, 12, and 24 h incubation. Microbial growth was followed by OD600 measurement (Implen OD600 DiluPhotometer, München, Germany). The bacterial samples were sonicated at 4 °C in a cup sonicator (Q-sonica, Newtown, Connecticut, USA) for 14.5 min at 100%, then 0.5 min rest, 2 cycles. Lysates were stored at −20 °C until further use. Samples were stored for maximally 2 months. As method controls, a similar growth experiment was performed using incubation of PGM medium without bacteria, and incubation of the MDSC on Glc as carbon source without PGM.

Relative abundance of bacteria was measured at 12 and 24 h by 16S rRNA gene amplicon sequencing based on previously described protocols by Shetty et al. (2022) and Berkhout et al. (2024) (number of 16S rRNA gene copies per bacterium is described in Table S4). The total bacterial abundance was determined by qPCR as described previously (Berkhout et al. 2024). Standards were prepared with *B. fragilis* DNA by amplification of the 16S rRNA gene, samples were diluted to 1 ng/μL, and qPCR was performed with primers BACT-F-1369 (5’-CGGTGAATACGTTCYCGG) and PROK-R-1492 (5’-GGWTACCTTGTTACGACTT) (Suzuki et al. 2000). Results were analysed with CFX Manager software v3.1 (Bio-Rad) to calculate the 16S rRNA gene copy number/μL as described previously (Berkhout et al. 2024).

### Proteomics sample preparation

Samples were prepared for proteomics analysis using the protein aggregation capture (PAC) method (Hughes et al. 2014; Batth et al. 2019; Dagley et al. 2019). This method yields additional hydrophobic peptides compared to filter-aided sample preparation (FASP) (Murfuni et al. 2024). Samples prepared for proteomics analysis included monocultures *A. muciniphila*, *R. torques*, and *B. thetaiotaomicron*, co-cultures thereof, and the MDSC after incubation on PGM for 24 h. Samples were prepared from triplicate culture experiments. Firstly, 4 mL acetone was added to 1 mL of bacterial sample and incubated at room temperature (RT) for 30 min. Samples were centrifuged at 4000 x *g* for 30 min, and the supernatant was removed. The pellet was dissolved in 75 μL TRIS buffer pH 8 with protease inhibitor cocktail and the solution was sonicated in a Qsonica cup sonicator for 30 min alternating 15 s pulse and 15 s break at 100% amplitude at 4 °C. Subsequently, 40 μL of this mixture was used for proteomics sample preparation. To each sample, 4 μL of 150 mM DTT was added to a final concentration of 14 mM and incubated at 45 °C for 30 min at 300 rpm. Then, the samples were diluted four times by adding 132 μL of 8 M urea in TRIS buffer (pH 8). To each sample 18 μL of 200 mM acrylamide was added to a final concentration of 18 mM and incubated at RT for 30 min. After incubation, the pH of the samples was adjusted to pH 7 by addition of 2.6 μL of 10% TFA. Then, the SpeedBeads were prepared by mixing 75 μL of both types (0.70–1.10 μm and 1 μm) and washing the beads three times with 1 mL ultra-pure water followed by resuspending the beads in 150 μL ultra-pure water. 5.5 μL of SpeedBeads mixture was added per sample and 505 μL of ACN was added. Samples were incubated at RT under continuous shaking for 30 min. Beads were separated from the liquid using a magnetic rack for 30 s, and the liquid was removed from each sample. Then, samples were washed 3x 1 mL of 70% ethanol (EtOH), beads were separated, and liquid was removed. This was repeated 3x using 1 mL ACN. Protein digestion was performed by addition of 100 μL 5 ng/μL trypsin in 50 mM NH_4_HCO_3_ to each sample. Samples were incubated at RT at 300 rpm overnight. After 2 h an additional 50 μL 5 ng/μL trypsin in 50 mM NH_4_HCO_3_ was added to each sample. The following day, digested peptide samples were acidified by addition of 3 μL of 10% TFA to pH 3, pulse centrifuged, and the beads were separated on a magnetic rack. Sample liquid was transferred to clean tubes. Beads were washed with 100 μL of 1 mL/L formic acid (FA) in water, beads were separated, and the liquid was added to the clean tubes. Then, beads were washed with 50 μL of 1 mL/L FA, beads were separated, and the liquid was added to the clean tubes. Samples were centrifuged for 10 min at 10,000 rpm, transferred to clean tubes, and the liquid was evaporated using a centrifugal evaporator. The cleaned-up peptide samples were reconstituted in 50 μL using 1 mL/L FA and 1 μL of sample was injected into the mass spectrometer per measurement.

### nLC–MS/MS measurement of obtained peptides

Proteomics of the protease treated samples was conducted to study the enzymes present in the bacterial lysates with a nLC–MS/MS system. One μL of sample was injected onto a nLC system (Vanquish Neo, ThermoFisher Scientific) coupled to an Orbitrap Exploris 480 mass spectrometer (ThermoFisher Scientific) as described by Zheng et al. (2024). The nLC–MS/MS data was analysed using MaxQuant v2.0.3.0 (Cox lab) (Cox and Mann 2008) as described by Zheng et al. (2024) except for the methylglyoxal modifications. The reference database consisted of proteomes of MDSC members as retrieved from the Uniprot database (Bateman et al. 2025). The used proteome of each MDSC species is specified in Table S5.

### Release of O-glycans from PGM and BSM using reductive β-elimination

*O*-glycans were released from PGM and BSM as described previously (de Ram et al. 2024). In short, 750 μL of 1 M NaBH_4_ in 0.05 M NaOH was added to 3 mg of PGM or BSM. Samples were homogenised and maltopentaose DP5 was added as internal standard (IS, 1 μL of 1 mg/mL solution). Glycans were released from the protein backbone using reductive β-elimination overnight (20 h) at 40 °C with continuous shaking at 300 rpm in an Eppendorf Thermomixer comfort (Nijmegen, the Netherlands). Then, samples were acidified to pH 6 using glacial acetic acid, cleaned-up using C18 and PGC SPE, and vacuum dried (Savant centrifugal evaporator, Thermo Scientific).

### Enzymatic degradation of human milk oligosaccharides by bacterial enzymes

First, 75 μL ULC–MS grade water and 75 μL 180 mM sodium phosphate buffer (pH 6.0) were combined. Then, 15 μL of 1 mg/mL human milk oligosaccharide (HMO) was added. Subsequently, 15 μL of bacterial lysate after incubation on PGM for 24 h, was added to each HMO sample (ratio based on preliminary experiments of which results are not shown). Samples were mixed by pipetting up and down and incubated at 37 °C with continuous shaking at 300 rpm for 24 h. After 0, 6, and 24 h an aliquot of 50 μL was taken and heated at 100 °C for 5 min with continuous shaking at 300 rpm to inactivate enzymes present. Samples were stored at −20 °C until analysis.

### Enzymatic degradation of sulphated glycans by bacterial enzymes

First, 30 μL ULC–MS grade water and 30 μL 180 mM sodium phosphate buffer (pH 6.0) were combined. Then, 30 μL of 1 mg/mL sulphated glycan was added. Subsequently, 30 μL of bacterial lysate after incubation on PGM for 24 h, was added to each sulphated glycan sample (ratio based on previous experiments, results not shown). The samples were mixed by pipetting up and down and incubated at 37 °C with continuous shaking at 300 rpm for 24 h. After 0, 4, 8, and 24 h an aliquot of 28 μL was taken and heated at 100 °C for 5 min with continuous shaking at 300 rpm to inactivate enzymes present. Samples were stored at −20 °C until analysis. The experiment was performed in duplicate.

### Enzymatic degradation of *O*-glycans released from PGM and BSM by bacterial enzymes

*O-*glycans released from 3 mg starting amount of PGM or BSM were dissolved in 75 μL ULC–MS grade water. Then, 30 μL of released PGM or BSM *O-*glycan solution was added to 30 μL ULC–MS grade water and 30 μL 180 mM sodium phosphate buffer (pH 6.0). Subsequently, 30 μL of bacterial lysate after incubation on PGM for 24 h, was added. Samples were gently mixed by pipetting up and down and, subsequently, incubated at 37 °C with continuous shaking at 300 rpm for 24 h. After 0, 4, 8, and 24 h an aliquot of 28 μL was taken and heated at 100 °C for 5 minutes at 300 rpm to inactive enzymes present. Samples were stored at −20 °C until analysis. The experiment was performed in triplicate.

### High performance anion exchange chromatography analysis of bacterial enzyme digested HMOs

High performance anion exchange chromatography coupled to pulsed amperometric detection (HPAEC-PAD) was used to analyse the resulting glycan patterns after incubation of HMOs with bacterial enzymes. Samples of HMOs incubated with bacterial lysate after incubation on PGM for 24 h were diluted three times with ultra-pure water and 10 μL of each sample was injected onto the system. The analysis was performed using an ICS6000 HPAEC system with Pulsed Amperometry Detection (PAD; ICS5000 ED, Thermo Scientific, Sunnyvale, CA, USA) equipped with a CarboPac PA-1 column (250 mm × 2 mm i.d.) and a CarboPac PA guard column (25 mm × 2 mm i.d.). Mobile phases used were 0.1 M NaOH (A) and 1 M NaOAc in 0.1 M NaOH (B), the column temperature was set to 20 °C, and the flow rate was set to 0.3 mL/min. The gradient profile for elution was as follows: 0–7% B from 0–5 min, 7–30% B from 5–20 min, 30% B from 20–30 min, column washing with 100% B from 30.1–35 min, and, finally, column re-equilibration with 0% B from 35.1–45 min. Calibration curves of succinate, formate, acetate, 1,2-propanediol, propionate, butyrate, EtOH, and isovalerate (0.001–4 mg/mL, R2 ≥ 0.98) were used for quantification. Data analysis was performed using Chromeleon v7.3.1 software.

### PGC-LC–MS/MS analysis of bacterial enzyme digested sulphated glycans

Sulphated glycans were analysed by porous graphitised carbon liquid chromatography coupled to mass spectrometry (PGC-LC–MS/MS). The samples were diluted two times in ULC–MS grade water and 2 μL of sample was injected onto the system. A Vanquish ultra-high pressure liquid chromatography system (UPLC; Thermo Scientific) equipped with a PGC Hypercarb guard column (10 × 2.1 mm, particle size 3 μm, Thermo Scientific) and a PGC Hypercarb analytical column (150 × 2.1 mm, particle size 3 μm, Thermo Scientific) were used. The mobile phases used were 10 mM NH_4_HCO_3_ in 95:5 ULC–MS grade water:ACN (A) and 100% ULC–MS grade ACN (B), the column temperature was set to 80 °C, and the flow rate was set to 200 μL/min. The gradient profile for elution was as follows: 0% B for 3 min, 0–15% B from 3–7 min, 15–65% B from 7–9 min, and, finally, column re-equilibration with 0% B from 9–12 min. The UPLC set-up was coupled to a UV detector (Vanquish Variable Wavelength Detector, Thermo Scientific) set to 214 nm and then coupled via a HESI source to a Velos Pro ion trap MS (Thermo Scientific). Data acquisition was performed in negative ion mode (full MS *m/z* 50–1500 and MS/MS data dependent). Xcalibur Qual browser v4.5 and Xcalibur Quan Browser v4.5 were used for data analysis (Thermo Scientific).

### PGC-LC–MS/MS analysis of degradation by bacterial enzymes of *O*-glycans released from PGM and BSM

*O-*glycans were analysed by PGC-LC–MS/MS as described previously (de Ram et al. 2024). In short, samples were diluted two times in ULC-MS grade water, and 2 μL of sample was injected onto the system. A Vanquish ultra-high pressure liquid chromatography system (UPLC; Thermo Scientific) equipped with a PGC Hypercarb guard column (10 × 2.1 mm, particle size 3 μM, Thermo Scientific) and a PGC Hypercarb analytical column (150 × 2.1 mm, particle size 3 μM, Thermo Scientific) were used. The mobile phases used were 10 mM NH_4_HCO_3_ in ULC-MS grade water (A) and 10 mM NH_4_HCO_3_ in 60:40 ULC-MS grade water:ACN (B). Glycans were eluted with a flow rate of 200 μL/min using a gradient from 2% B to 60% B in 40 min. The UPLC set-up was coupled via a HESI source to a Velos Pro ion trap MS (Thermo Scientific). Data acquisition was performed in negative ion mode (full MS and MS/MS data dependent). Xcalibur Qual browser v4.5 and Xcalibur Quan Browser v4.5 were used for data analysis (Thermo Scientific).

### Glycan representation, MS data analysis, and construction of the heatmaps

GlycoWorkBench v1.1 (developed by the EUROCarbDB initiative) was used for glycan representation, annotation, and visualisation (Damerell et al. 2012). The glycan structures were represented according to the Symbol Nomenclature for Glycans (Neelamegham et al. 2019). Representative glycan structures were exported from GlycoWorkBench and used in the figures for visualisation and clarity of the structures identified in the MS data. *O-*glycan structures were assigned based on careful review of the full MS and fragmentation (MS/MS) data as described previously (de Ram et al. 2024), review of literature on PGM and BSM glycans (Hayes et al. 2011; Jin et al. 2017, 2019; Vos et al. 2023; Bechtella et al. 2024), and using the MS/MS search tool from UniCarb-DB (Campbell et al. 2014). Glycan MS data analysis was performed in a step-wise manner. Firstly, a qualitative analysis was performed to compare the *O-*glycans identified by MS among the different samples. Secondly, a semi-quantitative approach was used to compare the abundance (intensities) and relative amounts of identified *O-*glycans present in each sample. Glycan relative amounts were calculated based on the total peak area of identified *O-*glycans and compared between the samples. The *O-*glycan intensities were based on peak areas obtained by manual and automatic peak integration using Xcalibur Qual Browser and Xcalibur Quan Browser software v4.5 (Thermo Scientific). Lastly, the peak area of added IS MP5 was reviewed for each sample and consequently compared with the peak area of the other identified *O-*glycan peaks (reproducibility) (de Ram et al. 2024). Regarding the sulphated glycans, structures were also assigned based on careful review of the full MS and fragmentation (MS/MS) data. Intensity of selected masses based on removal of monomers from the sulphated structures were used for interpretation of the data. Heatmaps of the degradation of PGM and BSM *O-*glycans were made with GraphPad Prism v9.3.1 (GraphPad Software Inc., San Diego, CA, USA). For each sample, the intensity (based on peak area) at 0 h was set to 100% and the intensities at timepoints 4, 8, and 24 h were set relative to this 100%. The values were log transformed and *O-*glycan degradation was visualised by comparing the increase/decrease at the different timepoints relative to 0 h. The constructed heatmap of the proteomics data regarding the intensity of the identified GHs and sulphatases was also made with GraphPad Prism v9.3.1. Per sample, the identified proteins were assigned to CAZymes including GHs, sulphatases, carbohydrate esterases (CEs), polysaccharide lyases (PLs), carbohydrate-binding modules (CBMs), and glycosyltransferases (GTs), using the dbCAN HMMdb (Yin et al. 2012) v12 on the concatenated proteomes of Table S4, as was done previously (Ioannou et al. 2021). KO identifiers were assigned to the same proteomes using the online KofamKOALA tool v2024-02-01 with KEGG 109.0 (Aramaki et al. 2020). The functional annotations were linked to the expressed proteins using R software (R Core Team 2021). Then, the LFQ intensities (obtained using MaxQuant v2.0.3.0 (Cox and Mann 2008)) were log2 transformed. These values were used to construct the heatmap showing the intensities and identified GHs and sulphatases between the samples.

## Supplementary Material

Supporting_Information_DeRamEtAl_enzyme-glycan_interactions_2_cwag004

Supporting_Information_Table_S3_Proteomics_cwag004

## Data Availability

Raw data concerning HPAEC, PGC-LC-MS/MS, and proteomics analysis, processed data for creating the heatmaps, and Table S3 are stored via YODA and available online http://dx.doi.org/10.17887/WUR01-CX5O2Y.
