# Supplementary material for: Efficient mucin O-glycan degradation by specific mucin degrading intestinal bacteria: towards understanding enzyme-glycan interactions
Source: Glycobiology. 2026 Jan 12;36(3):cwag004. doi: 10.1093/glycob/cwag004 (PMC12834350; doi:10.1093/glycob/cwag004)
Supplement: Supporting_Information_DeRamEtAl_enzyme-glycan_interactions_2_cwag004 [file supporting_information_derametal_enzyme-glycan_interactions_2_cwag004.docx]

**Supporting information**

**Efficient mucin *O-*glycan degradation by specific mucin degrading intestinal bacteria: towards understanding enzyme-glycan interactions**

Carol de Ram^a^, Maryse D. Berkhout^b^, Marta Koziol^a^, Laura Blasco Matias^a^, Cynthia Klostermann^a^, Carolina O. Pandeirada^a^, Sjef Boeren^c^, Athanasia Ioannou^b^, Jean-Paul Vincken^a^, Clara Belzer^b‡^, Henk Schols^a*‡^

*a. Laboratory of Food Chemistry, Wageningen University & Research, Bornse Weilanden 9, 6708 WG Wageningen, the Netherlands*

*b. Laboratory of Microbiology, Wageningen University & Research, Stippeneng 4, 6708 WE Wageningen, the Netherlands*

*c. Laboratory of Biochemistry, Wageningen University & Research, Stippeneng 4, 6708 WE Wageningen, the Netherlands*

*Corresponding author

*E-mail address:* [henk.schols@wur.nl](mailto:henk.schols@wur.nl) (H.A. Schols)

^‡^ These authors contributed equally to this work

**Table of contents**

[Figure S1. Bacterial growth as measured by OD600 of *A. muciniphila* (Am), *R. torques* (Rt), *B. thetaiotaomicron* (Bt), co-cultures thereof (AmRt, AmBt, RtBt, and AmRtBt), and the mucin-degrading synthetic community (MDSC) during incubation on porcine gastric mucin (PGM) for 24 h. Bacterial growth was measured after 0, 6, 9, 12, and 24 h using OD600 measurement. OD600 values are the mean of three replicates with error bars indicating the standard deviation. 5](#_Toc215823469)

[Figure S2. Total bacterial abundance of *A. muciniphila* (Am), *R. torques* (Rt), *B. thetaiotaomicron* (Bt), co-cultures thereof (AmRt, AmBt, RtBt, and AmRtBt), and the mucin-degrading synthetic community (MDSC) during incubation on porcine gastric mucin (PGM) for 12 and 24 h as determined by qPCR (mean of three replicates). The x-axis shows the samples at the different cultures and the y-axis shows the average 16S rRNA copies per µL culture (log2 scale). 6](#_Toc215823470)

[Figure S3. Overview of bacterial composition of *A. muciniphila* (Am), *R. torques* (Rt), *B. thetaiotaomicron* (Bt), co-cultures thereof (AmRt, AmBt, RtBt, and AmRtBt), and the mucin-degrading synthetic community (MDSC) during incubation on porcine gastric mucin (PGM) for 12 and 24 h (relative abundance is the mean of three replicates). The composition is based on measured relative abundance by 16S rRNA gene amplicon sequencing and corrected for number of 16S rRNA gene copies (SI Table S3). 7](#_Toc215823471)

[Figure S4. Structures of the human milk oligosaccharides (HMOs) and the sulphated glycans used in this study to investigate specific glycan linkage cleavage and the identified degradation products. (A) HMO core structure (lactose). (B) structures of neutral core HMOs. (C) structures of fucosylated (neutral) HMOs. (D) structures of sialylated (acidic) HMOs. (E) all identified degradation products from tested HMOs by *A. muciniphila*, R. torques, *B. thetaiotaomicron*, and the MDSC. (F) structure of 6S-GlcNAc, GlcNAc with 6-linked sulphate. (G) structure of 3S6S-GlcNAc, GlcNAc with 3-linked and 6-linked sulphate. (H) structure of 6’-*O*-sulphated Lewis a, Lewis a structure with 6-linked sulphate. (I) identified degradation products from sulphated glycans. 8](#_Toc215823472)

[Figure S5. HPAEC-PAD profiles showing degradation of the neutral core HMOs (A) LNH and (B) LNnT, fucosylated (neutral) HMOs (C) DF-L and (D) LNFP-I, and sialylated (acidic) HMOs (E) LSTa and (F) LSTc by enzymes produced by *A. muciniphila* (Am), *R. torques* (Rt), *B. thetaiotaomicron* (Bt), and the mucin-degrading synthetic community (MDSC) during 24 h incubation at 37 °C. Sampling was performed after 0, 6, and 24 h. Peaks not indicated by colour could not be identified based on the available standards. 9](#_Toc215823473)

[Figure S6. PGC-LC-MS elution patterns of selected *m/z* 300 (base peak) of 6S-GlcNAc before (0 h) and after (24 h) incubation with *A. muciniphila* lysate. Top and bottom chromatograms are replicate incubations. 10](#_Toc215823474)

[Figure S7. PGC-LC-MS elution patterns of selected *m/z* 220 (base peak – mass of sulphate) of 6S-GlcNAc before (0 h) and after (24 h) incubation with *A. muciniphila* lysate. Top and bottom chromatograms are replicate incubations. 11](#_Toc215823475)

[Figure S8. PGC-LC-MS elution patterns of selected *m/z* 380 (base peak), *m/z* 300 (base peak - mass of sulphate), and *m/z* 200 (base peak - 2x mass of sulphate) of 3S6S-GlcNAc before (0 h) and after (24 h) incubation with *A. muciniphila* lysate. 12](#_Toc215823476)

[Figure S9. PGC-LC-MS elution patterns of selected *m/z* 608 (base peak), *m/z* 582 (base peak - mass of sulphate), *m/z* 462 (base peak - mass of Fuc), and *m/z* 382 (base peak - mass of sulphate and fucose) of 6’-*O*-sulphated Lewis a before (0 h) and after (24 h) incubation with *A. muciniphila* lysate. 13](#_Toc215823477)

[Figure S10. PGC-LC-MS elution patterns of selected *m/z* 300 (base peak) of 6S-GlcNAc before (0 h) and after (24 h) incubation with *R. torques* lysate. Top and bottom chromatograms are replicate incubations. 14](#_Toc215823478)

[Figure S11. PGC-LC-MS elution patterns of selected *m/z* 220 (base peak – mass of sulphate) of 6S-GlcNAc before (0 h) and after (24 h) incubation with *R. torques* lysate. Top and bottom chromatograms are replicate incubations. 15](#_Toc215823479)

[Figure S12. PGC-LC-MS elution patterns of selected *m/z* 380 (base peak), *m/z* 300 (base peak - mass of sulphate), and *m/z* 200 (base peak - 2x mass of sulphate) of 3S6S-GlcNAc before (0 h) and after (24 h) incubation with *R. torques* lysate. 16](#_Toc215823480)

[Figure S13. PGC-LC-MS elution patterns of selected *m/z* 608 (base peak), *m/z* 582 (base peak - mass of sulphate), *m/z* 462 (base peak - mass of Fuc), and *m/z* 382 (base peak - mass of sulphate and fucose) of 6’-*O*-sulphated Lewis a before (0 h) and after (24 h) incubation with *R. torques* lysate. 17](#_Toc215823481)

[Figure S14. PGC-LC-MS elution patterns of selected *m/z* 300 (base peak) of 6S-GlcNAc before (0 h) and after (24 h) incubation with *B. thetaiotaomicron* lysate. Top and bottom chromatograms are replicate incubations. 18](#_Toc215823482)

[Figure S15. PGC-LC-MS elution patterns of selected *m/z* 220 (base peak – mass of sulphate) of 6S-GlcNAc before (0 h) and after (24 h) incubation with *B. thetaiotaomicron* lysate. Top and bottom chromatograms are replicate incubations. 19](#_Toc215823483)

[Figure S16. PGC-LC-MS elution patterns of selected *m/z* 380 (base peak), *m/z* 300 (base peak - mass of sulphate), and *m/z* 200 (base peak - 2x mass of sulphate) of 3S6S-GlcNAc before (0 h) and after (24 h) incubation with *B. thetaiotaomicron* lysate. 20](#_Toc215823484)

[Figure S17. PGC-LC-MS elution patterns of selected *m/z* 608 (base peak), *m/z* 582 (base peak - mass of sulphate), *m/z* 462 (base peak - mass of Fuc), and *m/z* 382 (base peak - mass of sulphate and fucose) mass spectrum of 6’-*O*-sulphated Lewis a before (0 h) and after (24 h) incubation with *B. thetaiotaomicron* lysate. 21](#_Toc215823485)

[Figure S18. PGC-LC-MS elution patterns of selected m/z 300 (base peak) of 6S-GlcNAc before (0 h) and after (24 h) incubation with the mucin-degrading synthetic community (MDSC) lysate. Top and bottom chromatograms are replicate incubations. 22](#_Toc215823486)

[Figure S19. PGC-LC-MS elution patterns of selected *m/z* 220 (base peak – mass of sulphate) of 6S-GlcNAc before (0 h) and after (24 h) incubation with the mucin-degrading synthetic community (MDSC) lysate. Top and bottom chromatograms are replicate incubations. 23](#_Toc215823487)

[Figure S20. PGC-LC-MS elution patterns of selected *m/z* 380 (base peak), *m/z* 300 (base peak - mass of sulphate), and *m/z* 200 (base peak - 2x mass of sulphate) of 3S6S-GlcNAc before (0 h) and after (24 h) incubation with the mucin-degrading synthetic community (MDSC) lysate. 24](#_Toc215823488)

[Figure S21. PGC-LC-MS elution patterns of selected *m/z* 608 (base peak), *m/z* 582 (base peak - mass of sulphate), *m/z* 462 (base peak - mass of Fuc), and *m/z* 382 (base peak - mass of sulphate and fucose) of 6’-*O*-sulphated Lewis a before (0 h) and after (24 h) incubation with the mucin-degrading synthetic community (MDSC) lysate. 25](#_Toc215823489)

[Figure S22. PGC-LC-MS elution patterns showing the degradation of *O*-glycans released from PGM after incubation with *A. muciniphila* lysate during 24 h. Identified peaks (0 h) are indicated with a number and structure which correspond to the fragmentation MS/MS data as shown in Figure S23. 26](#_Toc215823490)

[Figure S23. PGC-LC-MS/MS fragmentation spectra demonstrating the identification of the present *O-*glycans released from PGM. 36](#_Toc215823491)

[Figure S24. PGC-LC-MS elution patterns showing the degradation of *O*-glycans released from PGM during 24 h incubation with *R. torques* lysate. 37](#_Toc215823492)

[Figure S25. PGC-LC-MS elution patterns showing the degradation of *O*-glycans released from PGM during 24 h incubation with *B. thetaiotaomicron* lysate. 37](#_Toc215823493)

[Figure S26. PGC-LC-MS elution patterns showing the degradation of *O*-glycans released from PGM during 24 h incubation with *A. muciniphila*/*R. torques* lysate. 38](#_Toc215823494)

[Figure S27. PGC-LC-MS elution patterns showing the degradation of *O*-glycans released from PGM during 24 h incubation with *A. muciniphila*/*B. thetaiotaomicron* lysate. 38](#_Toc215823495)

[Figure S28. PGC-LC-MS elution patterns showing the degradation of *O*-glycans released from PGM during 24 h incubation with *R. torques*/*B. thetaiotaomicron* lysate. 39](#_Toc215823496)

[Figure S29. PGC-LC-MS elution patterns showing the degradation of *O*-glycans released from PGM during 24 h incubation with *A. muciniphila*/*R. torques*/*B. thetaiotaomicron* lysate. 39](#_Toc215823497)

[Figure S30. PGC-LC-MS elution patterns showing the degradation of *O*-glycans released from PGM during 24 h incubation with the mucin-degrading synthetic community (MDSC) lysate. 40](#_Toc215823498)

[Figure S31. PGC-LC-MS elution patterns showing the degradation of *O*-glycans released from BSM during 24 h incubation with *A. muciniphila* lysate. Identified peaks (0 h) are indicated with a number and structure which correspond to the fragmentation MS/MS data as shown in Figure S32. 40](#_Toc215823499)

[Figure S32. PGC-LC-MS/MS fragmentation spectra demonstrating the identification of the present *O-*glycans released from BSM . 49](#_Toc215823500)

[Figure S33. PGC-LC-MS elution patterns showing the degradation of *O*-glycans released from BSM during 24 h incubation with *R. torques* lysate. 50](#_Toc215823501)

[Figure S34. PGC-LC-MS elution patterns showing the degradation of *O*-glycans released from BSM during 24 h incubation with *B. thetaiotaomicron* lysate. 50](#_Toc215823502)

[Figure S35. PGC-LC-MS elution patterns showing the degradation of *O*-glycans released from BSM during 24 h incubation with *A. muciniphila*/*R. torques* lysate. 51](#_Toc215823503)

[Figure S36. PGC-LC-MS elution patterns showing the degradation of *O*-glycans released from BSM after incubation with *A. muciniphila*/*B. thetaiotaomicron* lysate during 24 h. 51](#_Toc215823504)

[Figure S37. PGC-LC-MS elution patterns showing the degradation of *O*-glycans released from BSM during 24 h incubation with *R. torques*/*B. thetaiotaomicron* lysate. 52](#_Toc215823505)

[Figure S38. PGC-LC-MS elution patterns showing the degradation of *O*-glycans released from BSM during 24 h incubation with *A. muciniphila*/*R. torques*/*B. thetaiotaomicron* lysate. 52](#_Toc215823506)

[Figure S39. PGC-LC-MS elution patterns showing the degradation of *O*-glycans released from BSM during 24 h incubation with the mucin-degrading synthetic community (MDSC) lysate. 53](#_Toc215823507)

[Table S1. Literature overview of recognised glycoside hydrolyse (GH) families present in *A. muciniphila*, *R. torques*, and *B. thetaiotaomicron* with highlighted mucin glycan degrading CAZymes and their associated activities (Drula et al., 2022; Labourel et al., 2023; Raba & Luis, 2023). *bold GH families represent GH families involved in mucin degradation (mucin-degrading GH families). 55](#_Toc215823953)

[Table S2. Overview of carbohydrate esterases (CE), polysaccharide lyases (PL), auxiliarly activity enzymes (AA), carbohydrate-binding modules (CBM), and glycosyltransferases (GT) identified using proteomics of bacterial supernatant of *A. muciniphila* (Am), *R. torques* (Rt), and *B. thetaiotaomicron* (Bt) grown on porcine gastric mucin (PGM) for 24 h. Per CE, PL, AA, CBM, and GT family the identified genes are described. 56](#_Toc215823954)

[Table S3. Raw proteomics label free quantification (LFQ) results (separate Excel file titled ‘’Supporting_Information_Table_S3_Proteomics.xlsx’’). 58](#_Toc215823955)

[Table S4. Bactrial strains used and the associated number of 16S rRNA gene copies (Berkhout et al., 2024). 58](#_Toc215823956)

[Table S5. Proteomes from Uniprot used as reference database for proteomics analysis (Bateman et al., 2025). 59](#_Toc215823957)


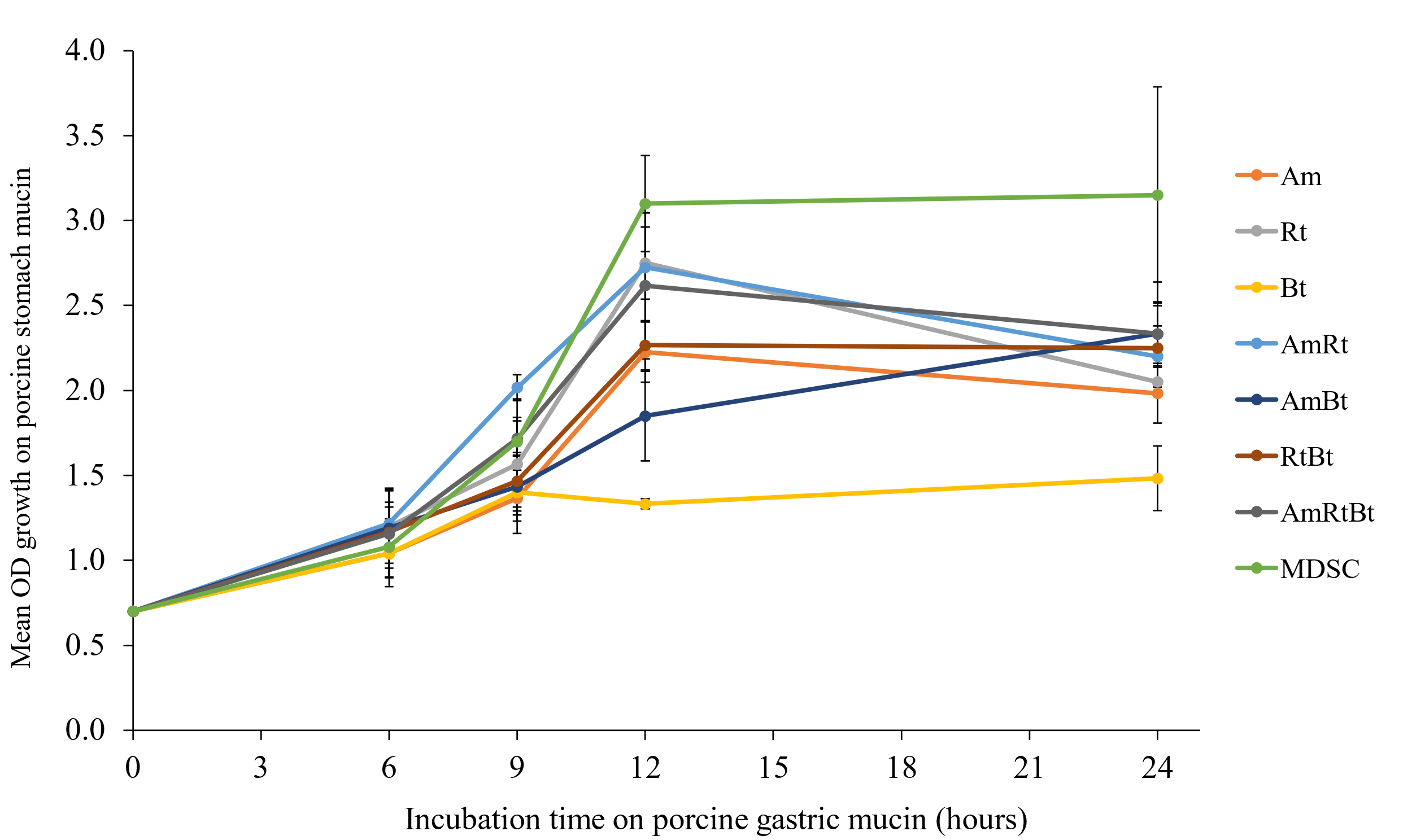


Figure S1. Bacterial growth as measured by OD600 of *A. muciniphila* (Am), *R. torques* (Rt), *B. thetaiotaomicron* (Bt), co-cultures thereof (AmRt, AmBt, RtBt, and AmRtBt), and the mucin-degrading synthetic community (MDSC) during incubation on porcine gastric mucin (PGM) for 24 h. Bacterial growth was measured after 0, 6, 9, 12, and 24 h using OD600 measurement. OD600 values are the mean of three replicates with error bars indicating the standard deviation.


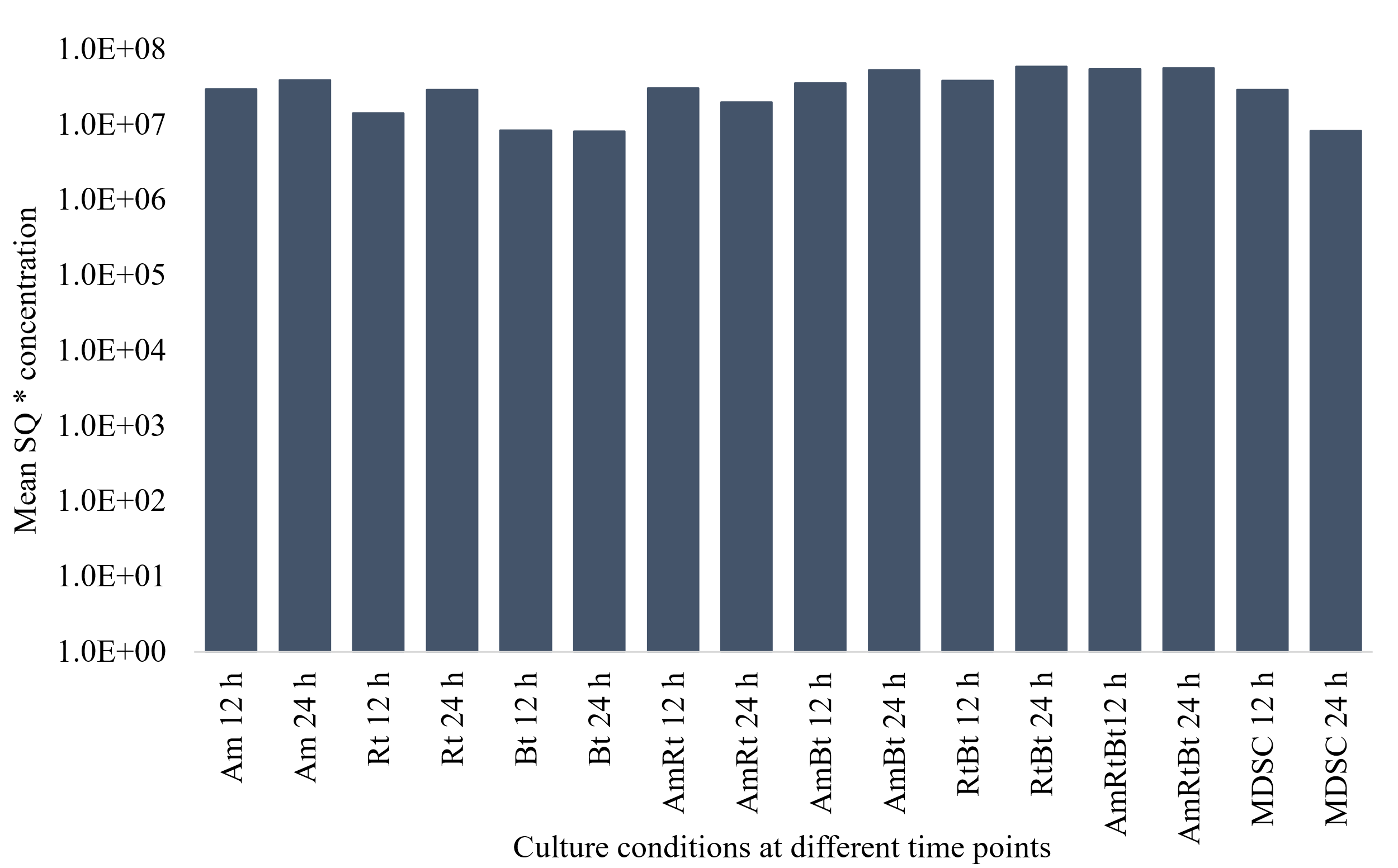


Figure S2. Total bacterial abundance of *A. muciniphila* (Am), *R. torques* (Rt), *B. thetaiotaomicron* (Bt), co-cultures thereof (AmRt, AmBt, RtBt, and AmRtBt), and the mucin-degrading synthetic community (MDSC) during incubation on porcine gastric mucin (PGM) for 12 and 24 h as determined by qPCR (mean of three replicates). The x-axis shows the samples at the different cultures and the y-axis shows the average 16S rRNA copies per µL culture (log2 scale).


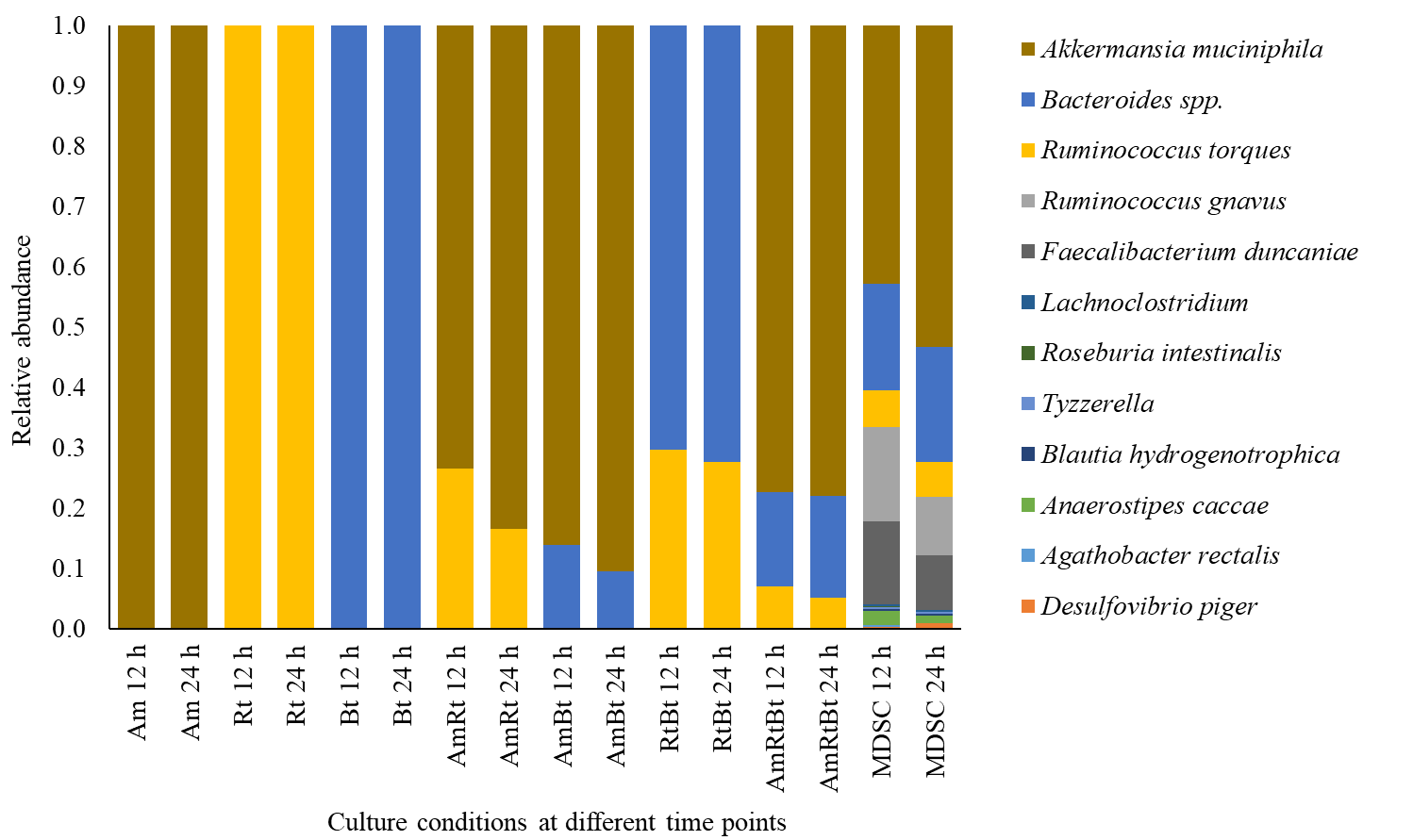


Figure S3. Overview of bacterial composition of *A. muciniphila* (Am), *R. torques* (Rt), *B. thetaiotaomicron* (Bt), co-cultures thereof (AmRt, AmBt, RtBt, and AmRtBt), and the mucin-degrading synthetic community (MDSC) during incubation on porcine gastric mucin (PGM) for 12 and 24 h (relative abundance is the mean of three replicates). The composition is based on measured relative abundance by 16S rRNA gene amplicon sequencing and corrected for number of 16S rRNA gene copies (SI Table S3).


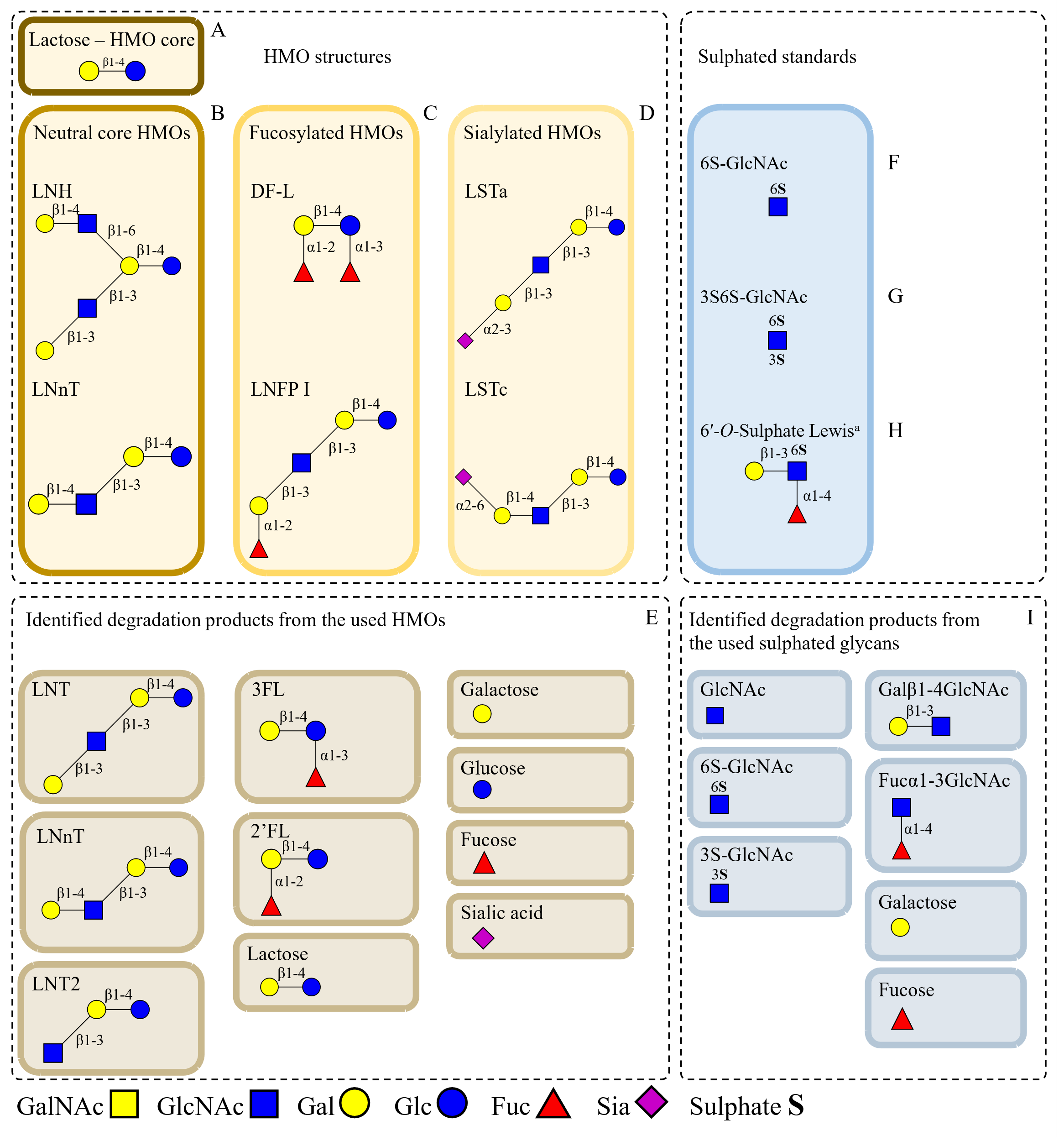


Figure S4. Structures of the human milk oligosaccharides (HMOs) and the sulphated glycans used in this study to investigate specific glycan linkage cleavage and the identified degradation products. (A) HMO core structure (lactose). (B) structures of neutral core HMOs. (C) structures of fucosylated (neutral) HMOs. (D) structures of sialylated (acidic) HMOs. (E) all identified degradation products from tested HMOs by *A. muciniphila*, R. torques, *B. thetaiotaomicron*, and the MDSC. (F) structure of 6S-GlcNAc, GlcNAc with 6-linked sulphate. (G) structure of 3S6S-GlcNAc, GlcNAc with 3-linked and 6-linked sulphate. (H) structure of 6’-*O*-sulphated Lewis a, Lewis a structure with 6-linked sulphate. (I) identified degradation products from sulphated glycans.


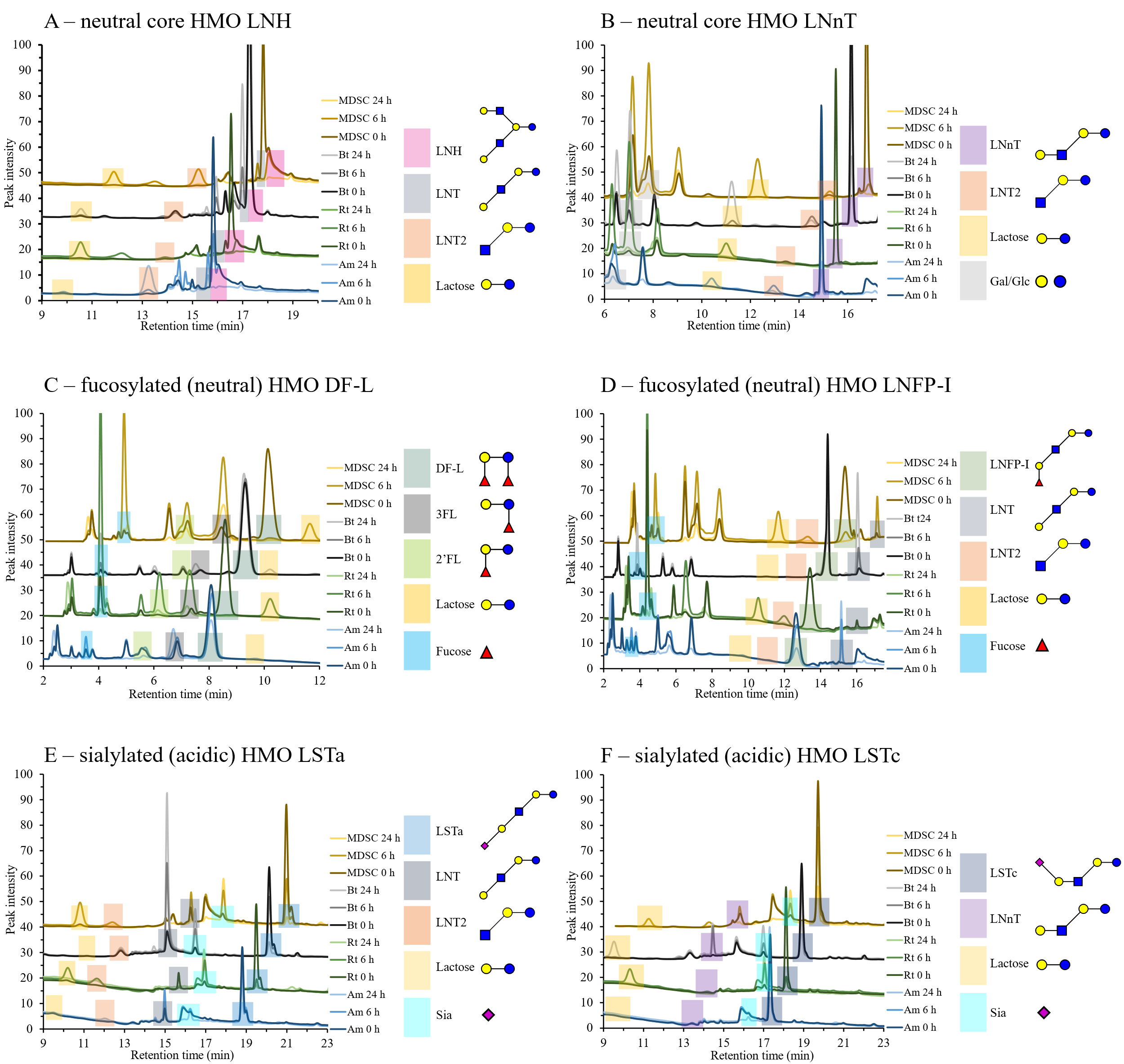


Figure S5. HPAEC-PAD profiles showing degradation of the neutral core HMOs (A) LNH and (B) LNnT, fucosylated (neutral) HMOs (C) DF-L and (D) LNFP-I, and sialylated (acidic) HMOs (E) LSTa and (F) LSTc by enzymes produced by *A. muciniphila* (Am), *R. torques* (Rt), *B. thetaiotaomicron* (Bt), and the mucin-degrading synthetic community (MDSC) during 24 h incubation at 37 °C. Sampling was performed after 0, 6, and 24 h. Peaks not indicated by colour could not be identified based on the available standards.


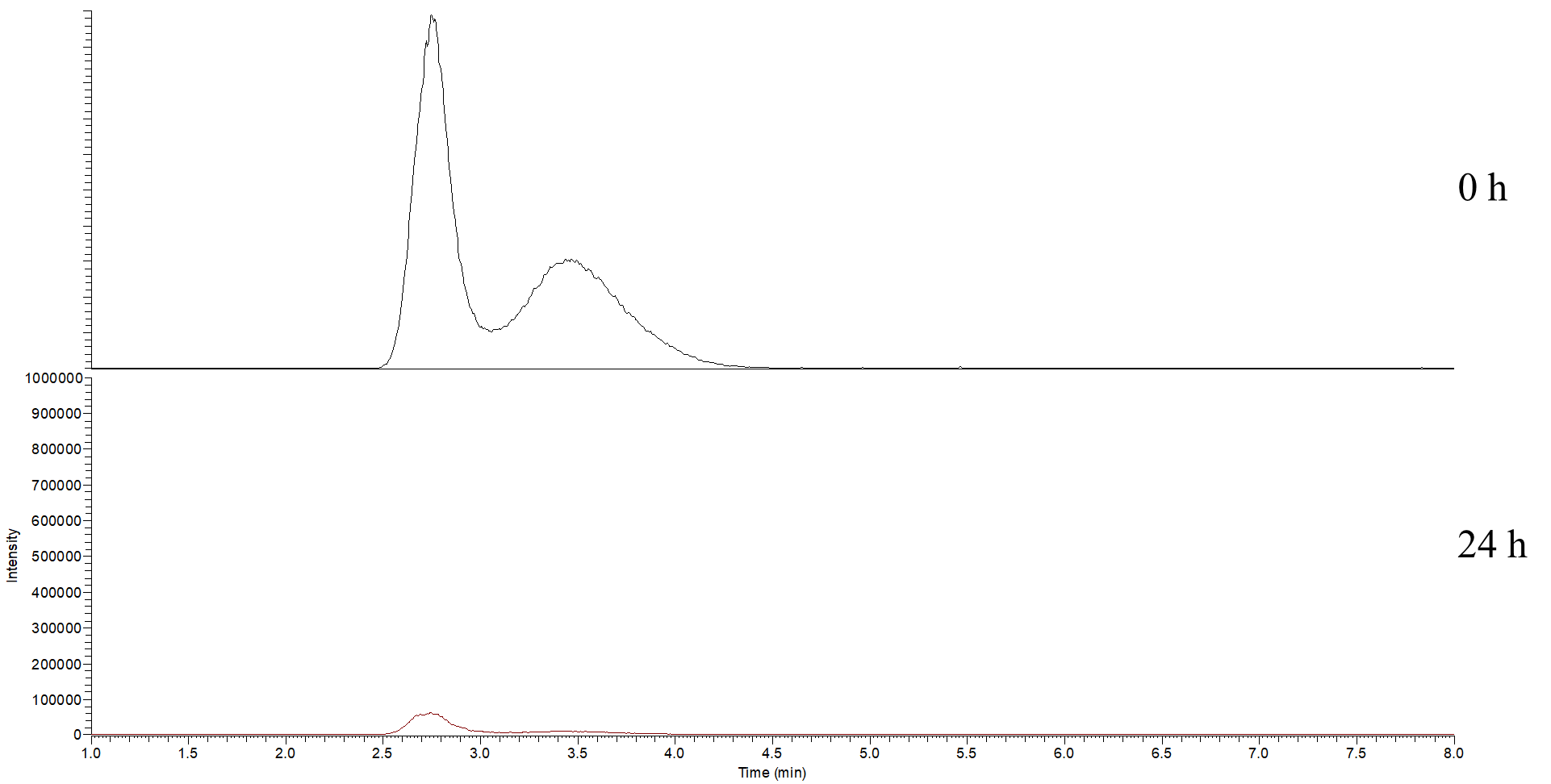


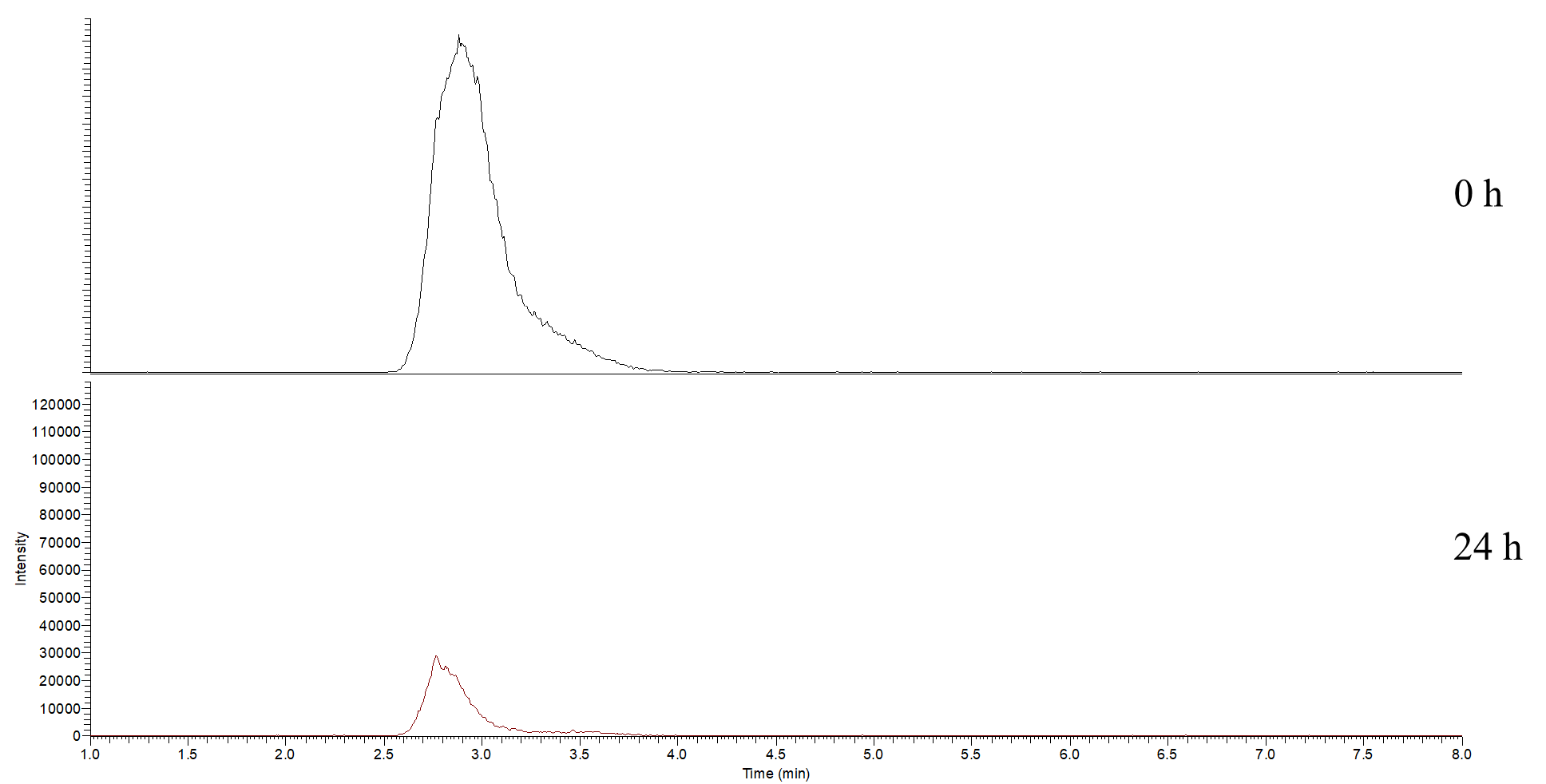


Figure S6. PGC-LC-MS elution patterns of selected *m/z* 300 (base peak) of 6S-GlcNAc before (0 h) and after (24 h) incubation with *A. muciniphila* lysate. Top and bottom chromatograms are replicate incubations.


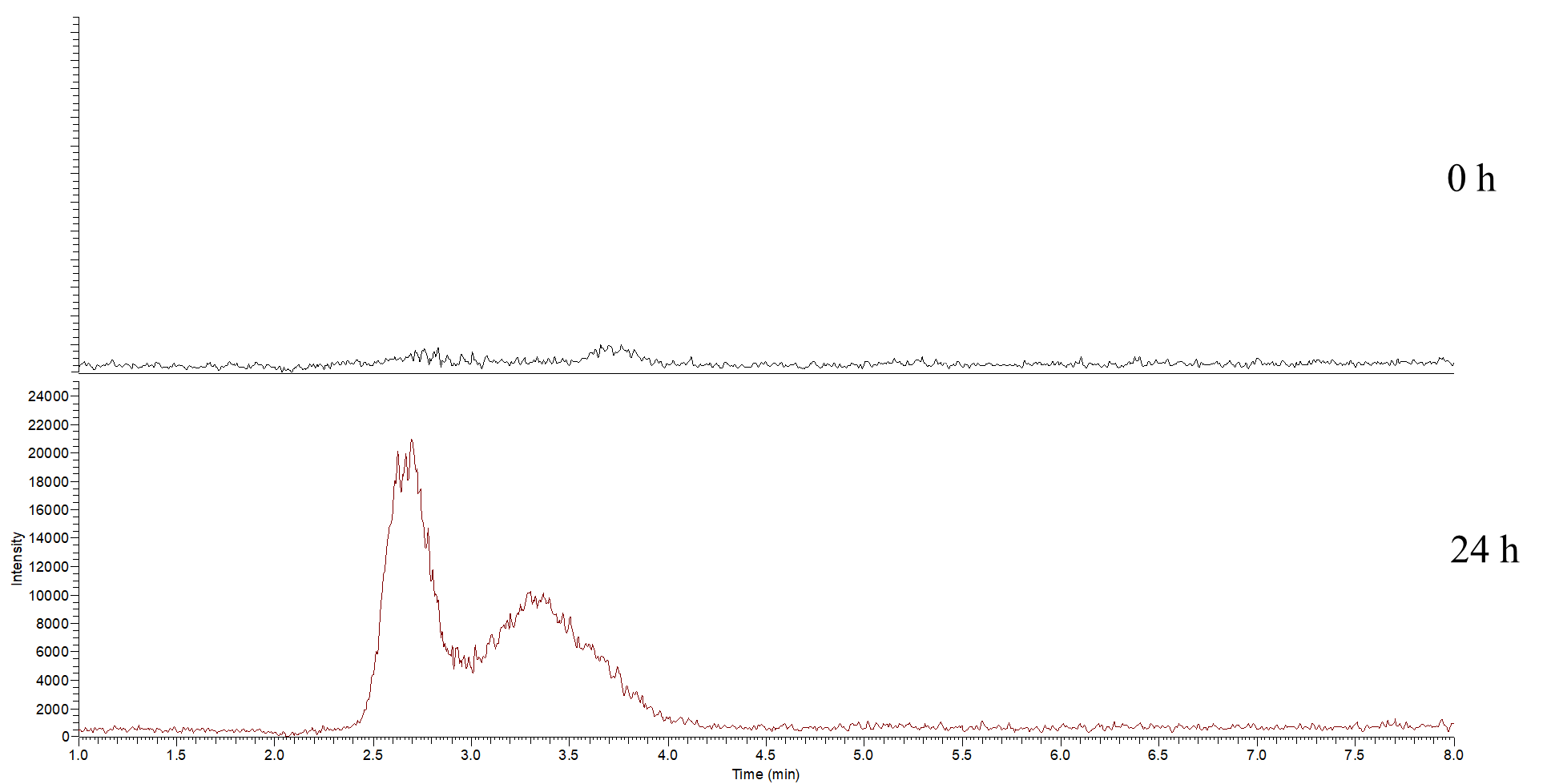


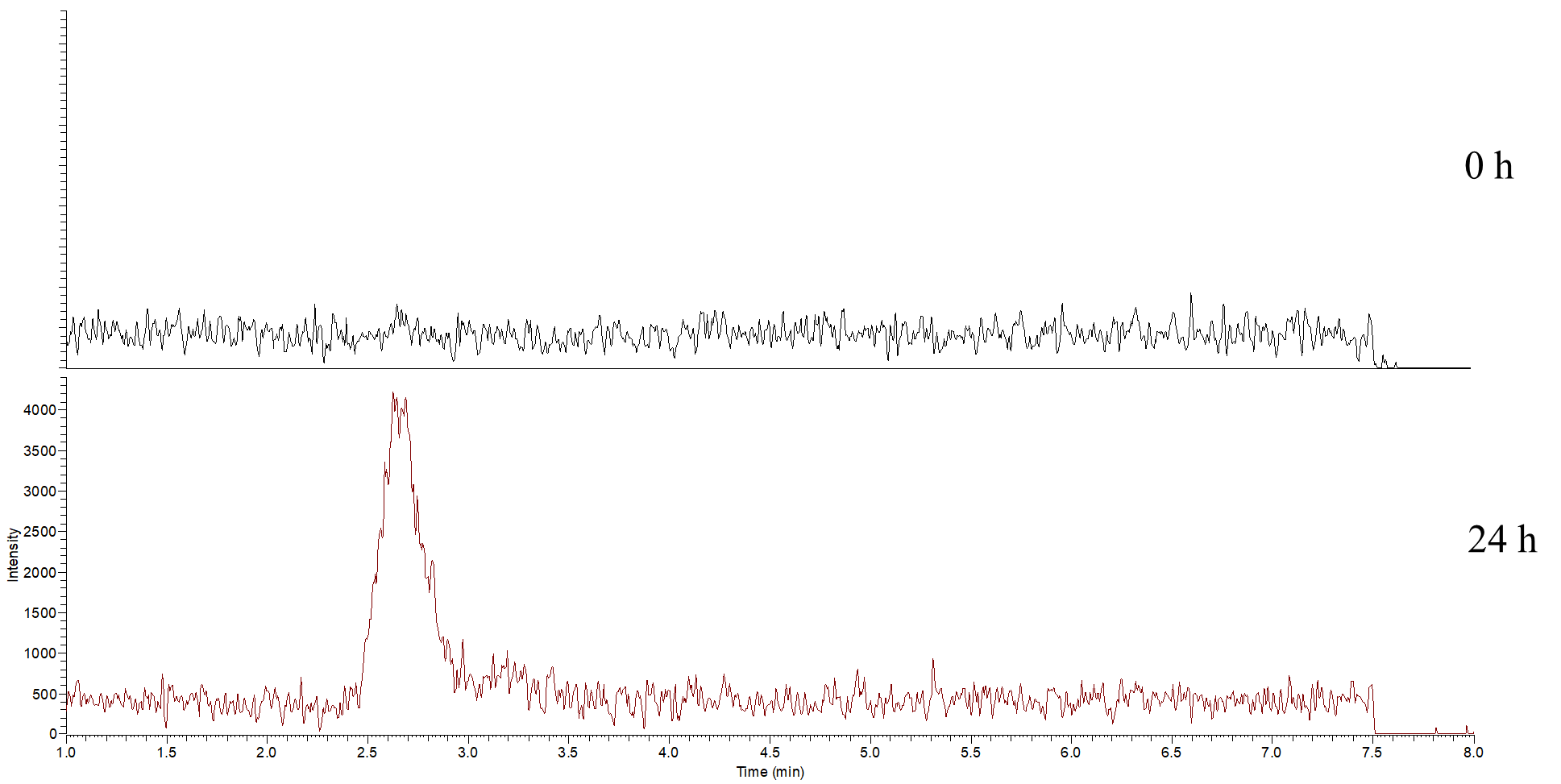


Figure S7. PGC-LC-MS elution patterns of selected *m/z* 220 (base peak – mass of sulphate) of 6S-GlcNAc before (0 h) and after (24 h) incubation with *A. muciniphila* lysate. Top and bottom chromatograms are replicate incubations.


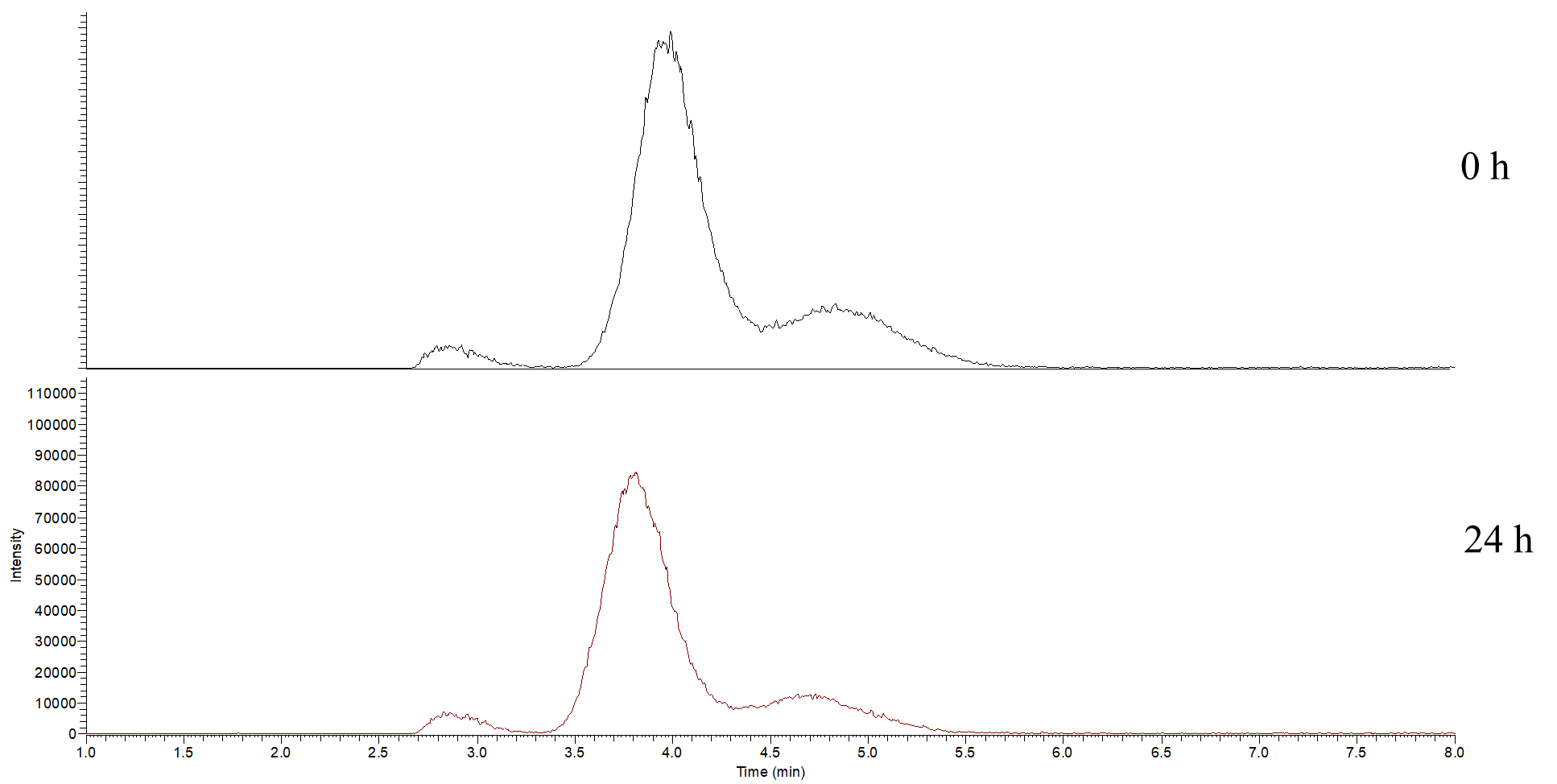


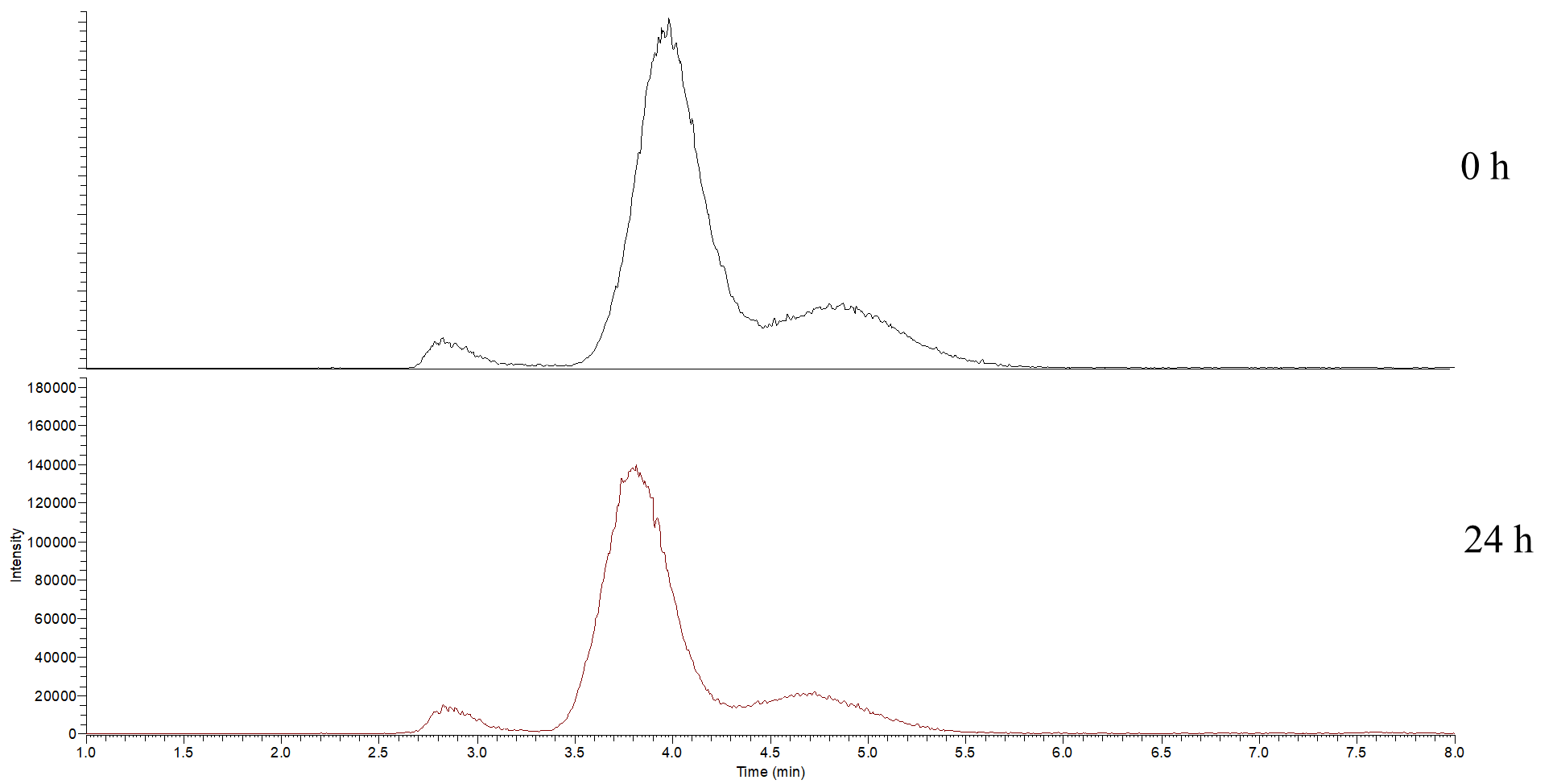


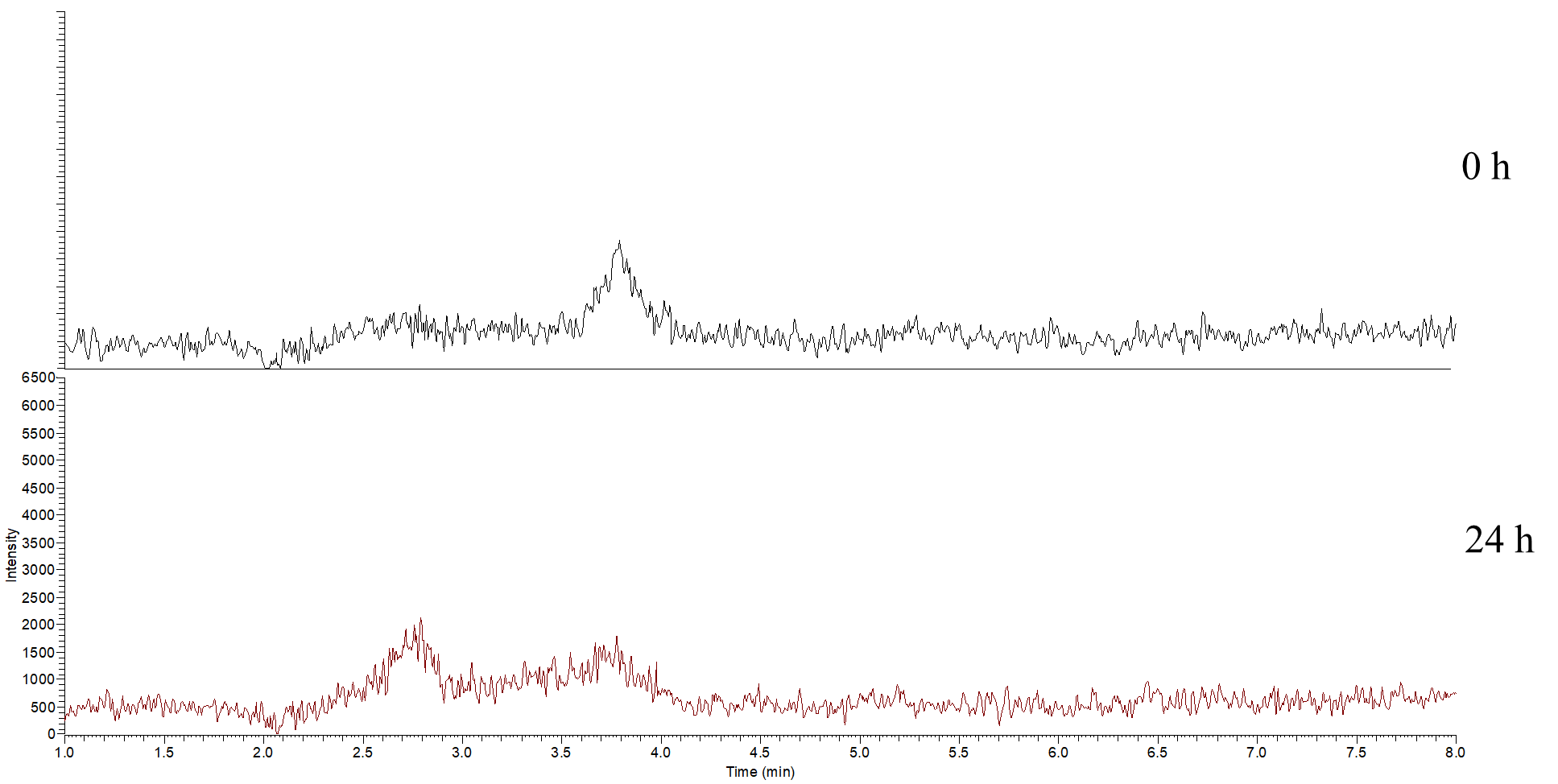


Figure S8. PGC-LC-MS elution patterns of selected *m/z* 380 (base peak), *m/z* 300 (base peak - mass of sulphate), and *m/z* 200 (base peak - 2x mass of sulphate) of 3S6S-GlcNAc before (0 h) and after (24 h) incubation with *A. muciniphila* lysate.


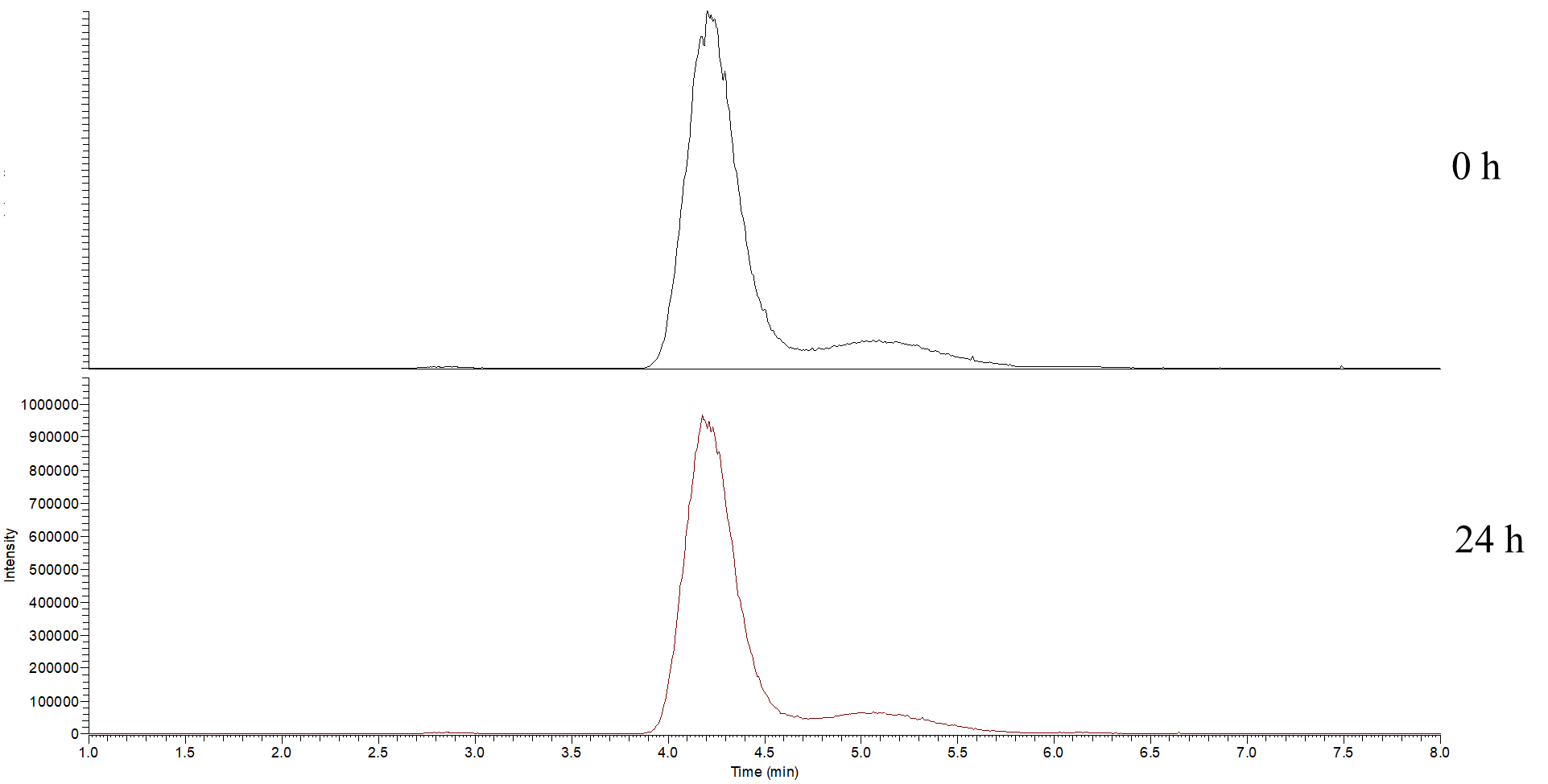


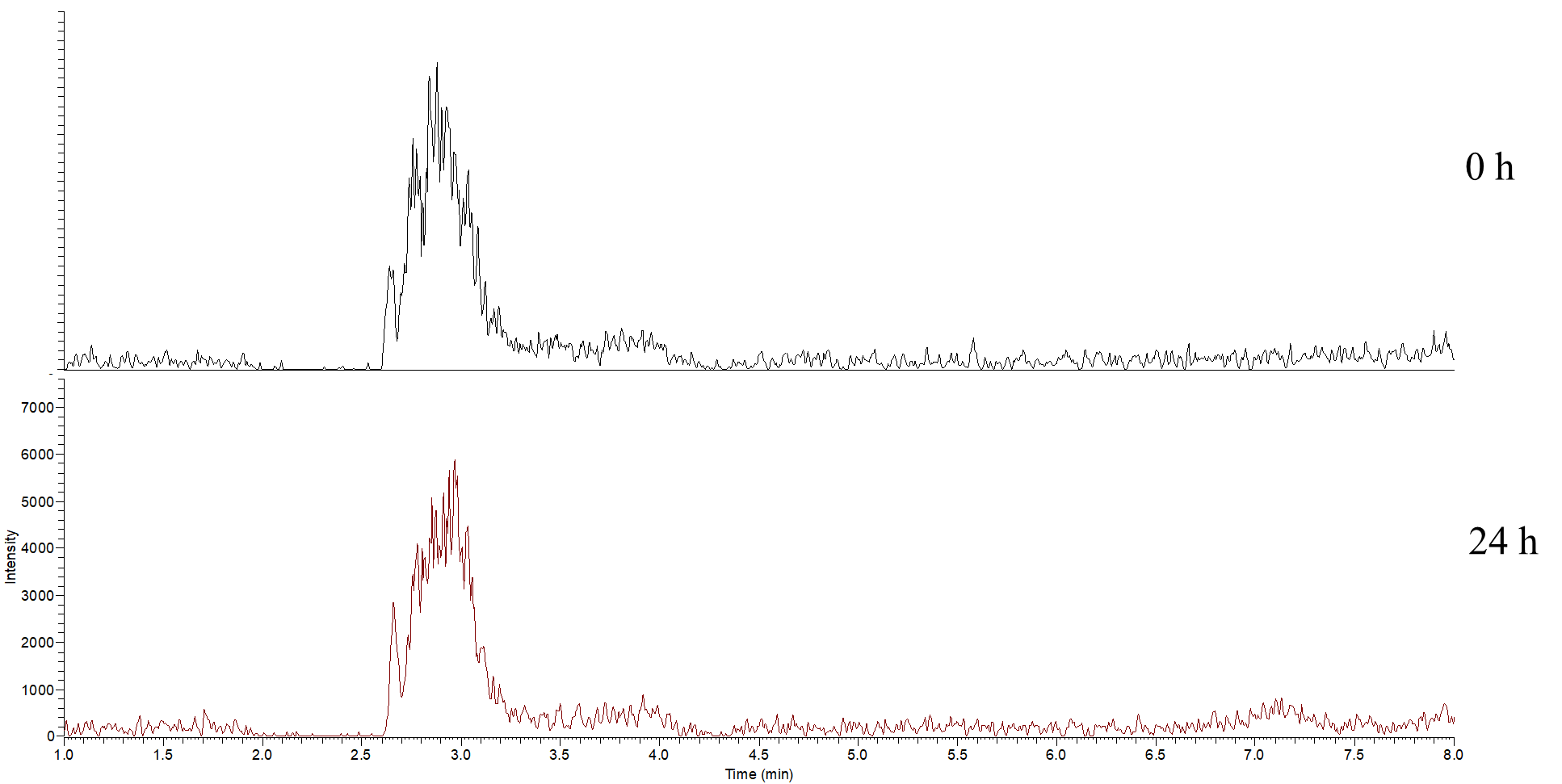


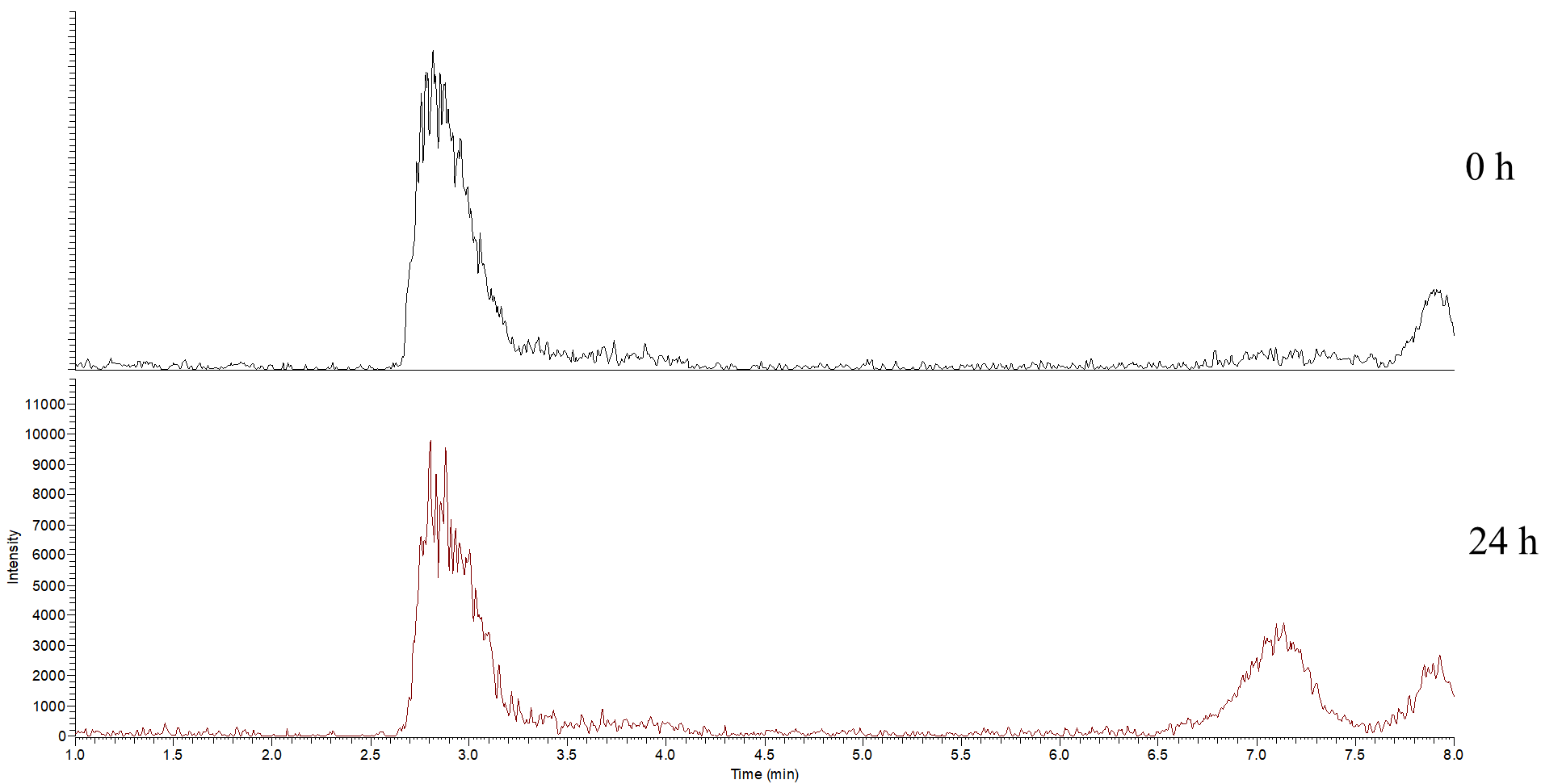


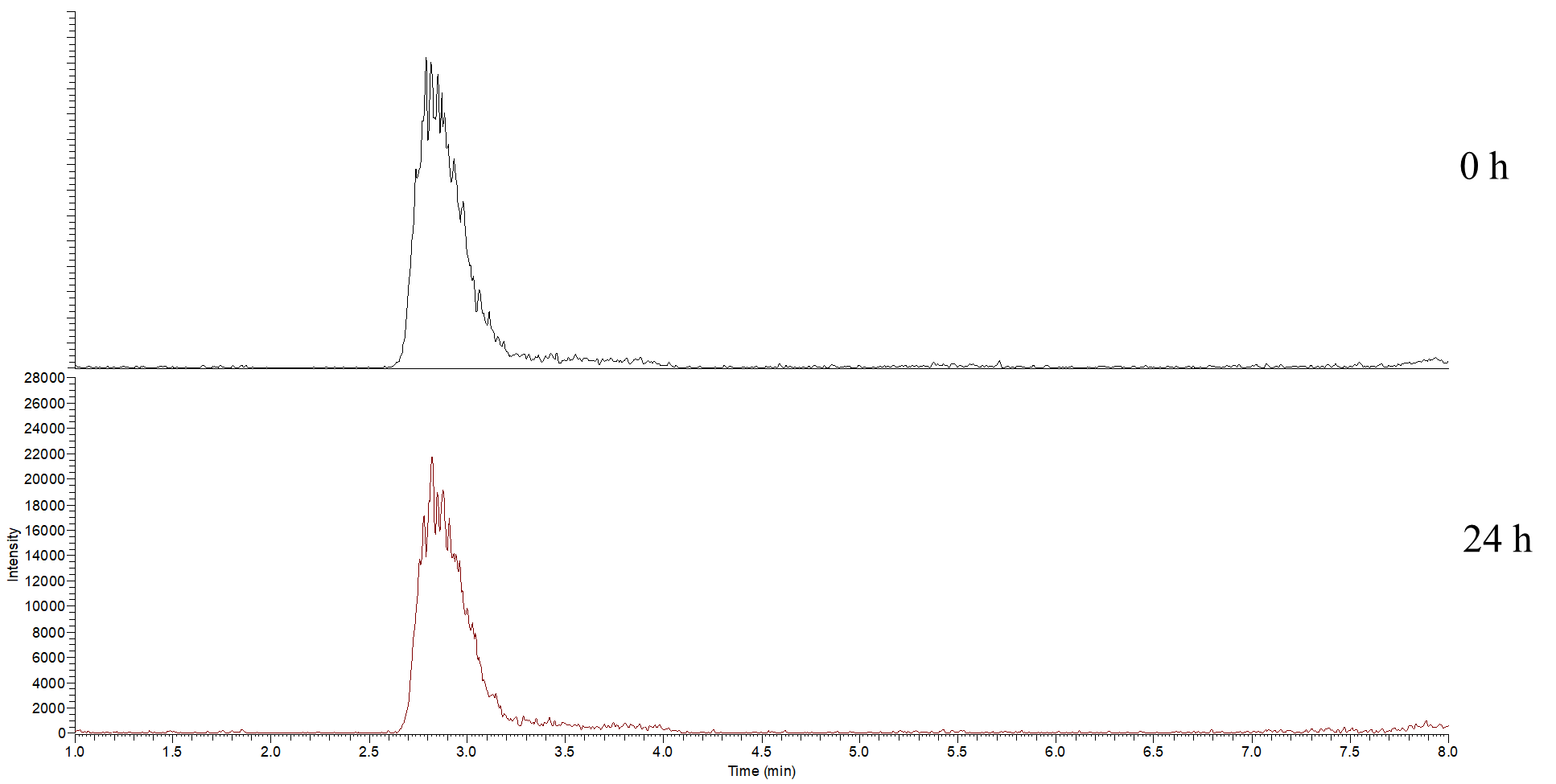


Figure S9. PGC-LC-MS elution patterns of selected *m/z* 608 (base peak), *m/z* 582 (base peak - mass of sulphate), *m/z* 462 (base peak - mass of Fuc), and *m/z* 382 (base peak - mass of sulphate and fucose) of 6’-*O*-sulphated Lewis a before (0 h) and after (24 h) incubation with *A. muciniphila* lysate.


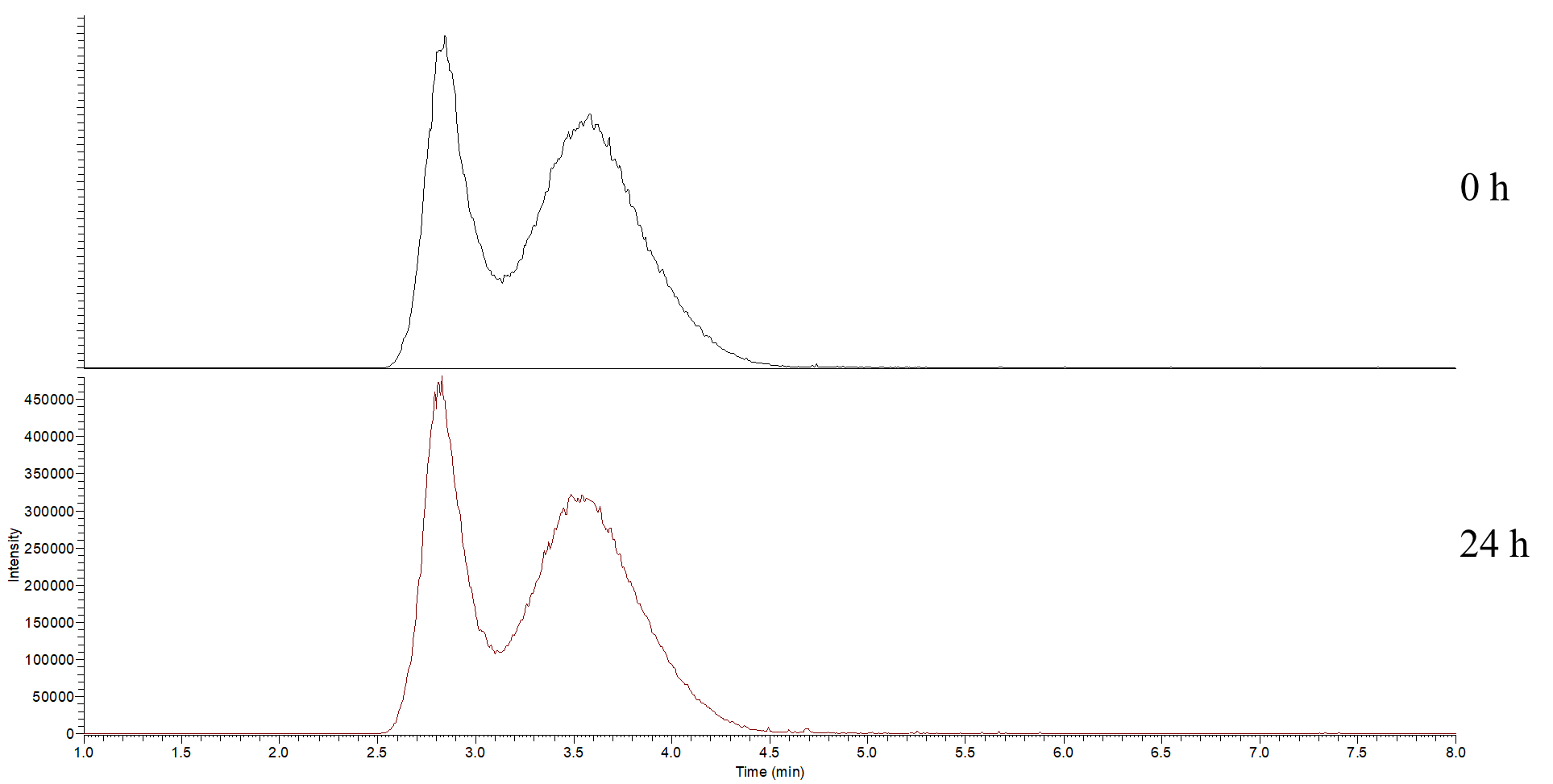


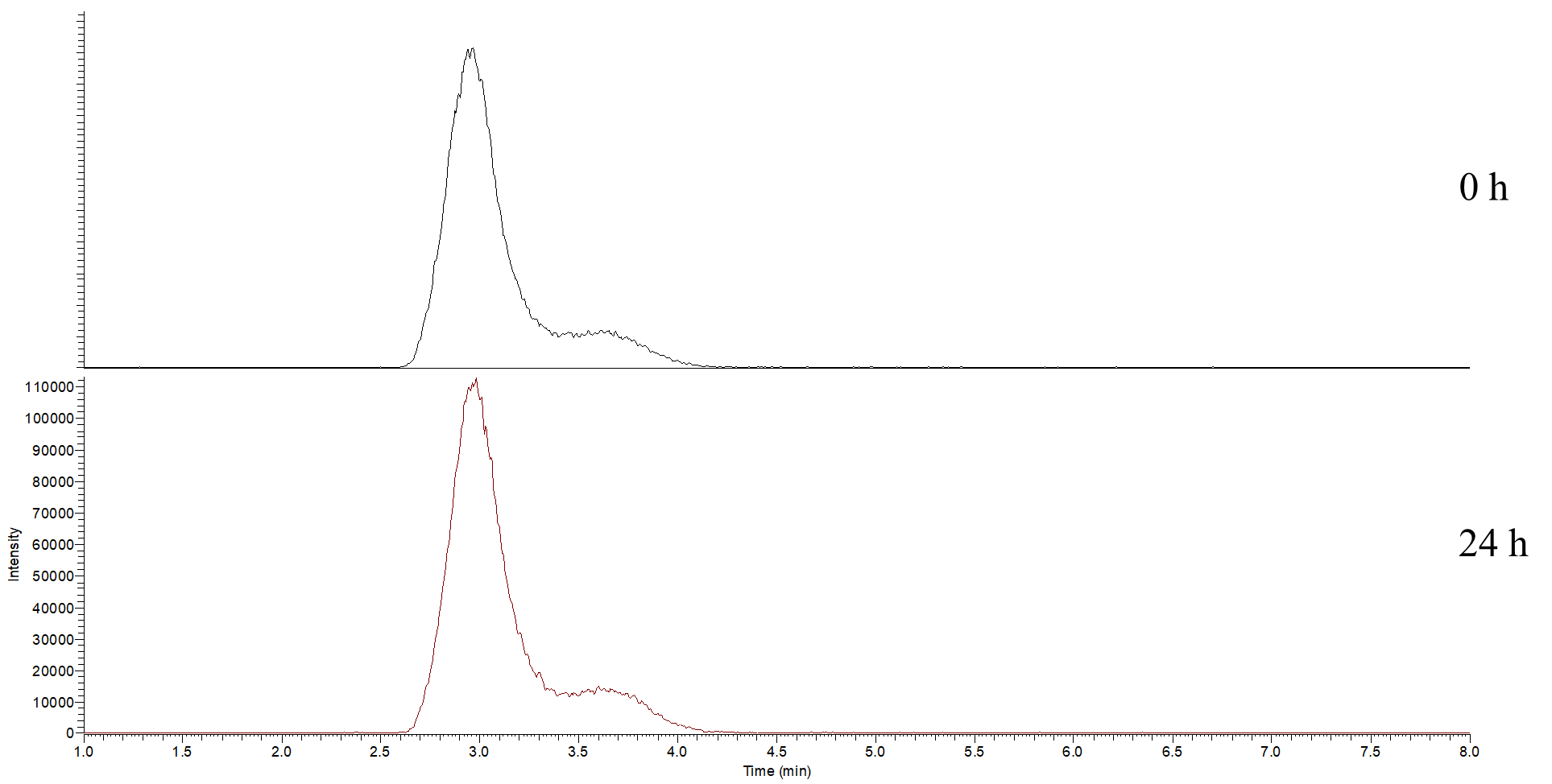


Figure S10. PGC-LC-MS elution patterns of selected *m/z* 300 (base peak) of 6S-GlcNAc before (0 h) and after (24 h) incubation with *R. torques* lysate. Top and bottom chromatograms are replicate incubations.


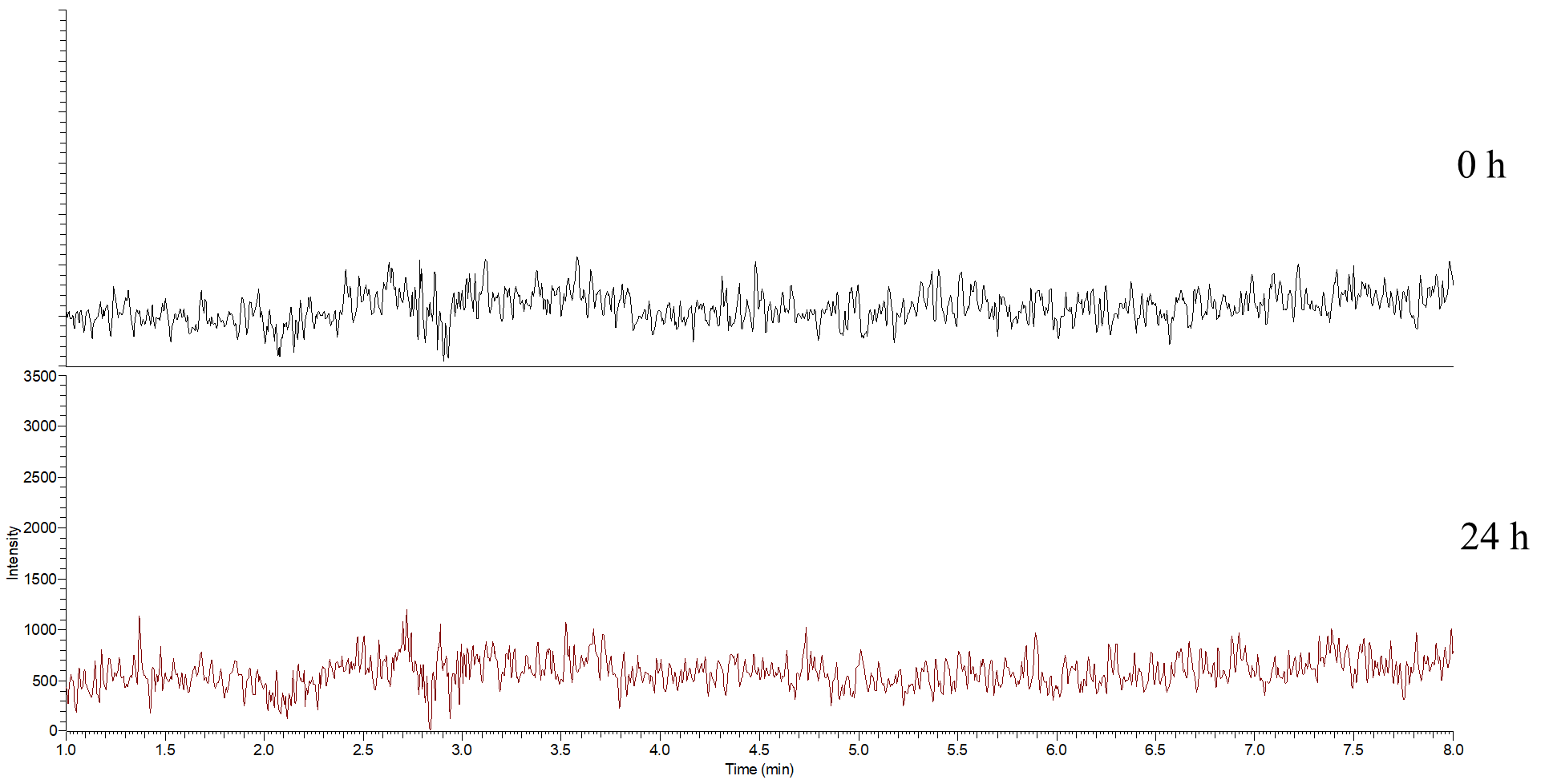


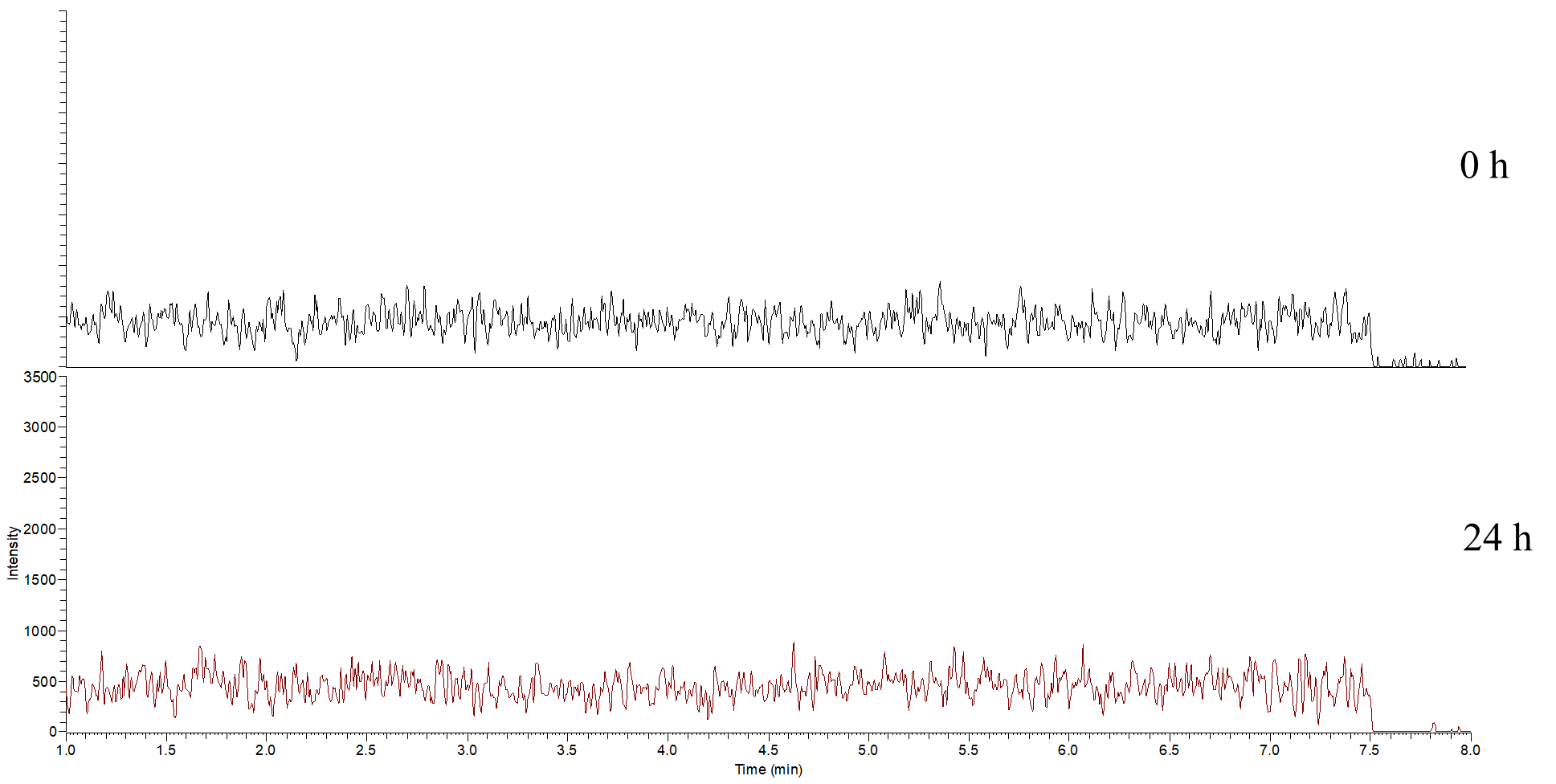


Figure S11. PGC-LC-MS elution patterns of selected *m/z* 220 (base peak – mass of sulphate) of 6S-GlcNAc before (0 h) and after (24 h) incubation with *R. torques* lysate. Top and bottom chromatograms are replicate incubations.


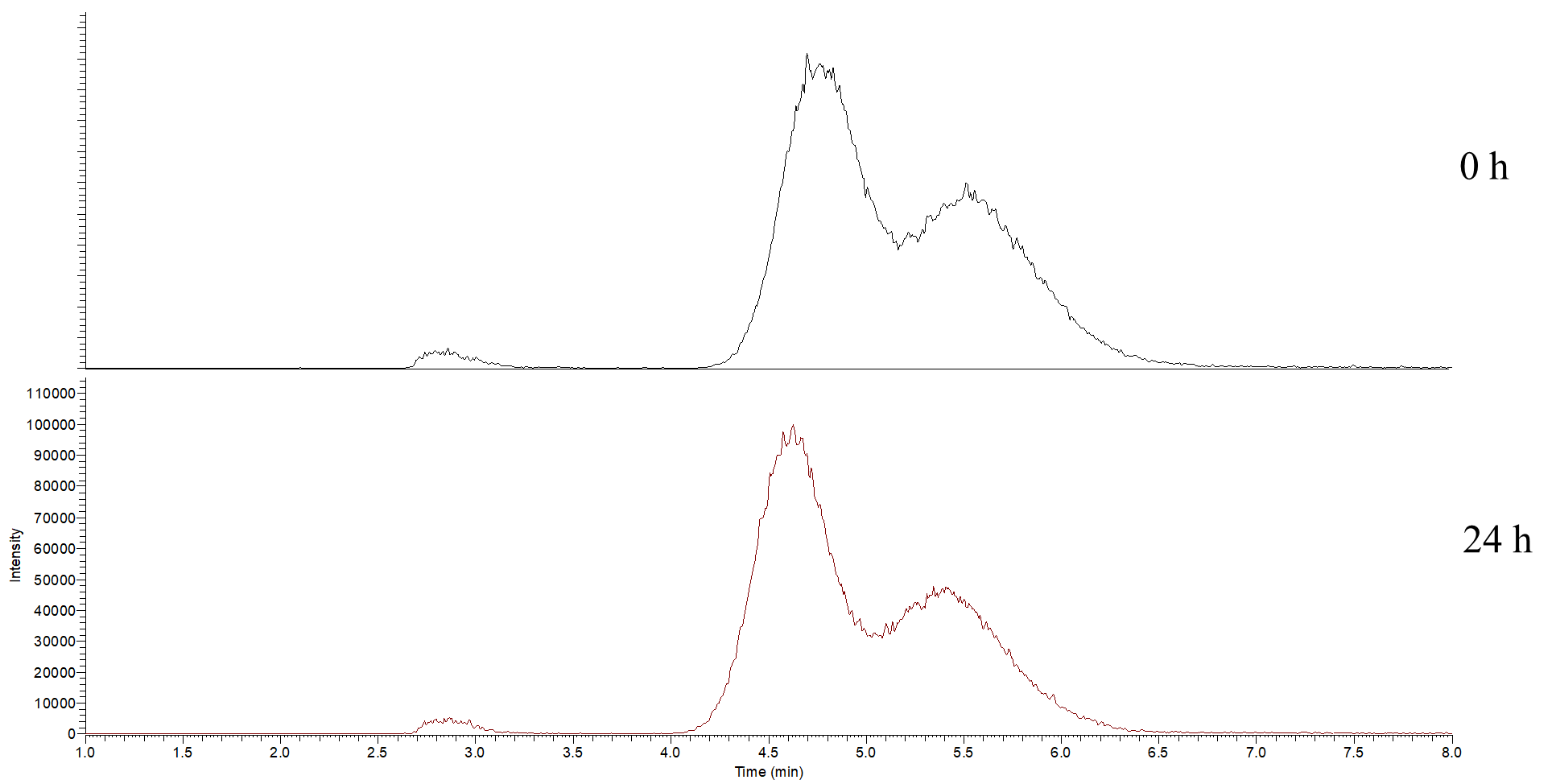


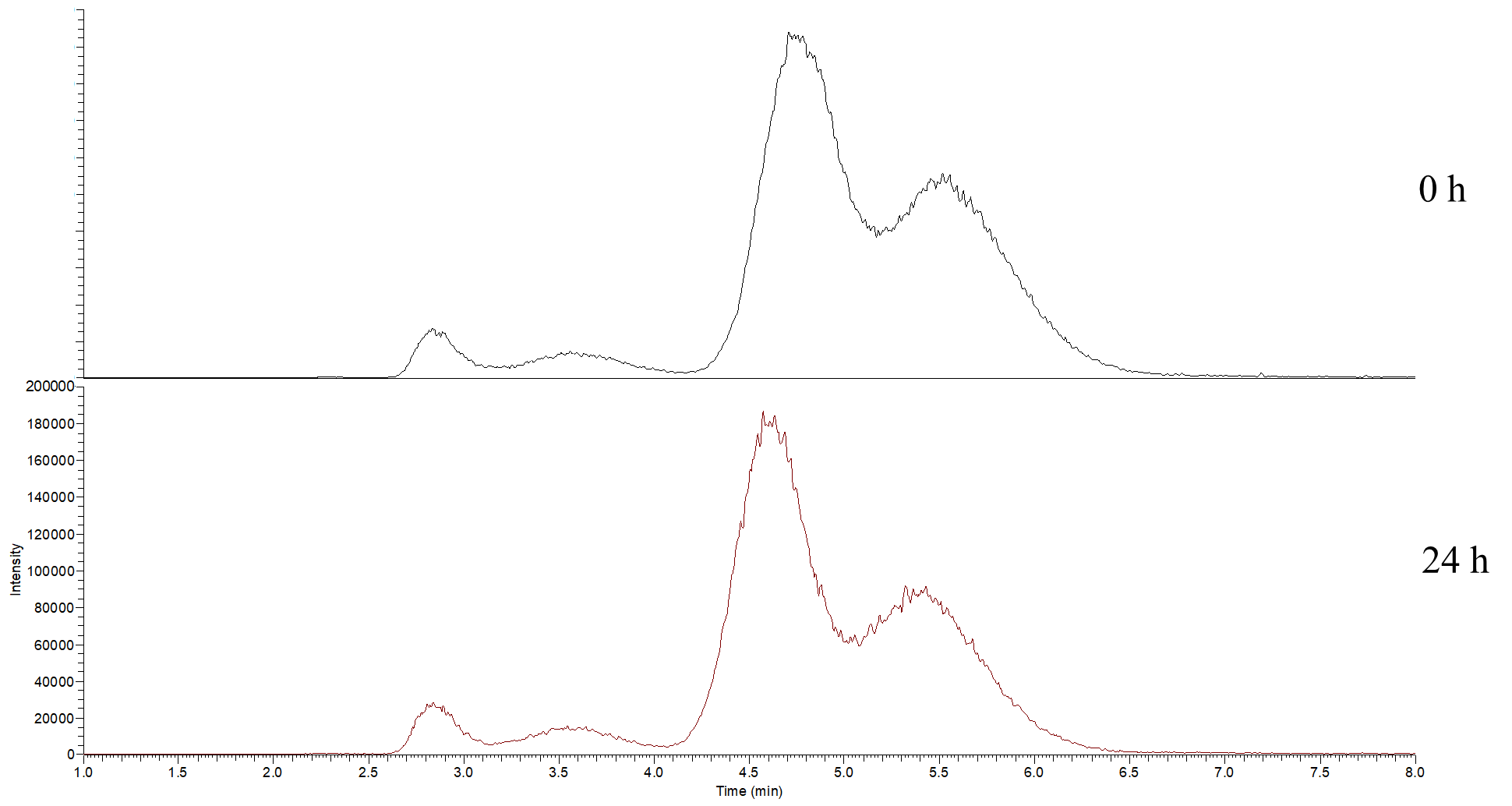


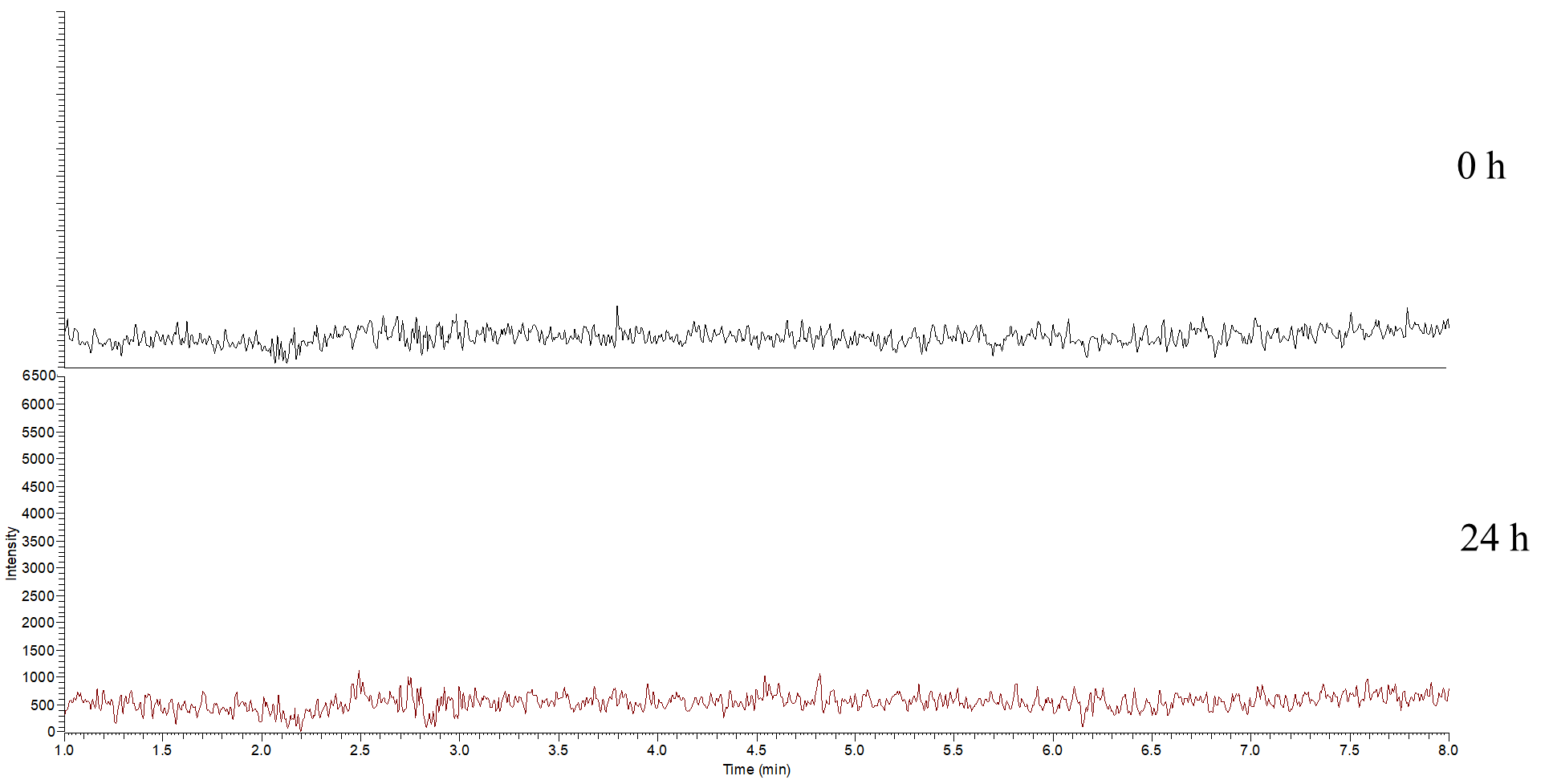


Figure S12. PGC-LC-MS elution patterns of selected *m/z* 380 (base peak), *m/z* 300 (base peak - mass of sulphate), and *m/z* 200 (base peak - 2x mass of sulphate) of 3S6S-GlcNAc before (0 h) and after (24 h) incubation with *R. torques* lysate.


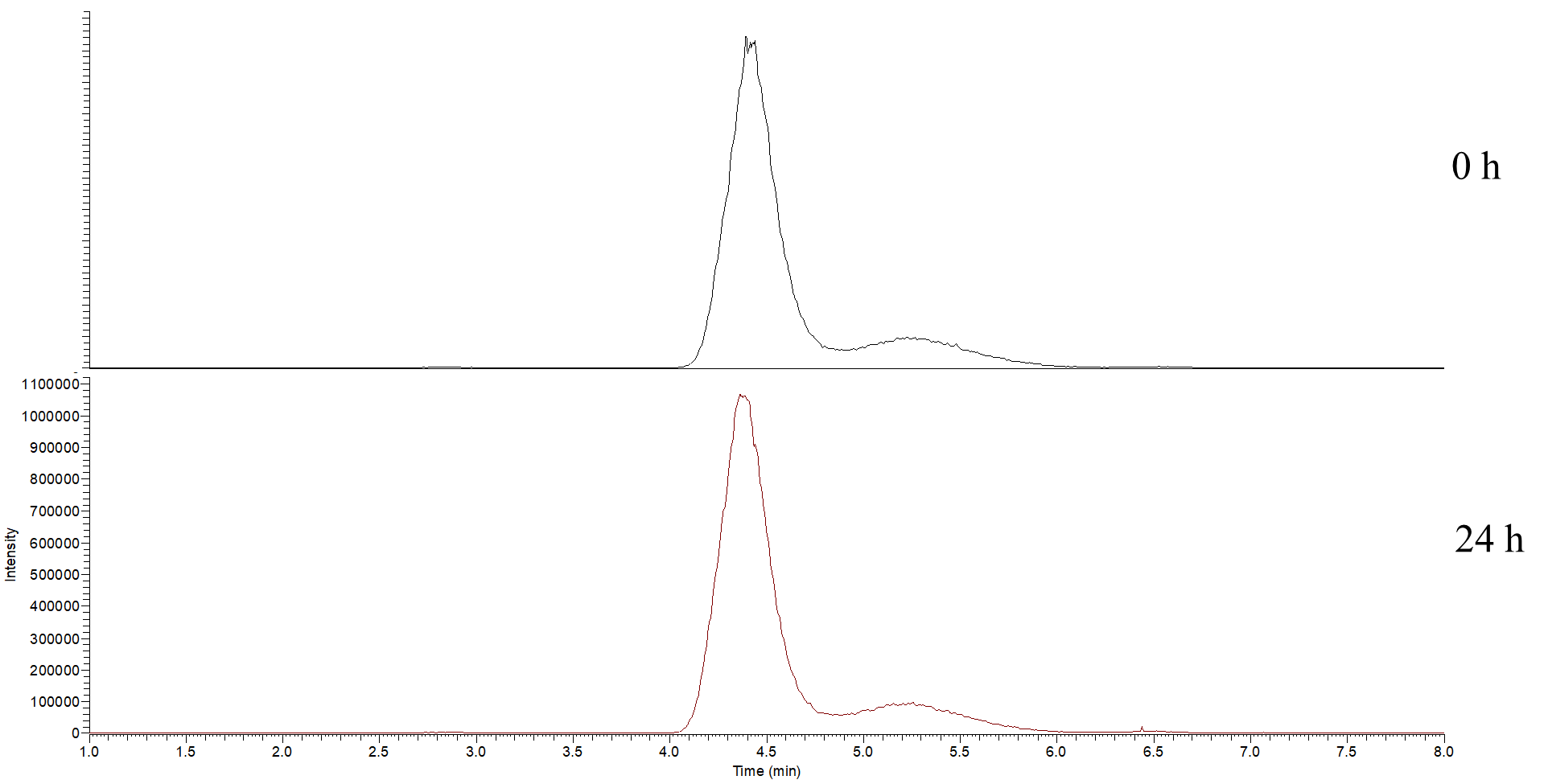


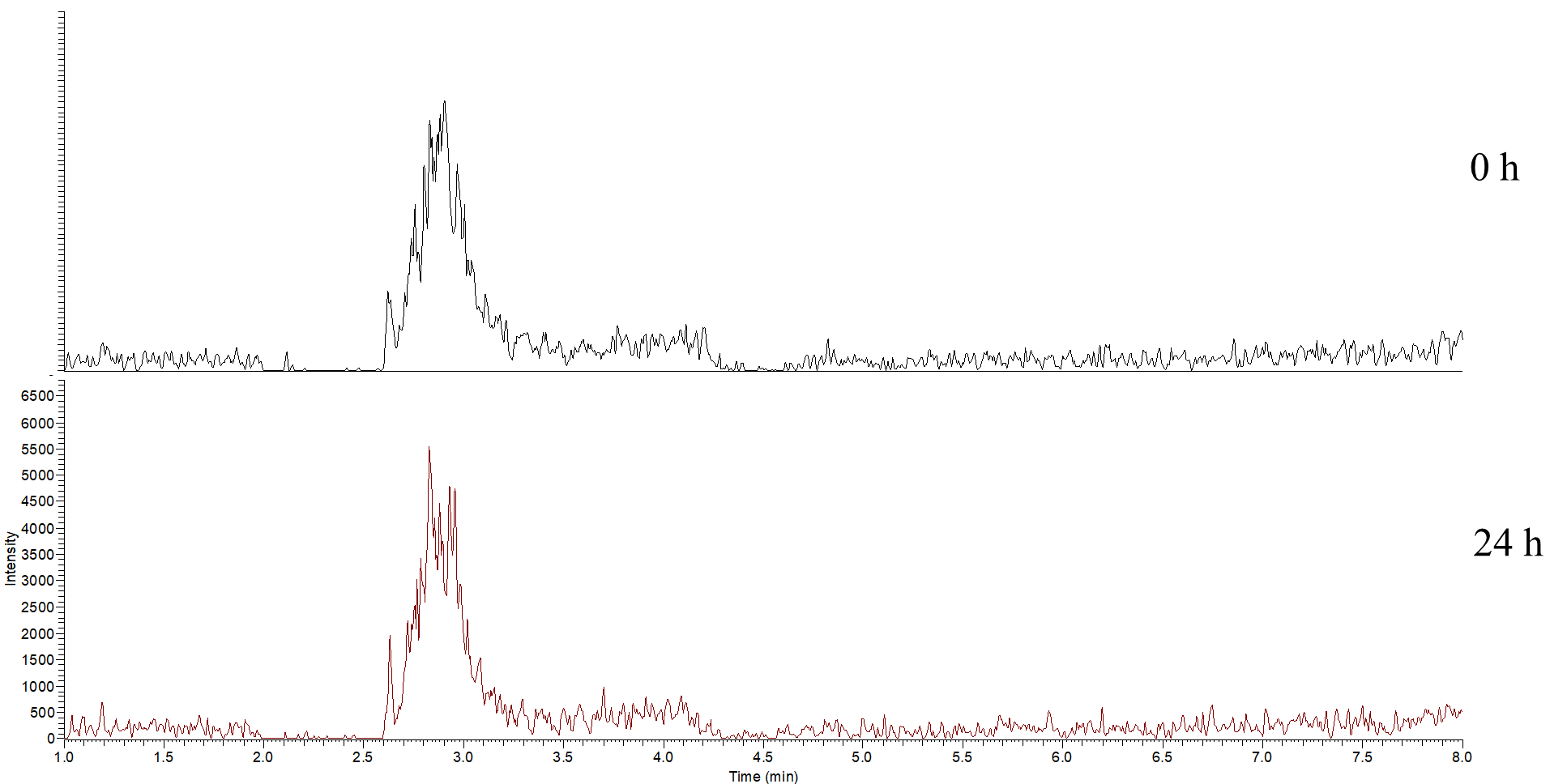


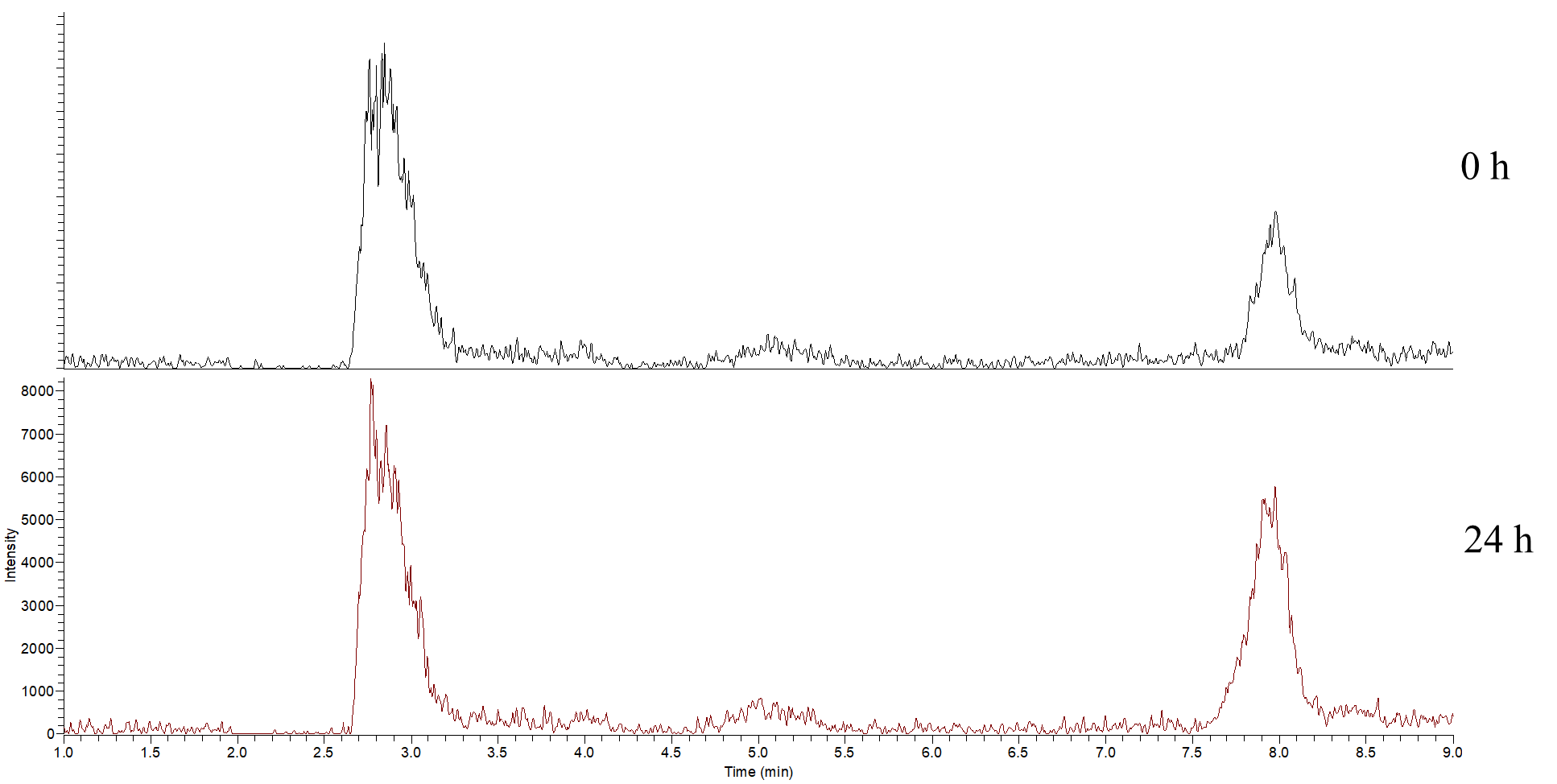


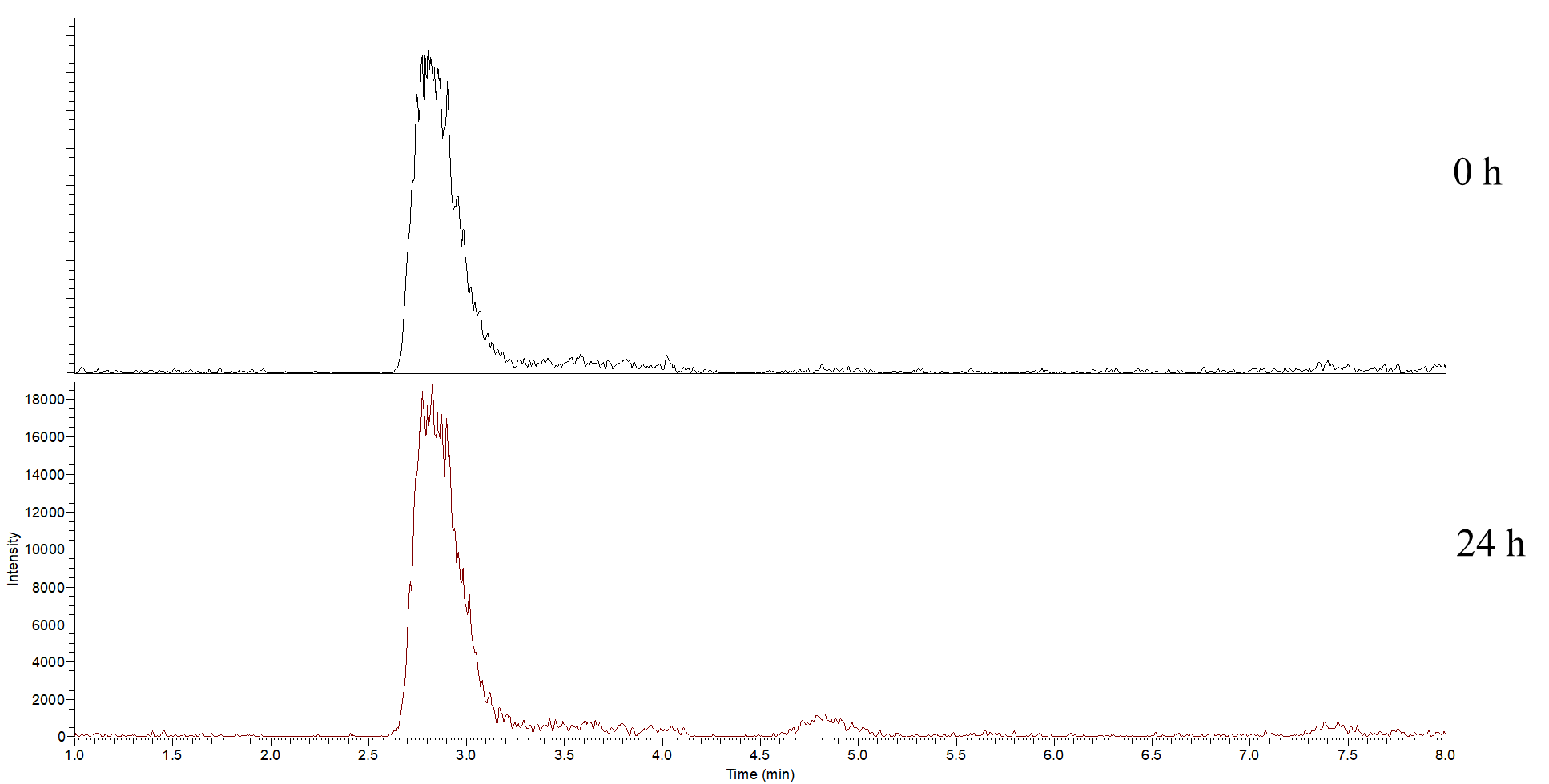


Figure S13. PGC-LC-MS elution patterns of selected *m/z* 608 (base peak), *m/z* 582 (base peak - mass of sulphate), *m/z* 462 (base peak - mass of Fuc), and *m/z* 382 (base peak - mass of sulphate and fucose) of 6’-*O*-sulphated Lewis a before (0 h) and after (24 h) incubation with *R. torques* lysate.


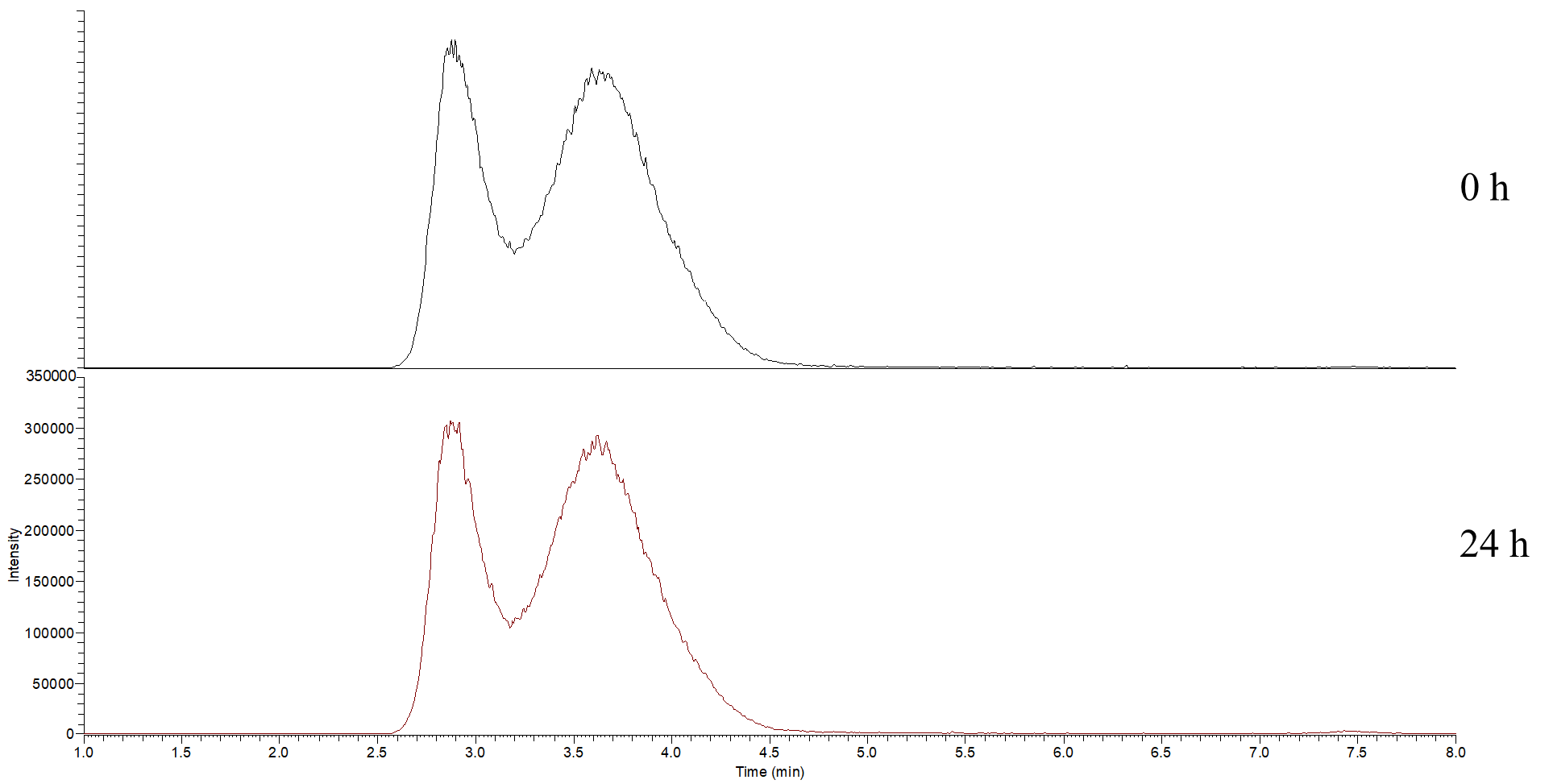


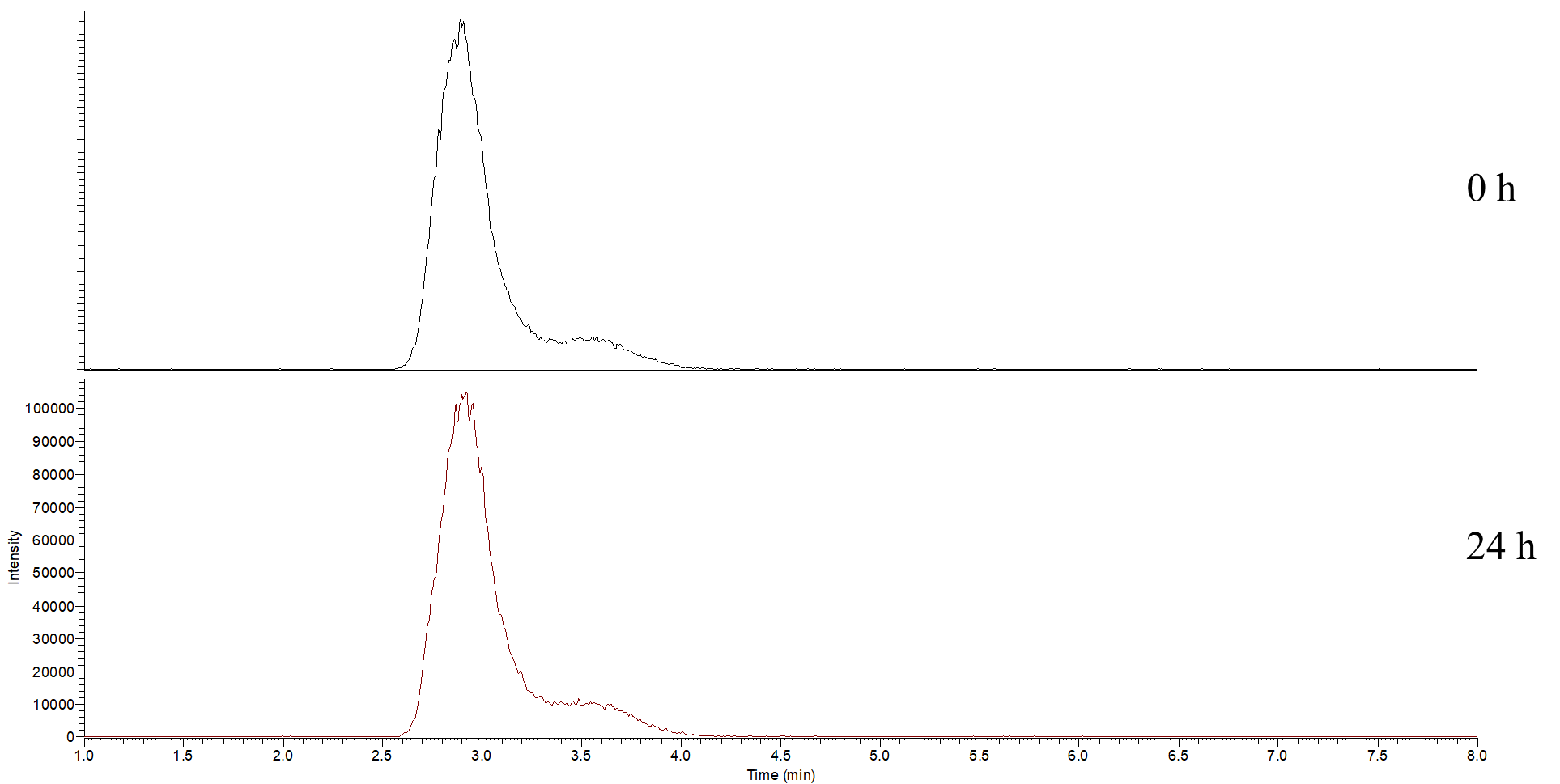


Figure S14. PGC-LC-MS elution patterns of selected *m/z* 300 (base peak) of 6S-GlcNAc before (0 h) and after (24 h) incubation with *B. thetaiotaomicron* lysate. Top and bottom chromatograms are replicate incubations.


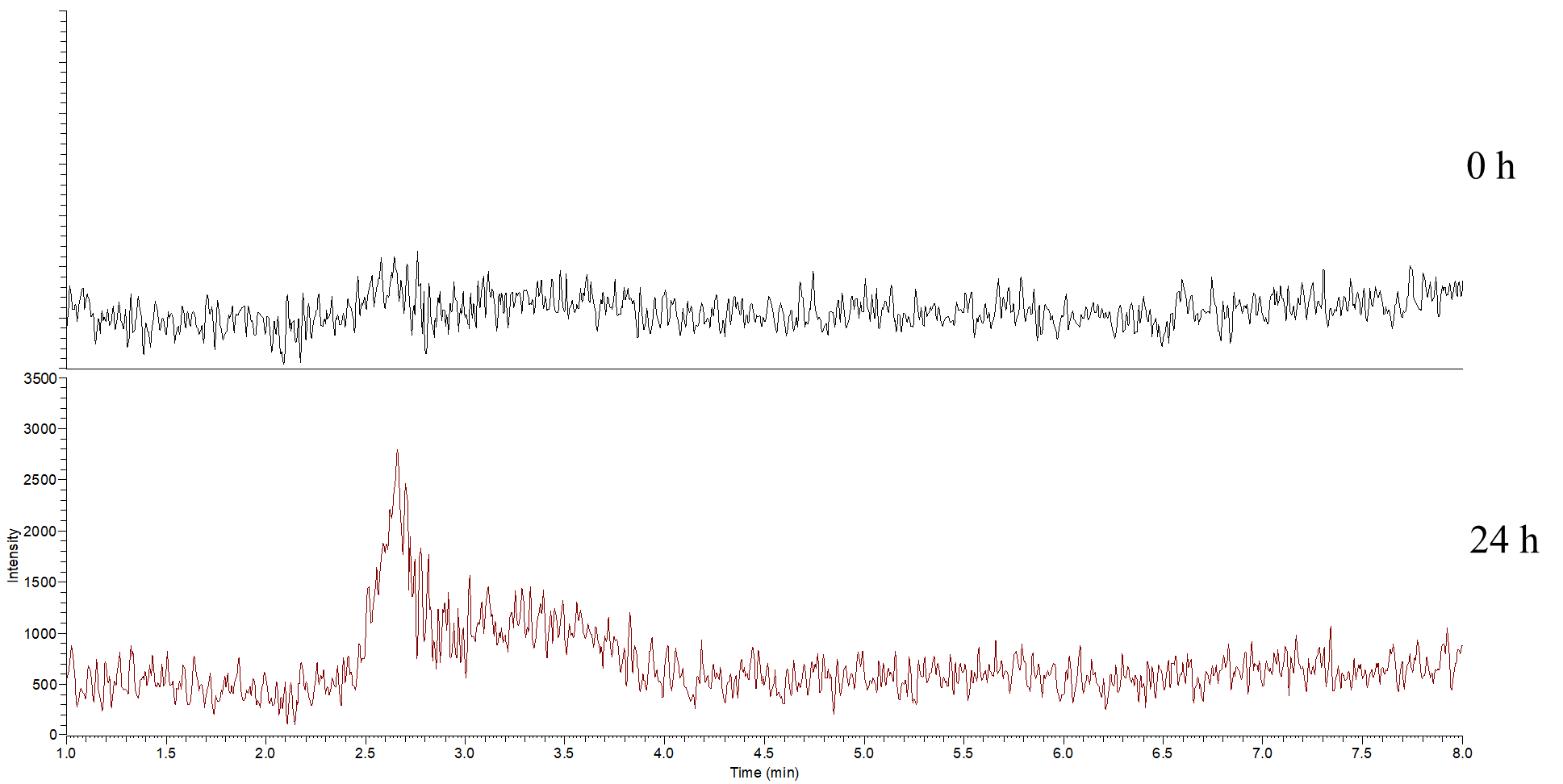


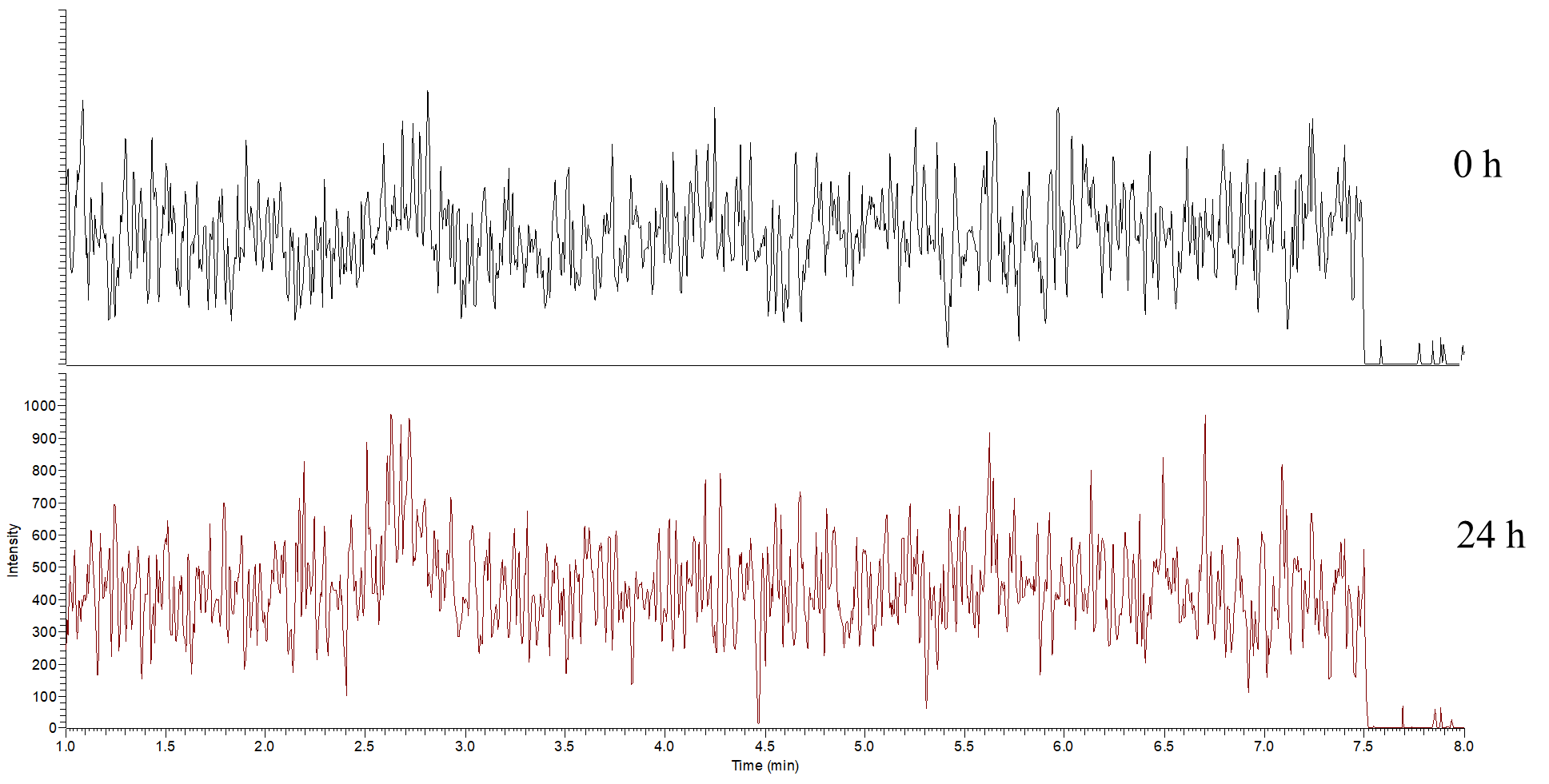


Figure S15. PGC-LC-MS elution patterns of selected *m/z* 220 (base peak – mass of sulphate) of 6S-GlcNAc before (0 h) and after (24 h) incubation with *B. thetaiotaomicron* lysate. Top and bottom chromatograms are replicate incubations.


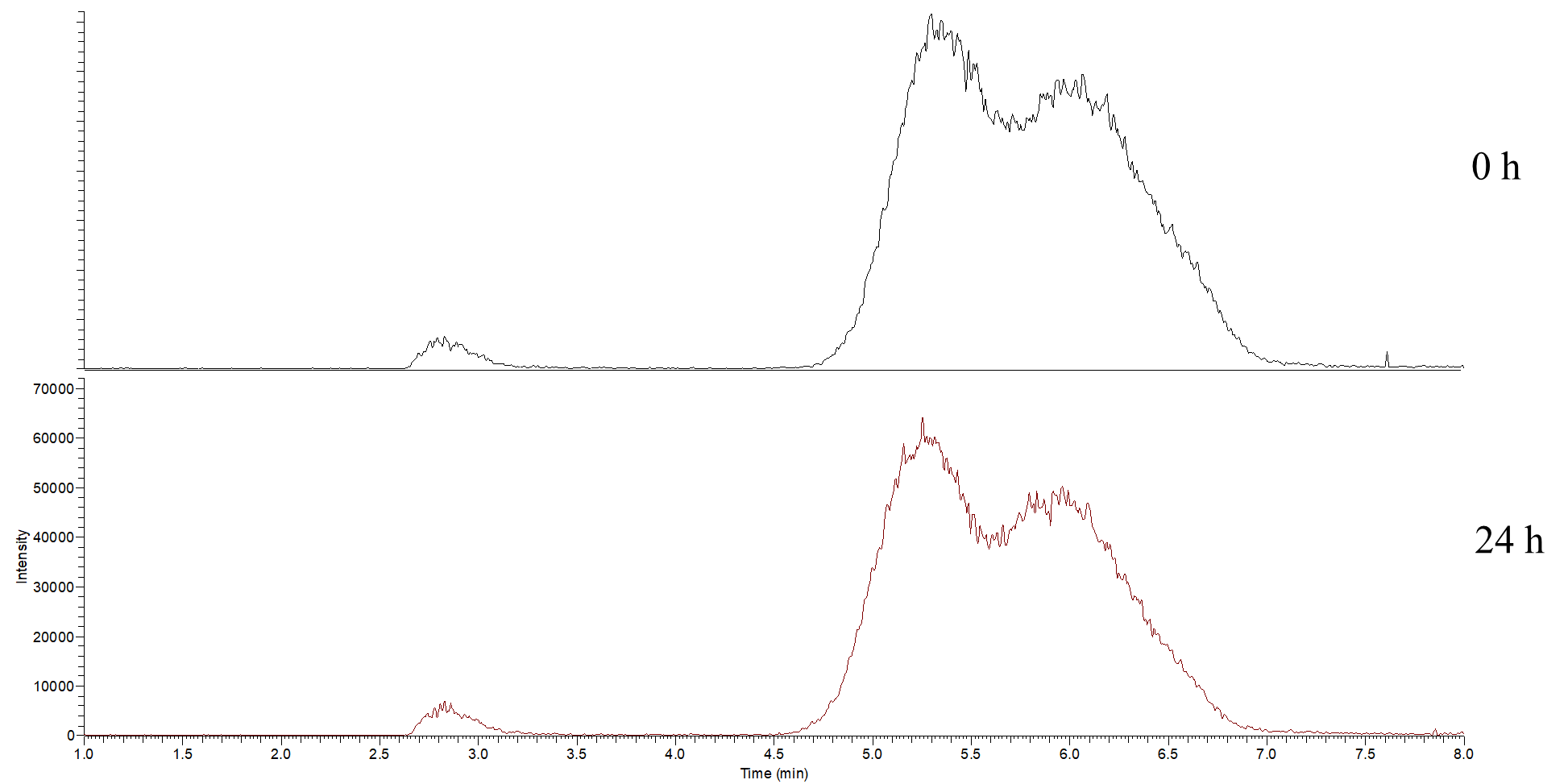


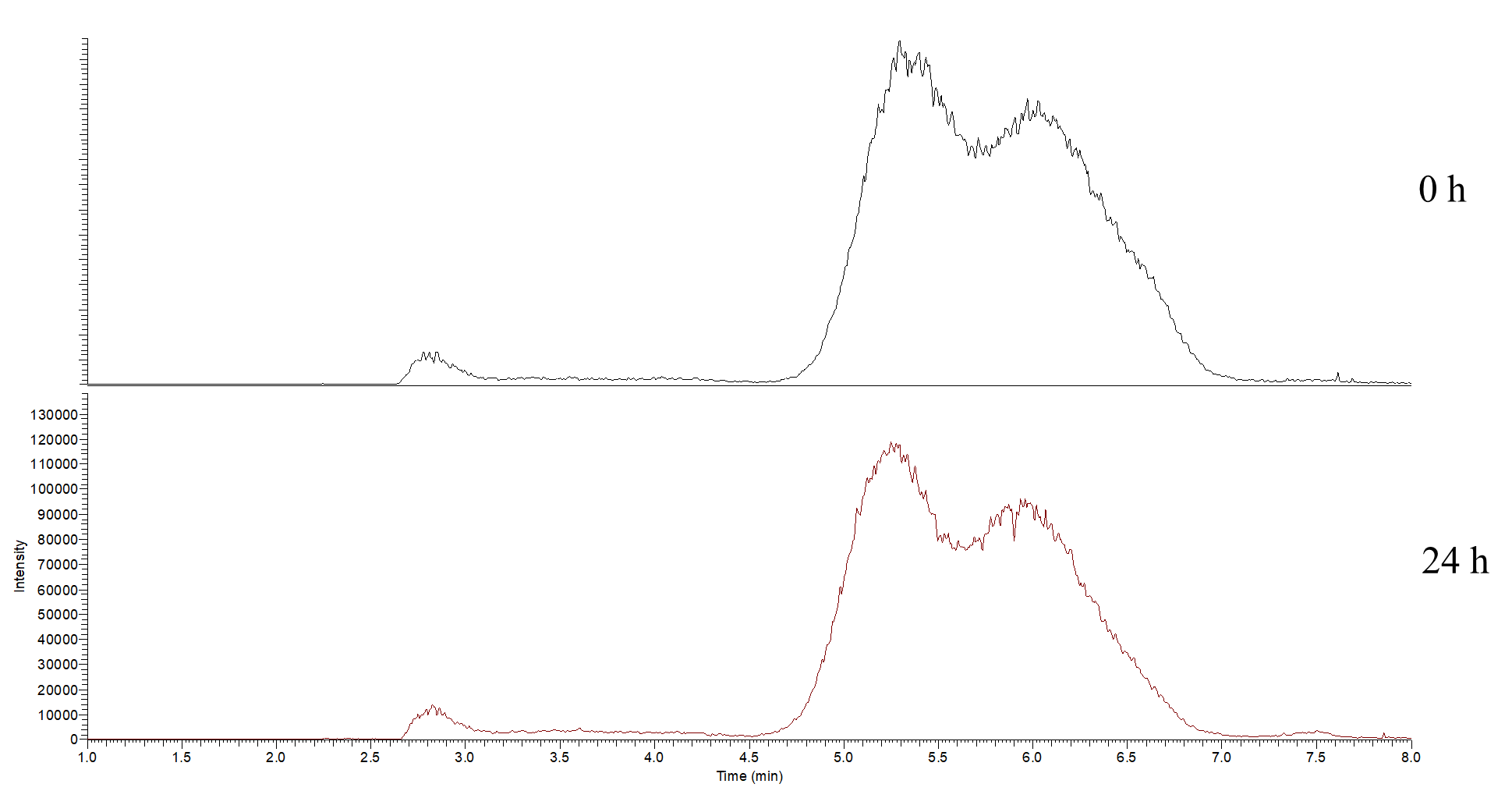


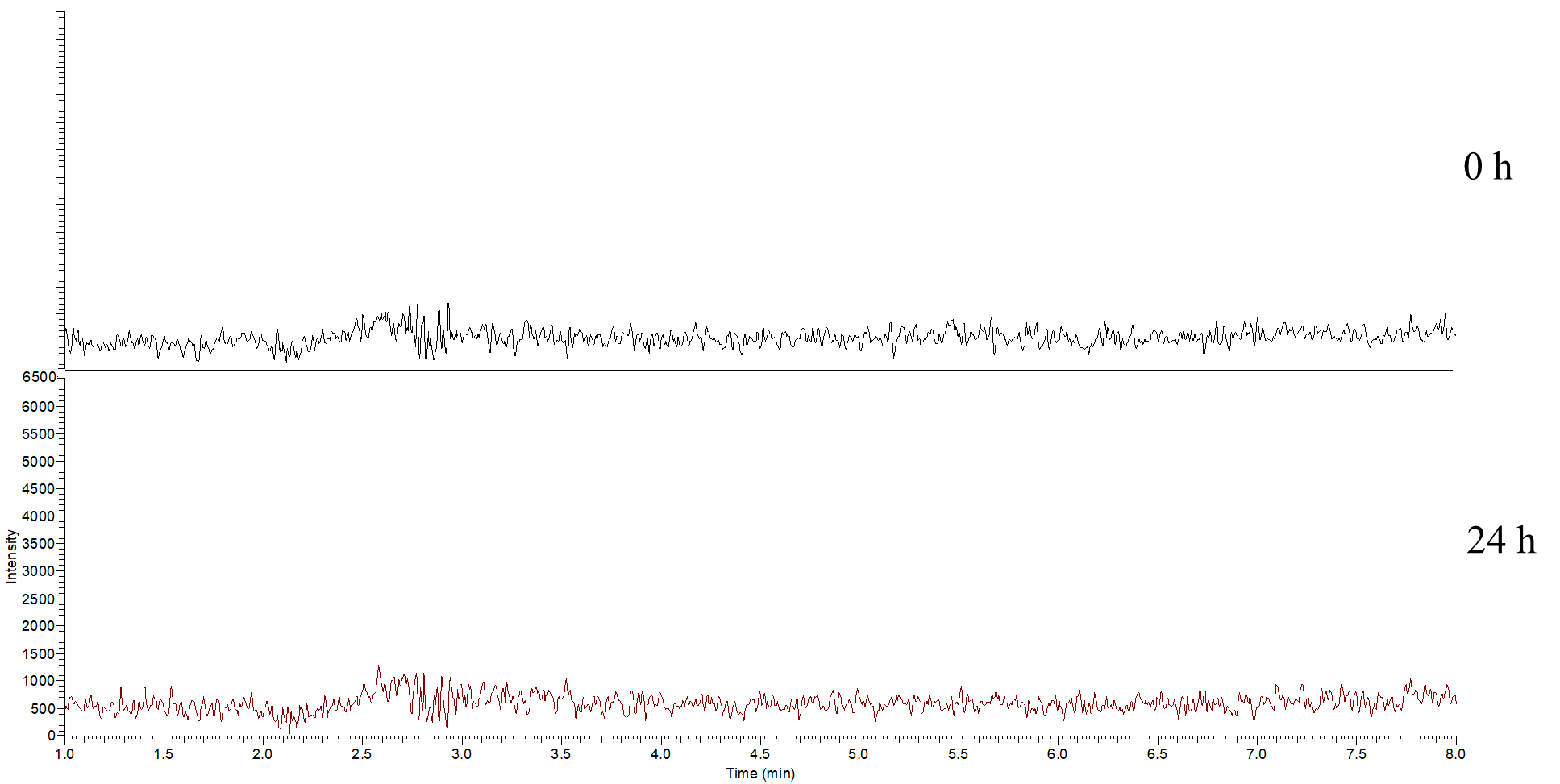


Figure S16. PGC-LC-MS elution patterns of selected *m/z* 380 (base peak), *m/z* 300 (base peak - mass of sulphate), and *m/z* 200 (base peak - 2x mass of sulphate) of 3S6S-GlcNAc before (0 h) and after (24 h) incubation with *B. thetaiotaomicron* lysate.


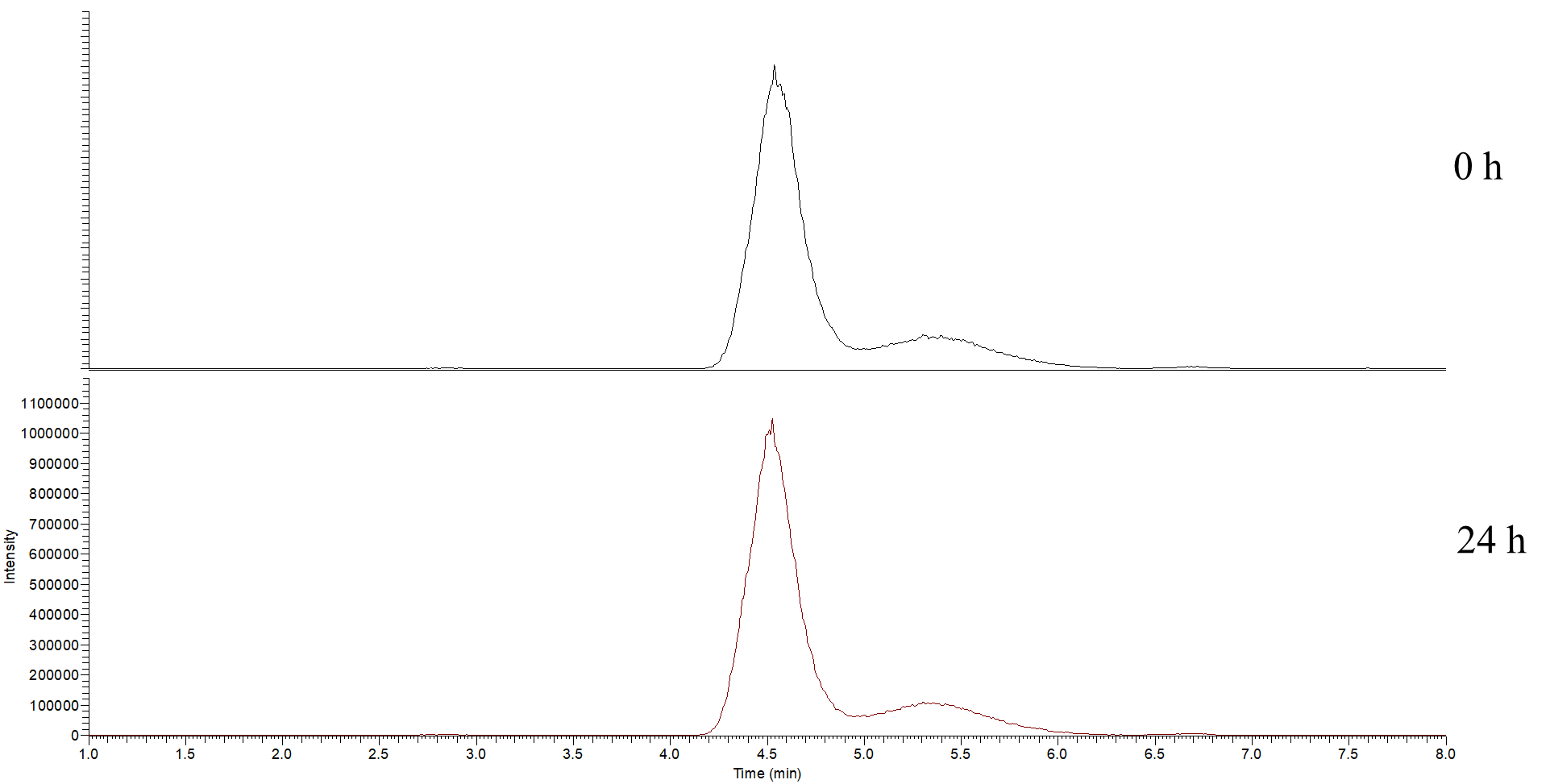


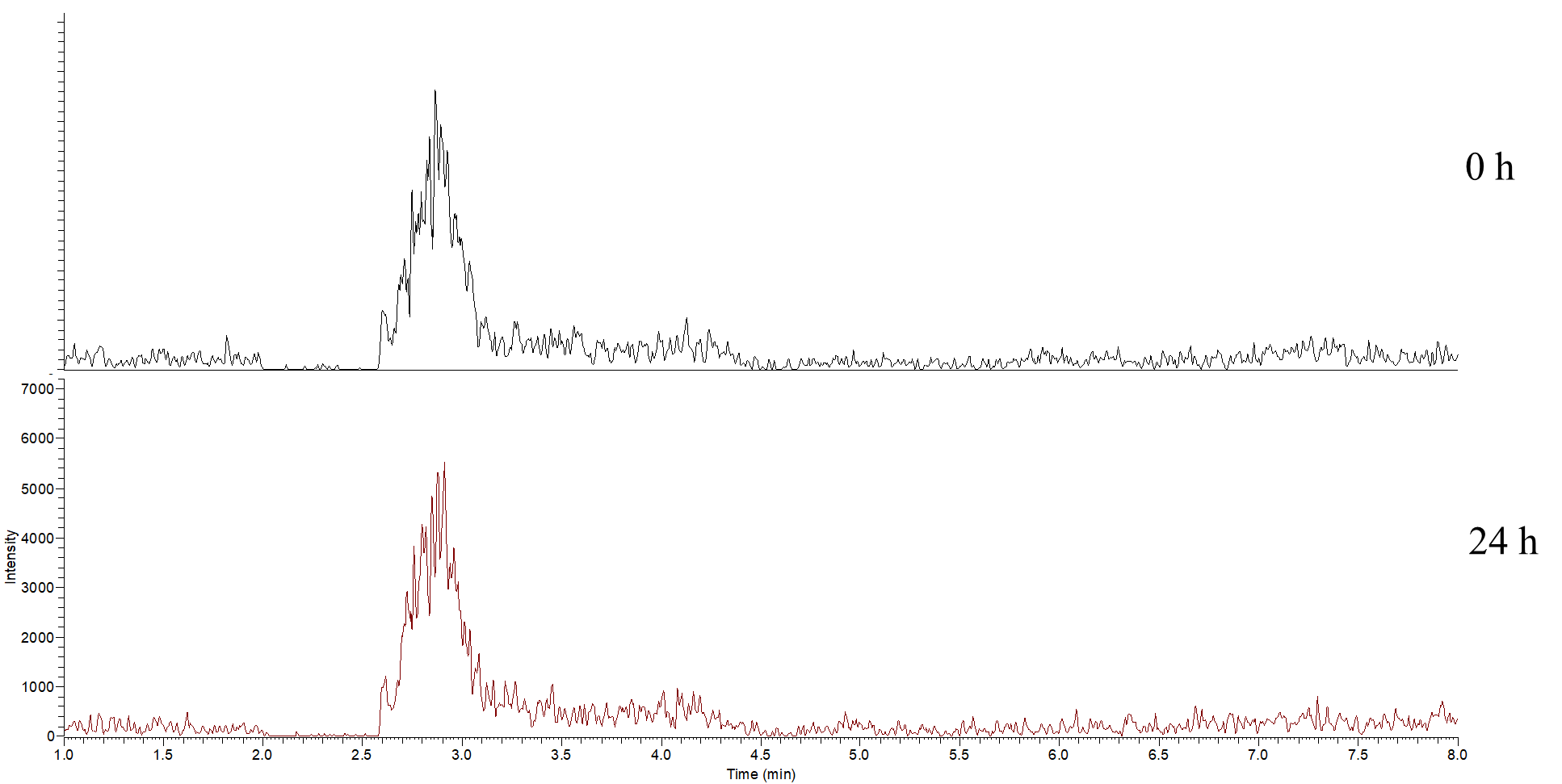


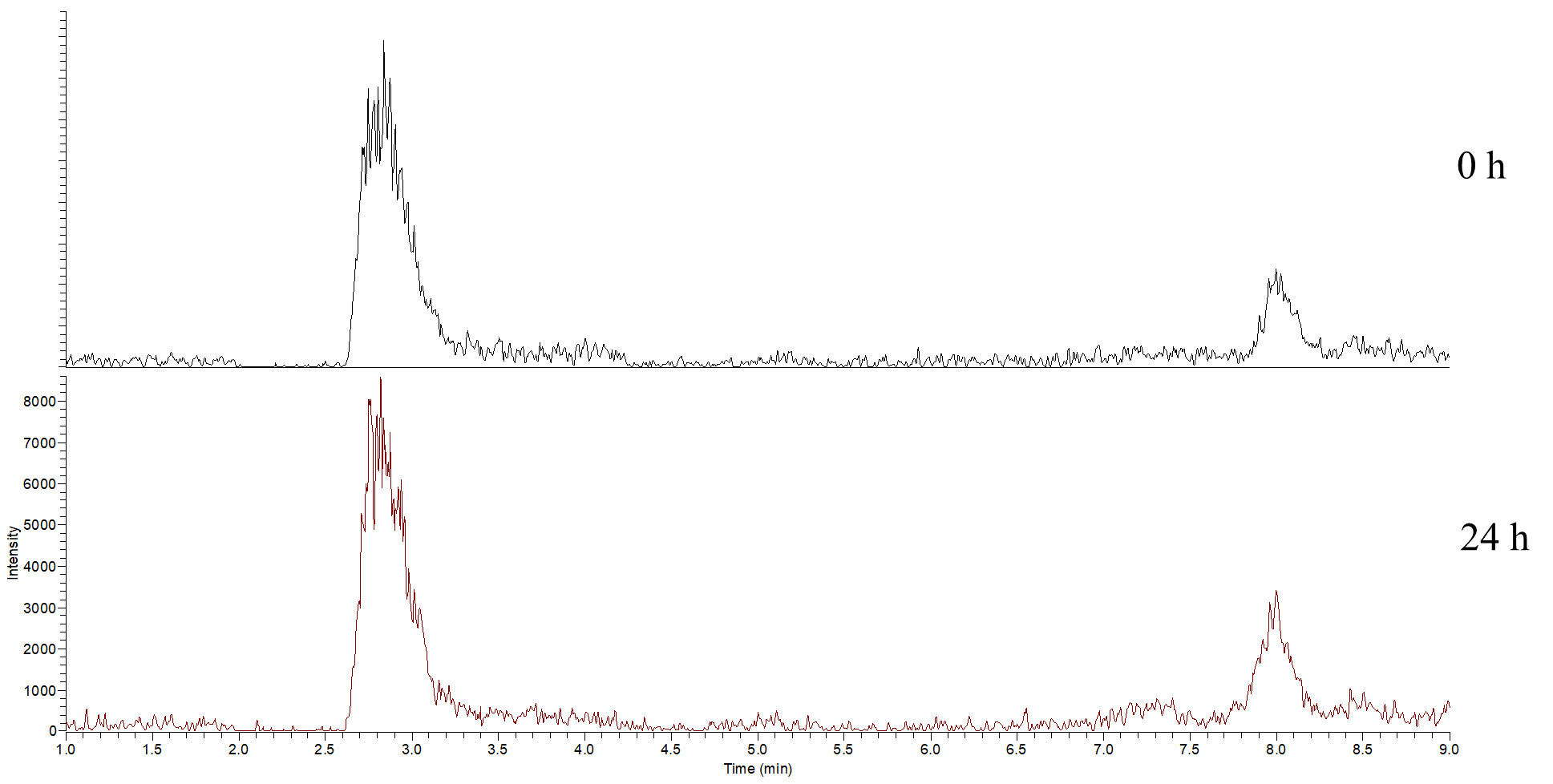


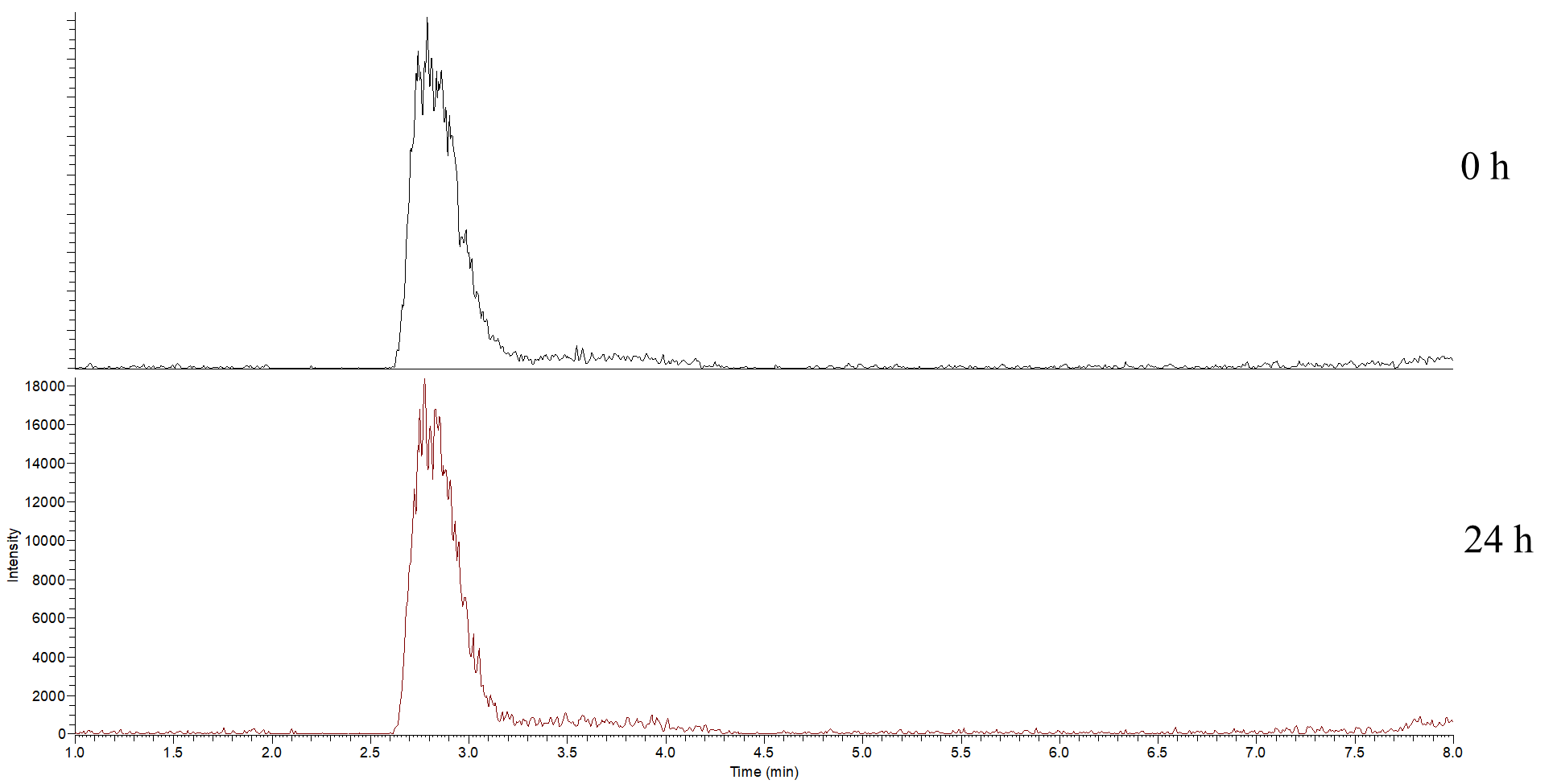


Figure S17. PGC-LC-MS elution patterns of selected *m/z* 608 (base peak), *m/z* 582 (base peak - mass of sulphate), *m/z* 462 (base peak - mass of Fuc), and *m/z* 382 (base peak - mass of sulphate and fucose) mass spectrum of 6’-*O*-sulphated Lewis a before (0 h) and after (24 h) incubation with *B. thetaiotaomicron* lysate.


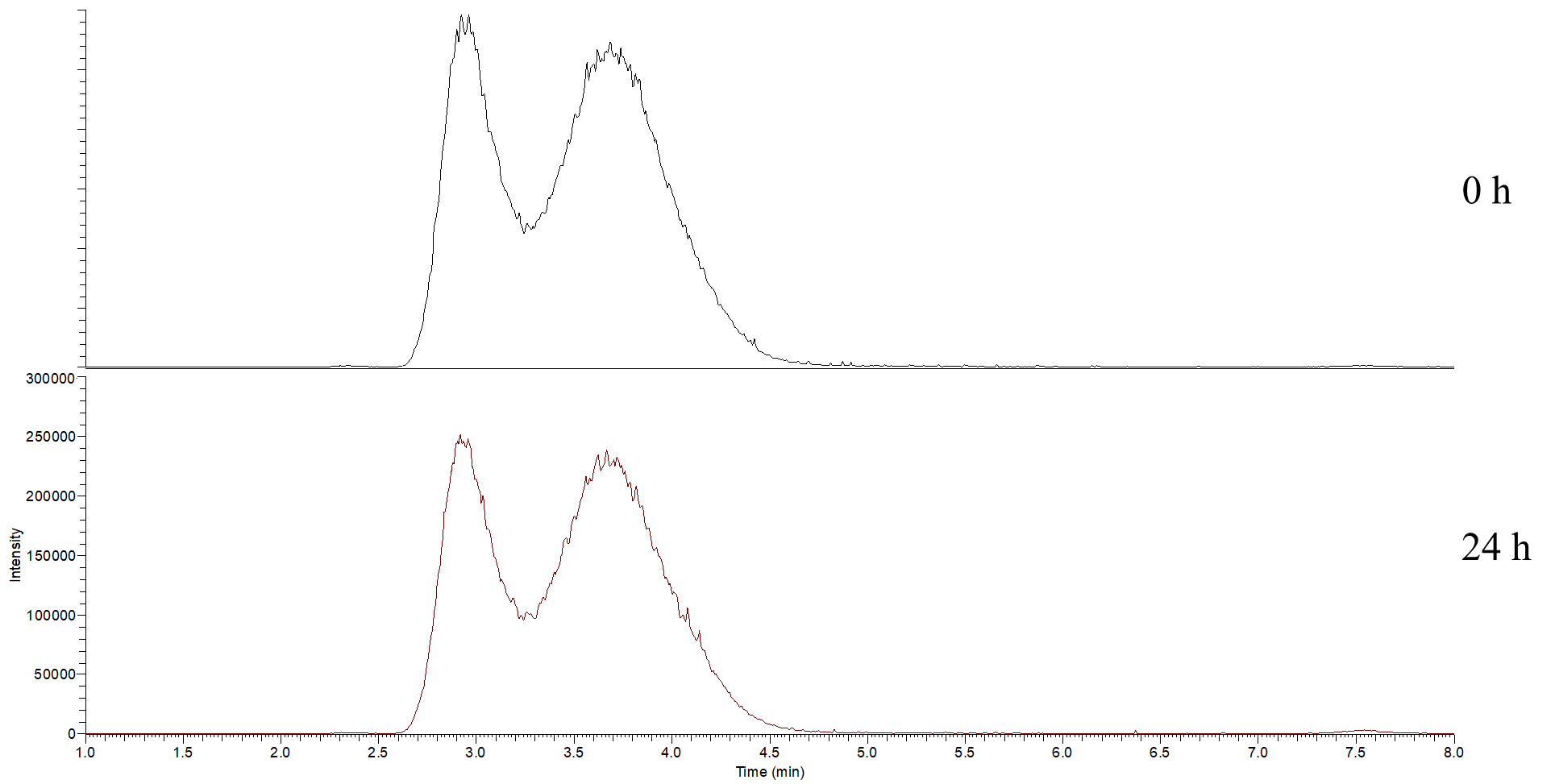


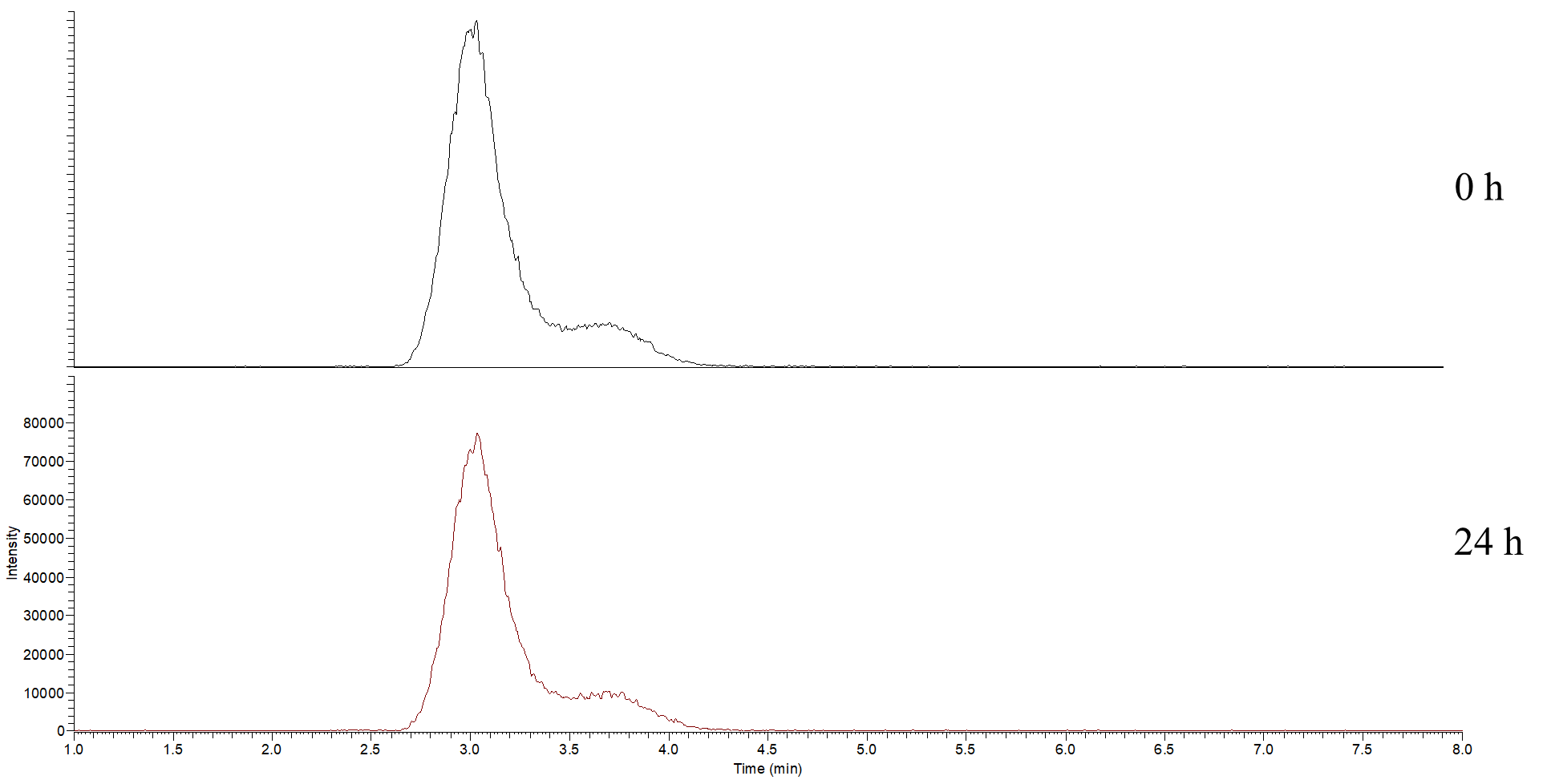


Figure S18. PGC-LC-MS elution patterns of selected m/z 300 (base peak) of 6S-GlcNAc before (0 h) and after (24 h) incubation with the mucin-degrading synthetic community (MDSC) lysate. Top and bottom chromatograms are replicate incubations.


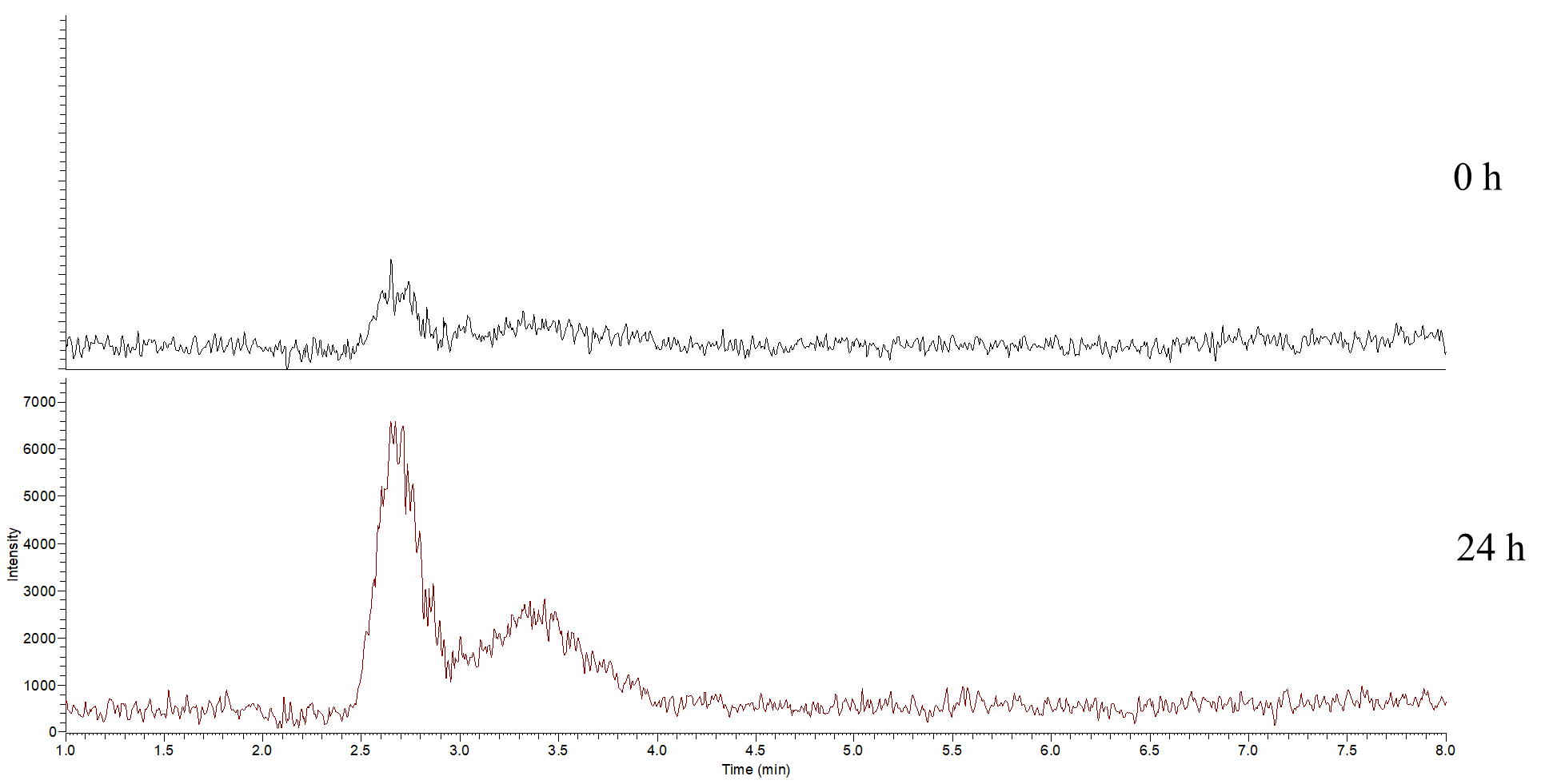


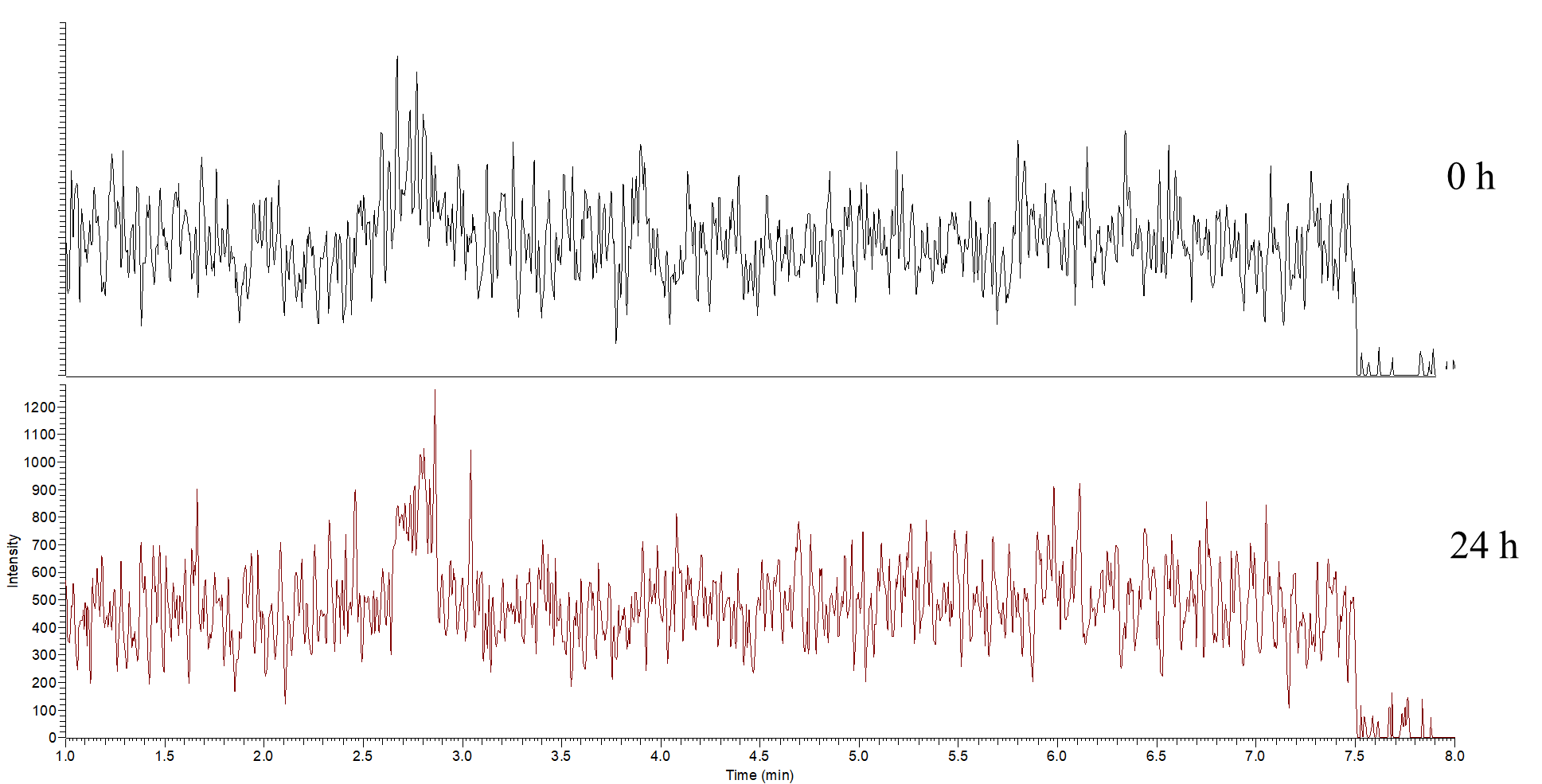


Figure S19. PGC-LC-MS elution patterns of selected *m/z* 220 (base peak – mass of sulphate) of 6S-GlcNAc before (0 h) and after (24 h) incubation with the mucin-degrading synthetic community (MDSC) lysate. Top and bottom chromatograms are replicate incubations.


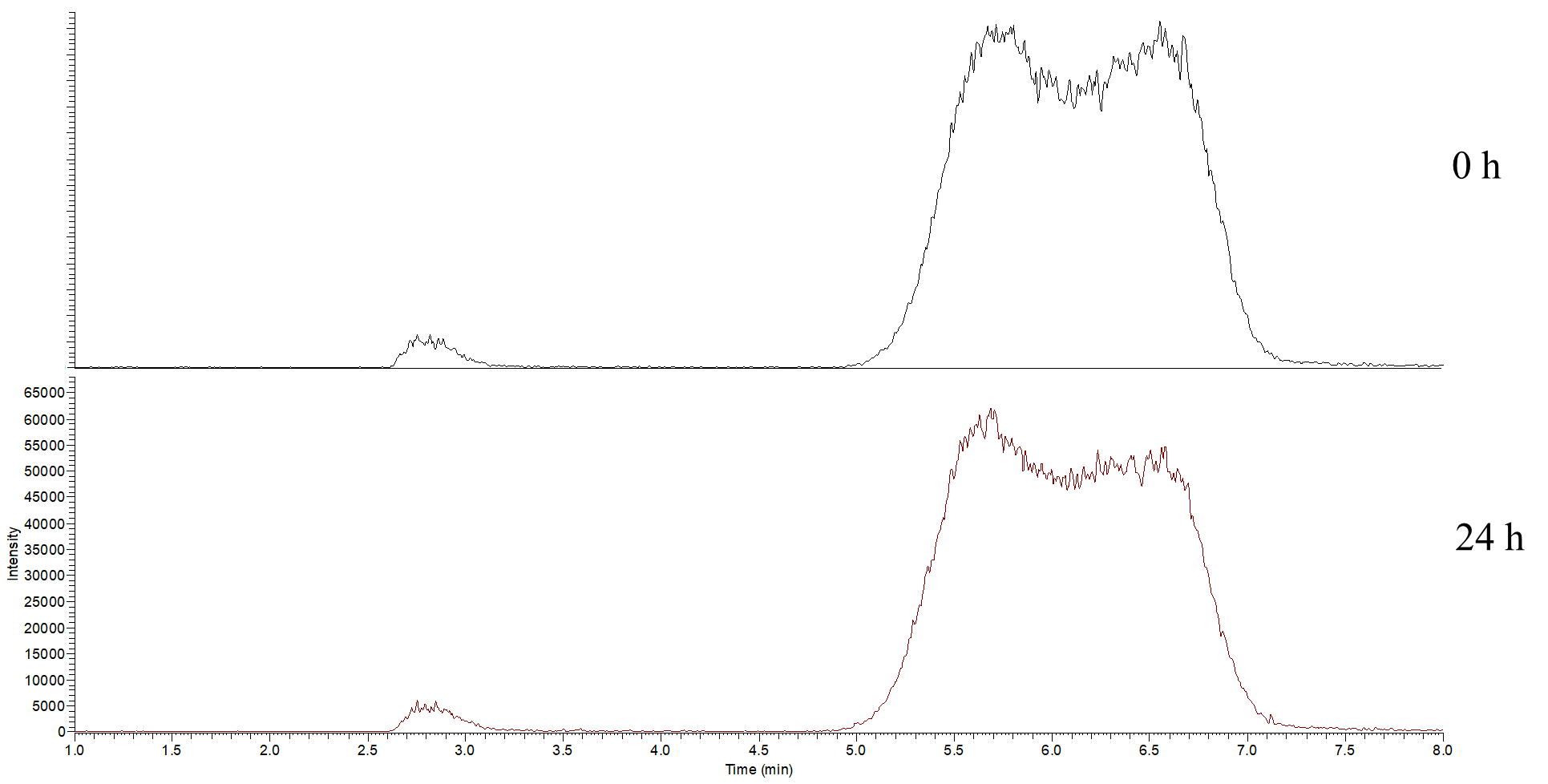


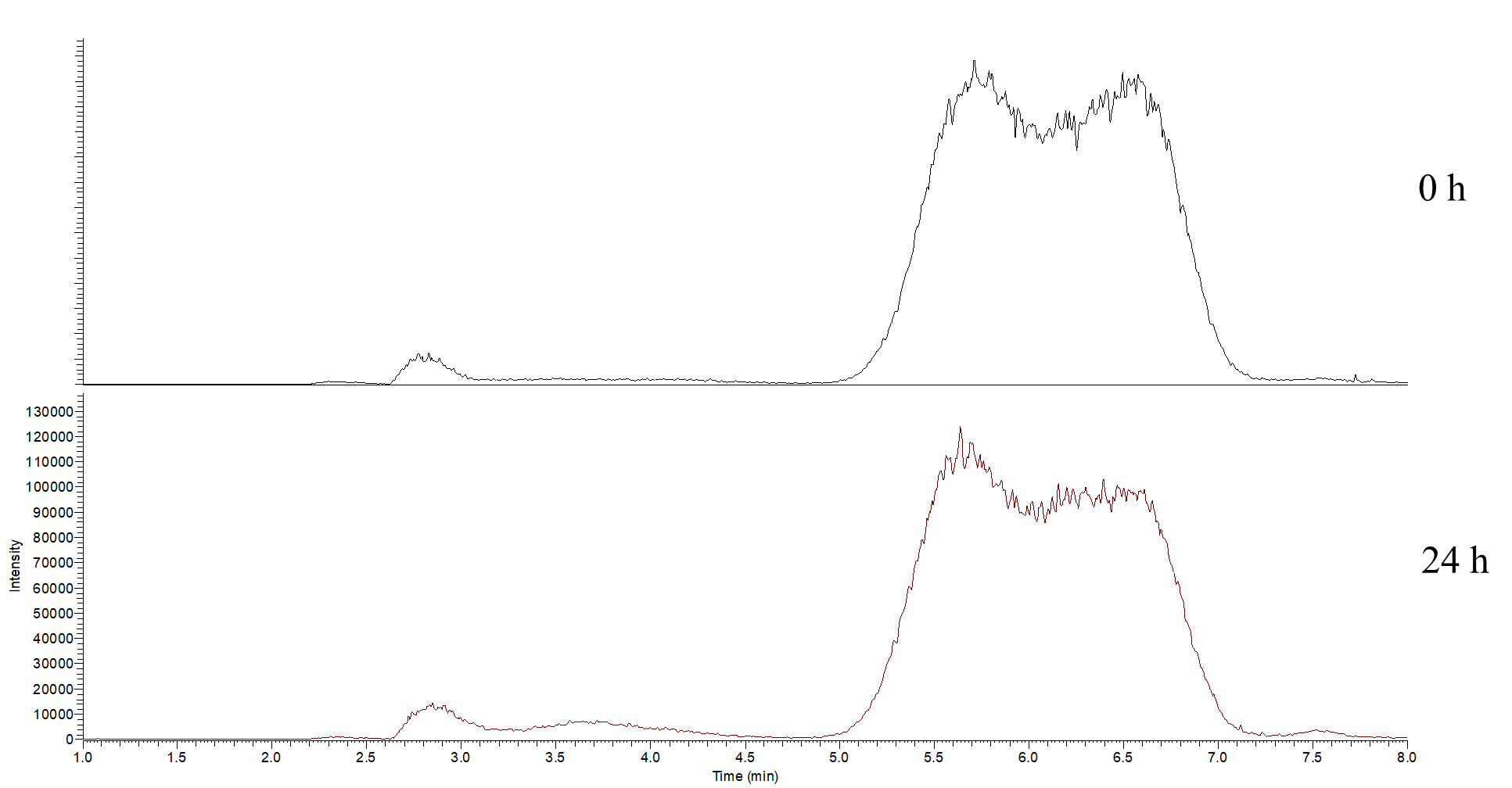


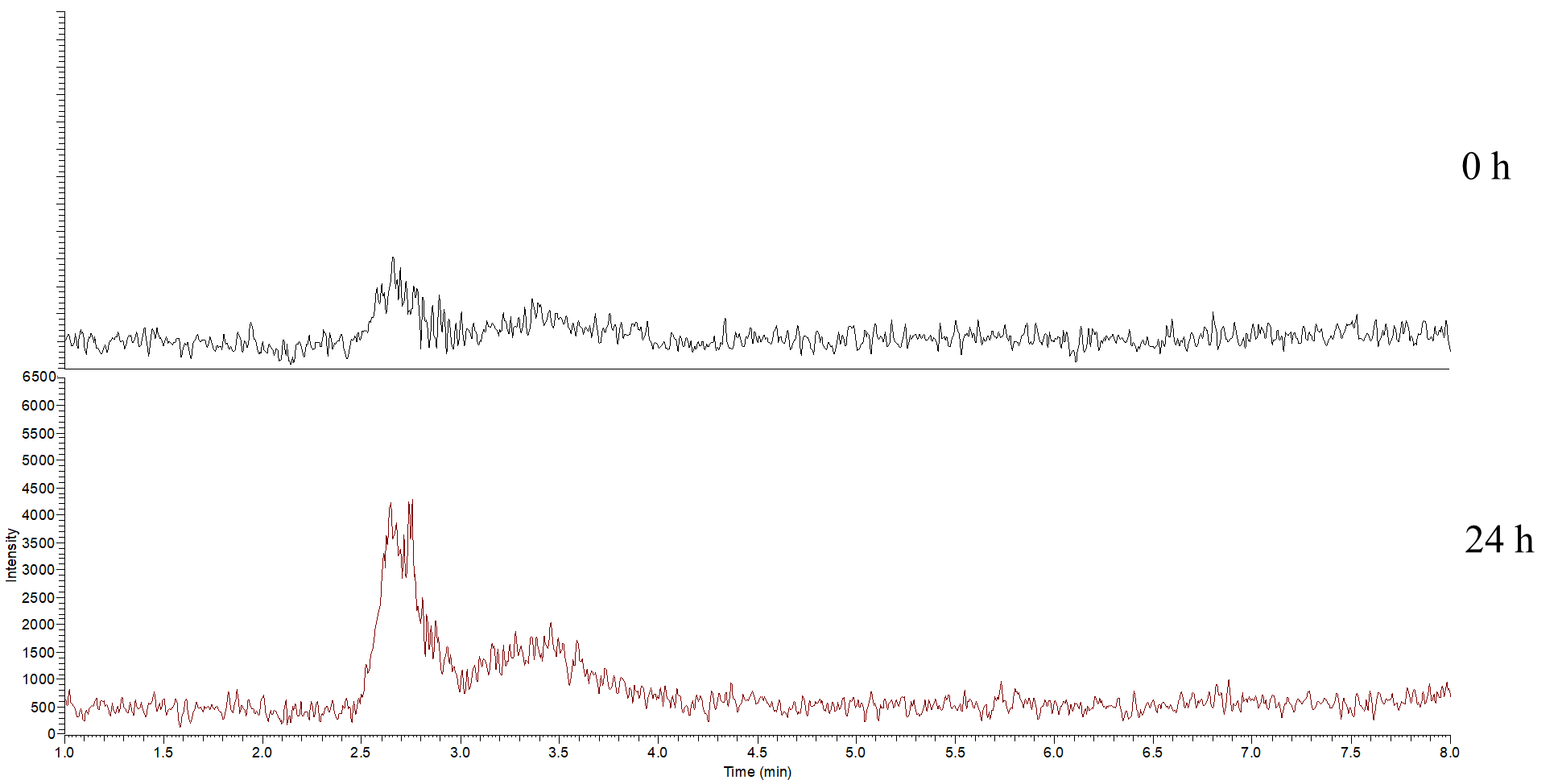


Figure S20. PGC-LC-MS elution patterns of selected *m/z* 380 (base peak), *m/z* 300 (base peak - mass of sulphate), and *m/z* 200 (base peak - 2x mass of sulphate) of 3S6S-GlcNAc before (0 h) and after (24 h) incubation with the mucin-degrading synthetic community (MDSC) lysate.


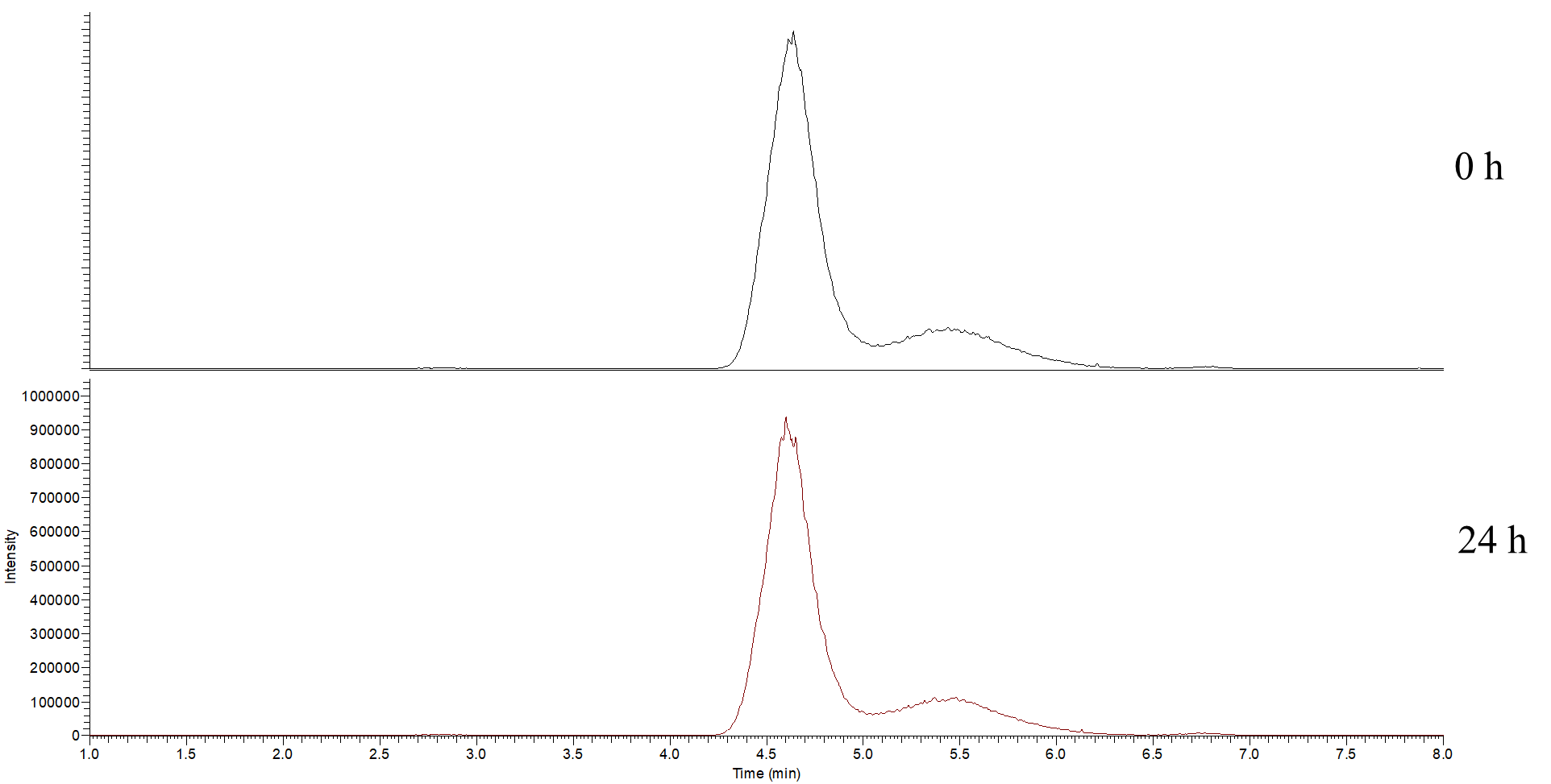


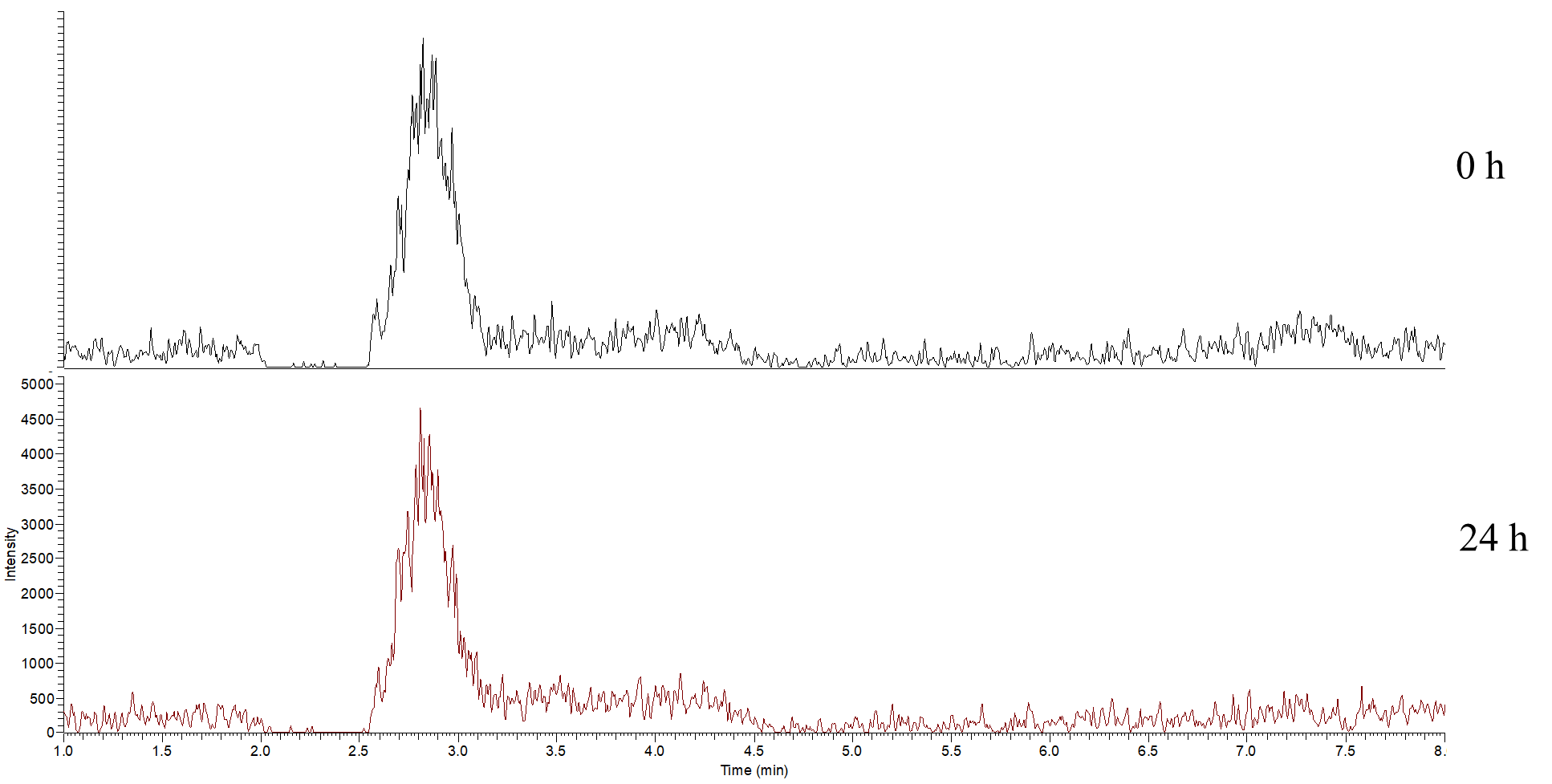


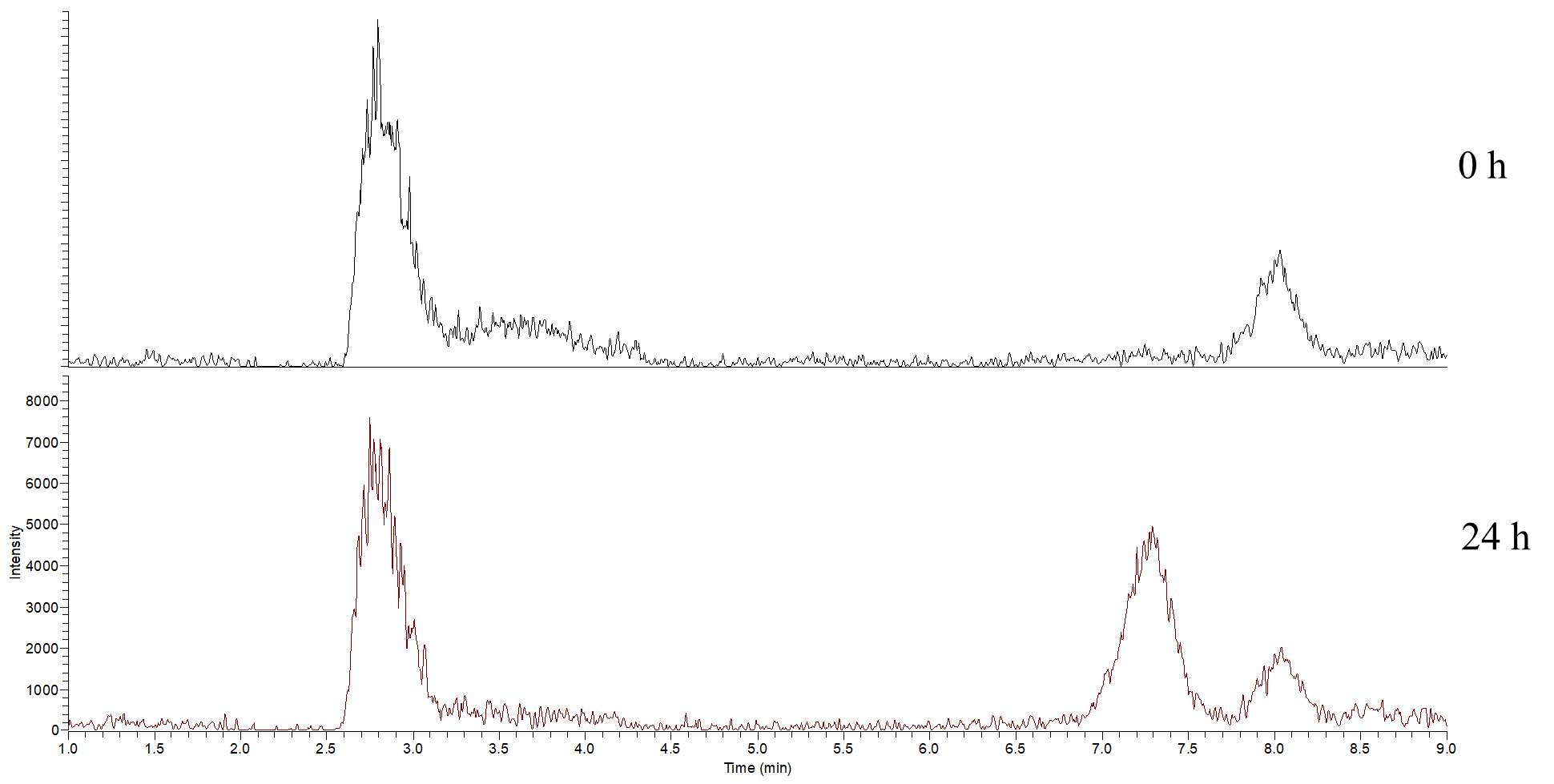


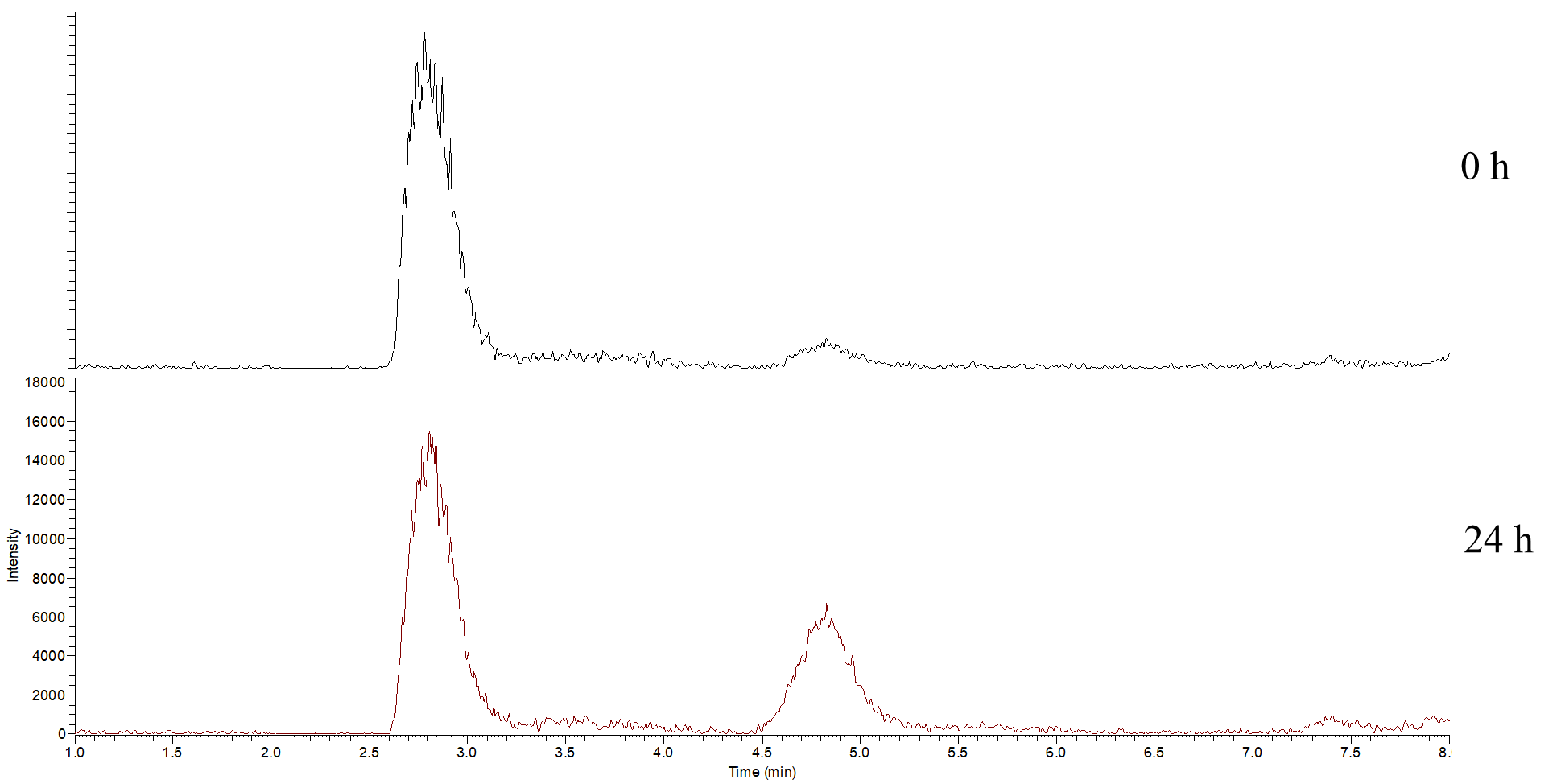


Figure S21. PGC-LC-MS elution patterns of selected *m/z* 608 (base peak), *m/z* 582 (base peak - mass of sulphate), *m/z* 462 (base peak - mass of Fuc), and *m/z* 382 (base peak - mass of sulphate and fucose) of 6’-*O*-sulphated Lewis a before (0 h) and after (24 h) incubation with the mucin-degrading synthetic community (MDSC) lysate.


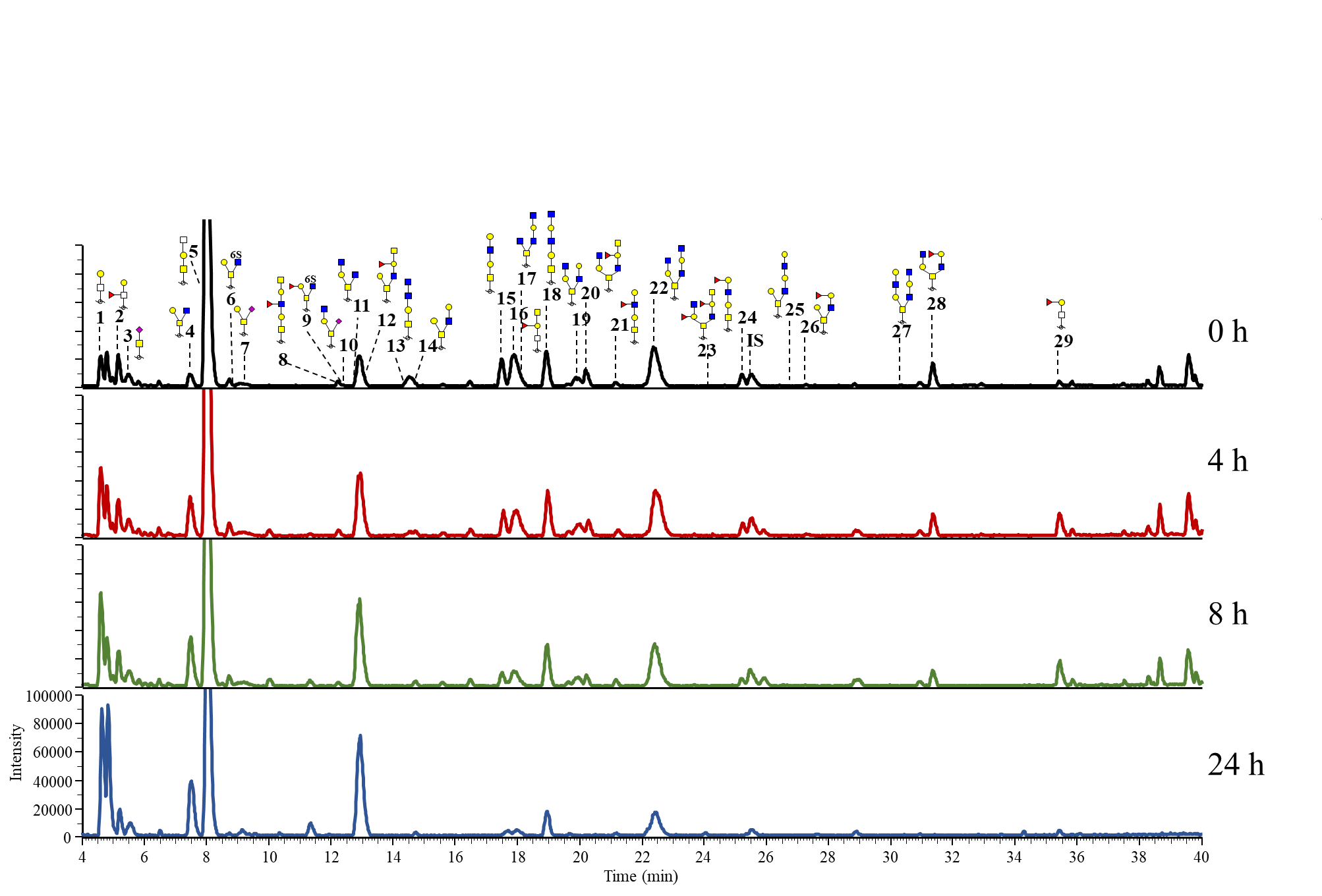


Figure S22. PGC-LC-MS elution patterns showing the degradation of *O*-glycans released from PGM after incubation with *A. muciniphila* lysate during 24 h. Identified peaks (0 h) are indicated with a number and structure which correspond to the fragmentation MS/MS data as shown in Figure S23.


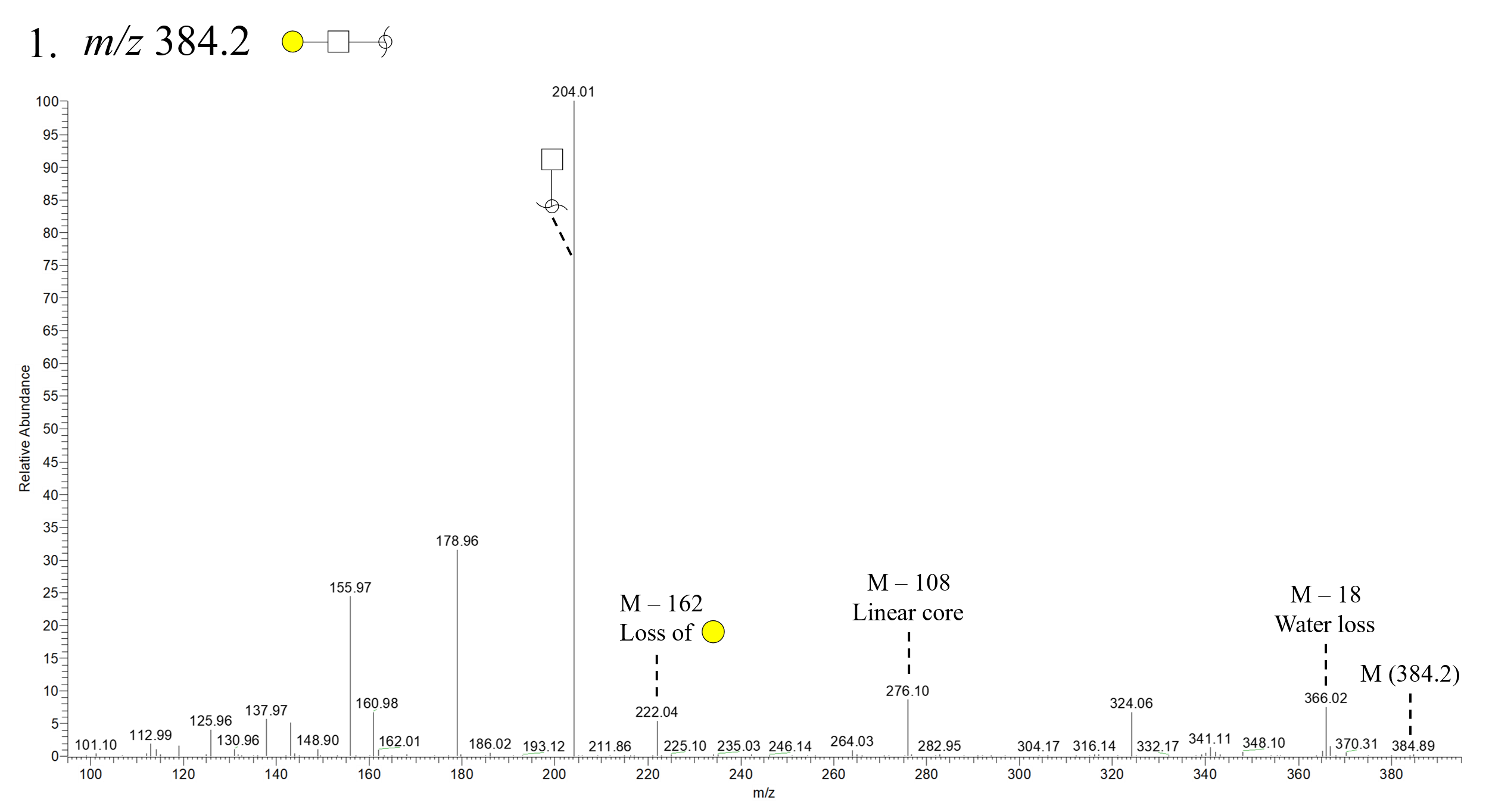


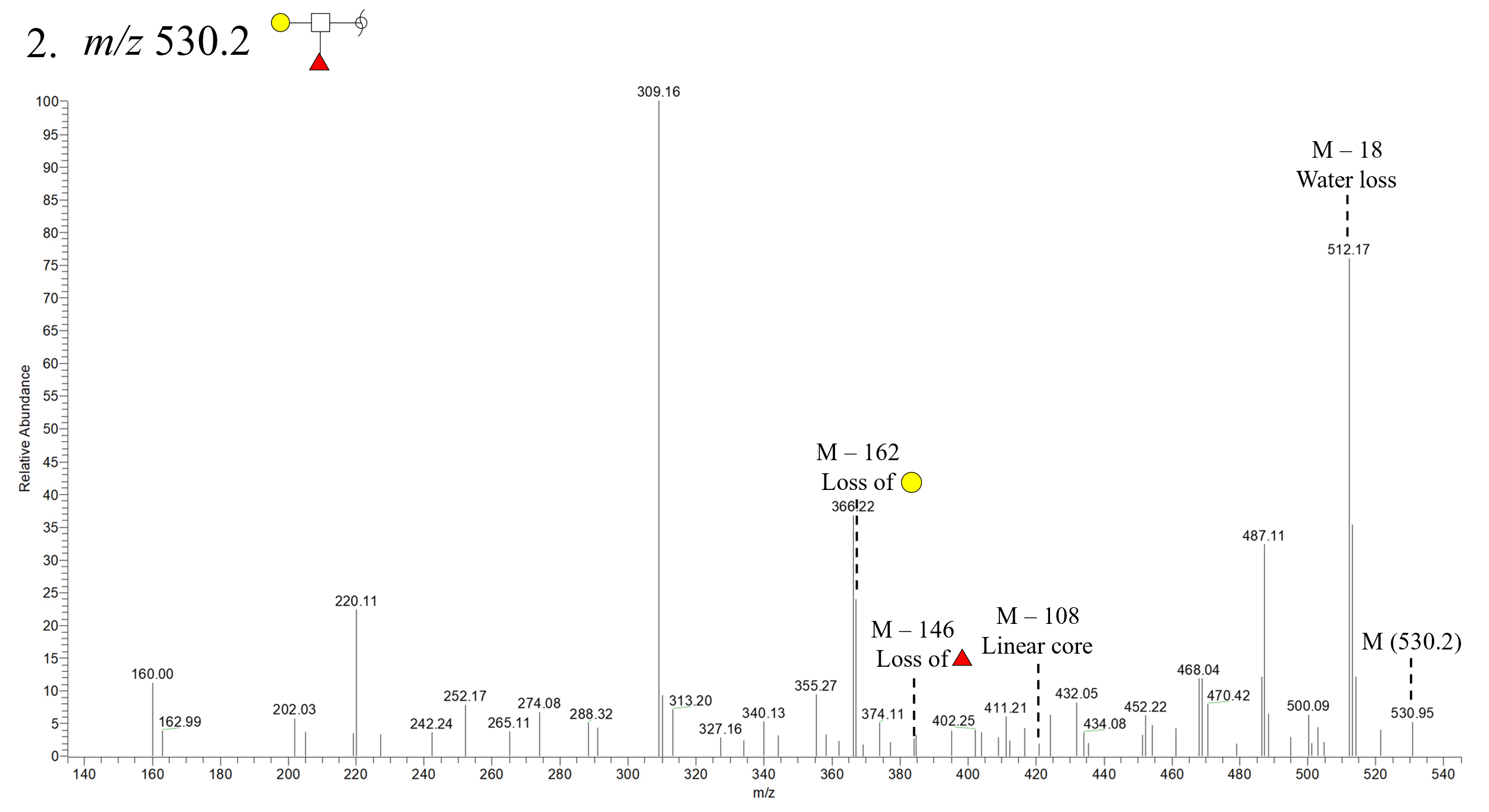


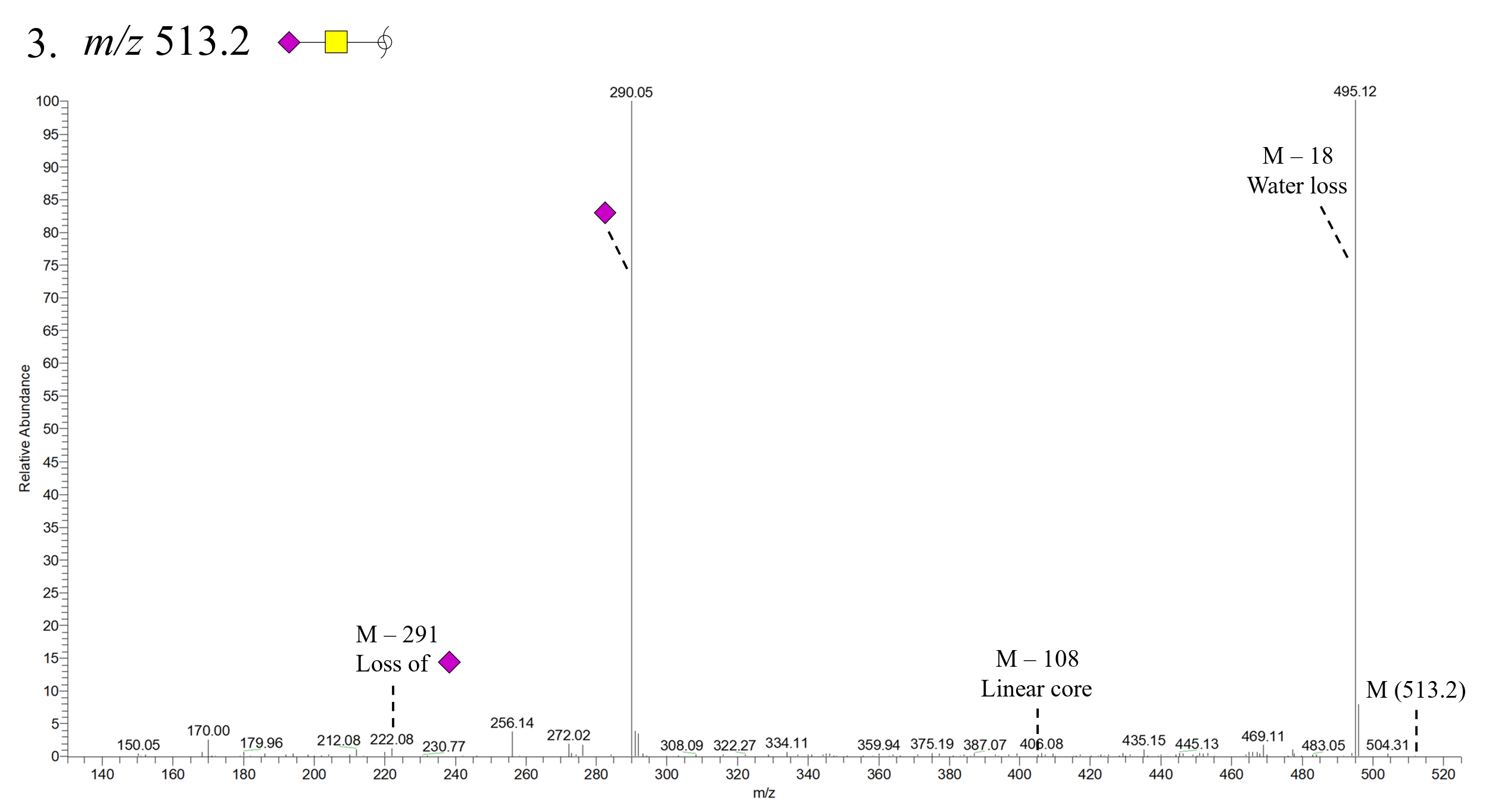


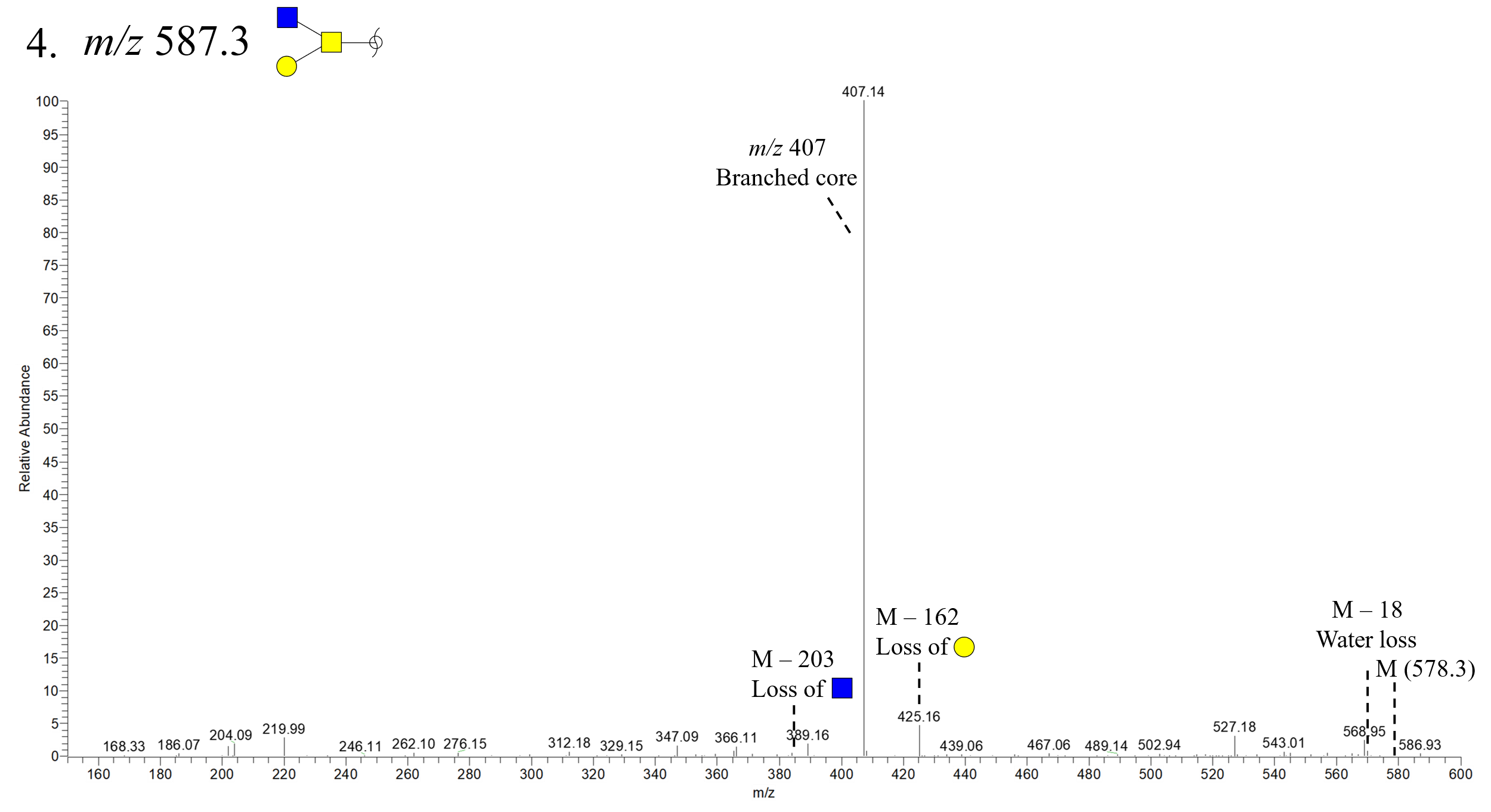


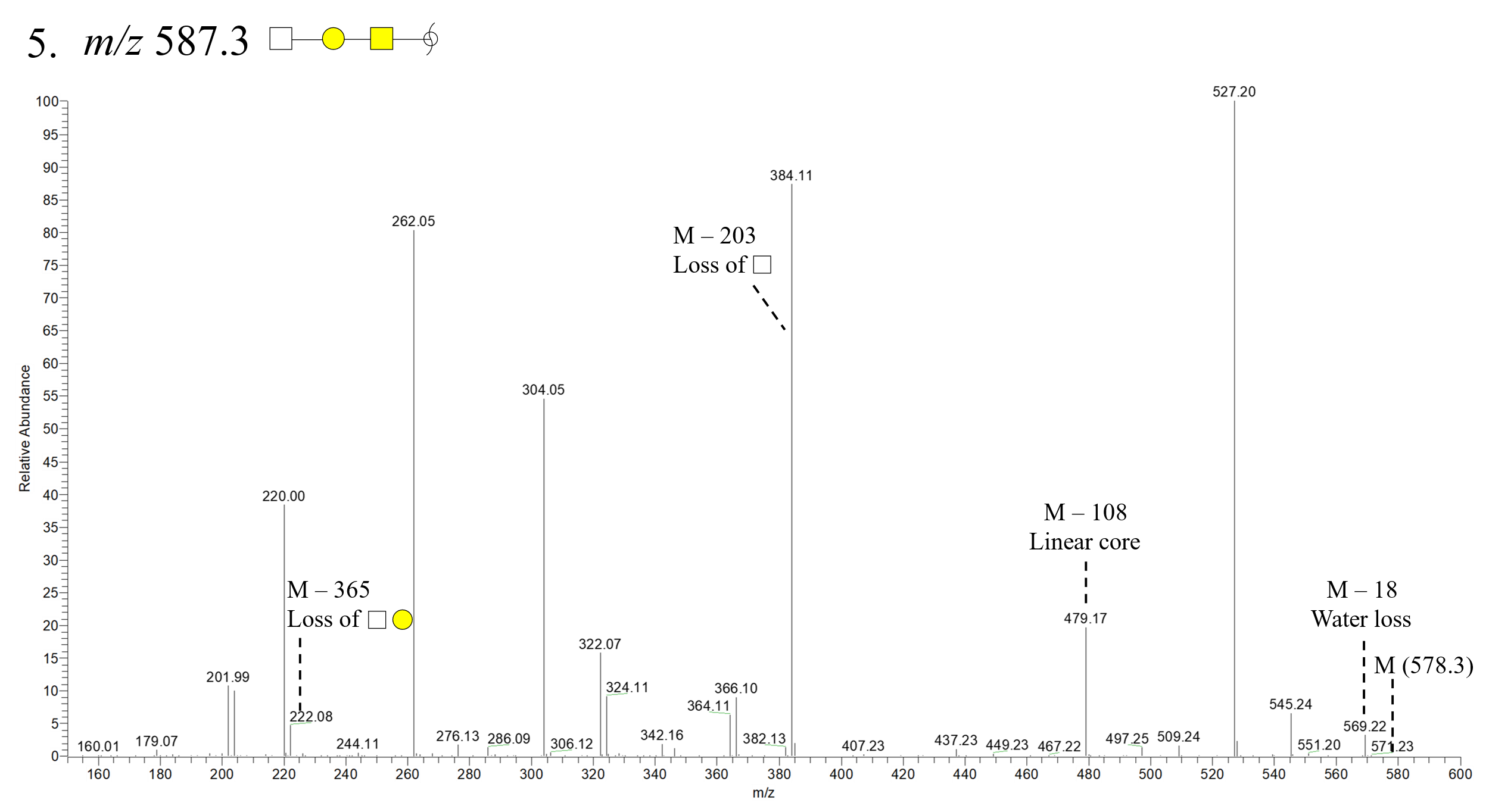


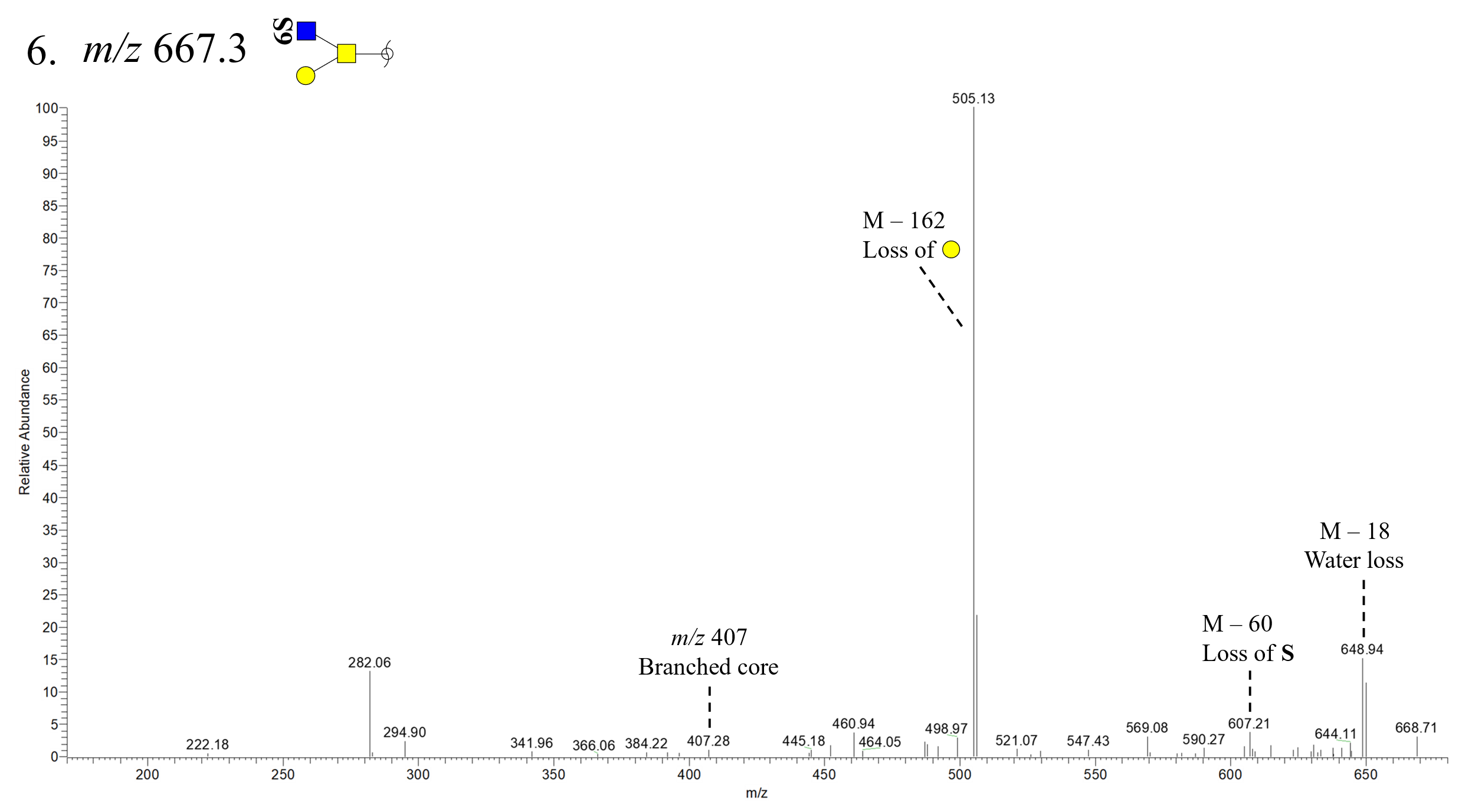


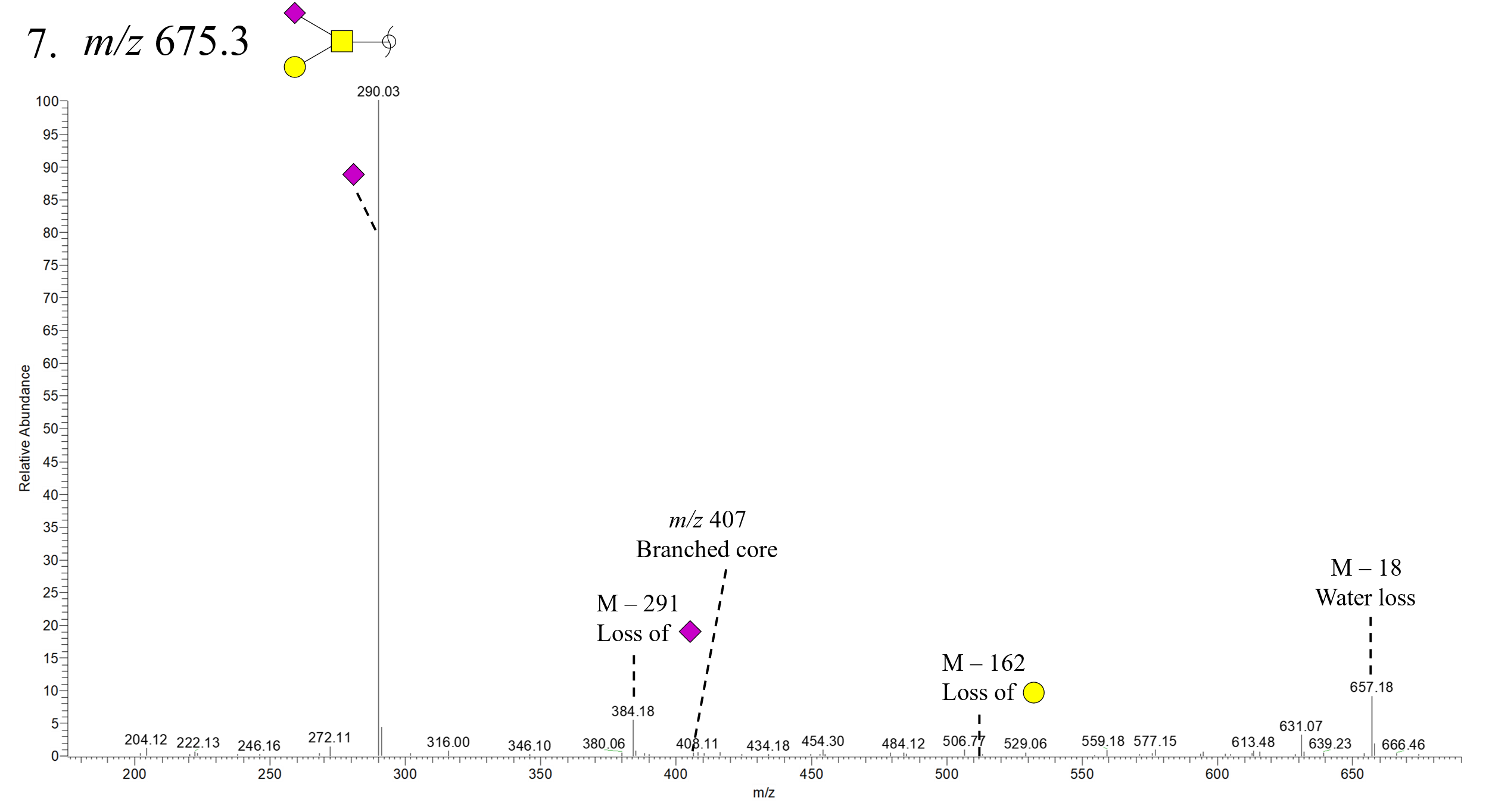


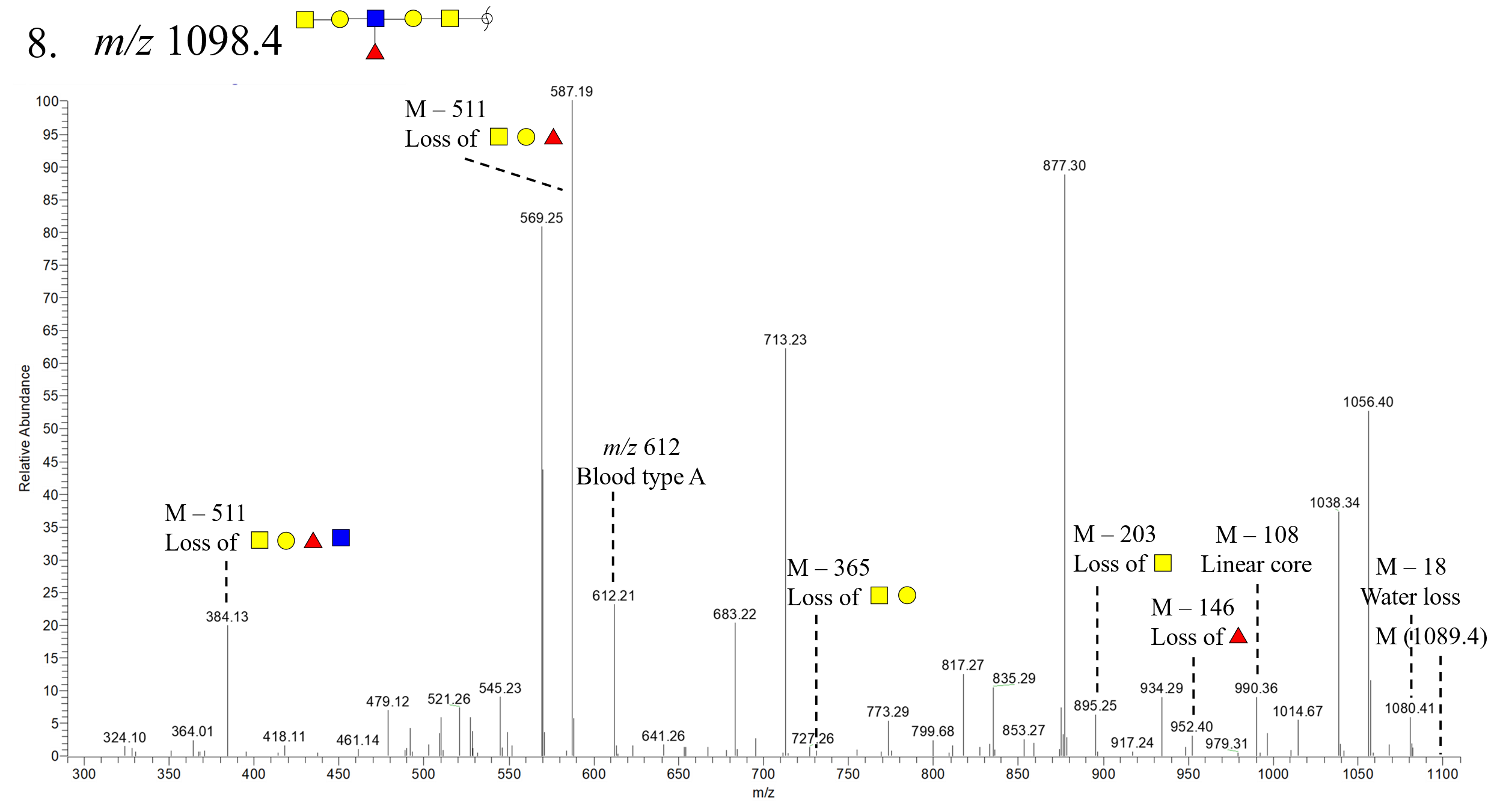


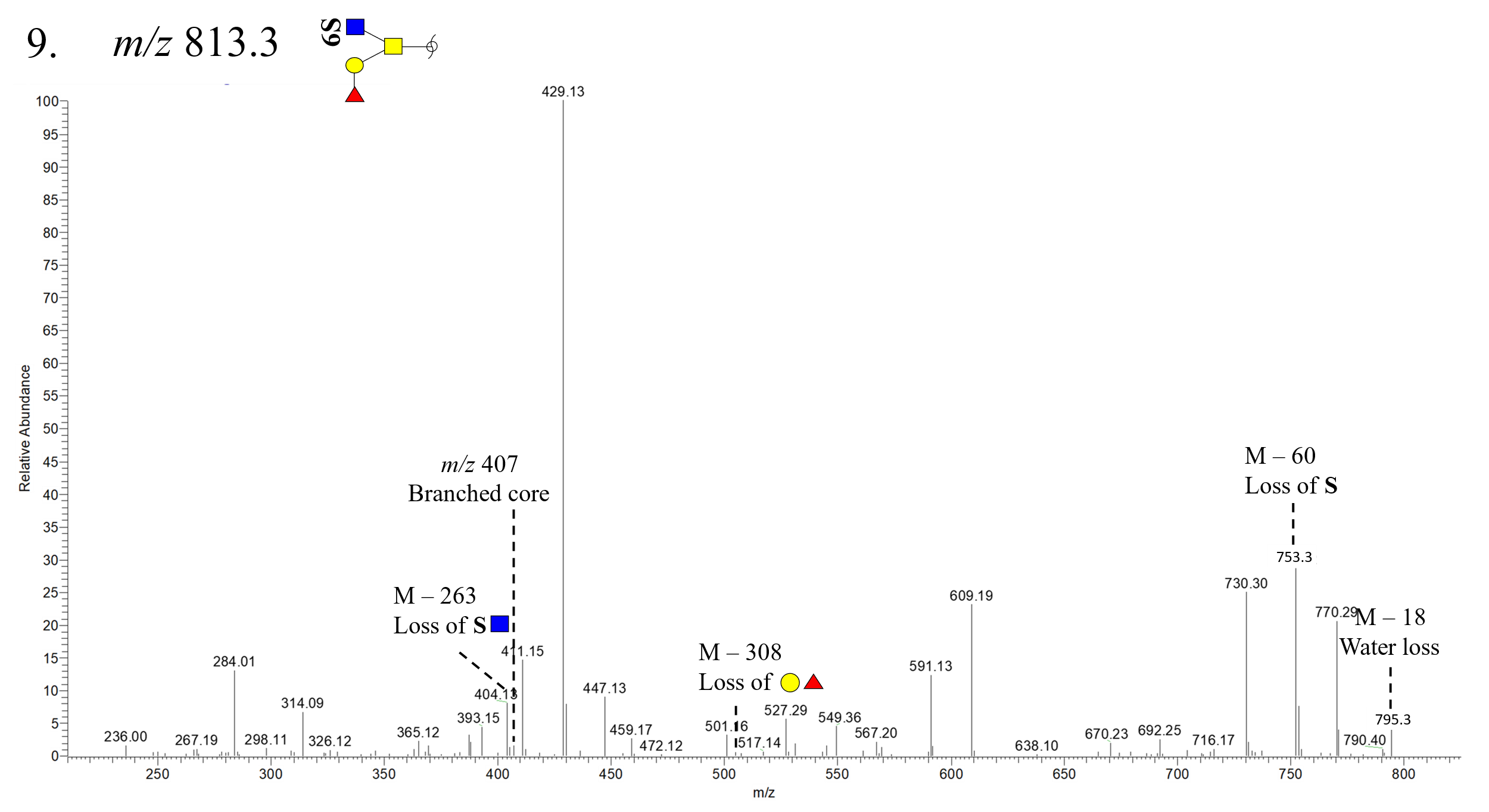


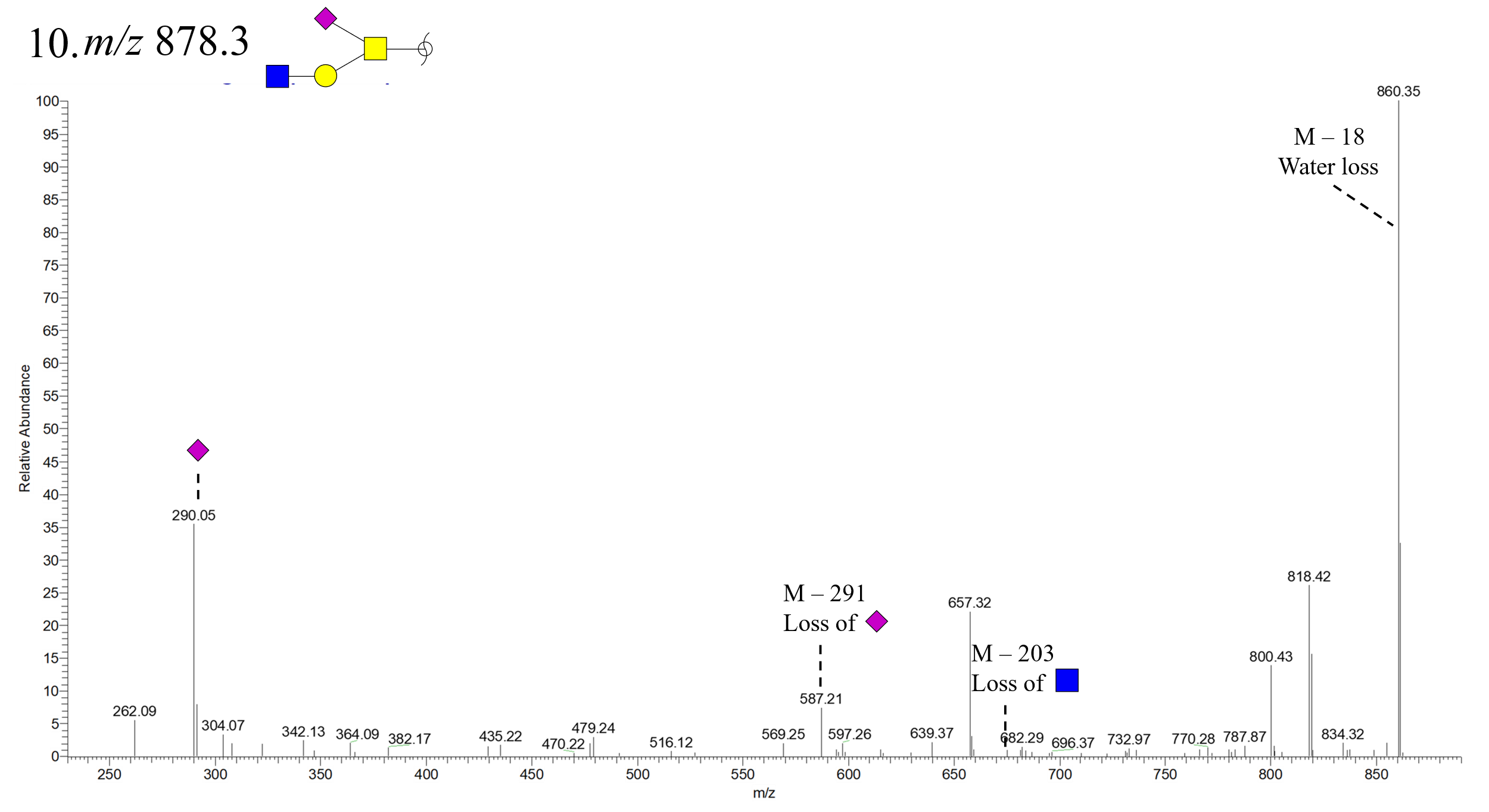


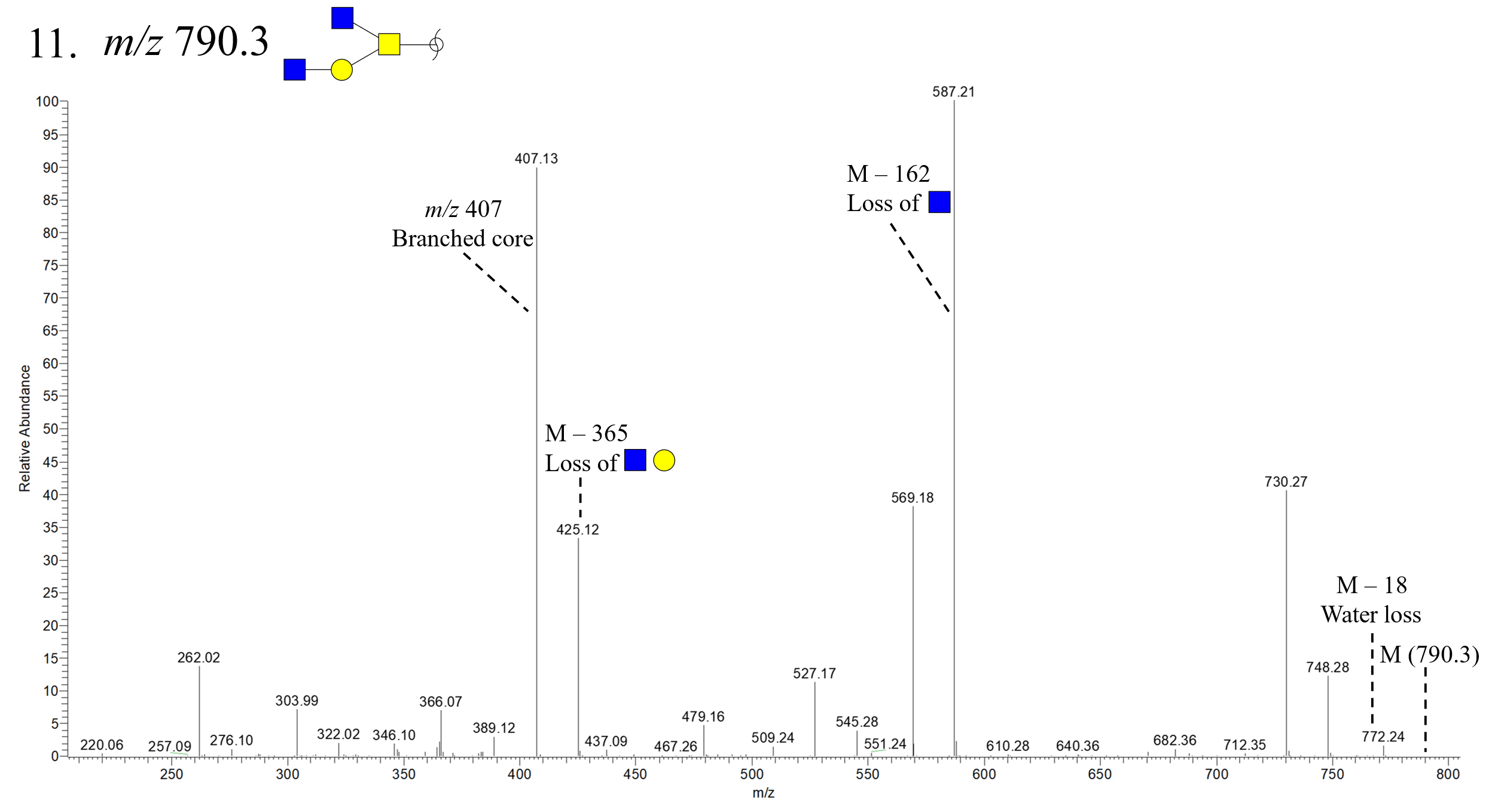


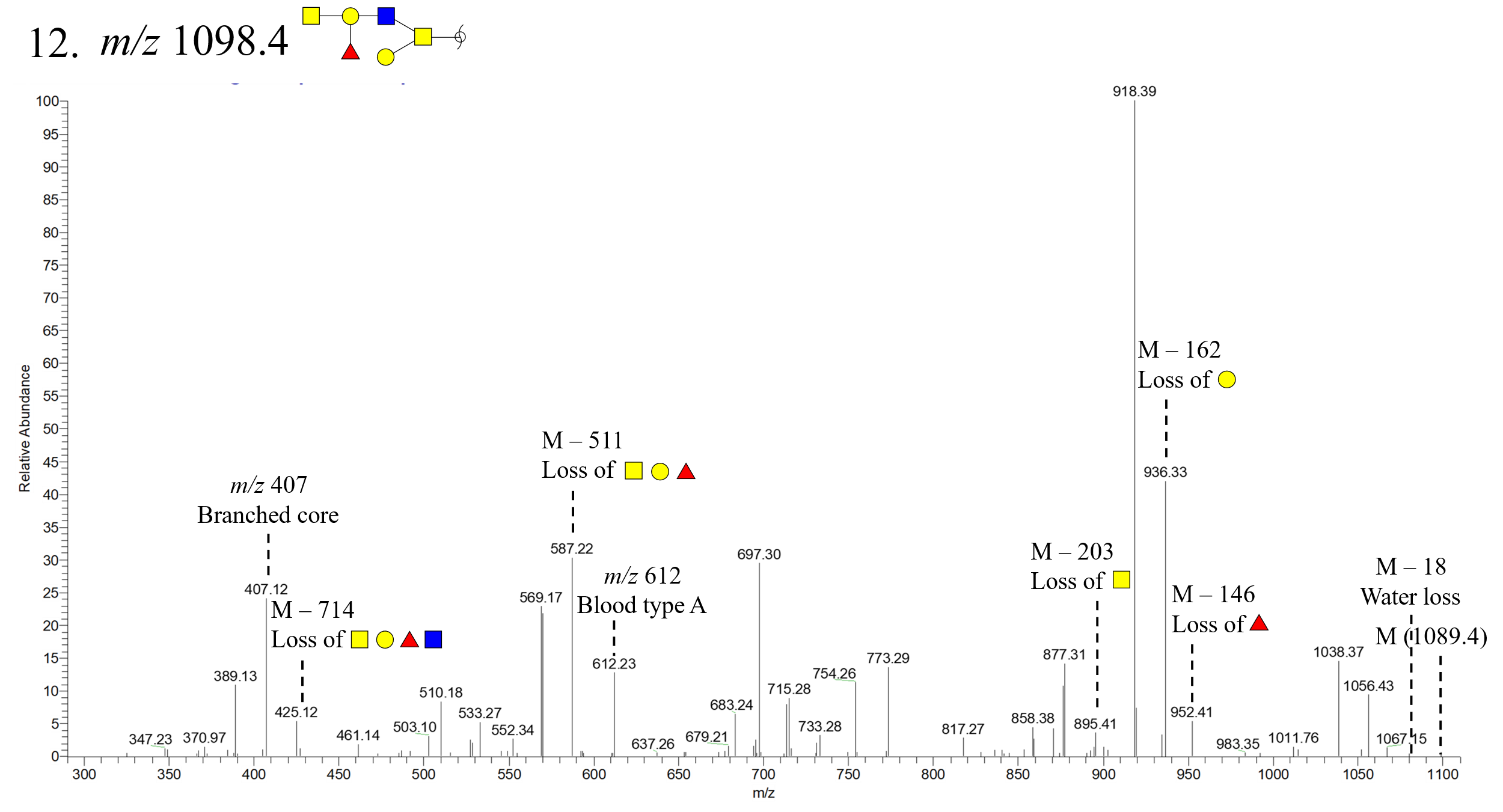


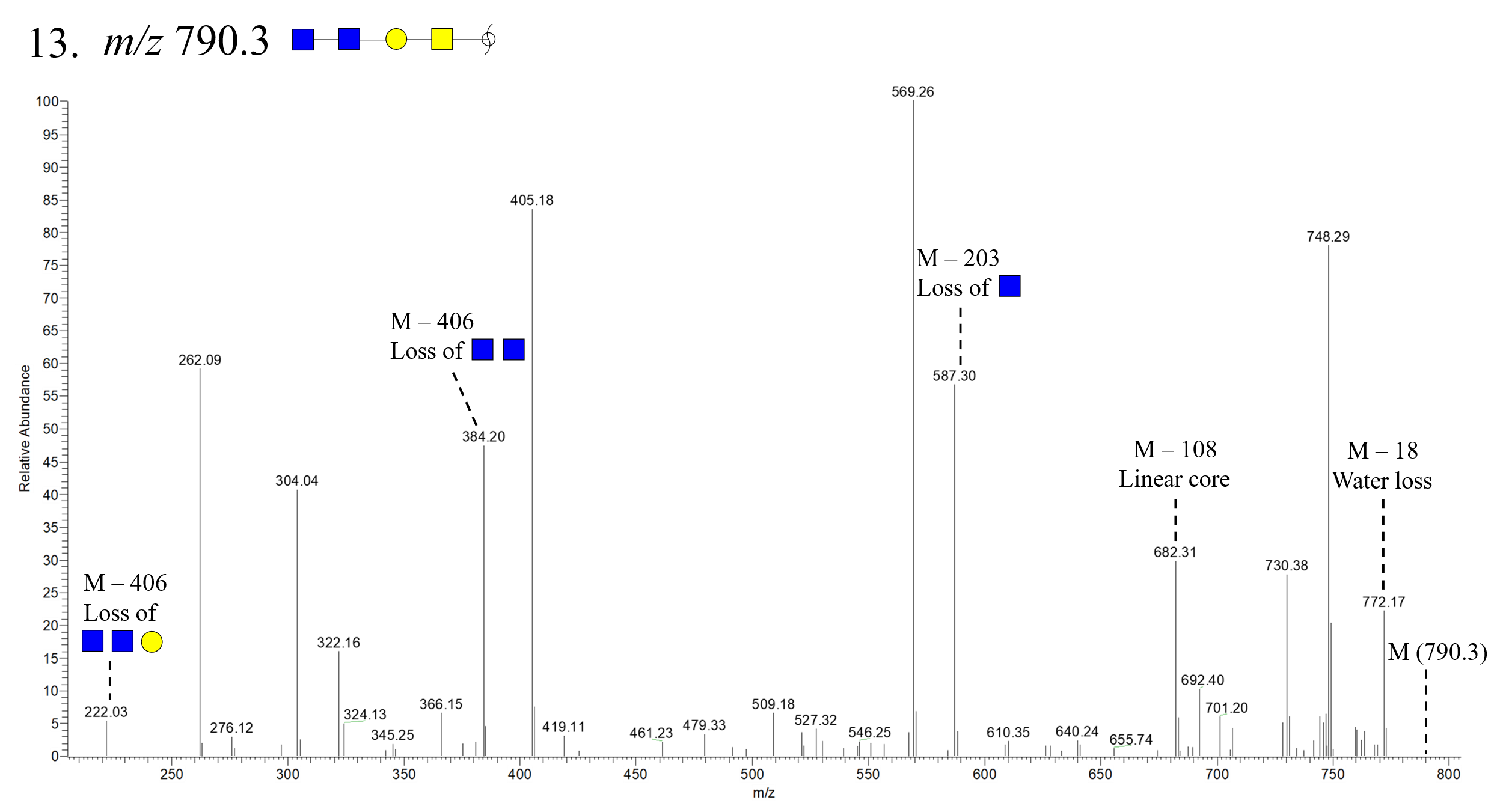


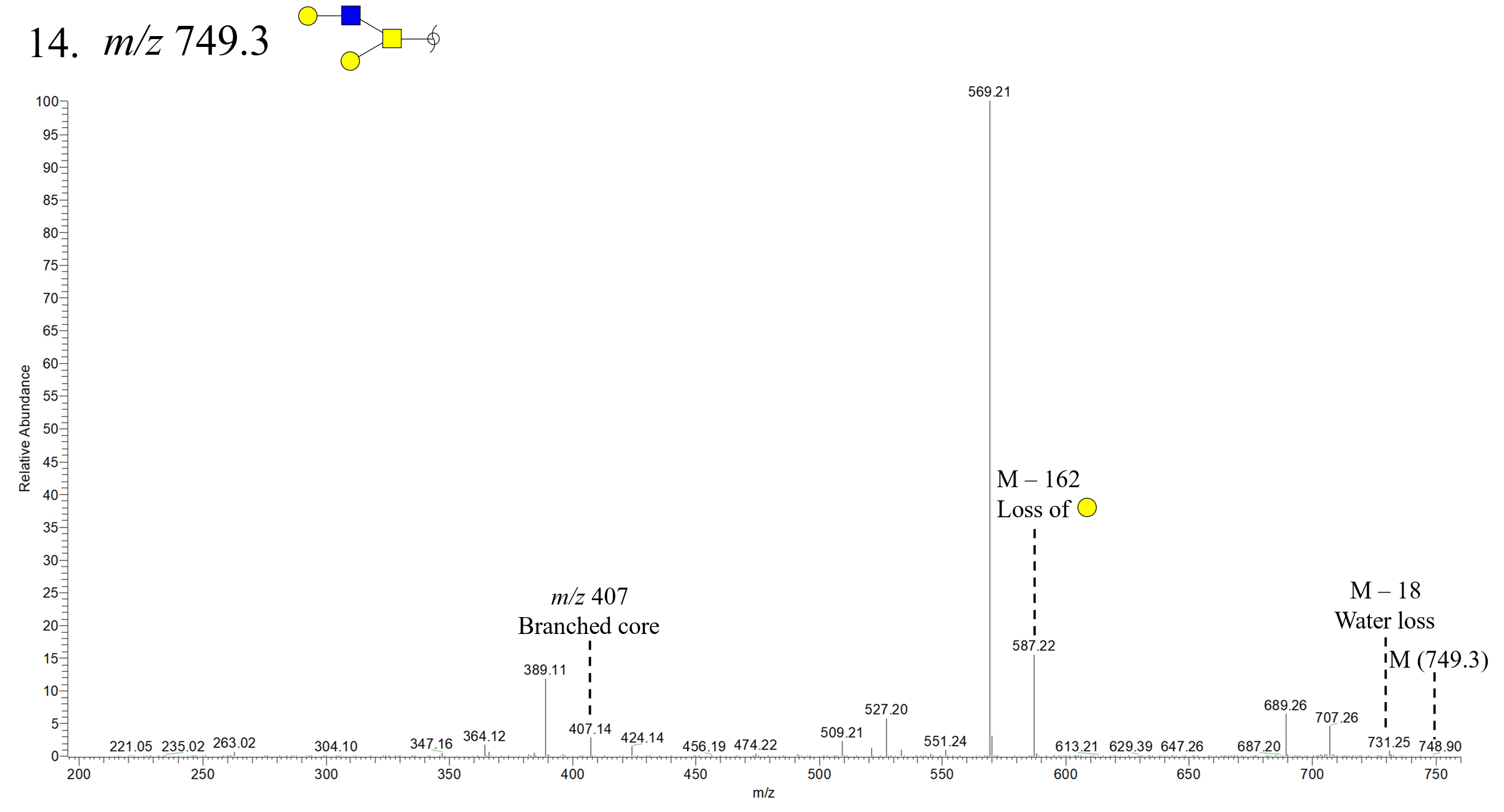


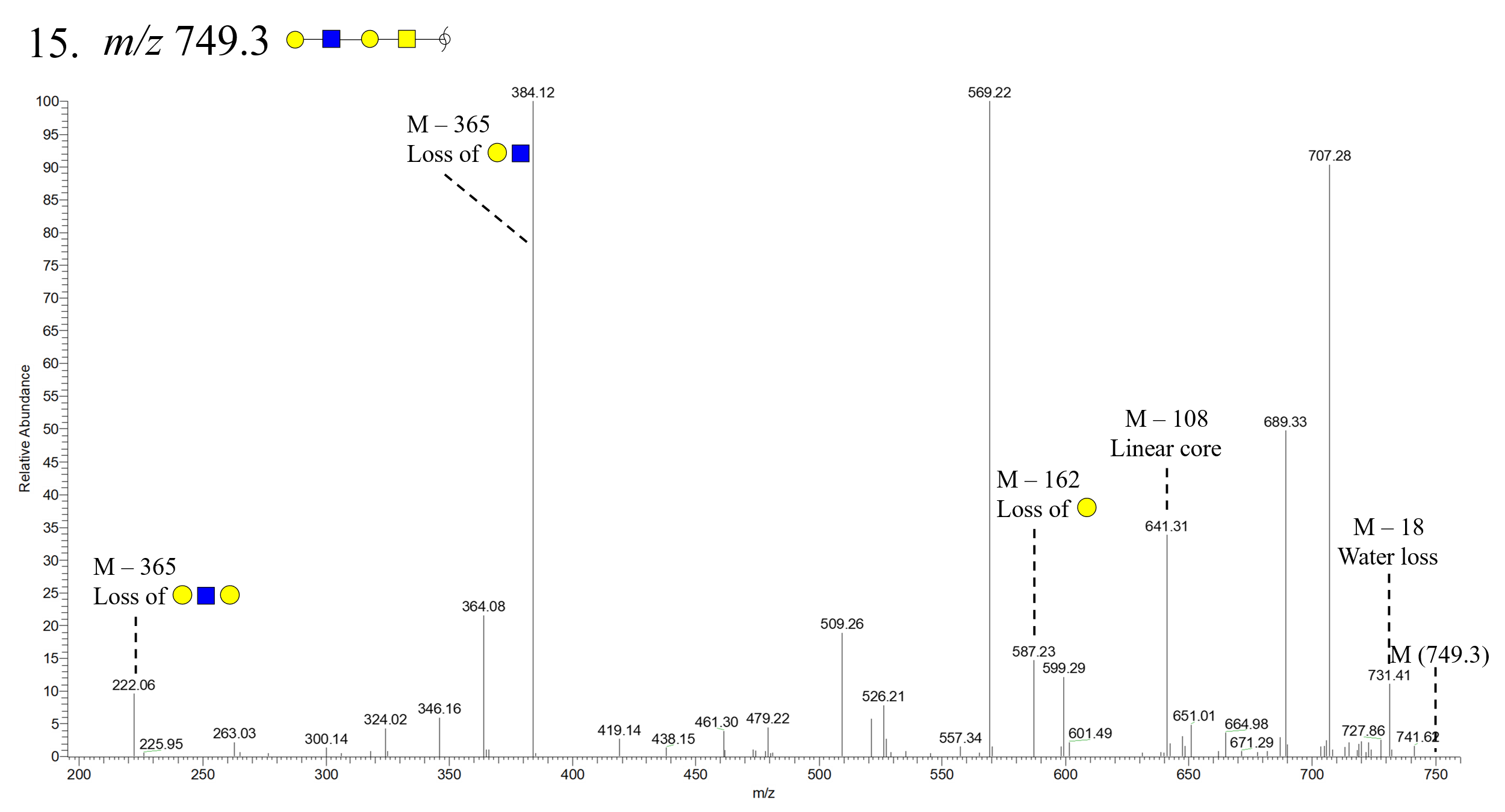


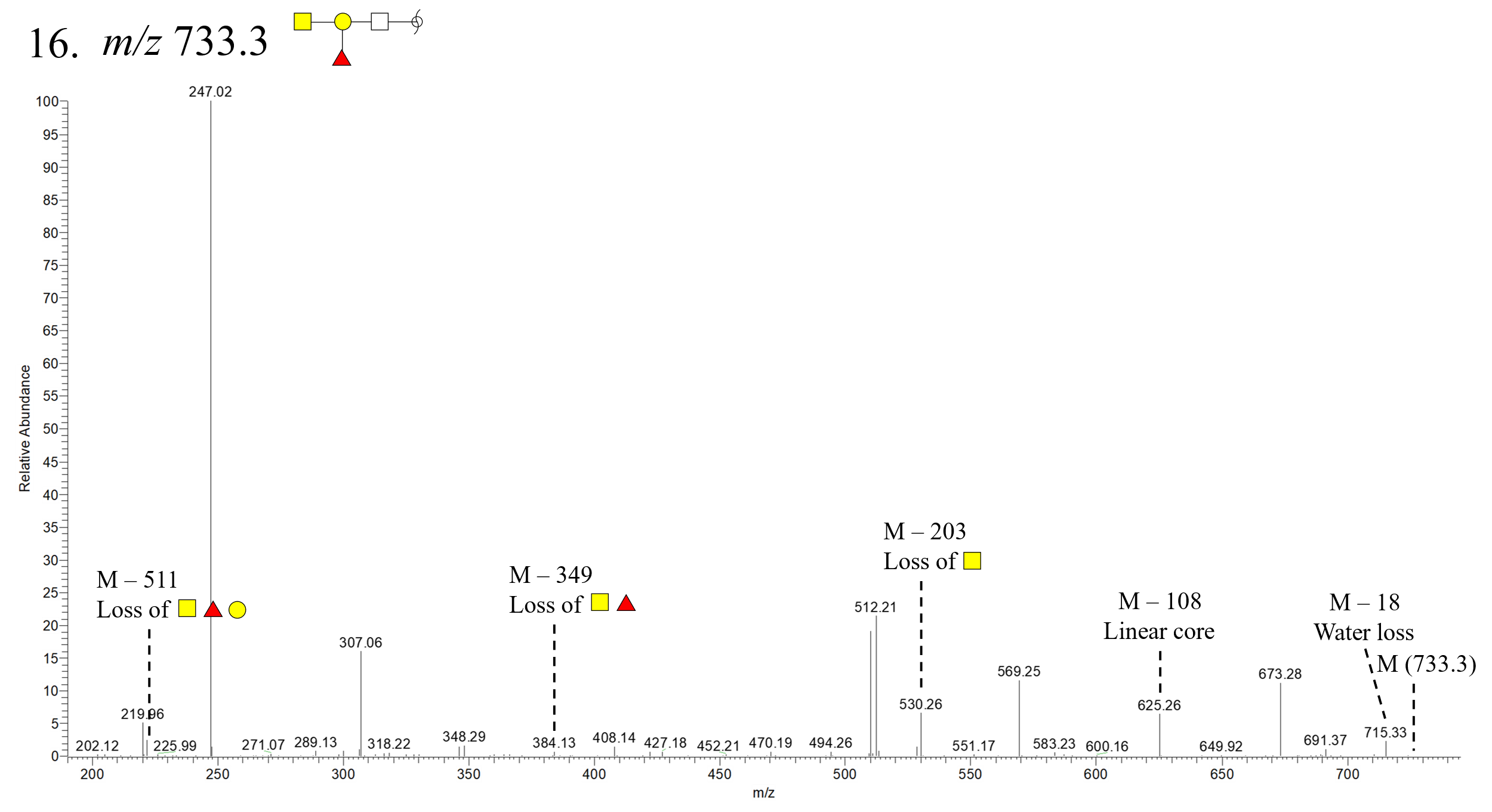


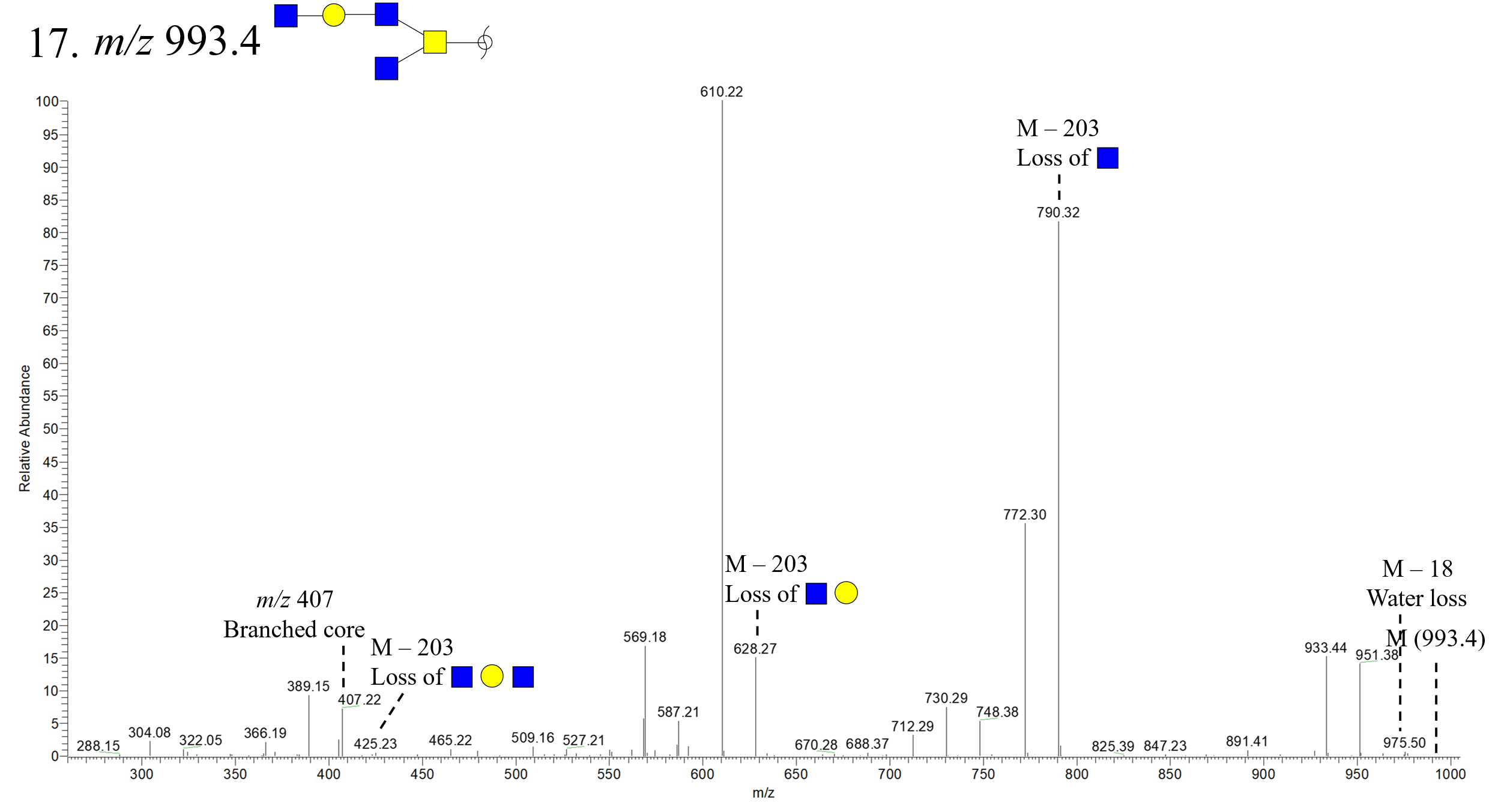


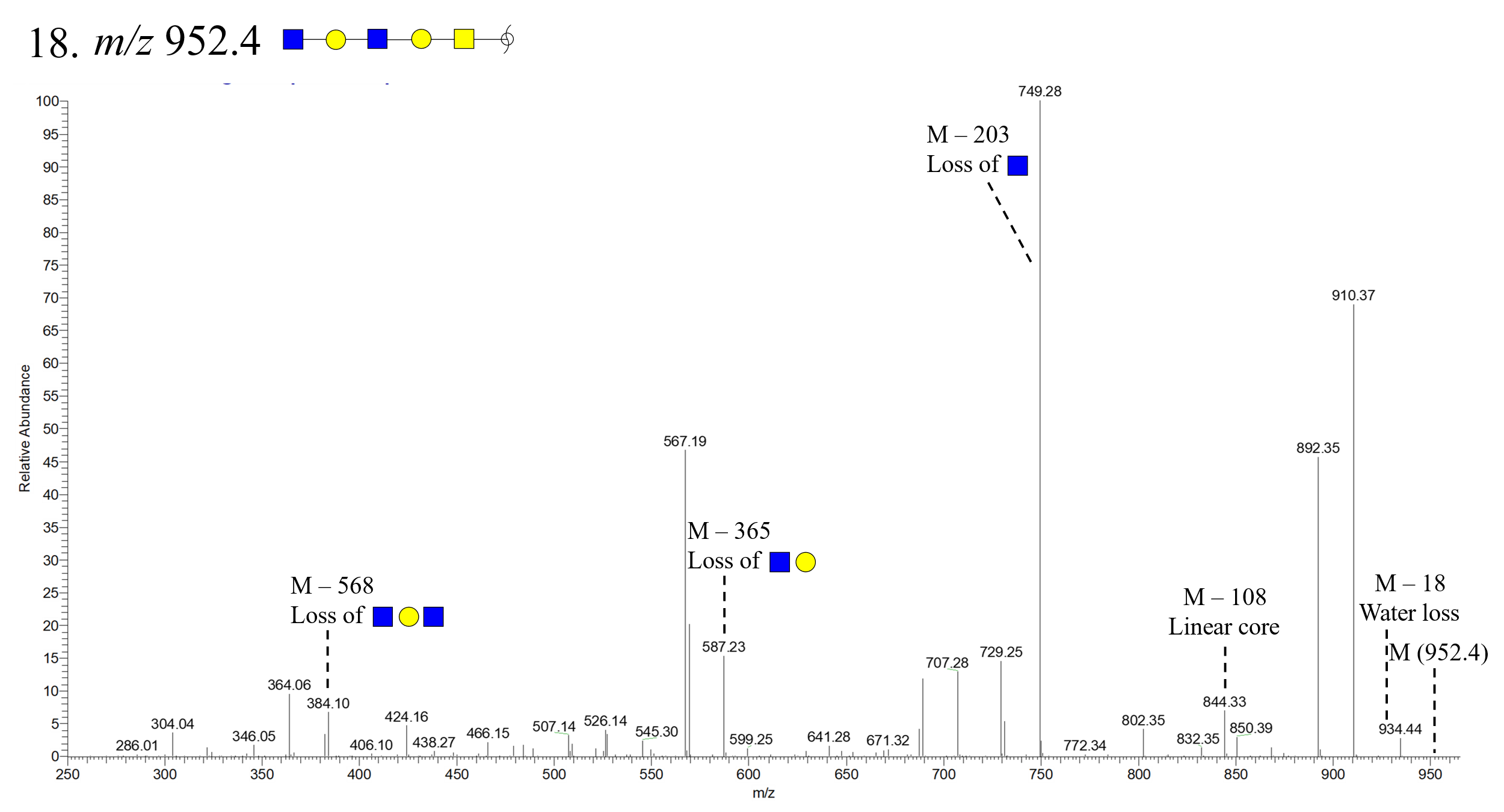


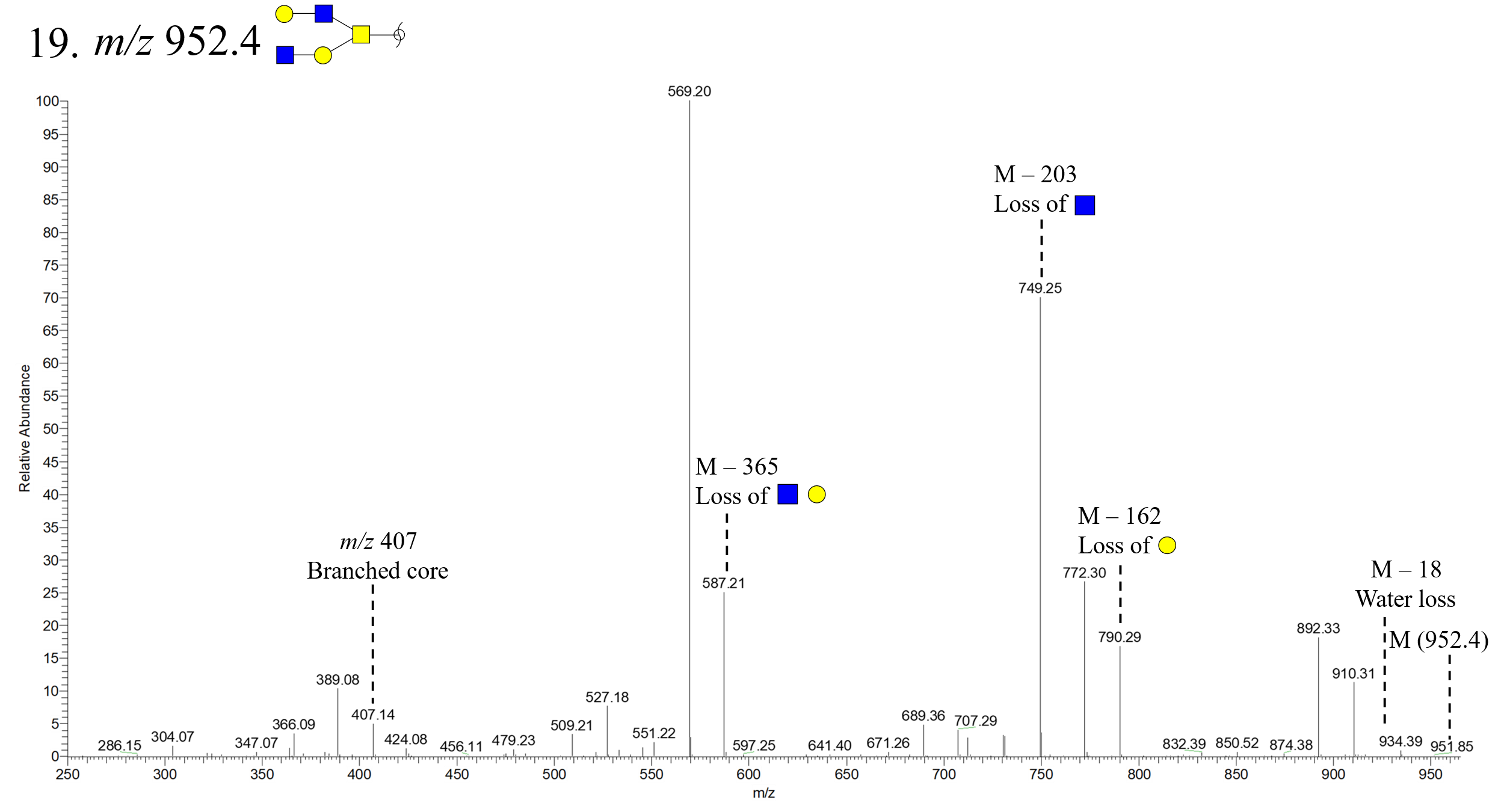


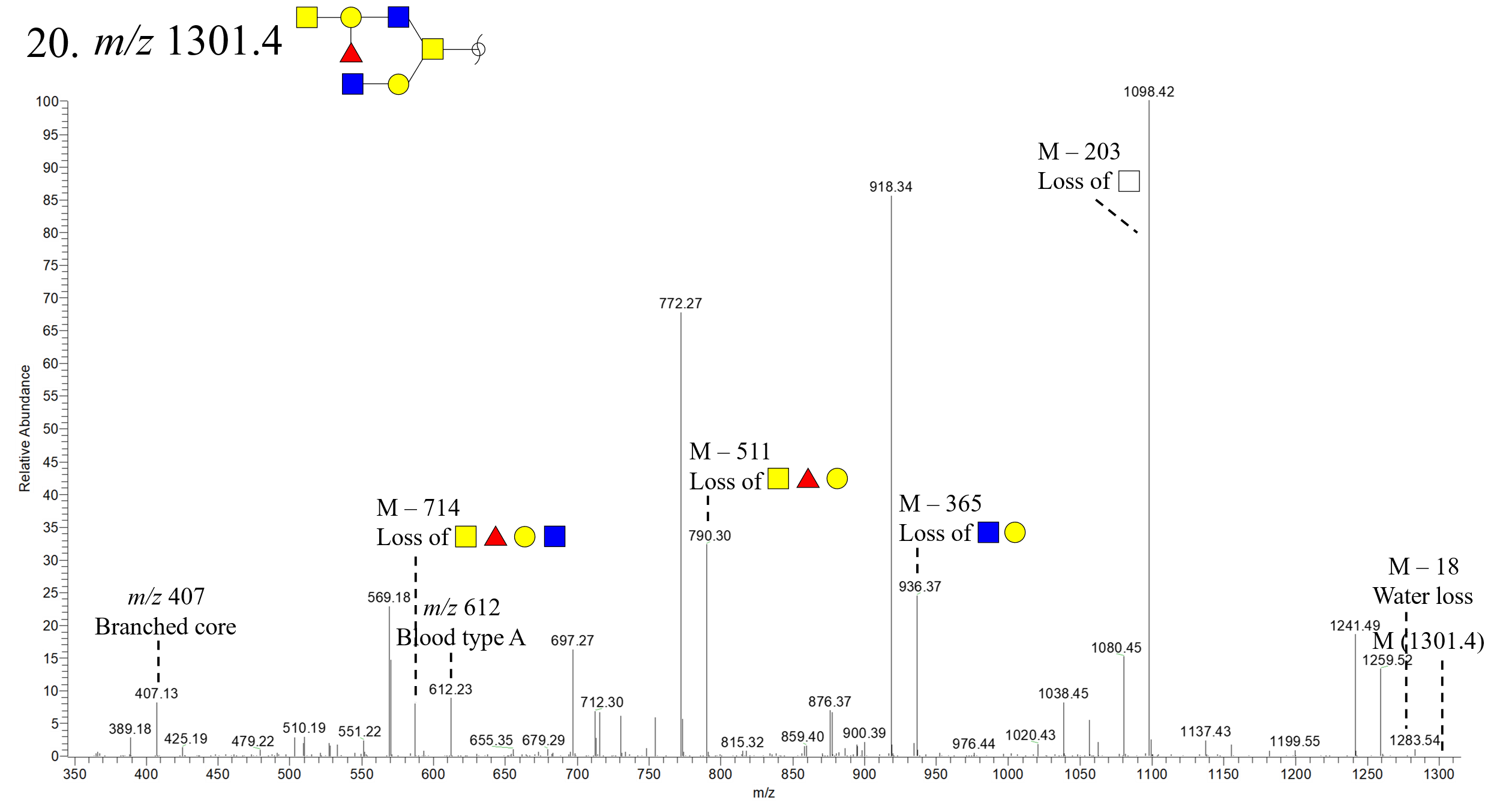


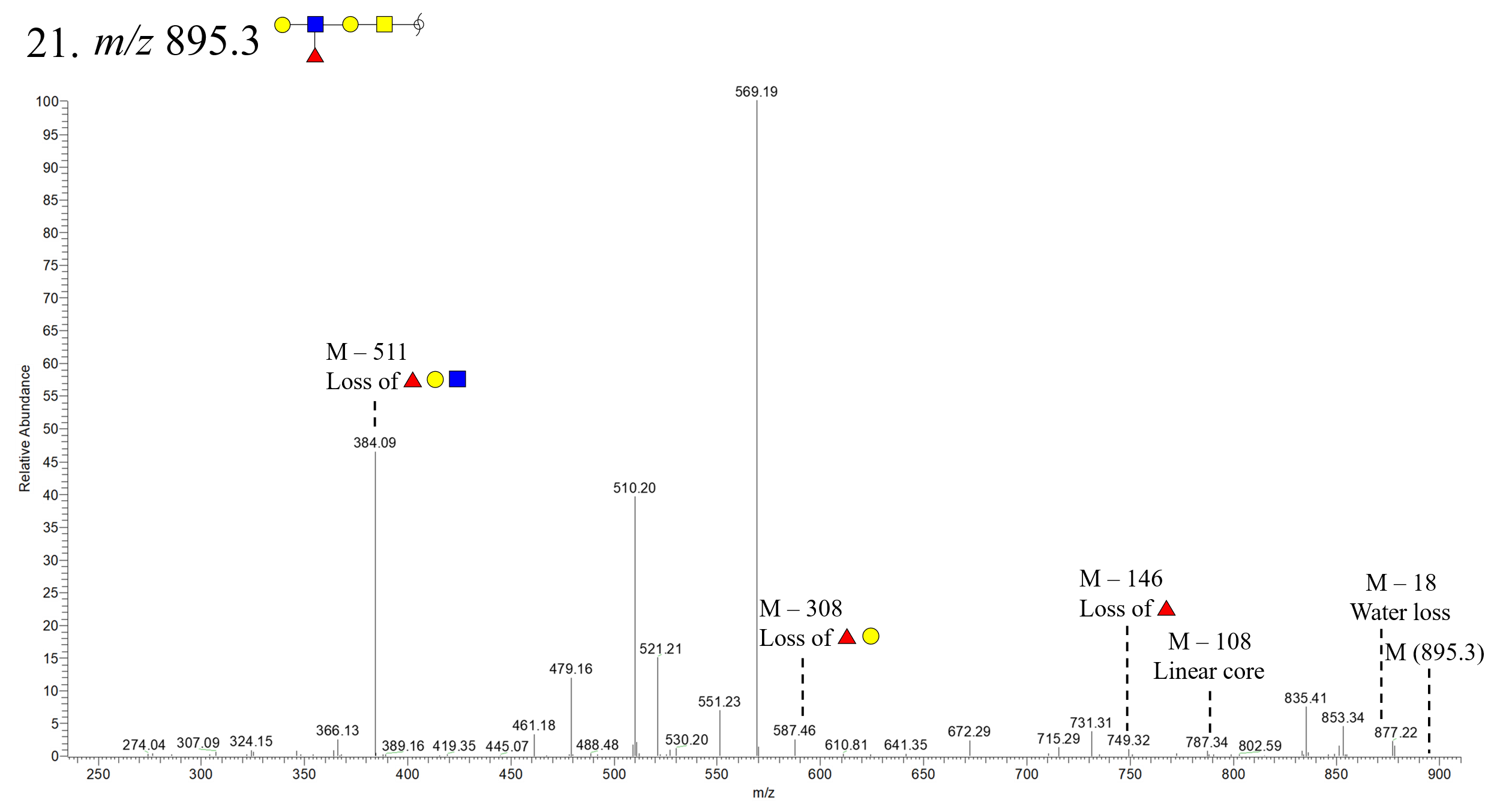


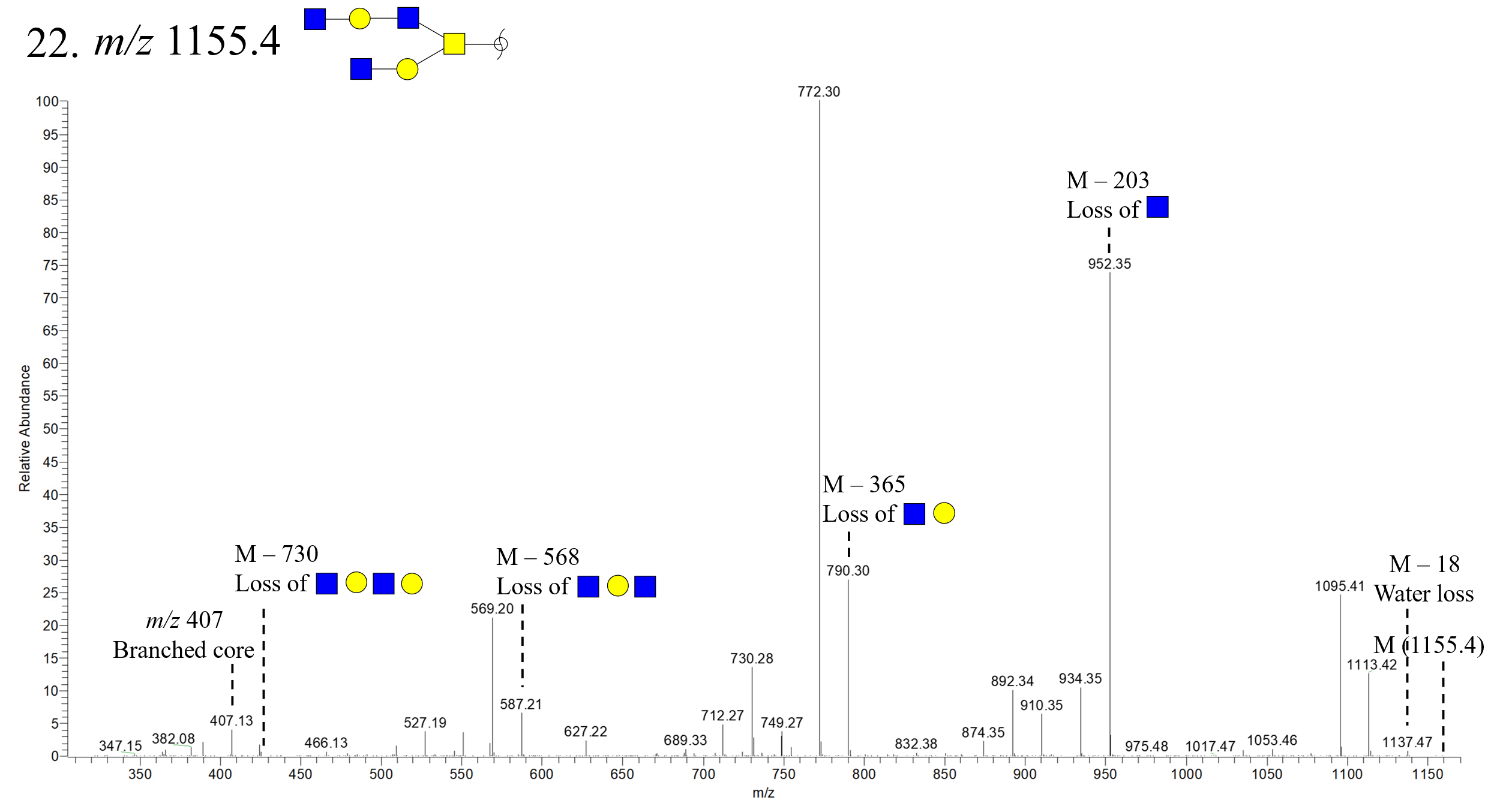


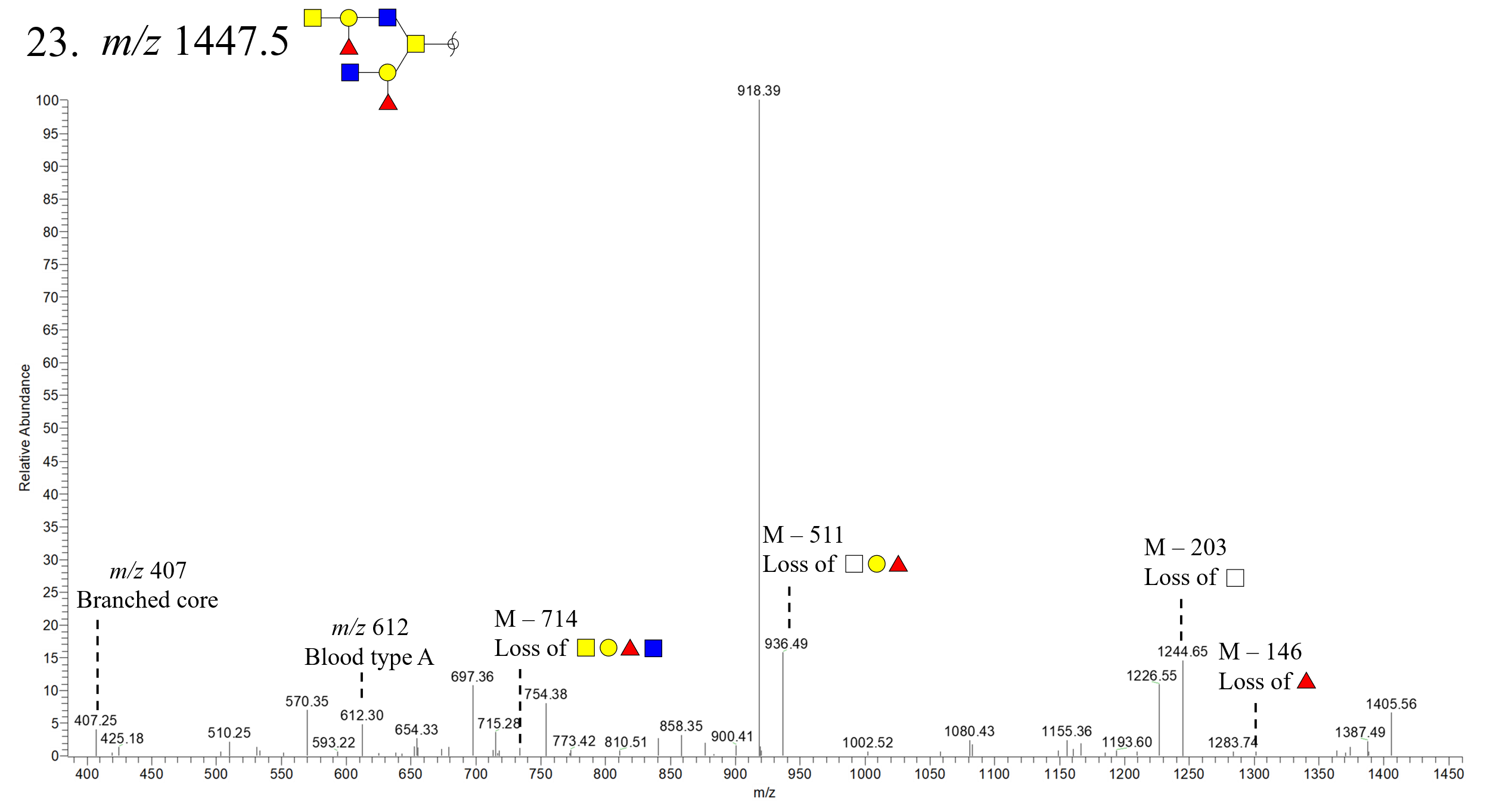


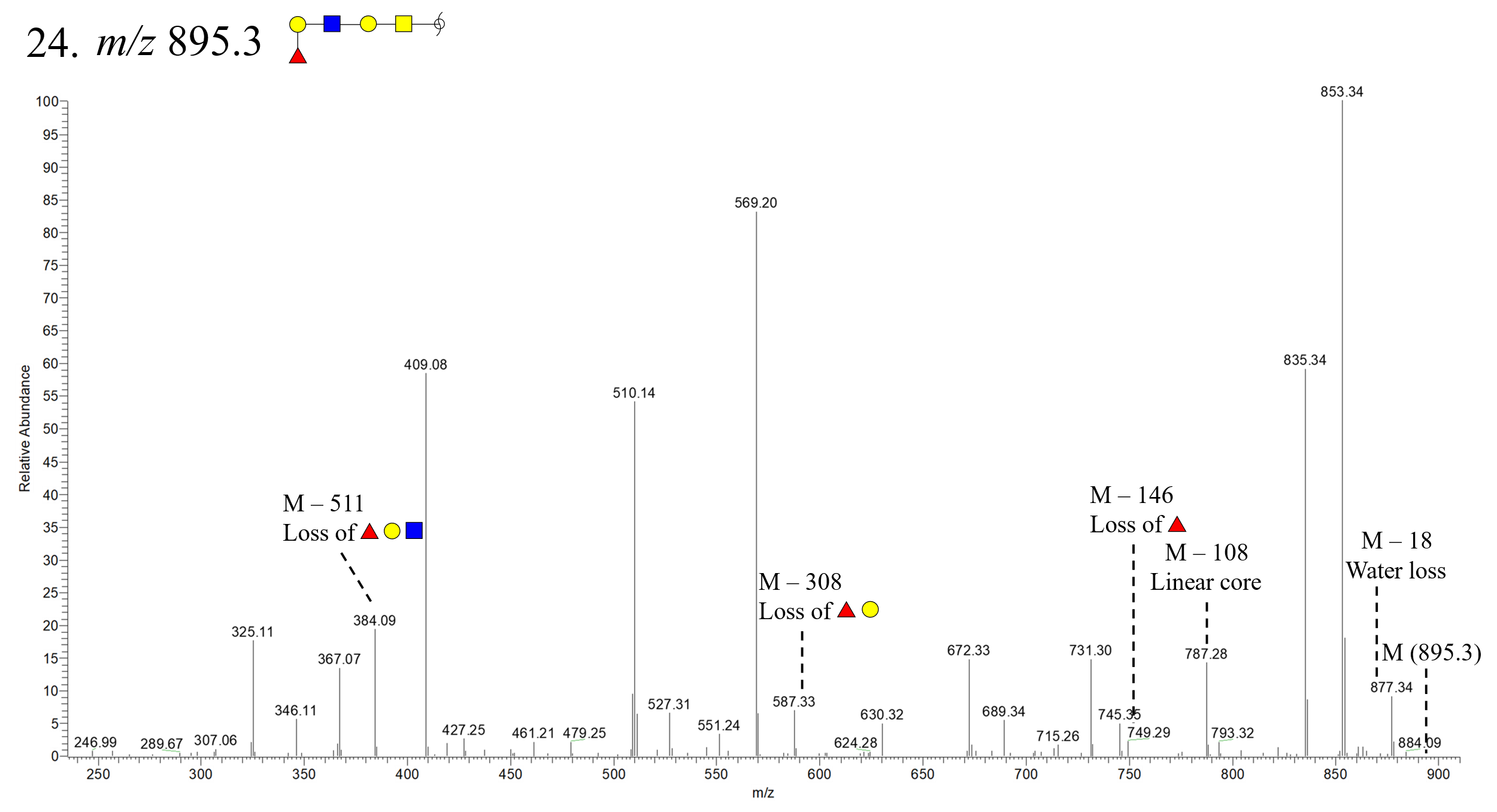


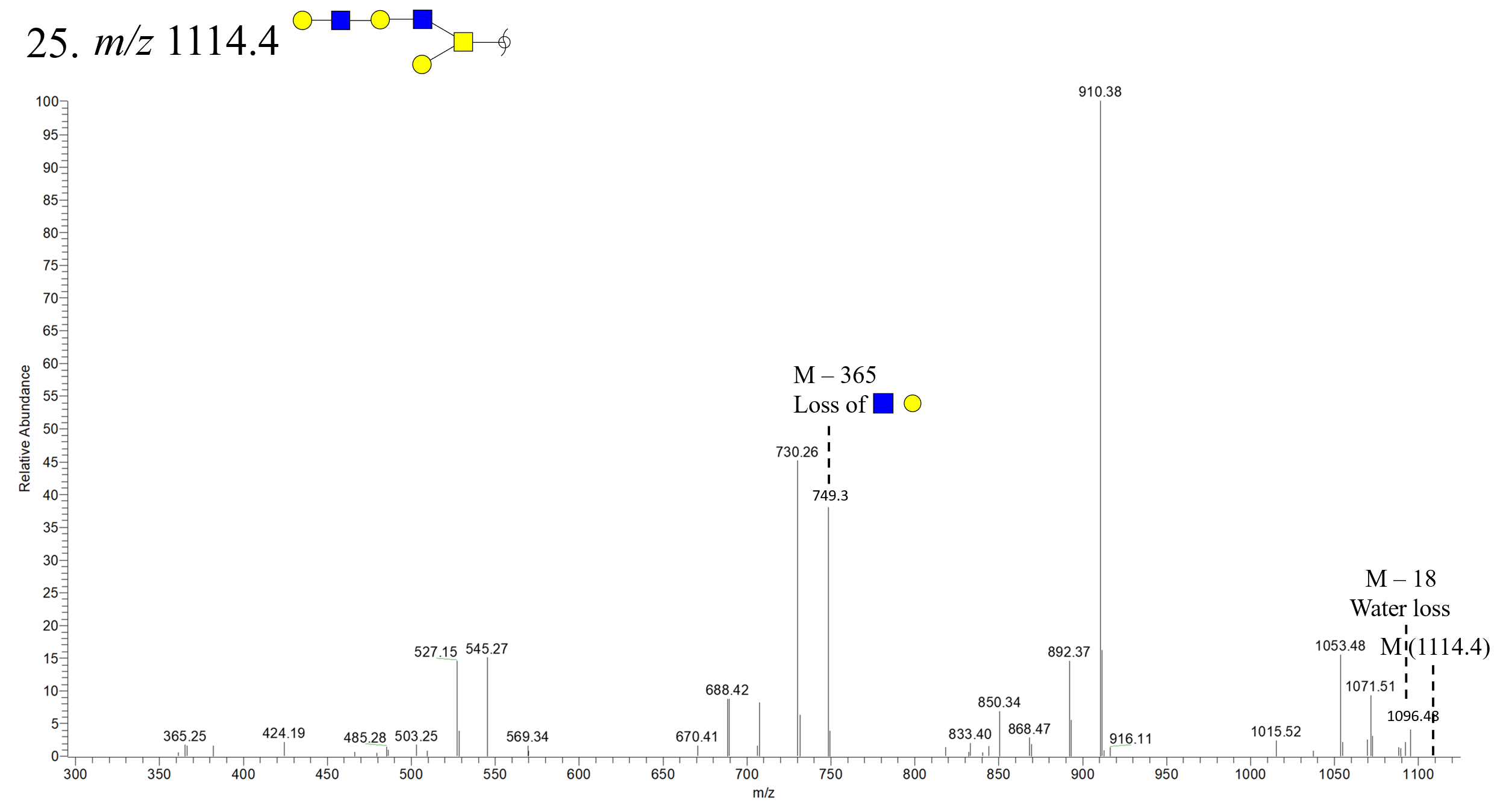


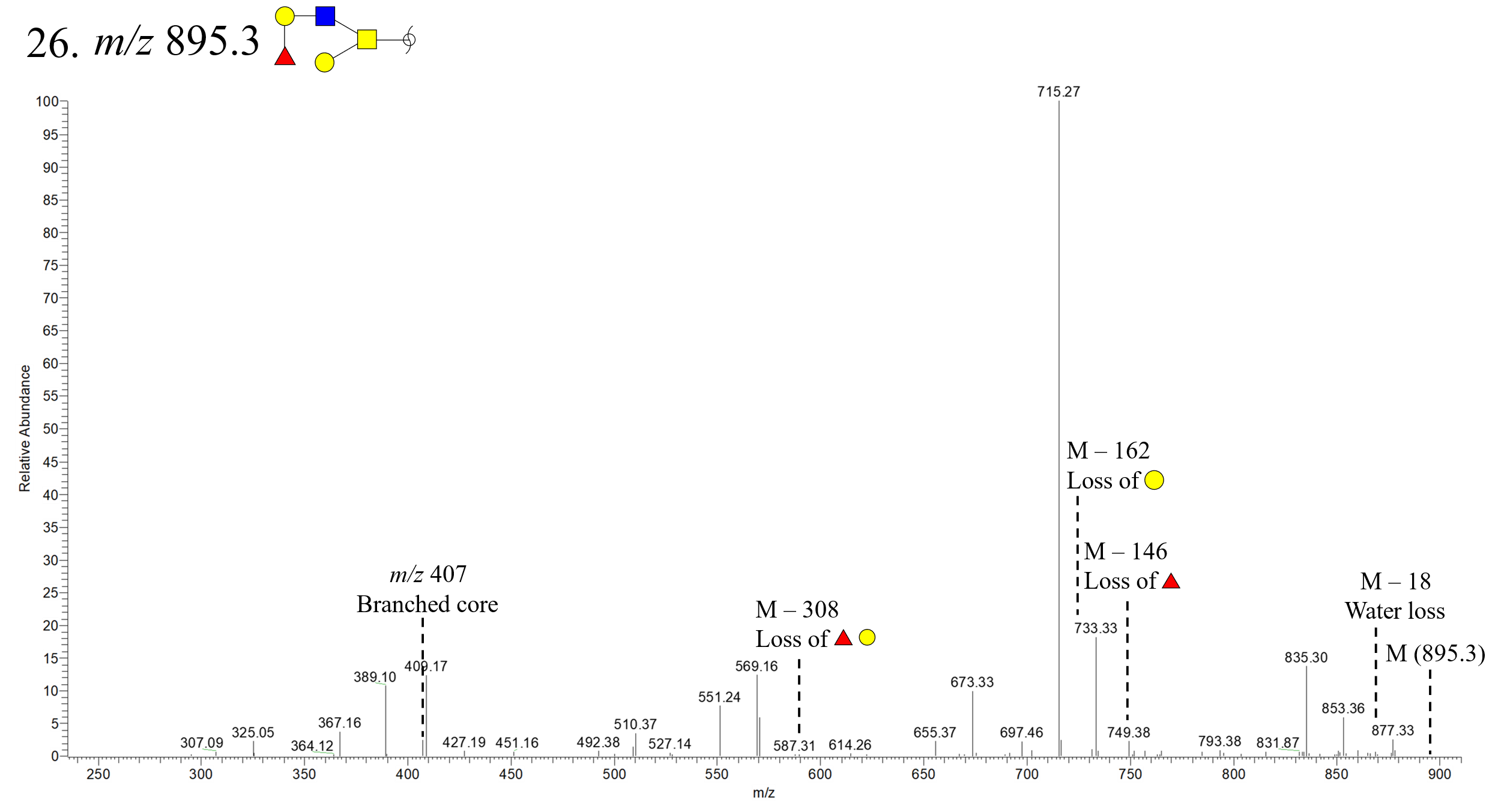


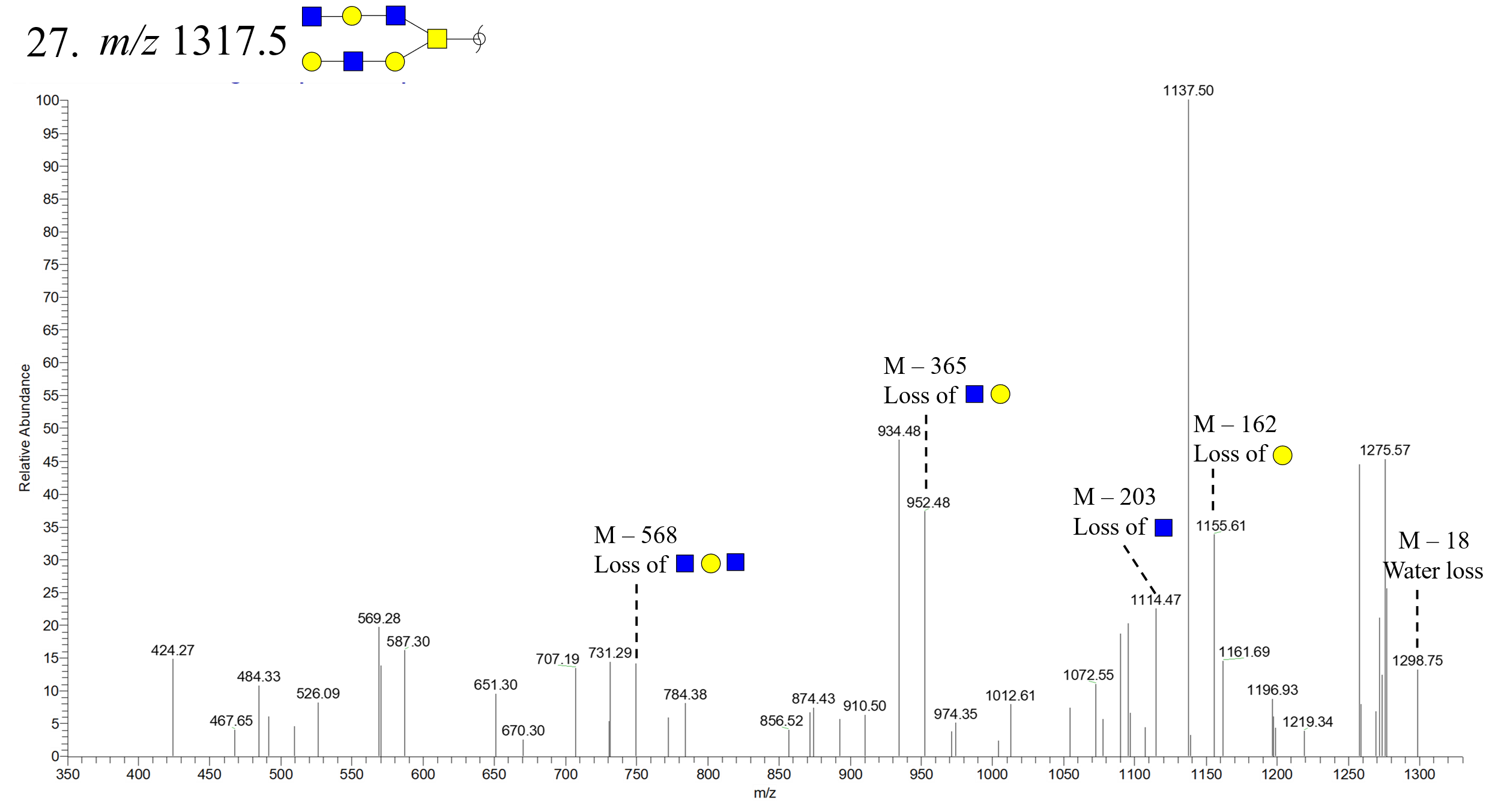


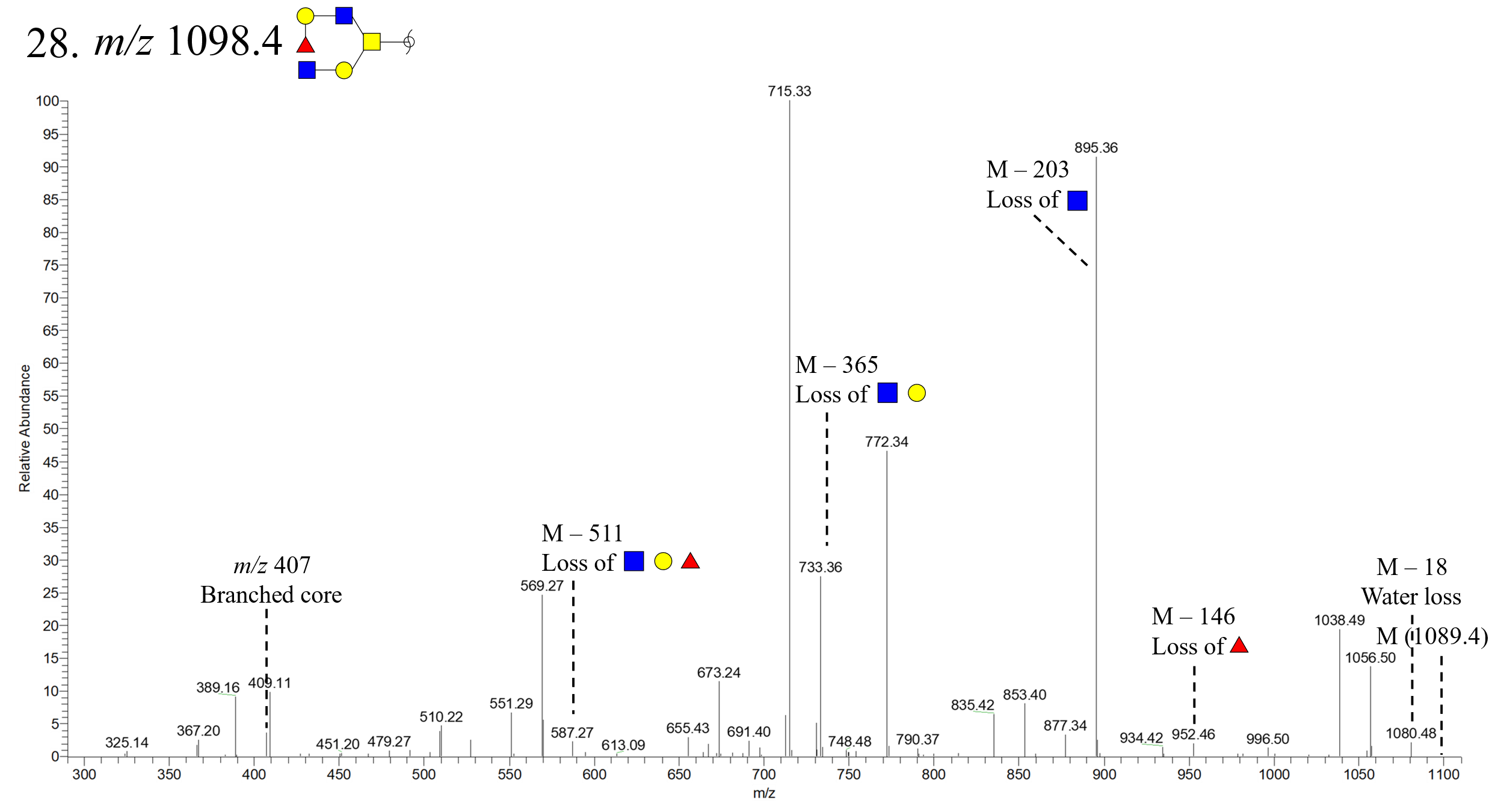


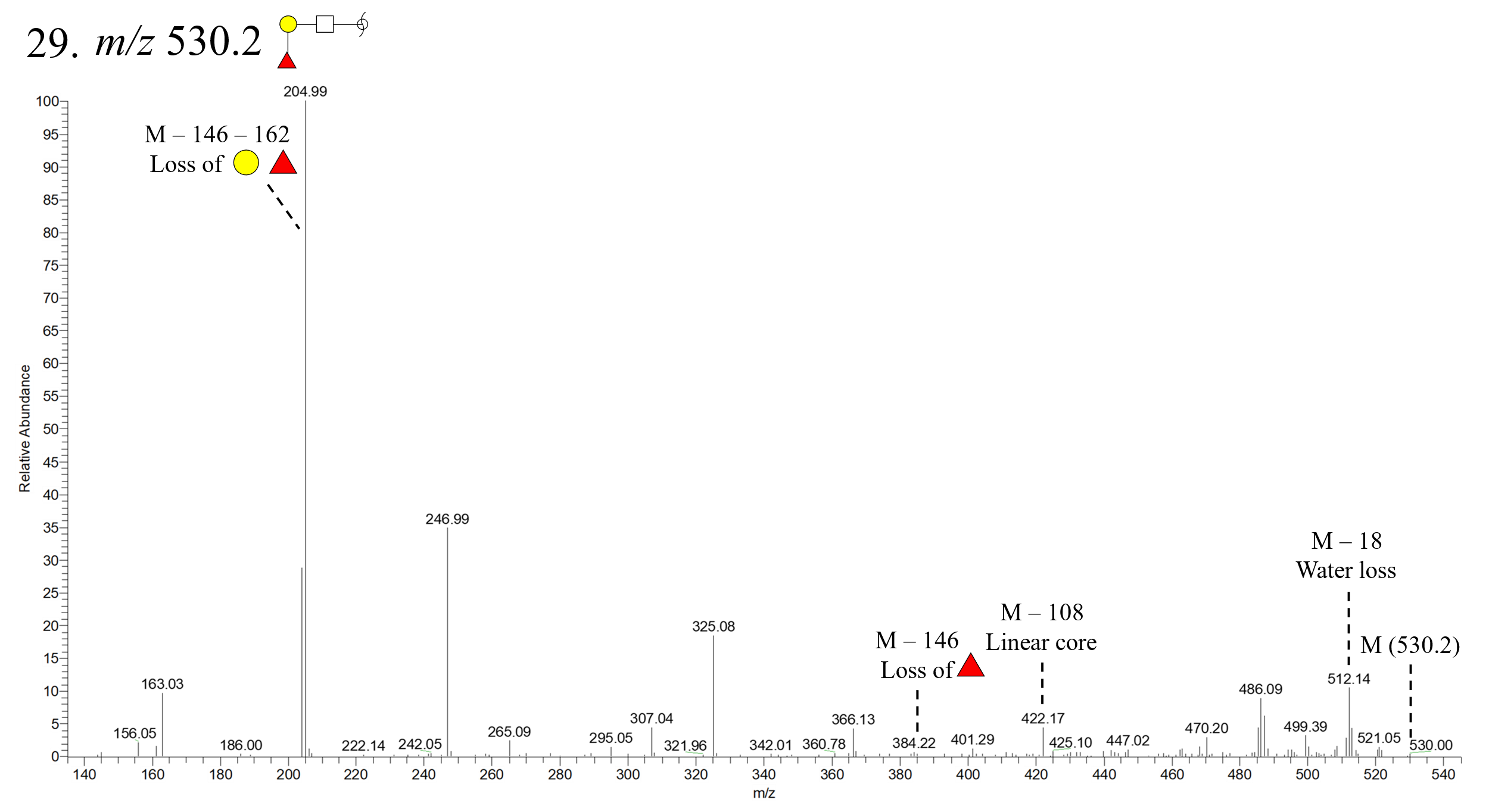


Figure S23. PGC-LC-MS/MS fragmentation spectra demonstrating the identification of the present *O-*glycans released from PGM. The fragmentation patterns are numbered (left corner) and these numbers correspond to the eluted peaks as indicated in Figure S22.


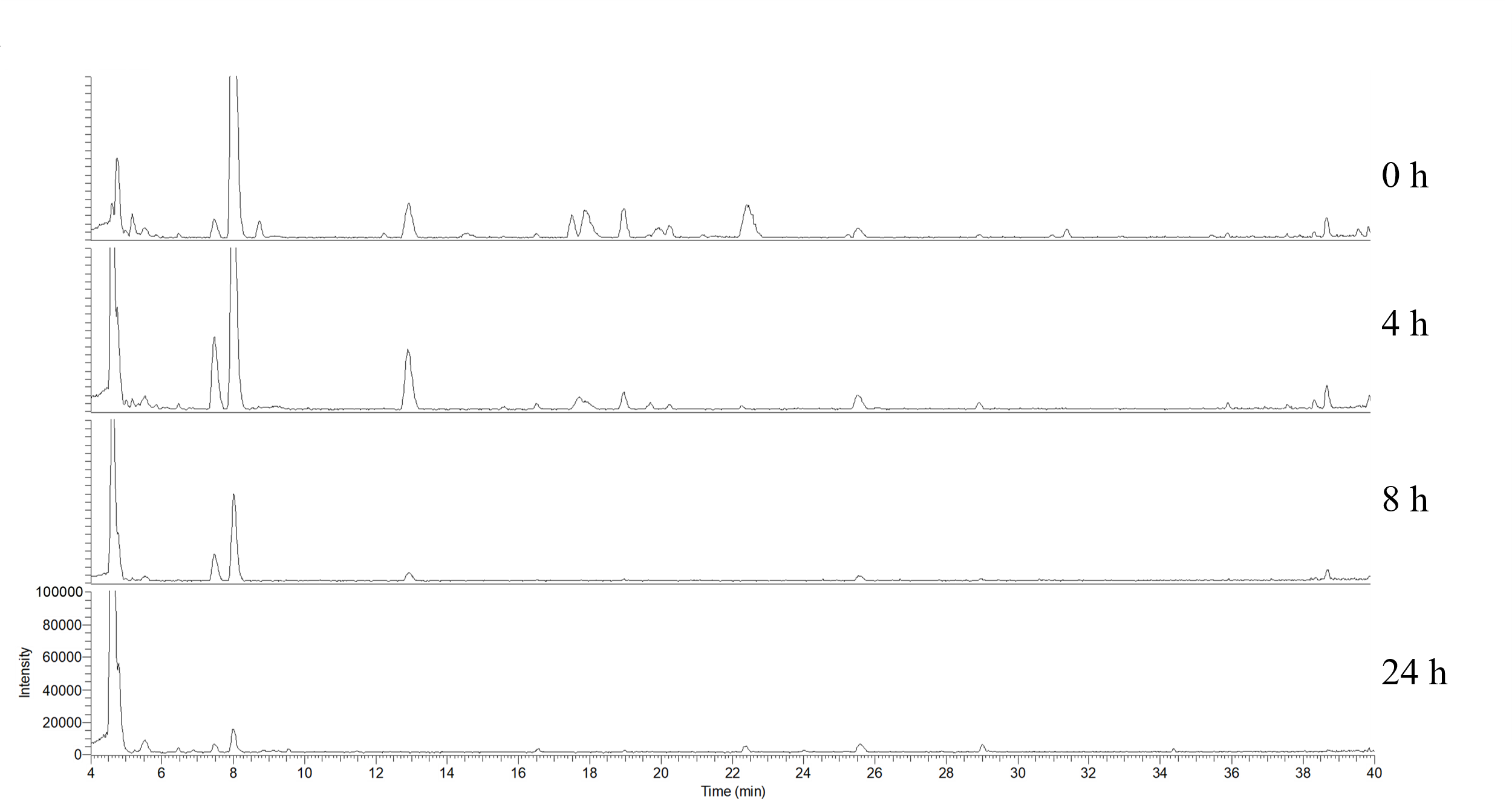


Figure S24. PGC-LC-MS elution patterns showing the degradation of *O*-glycans released from PGM during 24 h incubation with *R. torques* lysate.


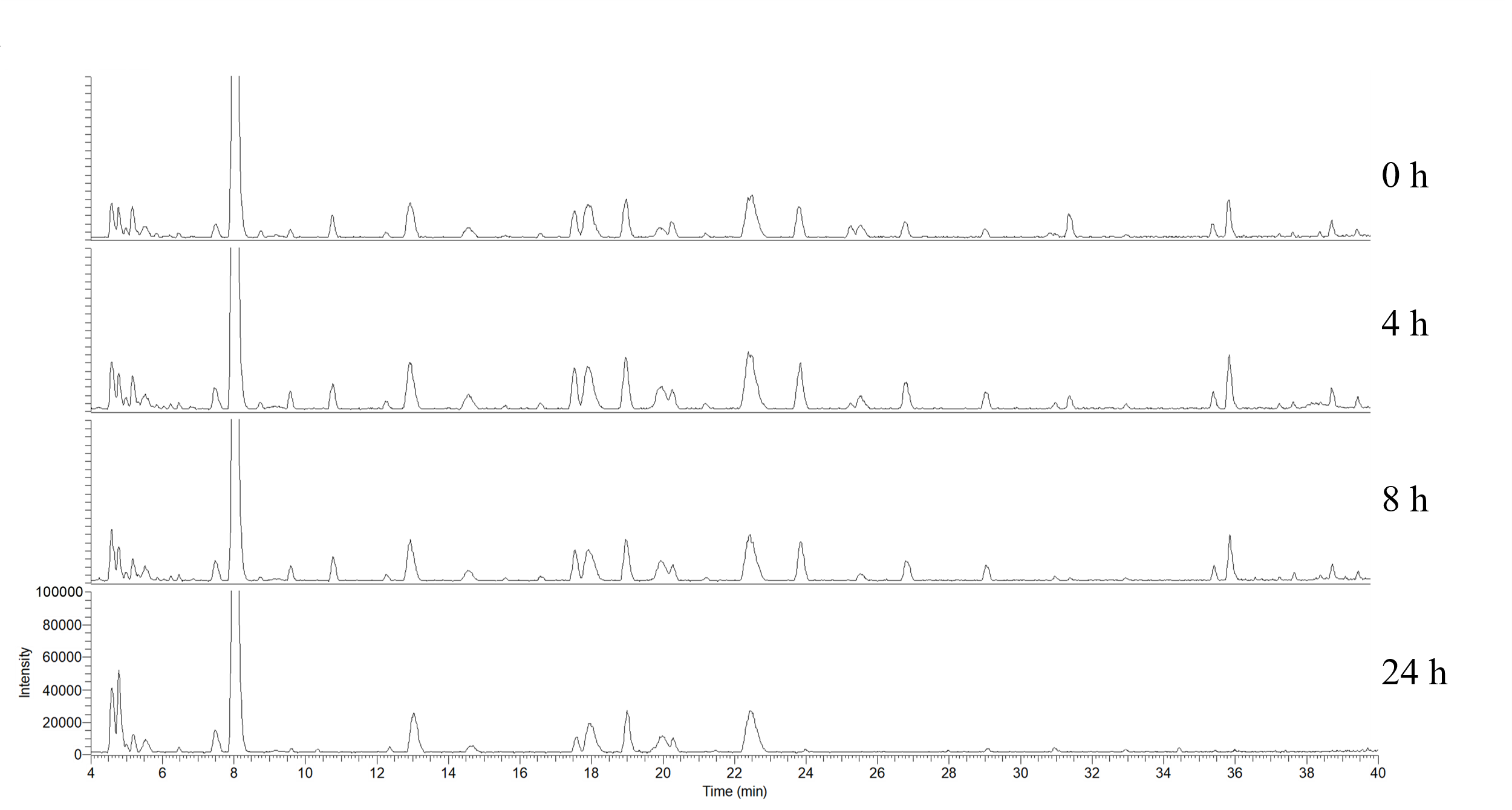


Figure S25. PGC-LC-MS elution patterns showing the degradation of *O*-glycans released from PGM during 24 h incubation with *B. thetaiotaomicron* lysate.


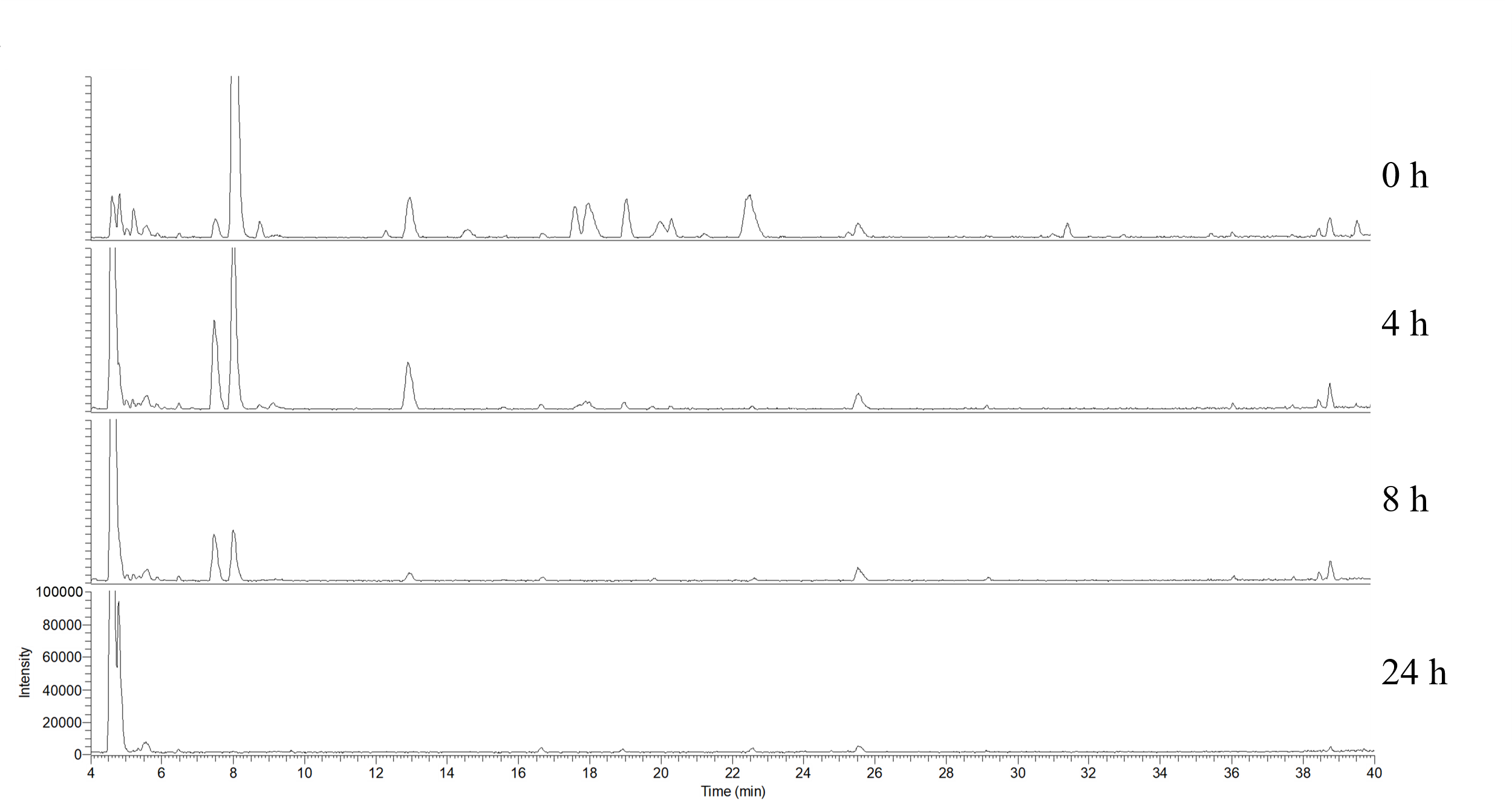


Figure S26. PGC-LC-MS elution patterns showing the degradation of *O*-glycans released from PGM during 24 h incubation with *A. muciniphila*/*R. torques* lysate.


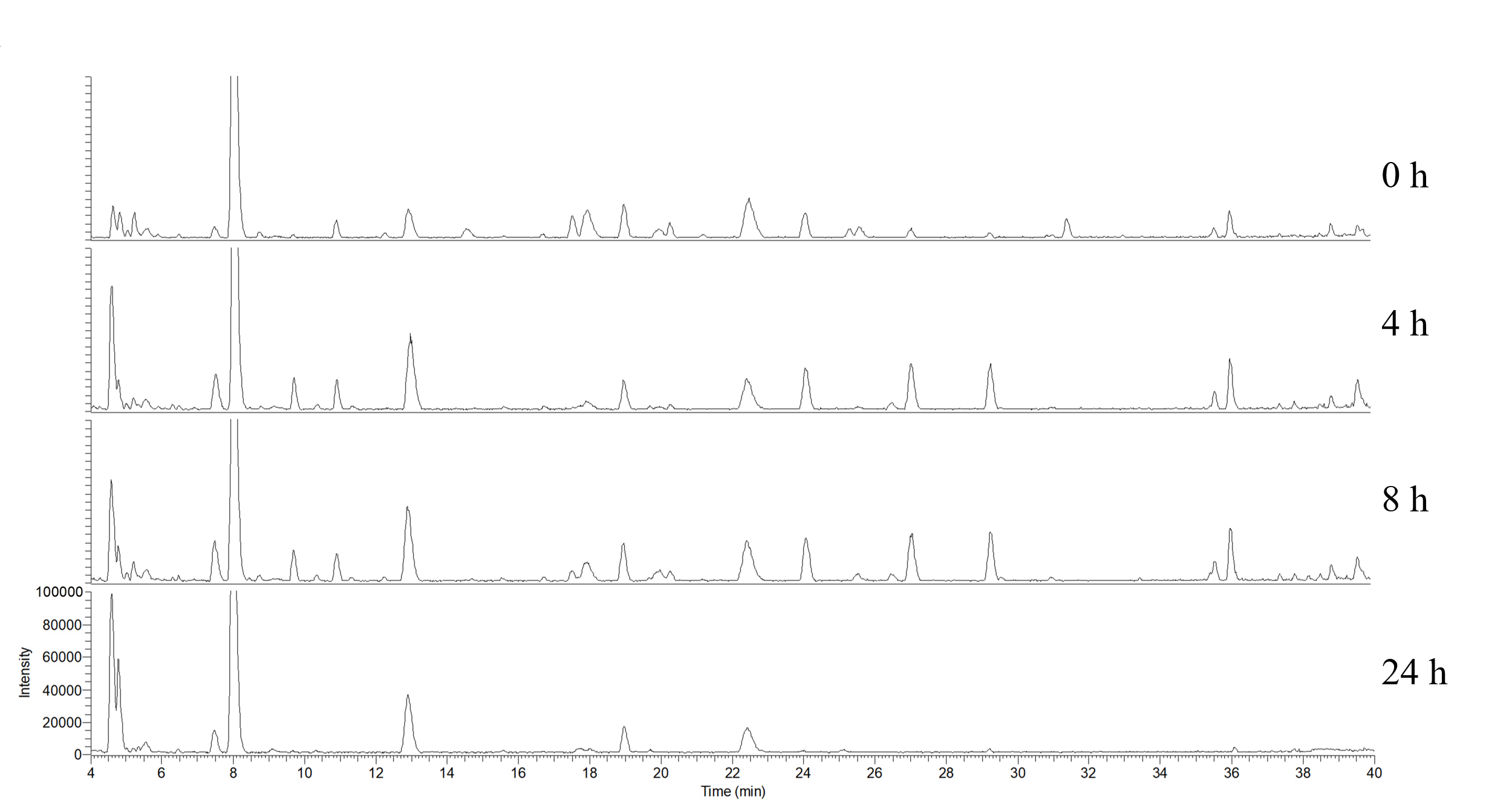


Figure S27. PGC-LC-MS elution patterns showing the degradation of *O*-glycans released from PGM during 24 h incubation with *A. muciniphila*/*B. thetaiotaomicron* lysate.


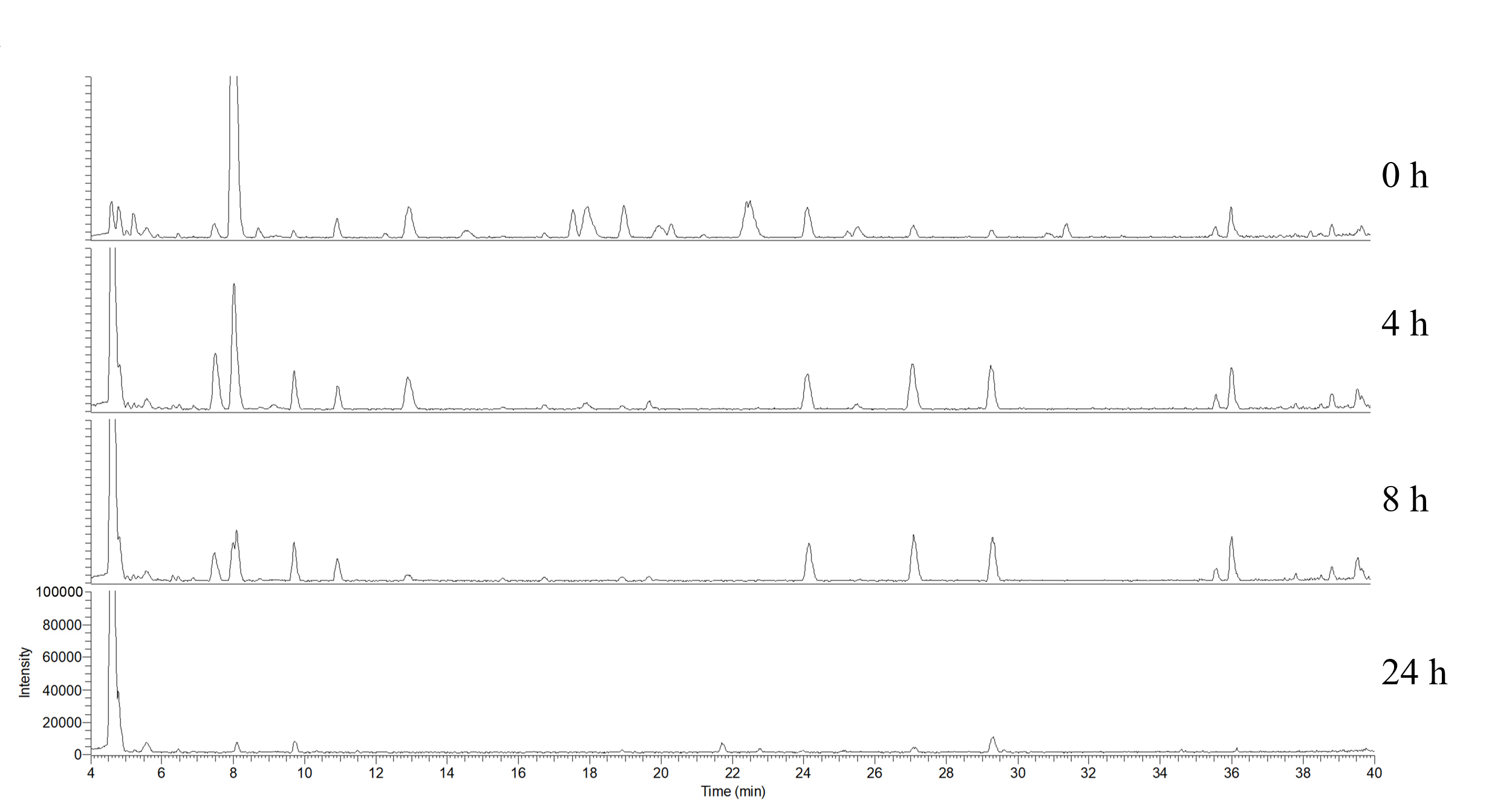


Figure S28. PGC-LC-MS elution patterns showing the degradation of *O*-glycans released from PGM during 24 h incubation with *R. torques*/*B. thetaiotaomicron* lysate.


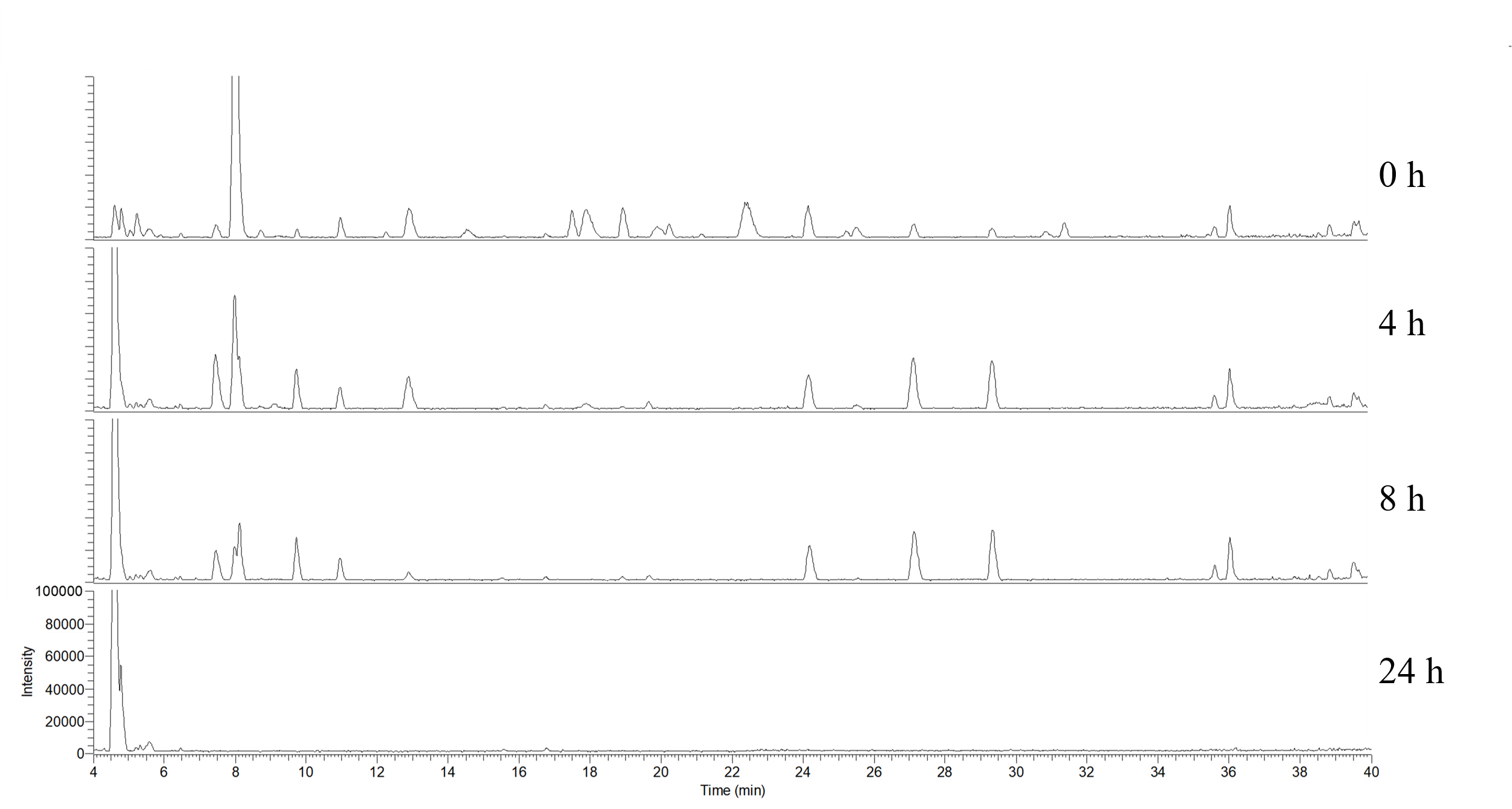


Figure S29. PGC-LC-MS elution patterns showing the degradation of *O*-glycans released from PGM during 24 h incubation with *A. muciniphila*/*R. torques*/*B. thetaiotaomicron* lysate.


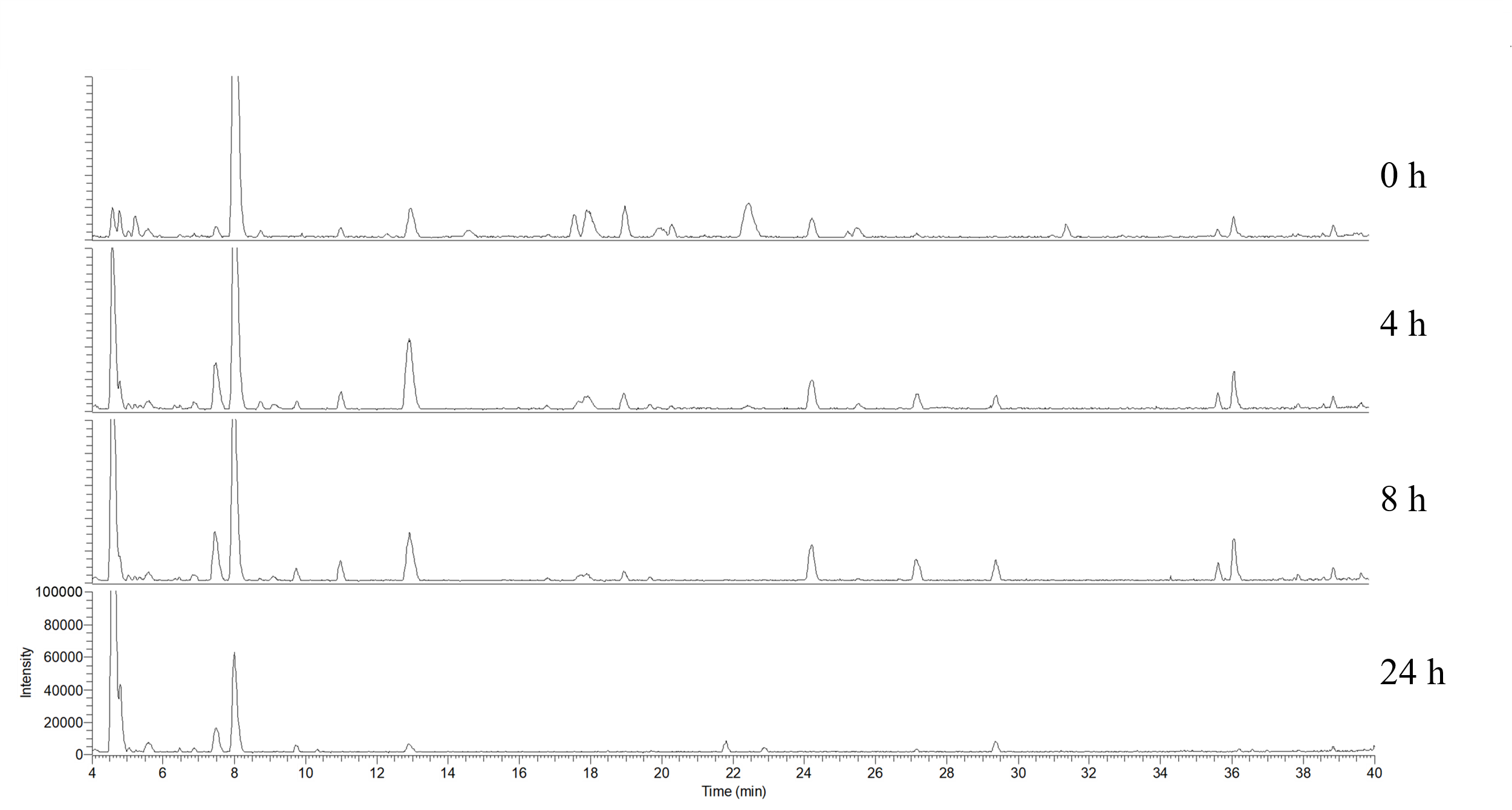


Figure S30. PGC-LC-MS elution patterns showing the degradation of *O*-glycans released from PGM during 24 h incubation with the mucin-degrading synthetic community (MDSC) lysate.


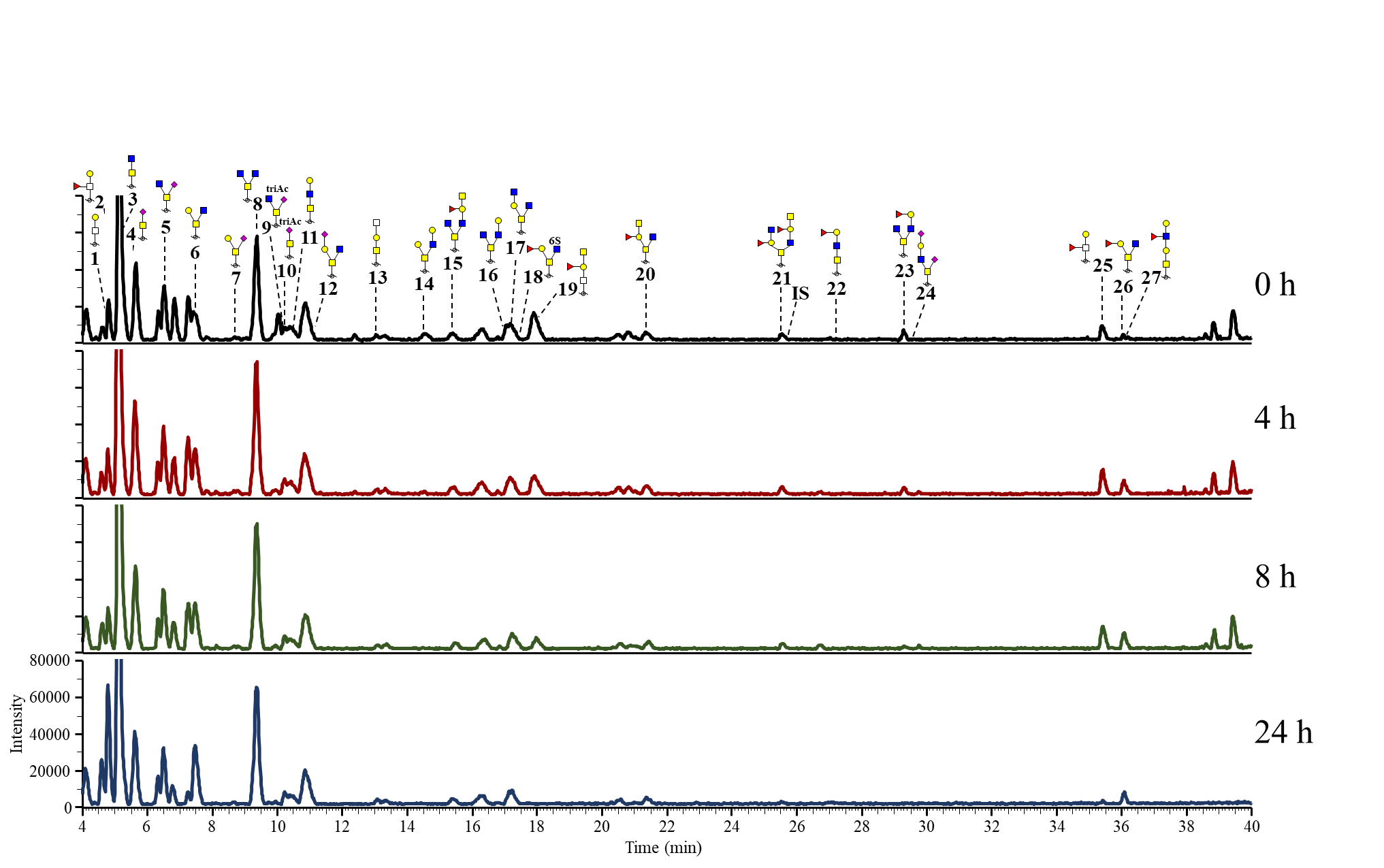


Figure S31. PGC-LC-MS elution patterns showing the degradation of *O*-glycans released from BSM during 24 h incubation with *A. muciniphila* lysate. Identified peaks (0 h) are indicated with a number and structure which correspond to the fragmentation MS/MS data as shown in Figure S32.


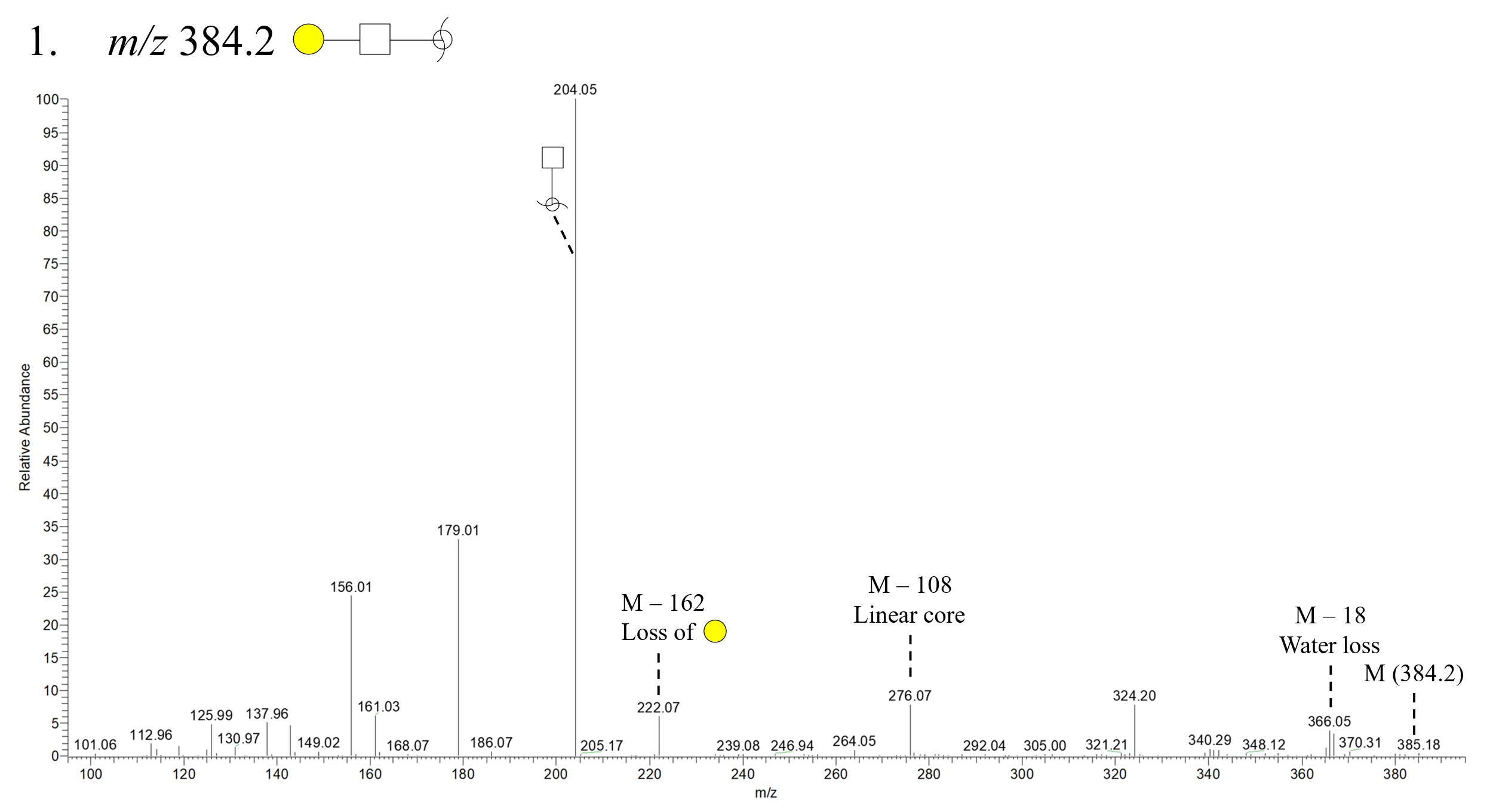

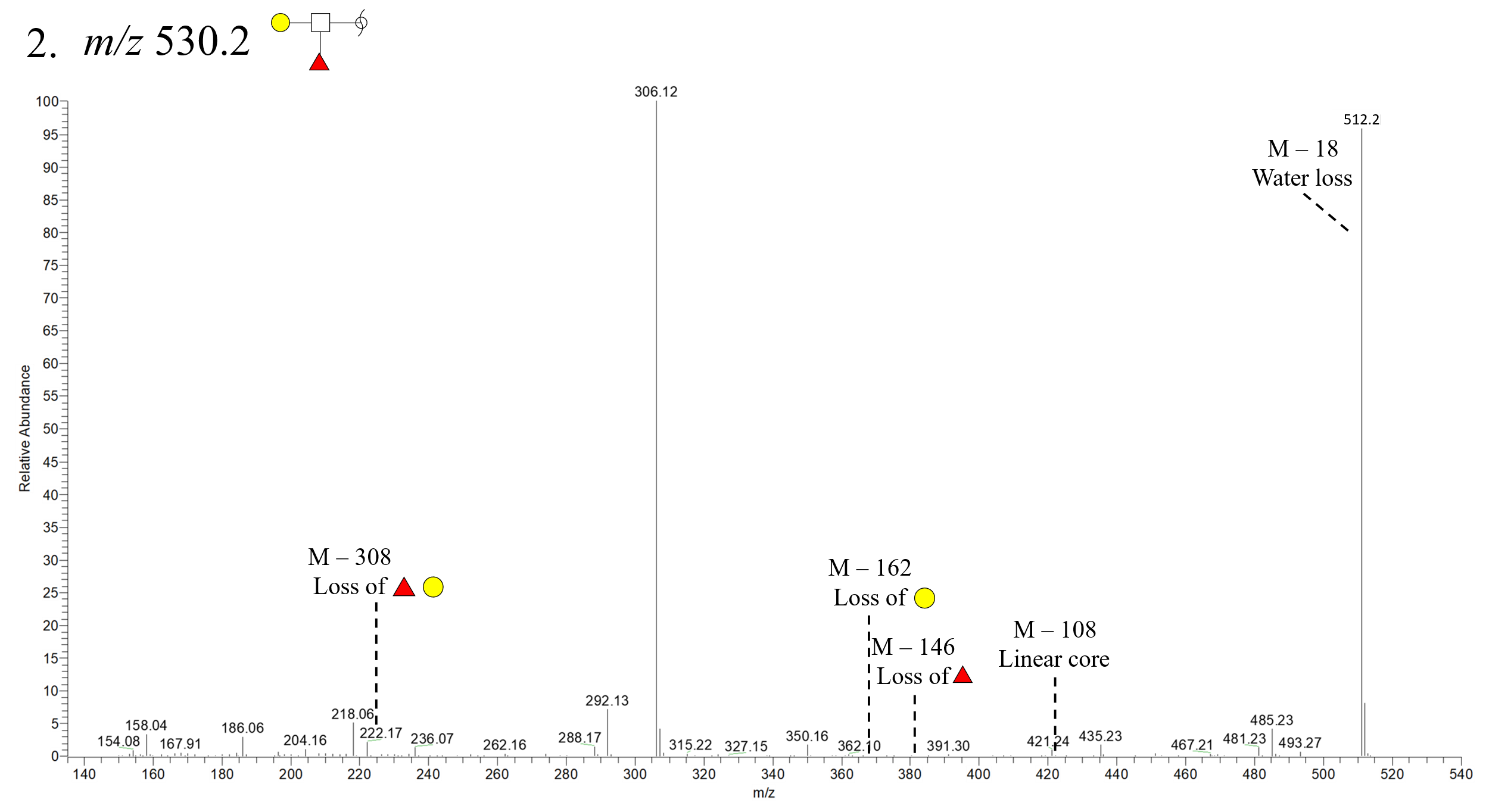

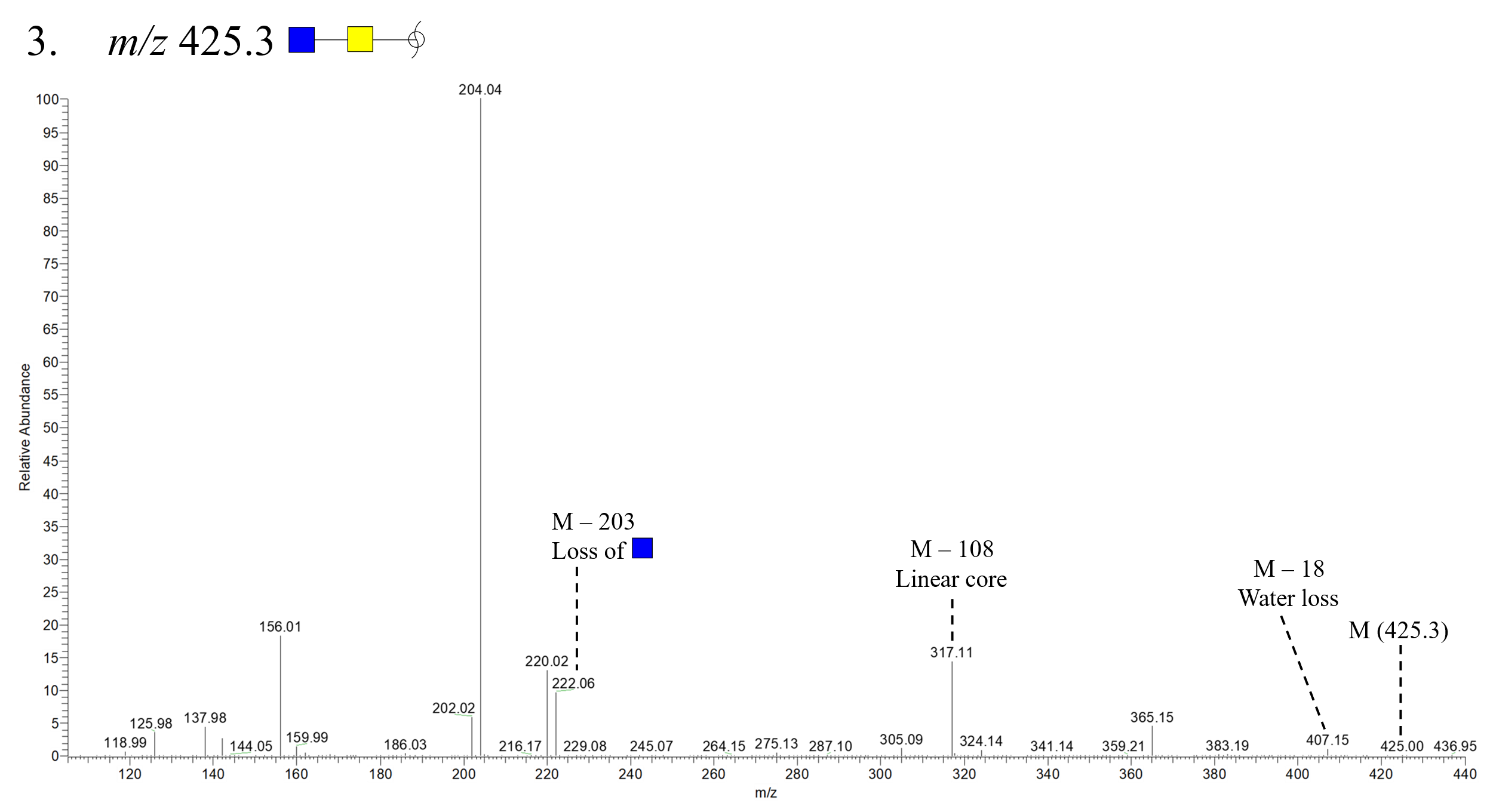


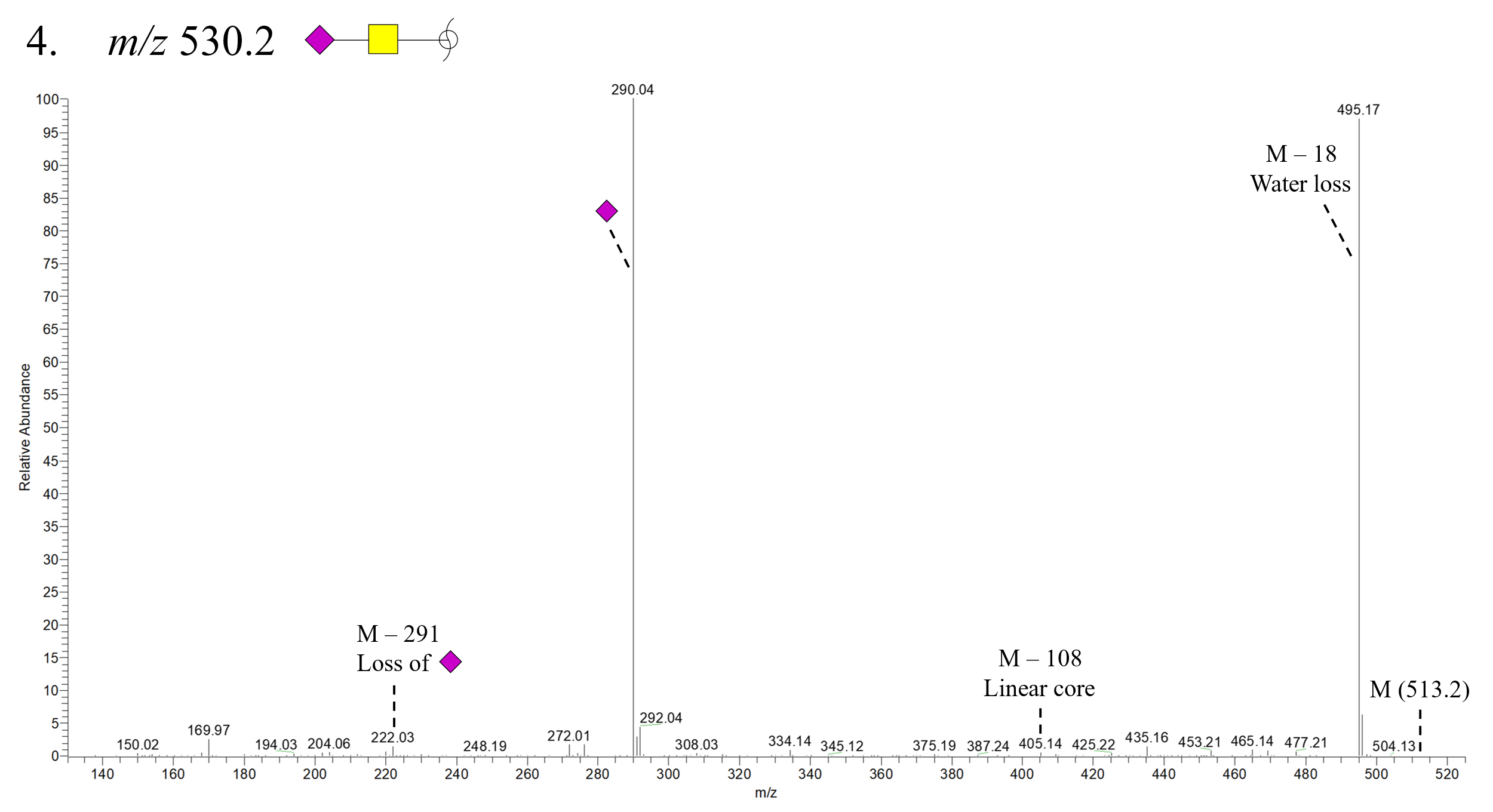

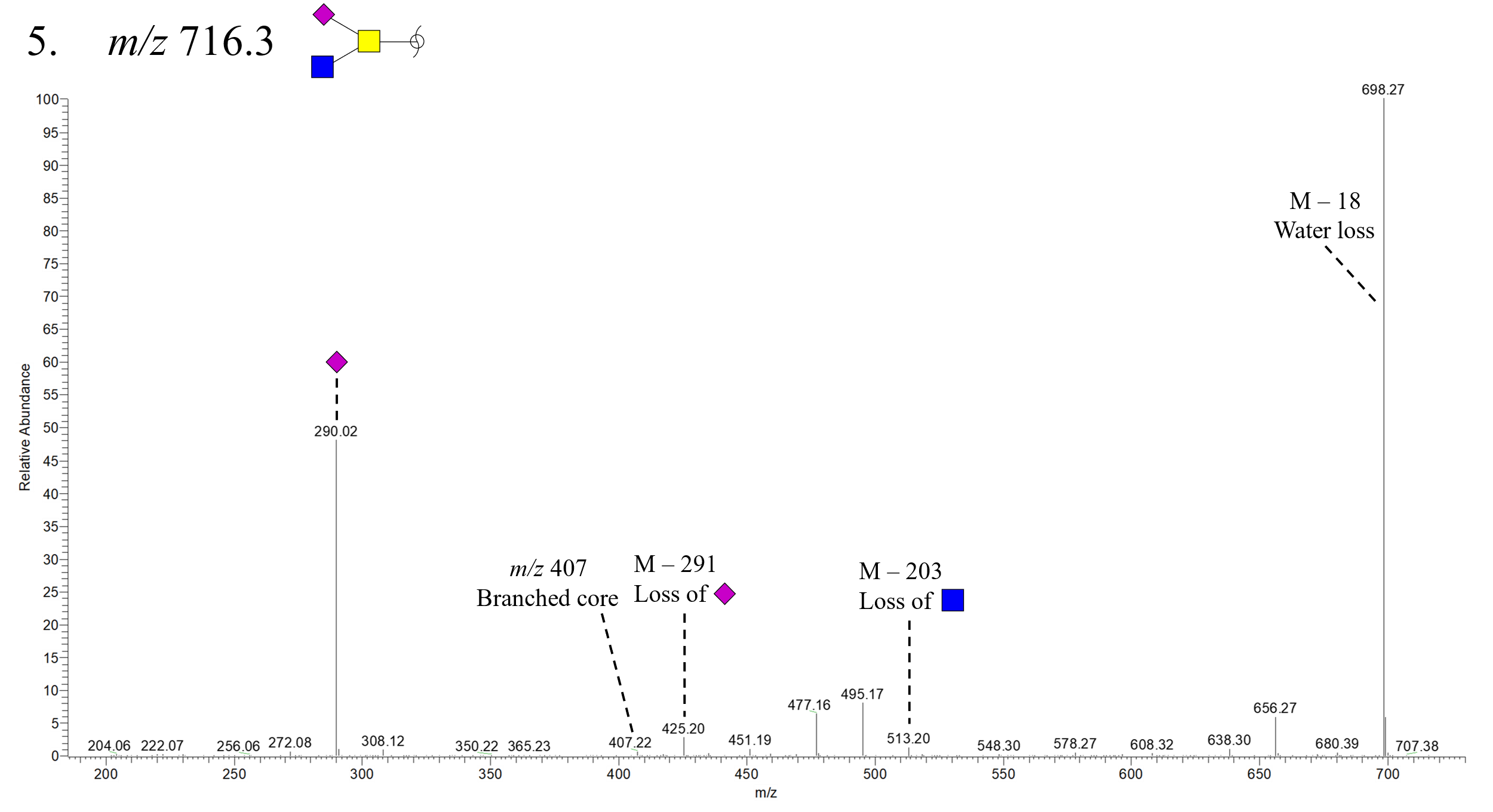

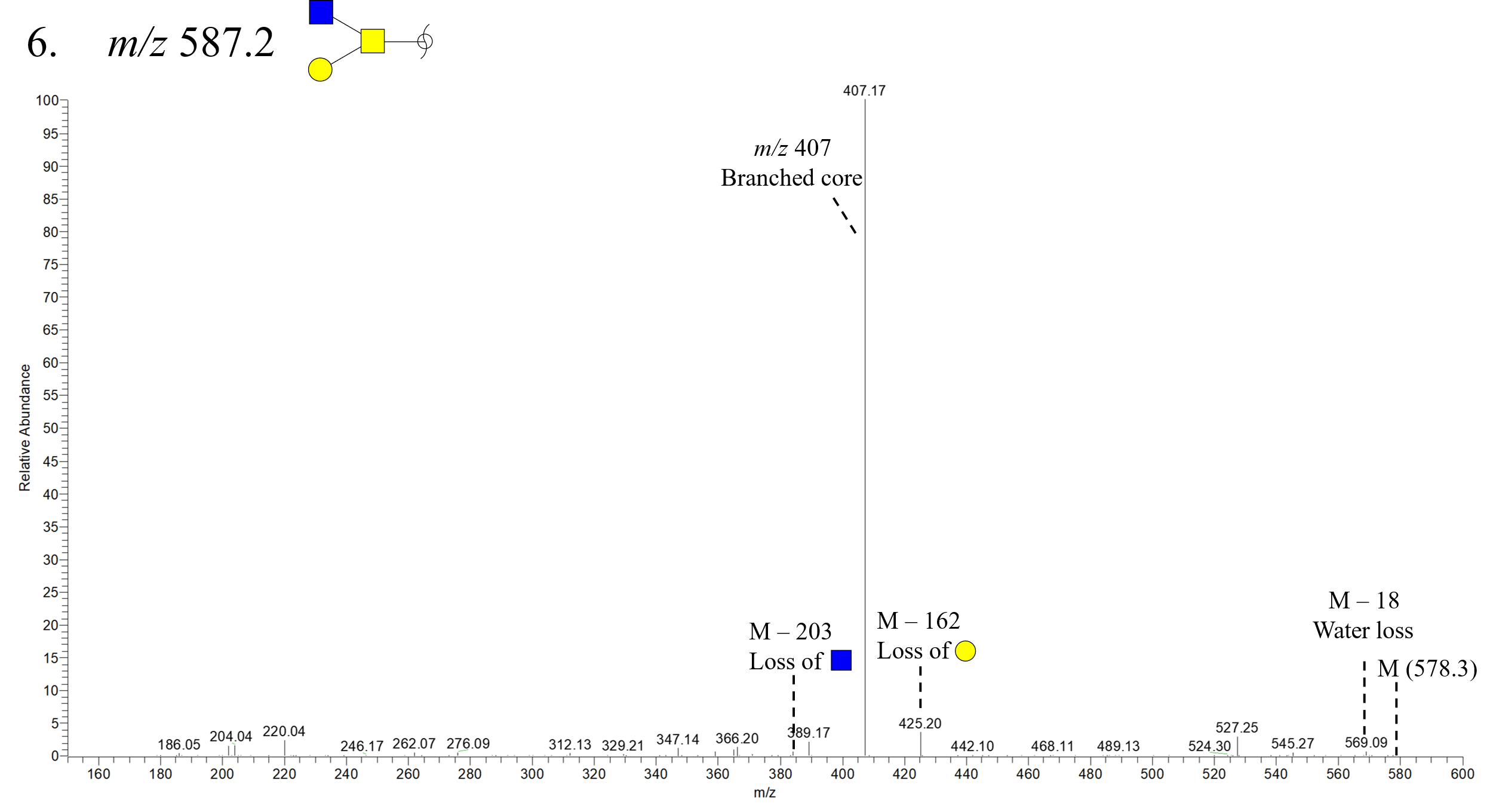


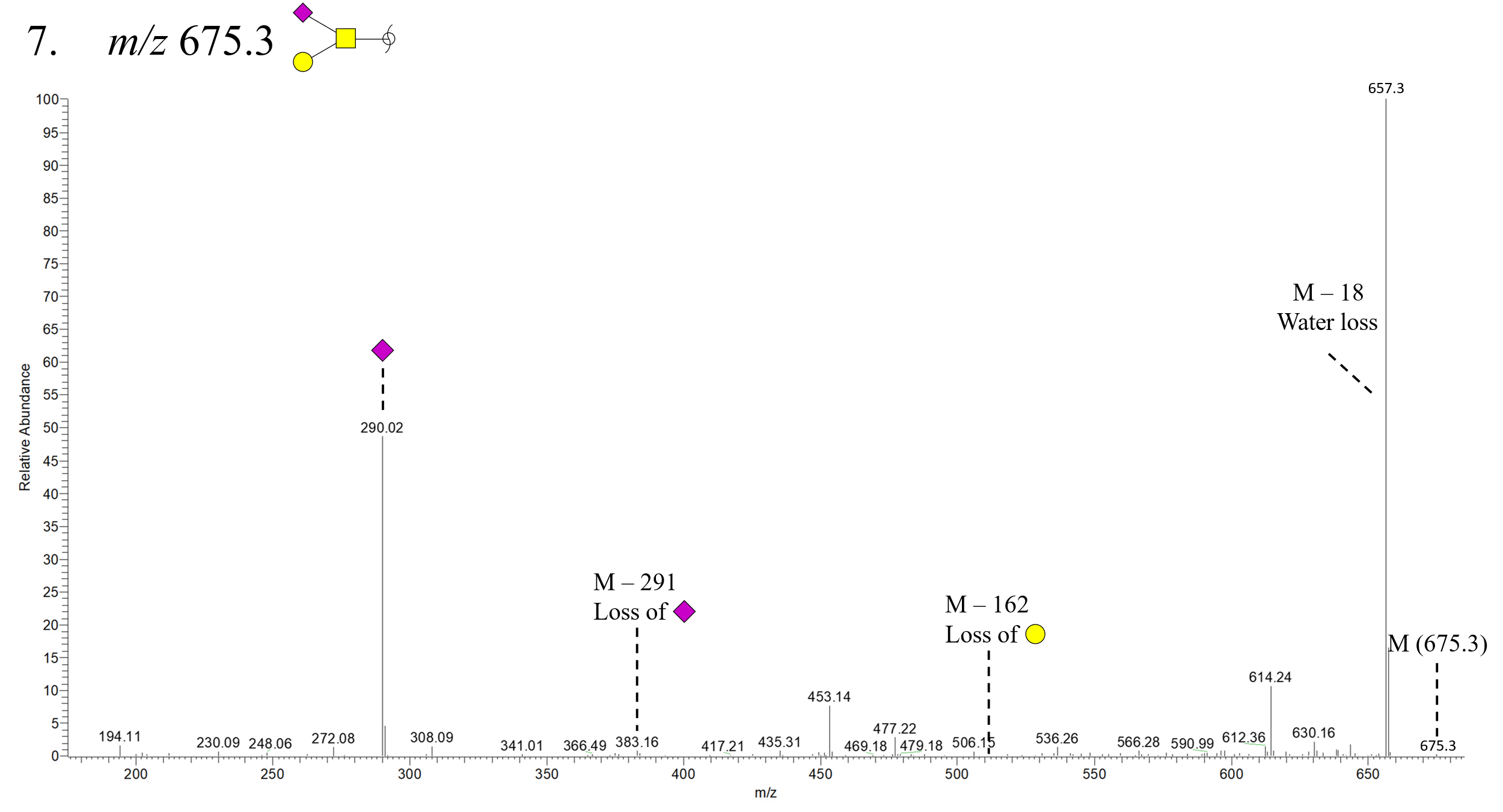

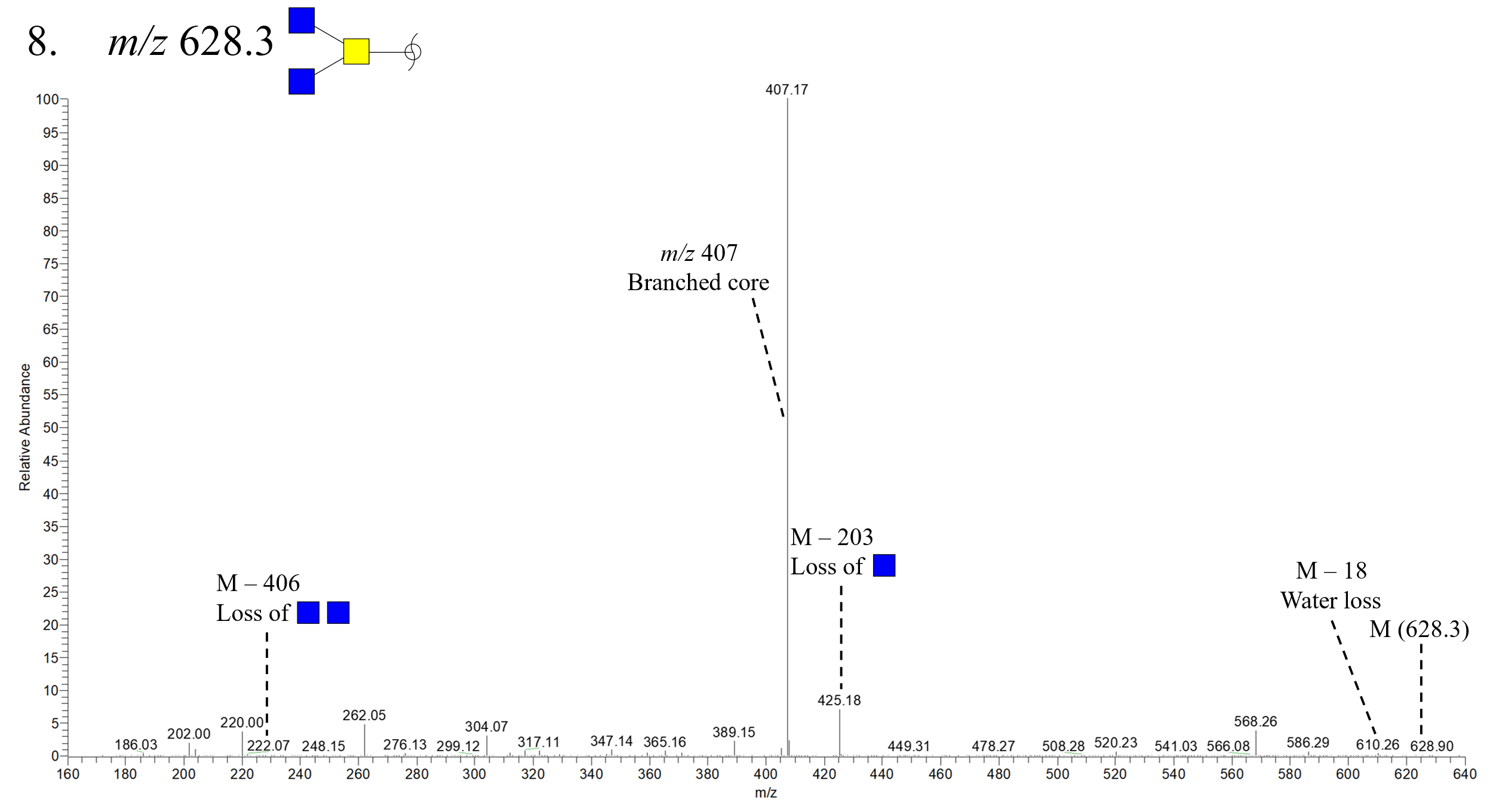

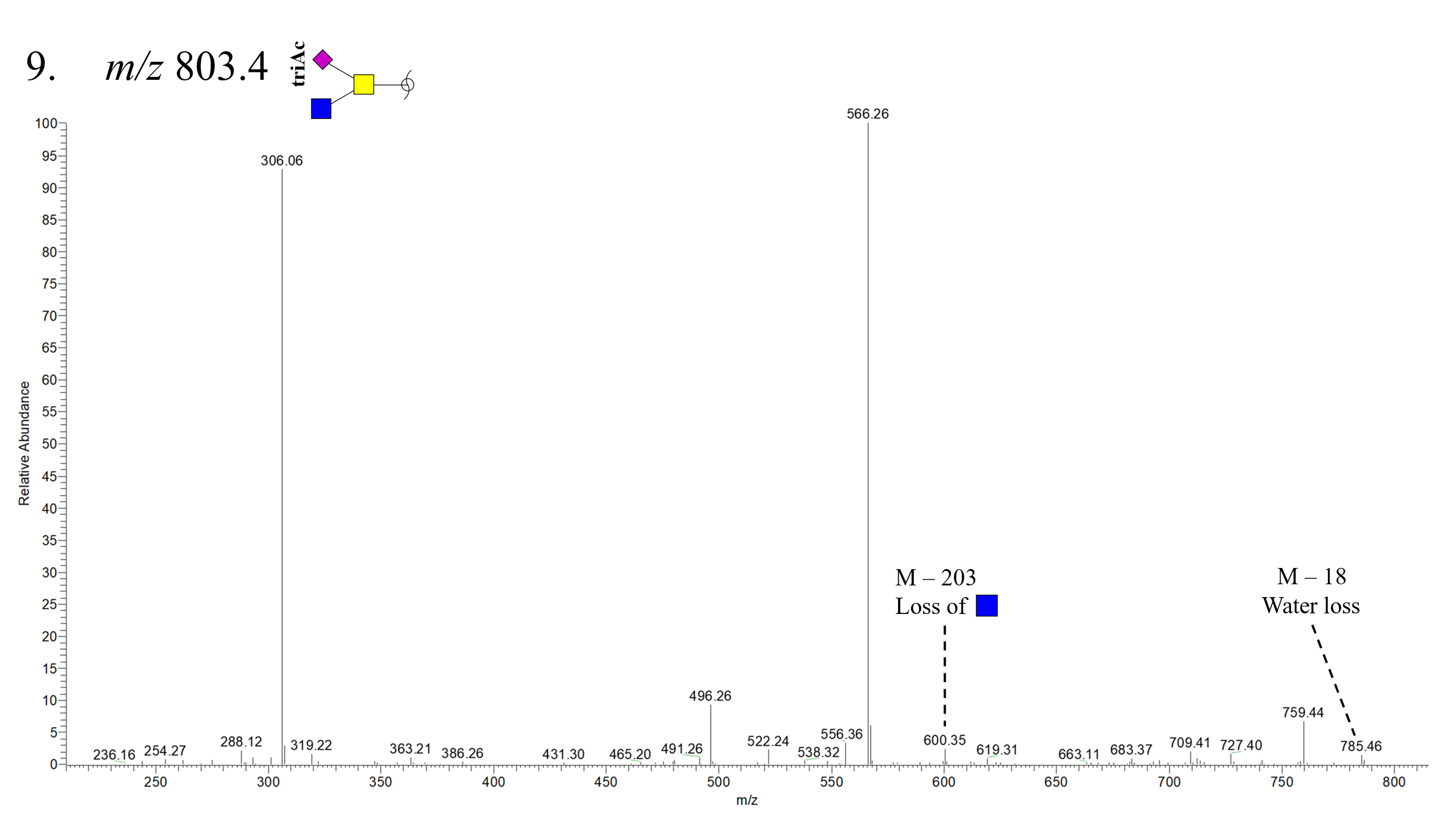


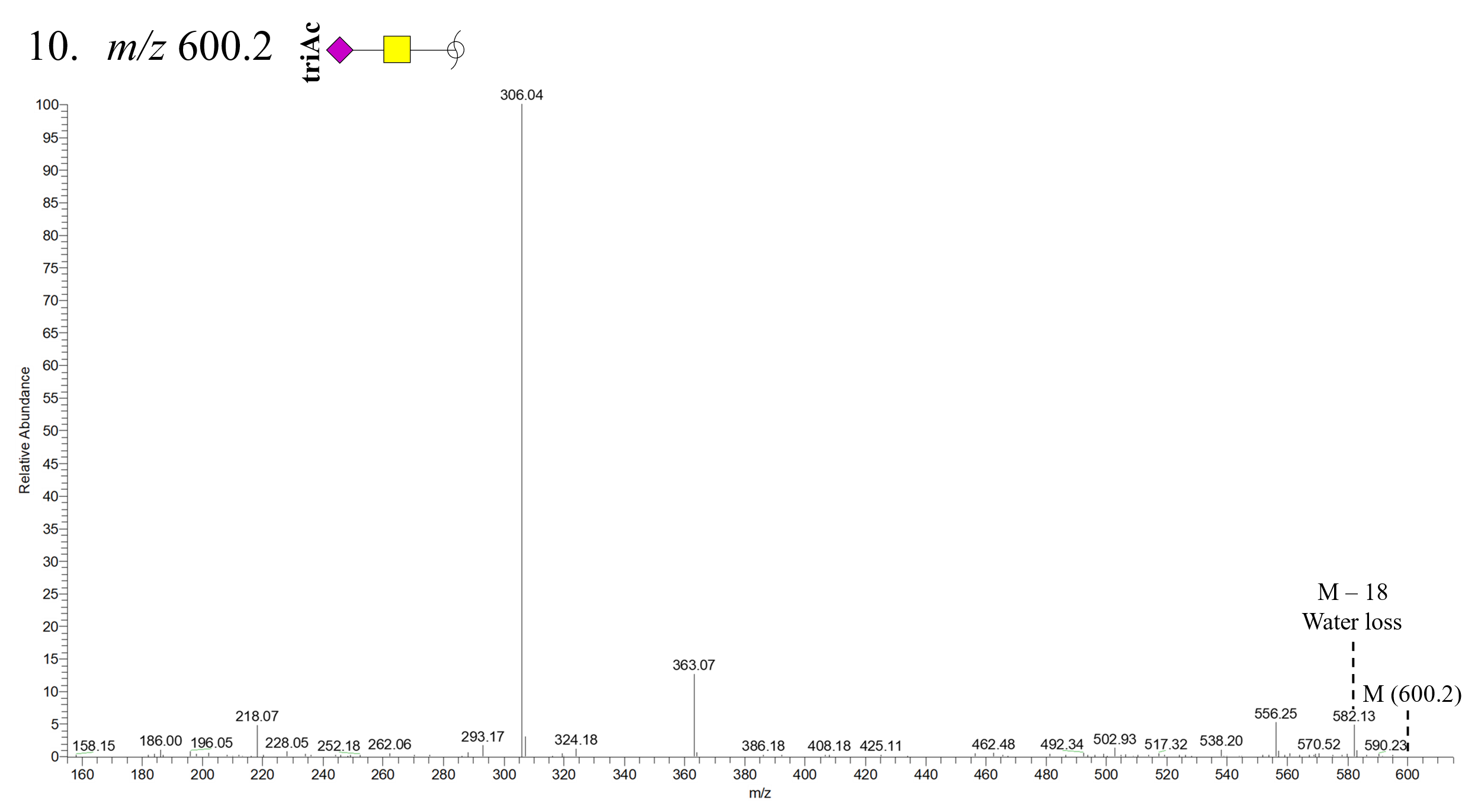

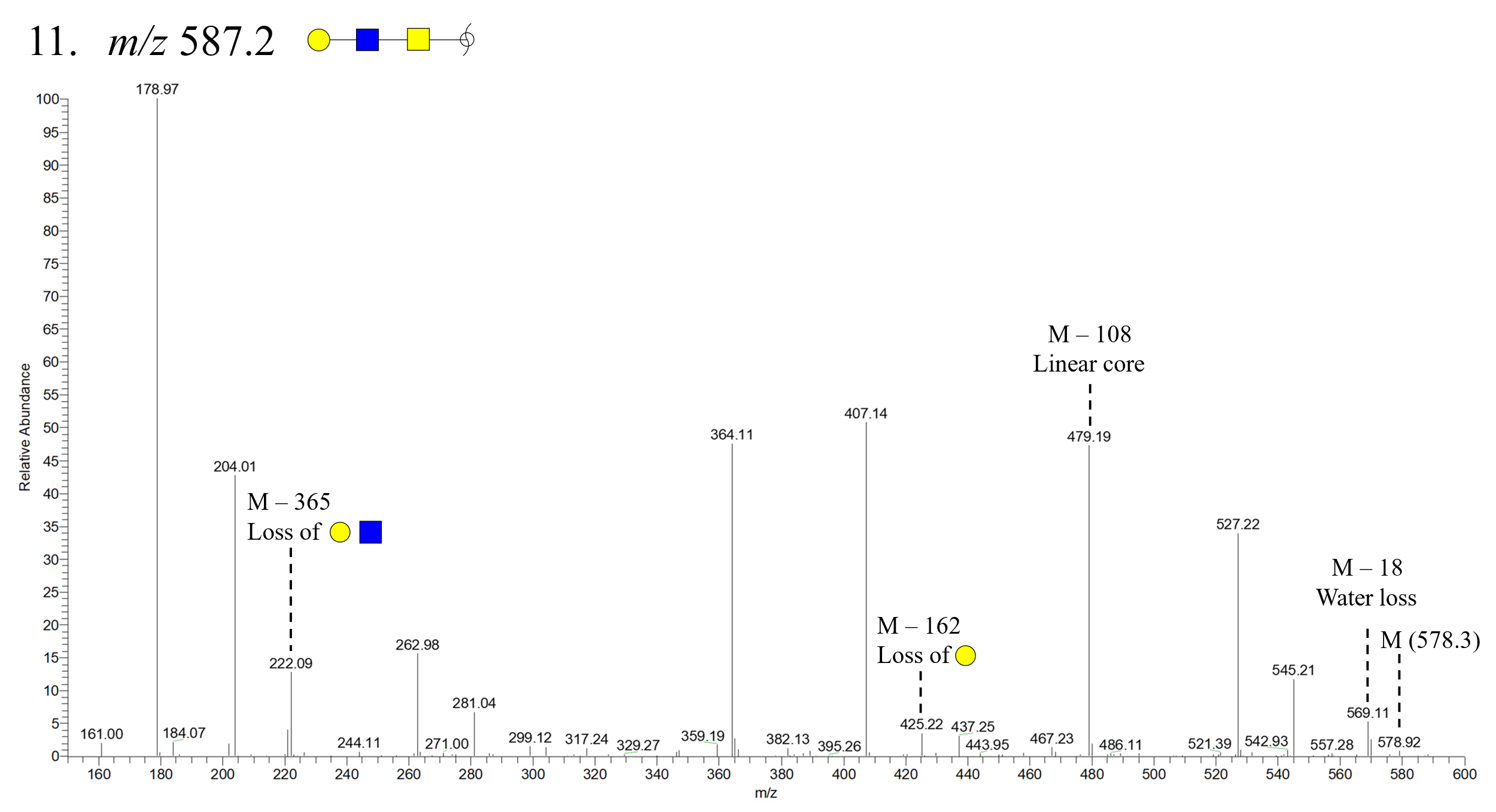

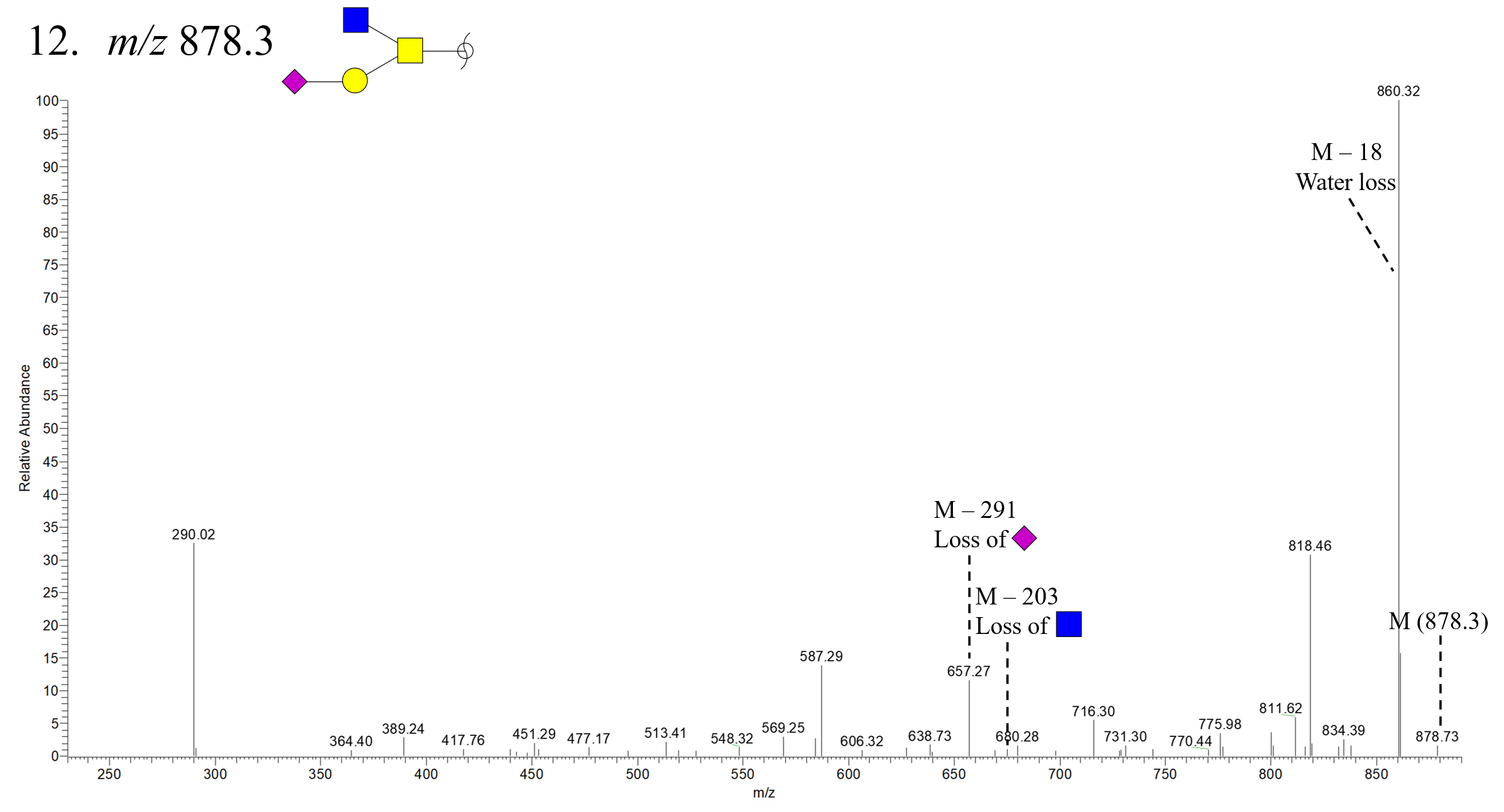


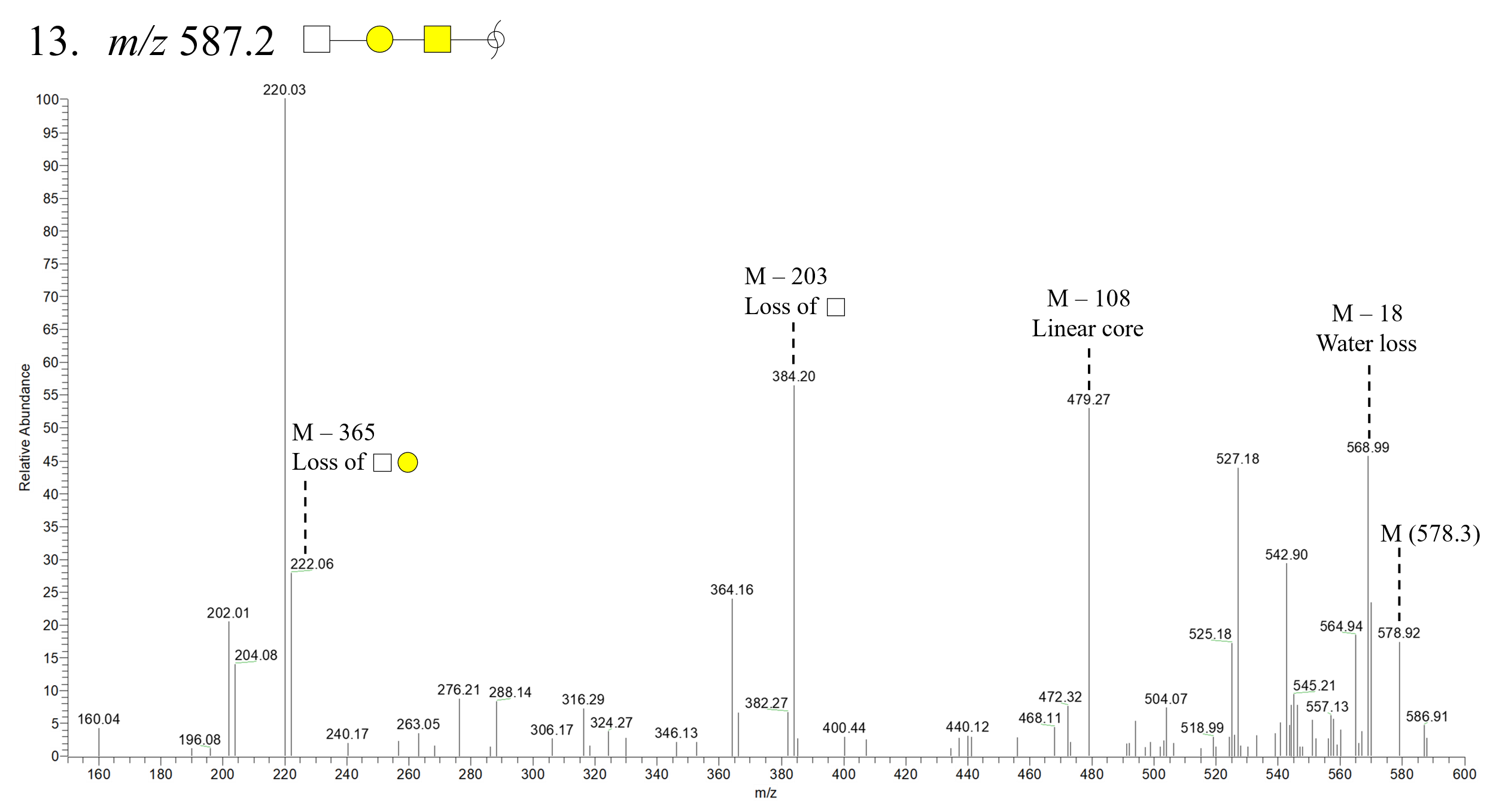


Figure S32. PGC-LC-MS/MS fragmentation spectra demonstrating the identification of the present *O-*glycans released from BSM. The fragmentation patterns are numbered (left corner) and these numbers correspond to the eluted peaks as indicated in Figure S31.

Figure S33. PGC-LC-MS elution patterns showing the degradation of *O*-glycans released from BSM during 24 h incubation with *R. torques* lysate.

Figure S34. PGC-LC-MS elution patterns showing the degradation of *O*-glycans released from BSM during 24 h incubation with *B. thetaiotaomicron* lysate.

Figure S35. PGC-LC-MS elution patterns showing the degradation of *O*-glycans released from BSM during 24 h incubation with *A. muciniphila*/*R. torques* lysate.

Figure S36. PGC-LC-MS elution patterns showing the degradation of *O*-glycans released from BSM after incubation with *A. muciniphila*/*B. thetaiotaomicron* lysate during 24 h.

Figure S37. PGC-LC-MS elution patterns showing the degradation of *O*-glycans released from BSM during 24 h incubation with *R. torques*/*B. thetaiotaomicron* lysate.

Figure S38. PGC-LC-MS elution patterns showing the degradation of *O*-glycans released from BSM during 24 h incubation with *A. muciniphila*/*R. torques*/*B. thetaiotaomicron* lysate.

Figure S39. PGC-LC-MS elution patterns showing the degradation of *O*-glycans released from BSM during 24 h incubation with the mucin-degrading synthetic community (MDSC) lysate.

Table S1. Literature overview of recognised glycoside hydrolyse (GH) families present in *A. muciniphila*, *R. torques*, and *B. thetaiotaomicron* with highlighted mucin glycan degrading CAZymes and their associated activities (Drula et al., 2022; Labourel et al., 2023; Raba & Luis, 2023). *bold GH families represent GH families involved in mucin degradation (mucin-degrading GH families).

| Species, phylum, and strain | Recognised GH families and **mucin-degrading GH families*** | Associated activity of **mucin-degrading GH families** |
| --- | --- | --- |
| *A. muciniphila*  Verrucomicrobiota  ATCC BAA-835^T^ | **2**, 3, 13, **16**, 18, **20**, 27, **29**, **31, 33, 35, 36**, 43, 57, 63, 77, **84, 89, 95**, 97, 105, **109**, 110, 123, 177, 181 | GH2: β-galactosidase |
|  |  | GH16: endo β1-4-galactosidase |
|  |  | GH20: β-*N*-acetylglucosaminidases |
|  |  | GH29: α-ʟ-fucosidase (preference α1-2/3/4 linkages) |
|  |  | GH31: α-*N*-acetylgalactosaminidase |
| *R. torques*  Bacillota  ATCC 27756^T^ | 1, **2**, 4, 13, 18, **20**, 25, **29, 31, 33, 36, 42**, 51, 73, 77, **84, 89, 95, 101, 112**, 123, **136** | GH33: sialidase (α2-3‐ and α2-6‐sialyl linkages) |
|  |  | GH35: β-galactosidase |
|  |  | GH36: α-galactosaminidase |
|  |  | GH42: β-galactosidase |
|  |  | GH84: β-*N*-acetylglucosaminidases |
| *B. thetaiotaomicron*  Bacteroidota  ATCC 29148^T^ | **2**, 3, 13, **16**, 18, **20**, 23, 25, 27, 28, **29**, 30, **31**, 32, **33, 35, 36**, 38, **42**, 43, 51, 53, 57, 66, 67, 73, 76, 77, 78, **84**, 88, **89**, 92, **95**, 97, 99, 105, 106, **109**, 110, 115, 116, 123, 125, 127, 130, 133, 137, 138, 139, 140, 141, 142, 143, 144, 146, 147, 154, 159, 163, 171, 182 | GH89: α-*N*-acetylglucosaminidase |
|  |  | GH95: α-ʟ-fucosidase (preference α1-2/3 linkages) |
|  |  | GH98: blood-group endo-β1-4-galactosidase |
|  |  | GH101: endo-enzymes specifically targeting core 1 structures |
|  |  | GH109: α-*N*-acetylgalactosaminidase |
|  |  | GH112: β1-3-galactosyl-*N*-acetylhexosamine phosphorylase |
|  |  | GH129: Tn antigen (GalNAcαSer), core 1 (Galβ1-3GalNAcαSer) |
|  |  | GH136: Lacto-*N*-biosidase (and recognises fucα1-4GlcNac) |

Table S2. Overview of carbohydrate esterases (CE), polysaccharide lyases (PL), auxiliarly activity enzymes (AA), carbohydrate-binding modules (CBM), and glycosyltransferases (GT) identified using proteomics of bacterial supernatant of *A. muciniphila* (Am), *R. torques* (Rt), and *B. thetaiotaomicron* (Bt) grown on porcine gastric mucin (PGM) for 24 h. Per CE, PL, AA, CBM, and GT family the identified genes are described.

| Enzyme function | Family number | Am | Rt | Bt |
| --- | --- | --- | --- | --- |
| Carbohydrate esterase (CE) | CE1 |  |  | BT_0152 |
|  |  |  |  | BT_0587 |
|  | CE4 | Amuc_1500 |  | BT_3246 |
|  |  | Amuc_1616 |  | BT_3387 |
|  | CE9 | Amuc_0948 | nagA | BT_0675 |
|  |  |  |  | BT_0676 |
|  | CE11 | lpxC |  | pxC/fabZ |
|  | CE19 | Amuc_1801 |  |  |
|  | CE20 | Amuc_0454 |  | BT_0457 |
| Polysaccharide lyase (PL) | PL8 |  |  | BT_3350 |
|  |  |  |  | chonabc |
|  | PL12 |  |  | BT_4657 |
|  |  |  |  | hepC |
|  | PL15 |  |  | BT_4652 |
|  | PL29 |  |  | BT_3328 |
|  | PL33 |  |  | BT_4410 |
| Auxillarly activity enzymes (AA) | AA4 | Amuc_1466 |  |  |
| Carbohydrate-binding modules (CBM) | CBM13 |  | RUMTOR_00862 |  |
|  | CBM20 |  |  | BT_2146 |
|  | CBM32 | Amuc_0290 | RUMTOR_01268 | BT_4050 |
|  |  | Amuc_0824 | RUMTOR_02806 | BT_3015 |
|  |  | Amuc_0392 |  | BT_3293 |
|  | CBM40 |  | RUMTOR_00151 |  |
|  |  |  | RUMTOR_02111 |  |
|  | CBM48 |  | glgB |  |
|  | CBM50 | Amuc_0821 |  |  |
|  | CBM51 | Amuc_1438 |  |  |

Table S2. (extended)

| Enzyme function | Family number | Am | Rt | Bt |
| --- | --- | --- | --- | --- |
| Glycosyltransferase (GT) | GT2 | Amuc_1581 | RUMTOR_00850 | BT_1183 |
|  |  | Amuc_1582 | RUMTOR_01223 | BT_4564 |
|  |  | Amuc_2081 | RUMTOR_01508 | BT_3622 |
|  |  | Amuc_2093 |  | BT_3388 |
|  |  | Amuc_2094 |  | BT_3366 |
|  |  | Amuc_0754 |  | BT_3365 |
|  |  | Amuc_0757 |  | BT_0251 |
|  |  | Amuc_0941 |  |  |
|  |  | Amuc_0943 |  |  |
|  |  | Amuc_0945 |  |  |
|  | GT4 | Amuc_1869 | RUMTOR_01500 | BT_4304 |
|  |  | Amuc_0442 |  | BT_2946 |
|  |  | Amuc_2082 |  | BT_4293 |
|  |  | Amuc_2083 |  |  |
|  |  | Amuc_2084 |  |  |
|  |  | Amuc_0638 |  |  |
|  |  | Amuc_2088 |  |  |
|  |  | Amuc_2089 |  |  |
|  |  | Amuc_2090 |  |  |
|  |  | Amuc_1077 |  |  |
|  | GT5 |  | glgA | BT_4307 |
|  | GT8 | Amuc_0753 |  |  |
|  |  | Amuc_0939 |  |  |
|  | GT9 | Amuc_0755 |  | BT_3362 |
|  | GT10 | Amuc_0760 |  |  |
|  |  | Amuc_0762 |  |  |
|  | GT11 | Amuc_2087 |  |  |
|  |  | Amuc_0942 |  |  |
|  | GT19 | Amuc_2087 |  | BT_4004 |
|  | GT26 | Amuc_0879 | RUMTOR_00847 |  |
|  | GT28 | Amuc_0973 | murG |  |
|  | GT30 | Amuc_0474 |  | BT_2747 |
|  | GT35 | Amuc_0235 | glgP | BT_1293 |
|  |  |  |  | BT_1100 |
|  | GT51 | Amuc_0177 | RUMTOR_00799 | BT_0743 |
|  |  | Amuc_2122 |  |  |
|  |  | Amuc_1241 |  |  |
|  | GT101 |  |  | BT_1179 |

Table S3. Raw proteomics label free quantification (LFQ) results (separate Excel file titled ‘’Supporting_Information_Table_S3_Proteomics.xlsx’’).

Table S4. Bactrial strains used and the associated number of 16S rRNA gene copies (Berkhout et al., 2024).

| Strain | Number of 16S rRNA gene copies |
| --- | --- |
| *Akkermansia muciniphila*DSM 22959^T^ | 3 |
| *Bacteroides caccae*DSM 19024^T^ | 5 |
| *Bacteroides fragilis*DSM 2151^T^ | 6 |
| *Bacteroides thetaiotaomicron*DSM 2079^T^ | 5 |
| *Phocaeicola vulgatus*ATCC 8482^T^ | 7 |
| *Ruminococcus gnavus* ATCC 29149^T^ | 5 |
| *Ruminococcus torques* ATCC 27756^T^ | 10 |
| *Anaerostipes caccae*DSM 14662^T^ | 4 |
| *Faecalibacterium duncaniae*DSM 17677^T^ | 6 |
| *Anaerobutyricum hallii*DSM 3353^T^ | 8 |
| *Agathobacter rectalis*ATCC 33656^T^ | 5 |
| *Roseburia intestinalis*DSM 14610^T^ | 6 |
| *Blautia hydrogenotrophica*DSM 10507^T^ | 5 |
| *Desulfovibrio piger*DSM 749^T^ | 7 |
| *Methanobrevibacter smithii*DSM 11975^T^ | 3 |

Table S5. Proteomes from Uniprot used as reference database for proteomics analysis (Bateman et al., 2025).

| Strain | Proteome |
| --- | --- |
| *Akkermansia muciniphila* DSM 22959^T^ | UP000001031 |
| *Bacteroides caccae* DSM 19024^T^ | UP000003325 |
| *Bacteroides fragilis* DSM 2151^T^ | UP000006731 |
| *Bacteroides thetaiotaomicron* DSM 2079^T^ | UP000001414 |
| *Phocaeicola vulgatus* ATCC 8482^T^ | UP000002861 |
| *Ruminococcus gnavus* ATCC 29149^T^ | UP000004410 |
| *Ruminococcus torques* ATCC 27756^T^ | UP000003577 |
| *Anaerostipes caccae* DSM 14662^T^ | UP000004935 |
| *Faecalibacterium duncaniae* DSM 17677^T^ | UP000004619 |
| *Agathobacter rectalis* ATCC 33656^T^ | UP000001477 |
| *Roseburia intestinalis* DSM 14610^T^ | UP000004828 |
| *Blautia hydrogenotrophica* DSM 10507^T^ | UP000003100 |
| *Desulfovibrio piger* DSM 749^T^ | UP000003676 |

**References**

Bateman, A., Martin, M.-J., Orchard, S., Magrane, M., Adesina, A., Ahmad, S., Bowler-Barnett, E. H., Bye-A-Jee, H., Carpentier, D., Denny, P., Fan, J., Garmiri, P., Gonzales, L. J. da C., Hussein, A., Ignatchenko, A., Insana, G., Ishtiaq, R., Joshi, V., Jyothi, D., … Zhang, J. (2025). UniProt: the universal protein knowledgebase in 2025. *Nucleic Acids Research*, *53*(D1), D609–D617. https://doi.org/10.1093/nar/gkae1010

Berkhout, M. D., Ioannou, A., de Ram, C., Boeren, S., Plugge, C. M., & Belzer, C. (2024). Mucin-driven ecological interactions in an *in vitro* synthetic community of human gut microbes. *Glycobiology*. https://doi.org/10.1093/glycob/cwae085/7816700

Drula, E., Garron, M. L., Dogan, S., Lombard, V., Henrissat, B., & Terrapon, N. (2022). The carbohydrate-active enzyme database: functions and literature. *Nucleic Acids Research*, *50*(D1), D571–D577. https://doi.org/10.1093/nar/gkab1045

Labourel, A., Parrou, J. L., Deraison, C., Mercier-Bonin, M., Lajus, S., & Potocki-Veronese, G. (2023). O-Mucin-degrading carbohydrate-active enzymes and their possible implication in inflammatory bowel diseases. *Essays in Biochemistry*, *67*(3), 331–344. https://doi.org/10.1042/EBC20220153

Raba, G., & Luis, A. S. (2023). Mucin utilization by gut microbiota: recent advances on characterization of key enzymes. *Essays in Biochemistry*, *67*(3), 345–353. https://doi.org/10.1042/EBC20220121
